# A phylogeny and revised classification of Squamata, including 4161 species of lizards and snakes

**DOI:** 10.1186/1471-2148-13-93

**Published:** 2013-04-29

**Authors:** R Alexander Pyron, Frank T Burbrink, John J Wiens

**Affiliations:** 1 Department of Biological Sciences, The George Washington University, 2023 G St. NW, Washington, DC 20052, USA; 2 Department of Biology, The Graduate School and University Center, The City University of New York, 365 5th Ave., New York, NY 10016, USA; 3 Department of Biology, The College of Staten Island, The City University of New York, 2800 Victory Blvd., Staten Island, NY 10314, USA; 4 Department of Ecology and Evolutionary Biology, University of Arizona, Tucson, AZ 85721-0088, USA

**Keywords:** Amphisbaenia, Lacertilia, Likelihood support measures, Missing data, Serpentes, Squamata, Phylogenetics, Reptilia, Supermatrices, Systematics

## Abstract

**Background:**

The extant squamates (>9400 known species of lizards and snakes) are one of the most diverse and conspicuous radiations of terrestrial vertebrates, but no studies have attempted to reconstruct a phylogeny for the group with large-scale taxon sampling. Such an estimate is invaluable for comparative evolutionary studies, and to address their classification. Here, we present the first large-scale phylogenetic estimate for Squamata.

**Results:**

The estimated phylogeny contains 4161 species, representing all currently recognized families and subfamilies. The analysis is based on up to 12896 base pairs of sequence data per species (average = 2497 bp) from 12 genes, including seven nuclear loci (BDNF, c-mos, NT3, PDC, R35, RAG-1, and RAG-2), and five mitochondrial genes (12S, 16S, cytochrome *b*, ND2, and ND4). The tree provides important confirmation for recent estimates of higher-level squamate phylogeny based on molecular data (but with more limited taxon sampling), estimates that are very different from previous morphology-based hypotheses. The tree also includes many relationships that differ from previous molecular estimates and many that differ from traditional taxonomy.

**Conclusions:**

We present a new large-scale phylogeny of squamate reptiles that should be a valuable resource for future comparative studies. We also present a revised classification of squamates at the family and subfamily level to bring the taxonomy more in line with the new phylogenetic hypothesis. This classification includes new, resurrected, and modified subfamilies within gymnophthalmid and scincid lizards, and boid, colubrid, and lamprophiid snakes.

## Background

Squamate reptiles (lizards, snakes, and amphisbaenians ["worm lizards"]) are among the most diverse radiations of terrestrial vertebrates. Squamata includes more than 9400 species as of December 2012 [1]. The rate of new species descriptions shows no signs of slowing, with a record 168 new species described in 2012 [1], greater than the highest yearly rates of the 18th and 19th centuries (e.g. 1758, 118 species; 1854, 144 species [1]). Squamates are presently found on every continent except Antarctica, and in the Indian and Pacific Oceans, and span many diverse ecologies and body forms, from limbless burrowers to arboreal gliders (summarized in [2-4]).

Squamates are key study organisms in numerous fields, from evolution, ecology, and behavior [3] to medicine [5,6] and applied physics [7]. They have also been the focus of many pioneering studies using phylogenies to address questions about trait evolution (e.g. [8,9]). Phylogenies are now recognized as being integral to all comparative studies of squamate biology (e.g. [10,11]). However, hypotheses about squamate phylogeny have changed radically in recent years [12], especially when comparing trees generated from morphological [13-15] and molecular data [16-20]. Furthermore, despite extensive work on squamate phylogeny at all taxonomic levels, a large-scale phylogeny (i.e. including thousands of species and multiple genes) has never been attempted using morphological or molecular data.

Squamate phylogenetics has changed radically in the last 10 years, revealing major conflicts between the results of morphological and molecular analyses [12]. Early estimates of squamate phylogeny [21] and recent studies based on morphological data [13-15,22] consistently supported a basal division between Iguania (including chameleons, agamids, and iguanids, *sensu lato*), and Scleroglossa, which comprises all other squamates (including skinks, geckos, snakes, and amphisbaenians). Within Scleroglossa, many phylogenetic analyses of morphological data have also supported a clade containing limb-reduced taxa, including various combinations of snakes, dibamids, amphisbaenians, and (in some analyses) limb-reduced skinks and anguids [13-15,19,22], though some of these authors also acknowledged that this clade was likely erroneous.

In contrast, recent molecular analyses have estimated very different relationships. Novel arrangements include placement of dibamids and gekkotans near the root of the squamate tree, a sister-group relationship between amphisbaenians and lacertids, and a clade (Toxicofera) uniting Iguania with snakes and anguimorphs within Scleroglossa [16-20,23,24]. These molecular results (and the results of combined morphological and molecular analyses) suggest that some estimates of squamate phylogeny based on morphology may have been misled, especially by convergence associated with adaptations to burrowing [19]. However, there have also been disagreements among molecular studies, such as placement of dibamids relative to gekkotans and other squamates, and relationships among snakes, iguanians, and anguimorphs (e.g. [17,20]).

Analyses of higher-level squamate relationships based on molecular data have so far included relatively few (less than 200) species, and none have included representatives from all described families and subfamilies [17-20,23,24]. This limited taxon sampling makes existing molecular phylogenies difficult to use for broad-scale comparative studies, with some exceptions based on supertrees [10,11]. In addition, limited taxon sampling is potentially a serious issue for phylogenetic accuracy [25-28]. Thus, an analysis with extensive taxon sampling is critically important to test hypotheses based on molecular datasets with more limited sampling, and to provide a framework for comparative analyses.

Despite the lack of a large-scale phylogeny across squamates, recent molecular studies have produced phylogenetic estimates for many of the major groups of squamates, including iguanian lizards [29-34], higher-level snake groups [35-37], typhlopoid snakes [38,39], colubroid snakes [40-46], booid snakes [47,48], scincid lizards [49-52], gekkotan lizards [53-60], teiioid lizards [61-64], lacertid lizards [65-69], and amphisbaenians [70,71]. These studies have done an outstanding job of clarifying the phylogeny and taxonomy of these groups, but many were limited in some ways by the number of characters and taxa that they sampled (and which were available at the time for sequencing).

Here, we present a phylogenetic estimate for Squamata based on combining much of the existing sequence data for squamate reptiles, using the increasingly well-established supermatrix approach [41,72-77]. We present a new phylogenetic estimate including 4161 squamate species. The dataset includes up to 12896 bp per species from 12 genes (7 nuclear, 5 mitochondrial). We include species from all currently described families and subfamilies. In terms of species sampled, this is 5 times larger than any previous phylogeny for any one squamate group [30,41], 3 times larger than the largest supertree estimate [11], and 25 times larger than the largest molecular study of higher-level squamate relationships [20]. While we did not sequence any new taxa specifically for this project, much of the data in the combined matrix were generated in our labs or from our previous collaborative projects [16,19,20,34,36,37,41,44],[78-82], including thousands of gene sequences from hundreds of species (>550 species; ~13% of the total).

The supermatrix approach can provide a relatively comprehensive phylogeny, and uncover novel relationships not seen in any of the separate analyses in which the data were generated. Such novel relationships can be revealed via three primary mechanisms. First, different studies may have each sampled different species from a given group for the same genes, and combining these data may reveal novel relationships not apparent in the separate analyses. Second, different studies may have used different genetic markers for the same taxa, and combining these markers can dramatically increase character sampling, potentially revealing new relationships and providing stronger support for previous hypotheses. Third, even for clades that were previously studied using complete taxon sampling and multiple loci, novel relationships may be revealed by including these lineages with other related groups in a large-scale phylogeny.

The estimated tree and branch-lengths should be useful for comparative studies of squamate biology. However, this phylogeny is based on a supermatrix with extensive missing data (mean = 81% per species). Some authors have suggested that matrices with missing cells may yield misleading estimates of topology, support, and branch lengths [83]. Nevertheless, most empirical and simulation studies have not found this to be the case, at least for topology and support [41,73,84,85]. Though fewer studies have examined the effects of missing data on branch lengths [44,86,87], these also suggest that missing data do not strongly impact estimates. Here, we test whether branch lengths for terminal taxa are related to their completeness.

In general, our results corroborate those of many recent molecular studies with regard to higher-level relationships, species-level relationships, and the monophyly, composition, and relationships of most families, subfamilies, and genera. However, our results differ from previous estimates for some groups, and reveal (or corroborate) numerous problems in the existing classification of squamates. We therefore provide a conservative, updated classification of extant squamates at the family and subfamily level based on the new phylogeny, while highlighting problematic taxonomy at the genus level, without making changes. The generic composition of all families and subfamilies under our revised taxonomy are provided in Appendix I.

We note dozens of problems in the genus-level taxonomy suggested by our tree, but we acknowledge in advance that we do not provide a comprehensive review of the previous literature dealing with all these taxonomic issues (this would require a monographic treatment). Similarly, we do not attempt to fix these genus-level problems here, as most will require more extensive taxon (and potentially character) sampling to adequately resolve.

Throughout the paper, we address only extant squamates. Squamata also includes numerous extinct species classified in both extant and extinct families, subfamilies, and genera. Relationships and classification of extinct squamates based on morphological data from fossils have been addressed by numerous authors (e.g. [14,15,19,22,88-93]). A classification based only on living taxa may create some problems for classifying fossil taxa, but these can be addressed in future studies that integrate molecular and fossil data [19,86].

## Results

### Supermatrix phylogeny

**Figure 1:**
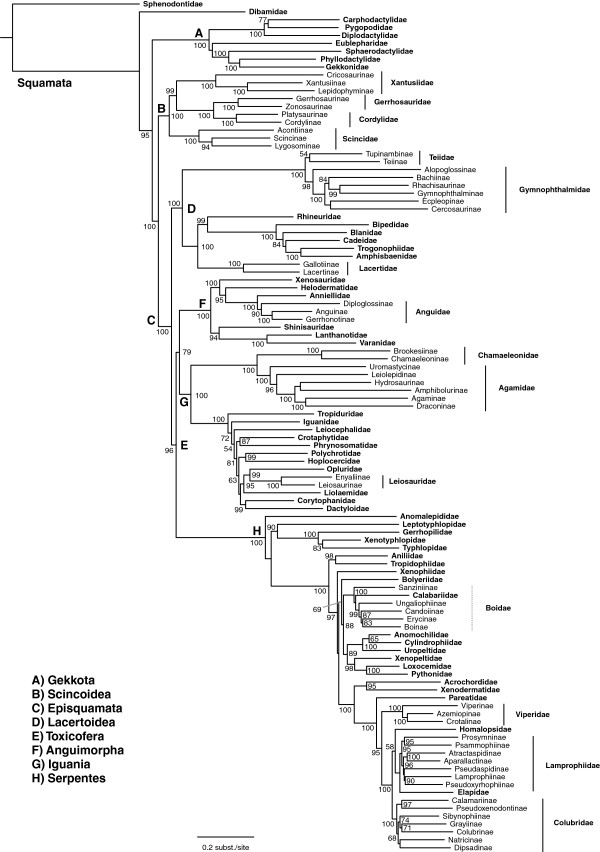
**Higher-level squamate phylogeny.** Skeletal representation of the 4161-species tree from maximum-likelihood analysis of 12 genes, with tips representing families and subfamilies (following our taxonomic revision; species considered *incertae sedis* are not shown). Numbers at nodes are SHL values greater than 50%. The full tree is presented in Figures 2, 3, 4, 5, 6, 7, 8, 9, 10, 11, 12, 13, 14, 15, 16, 17, 18, 19, 20, 21, 22, 23, 24, 25, 26, 27, 28.

**Figure 2:**
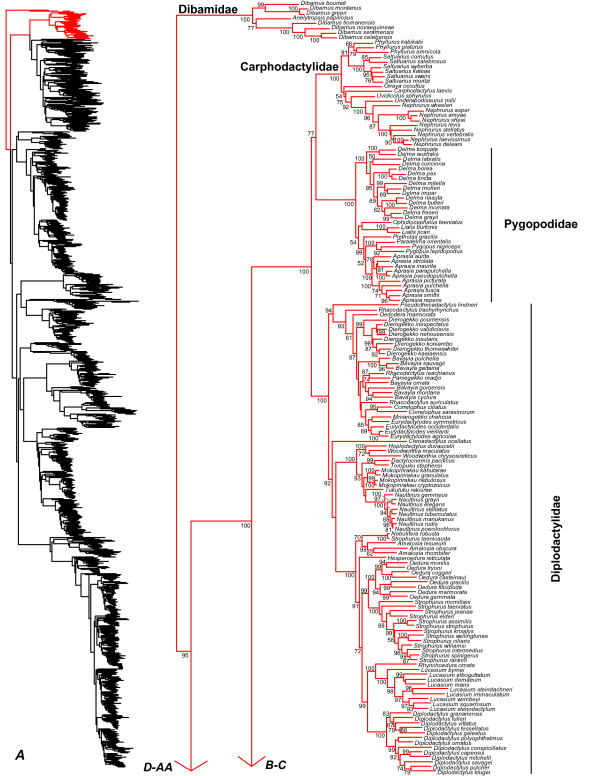
**Species-level squamate phylogeny.** Large-scale maximum likelihood estimate of squamate phylogeny, containing 4161 species. Numbers at nodes are SHL values greater than 50%. A skeletal version of this tree is presented in Figure 1. Bold italic letters indicate figure panels (**A-AA**). Within panels, branch lengths are proportional to expected substitutions per site, but the relative scale differs between panels.

**Figure 3:**
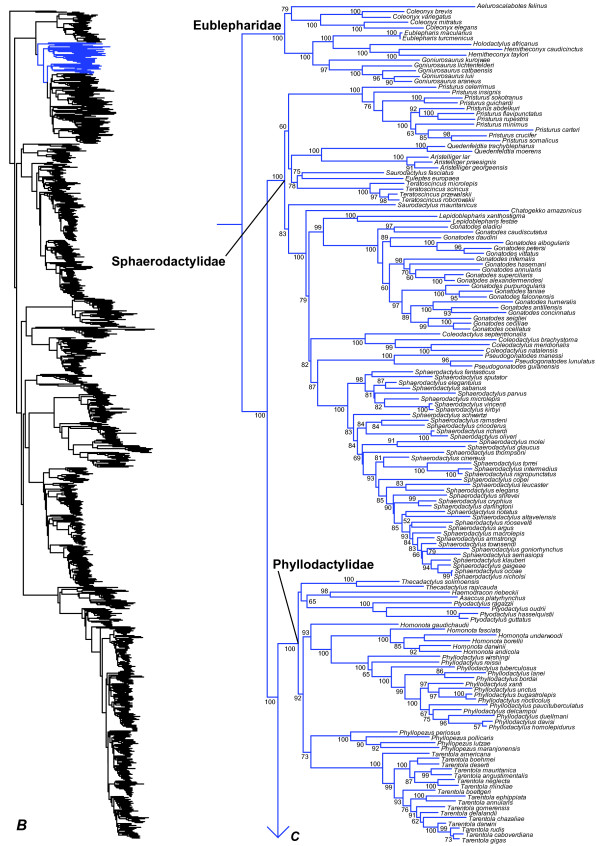
Species-level squamate phylogeny continued (B).

**Figure 4:**
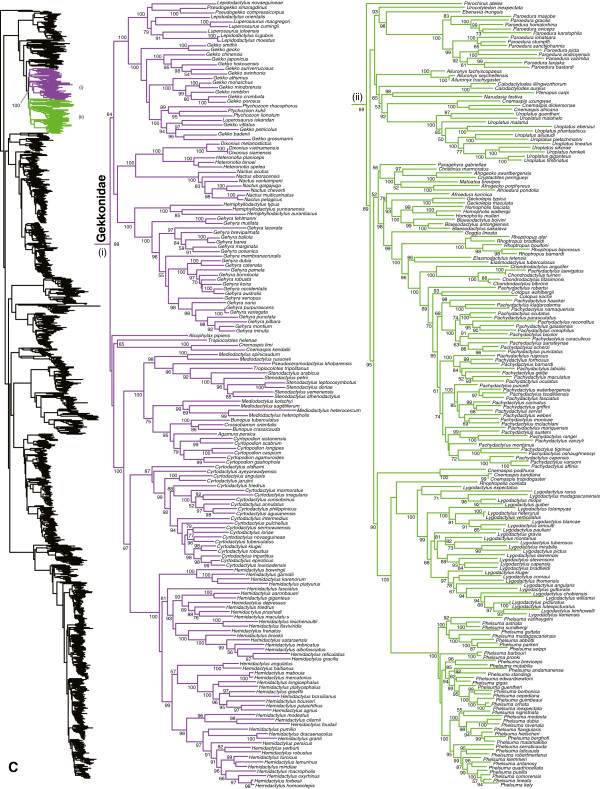
Species-level squamate phylogeny continued (C).

**Figure 5:**
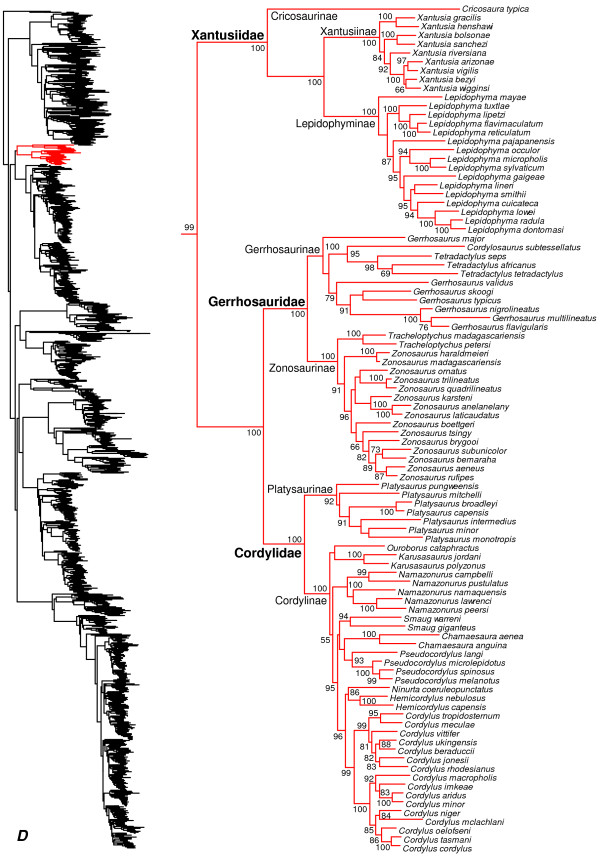
Species-level squamate phylogeny continued (D).

**Figure 6:**
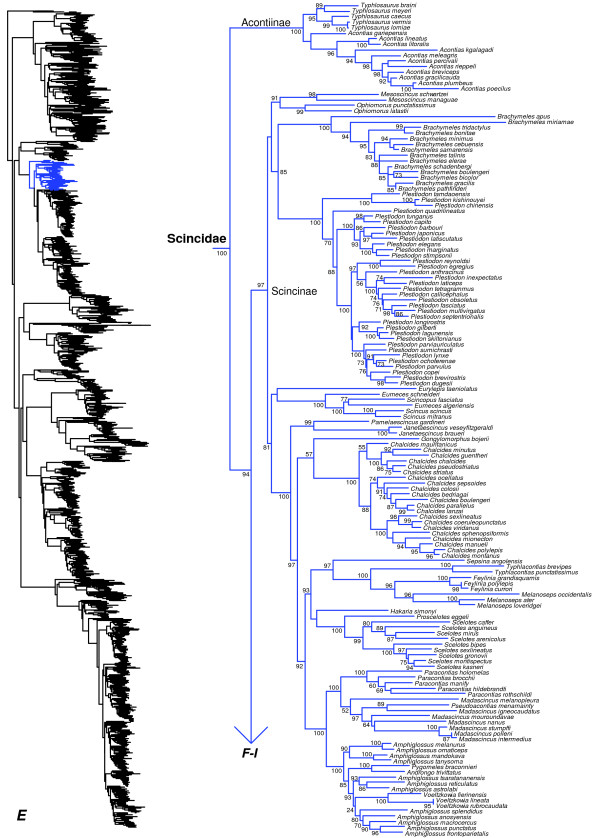
Species-level squamate phylogeny continued (E).

**Figure 7:**
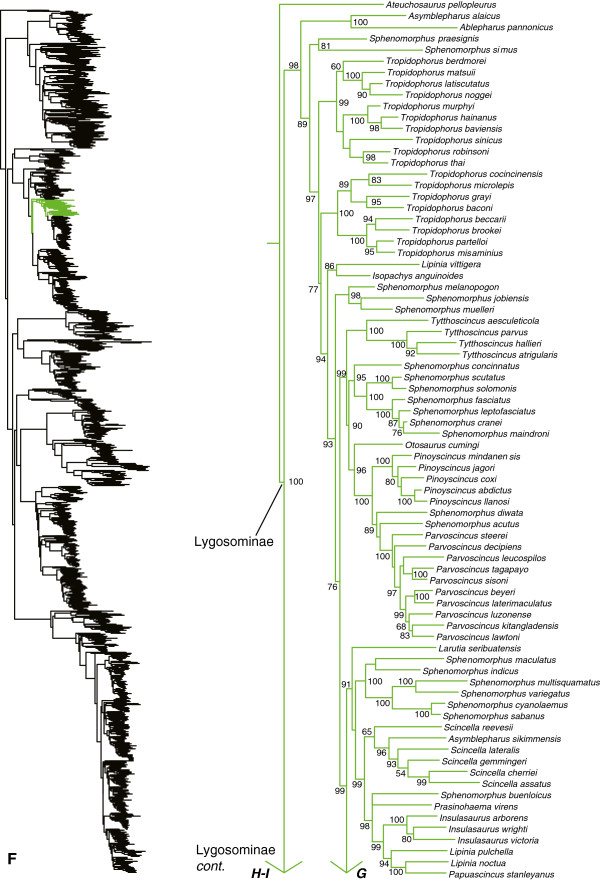
Species-level squamate phylogeny continued (F).

**Figure 8:**
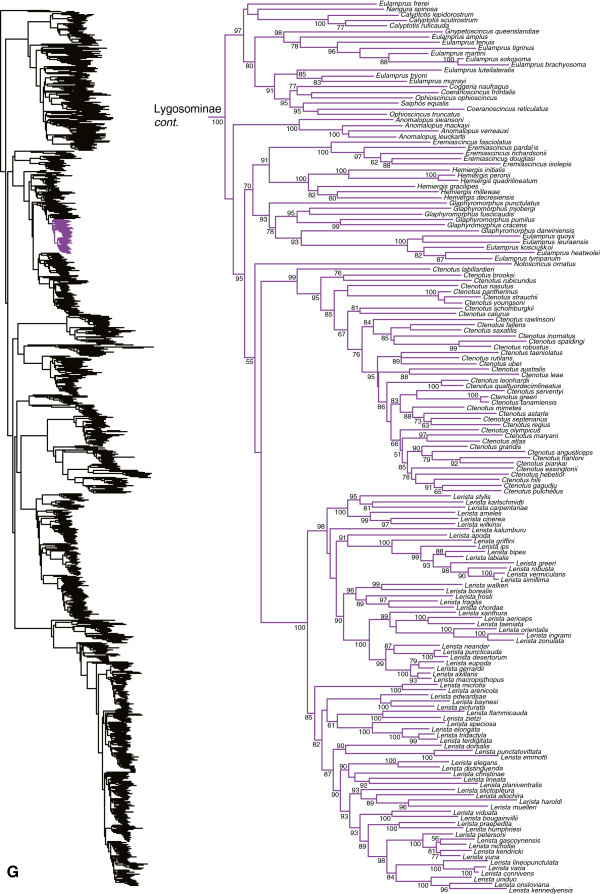
Species-level squamate phylogeny continued (G).

**Figure 9:**
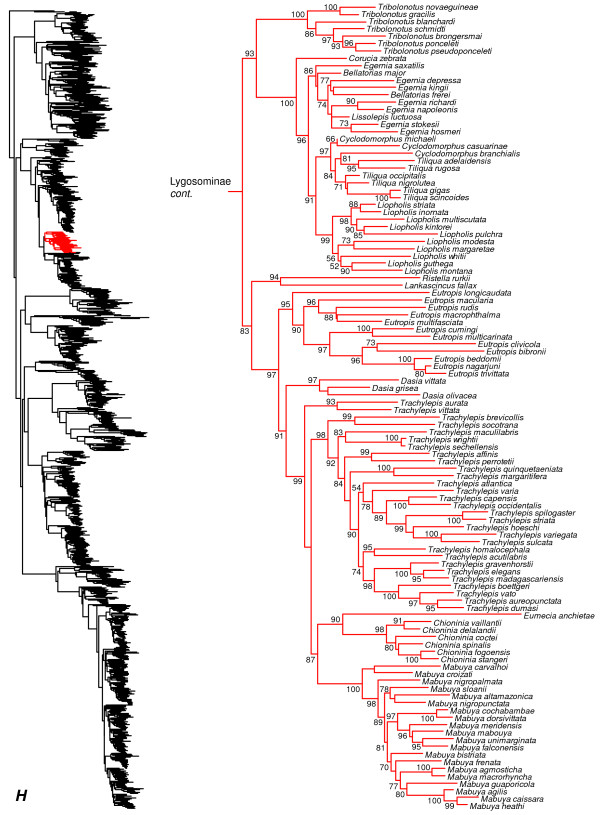
Species-level squamate phylogeny continued (H).

**Figure 10:**
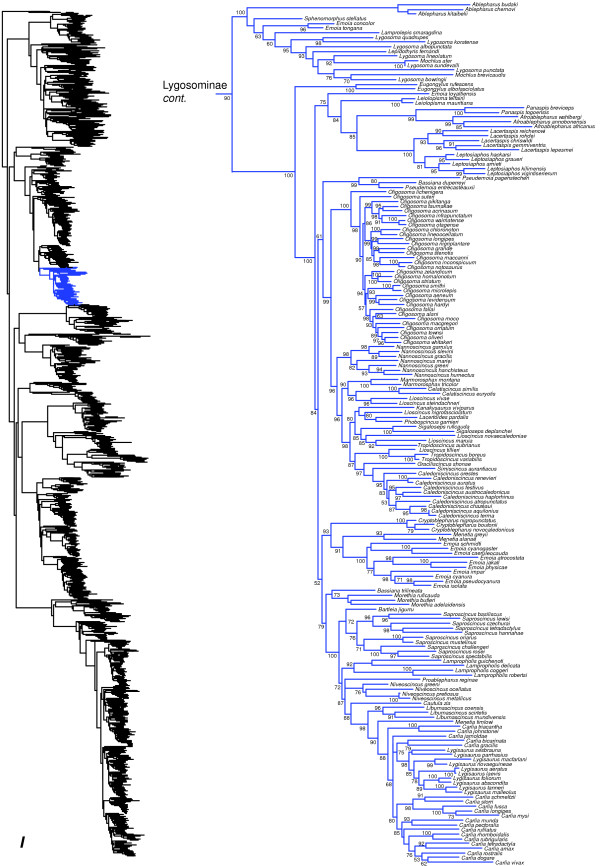
Species-level squamate phylogeny continued (I).

**Figure 11:**
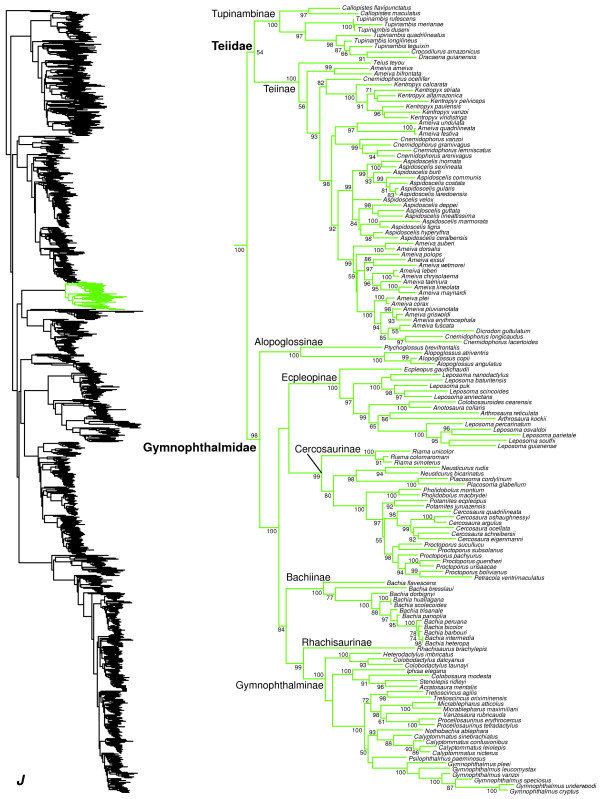
Species-level squamate phylogeny continued (J).

**Figure 12:**
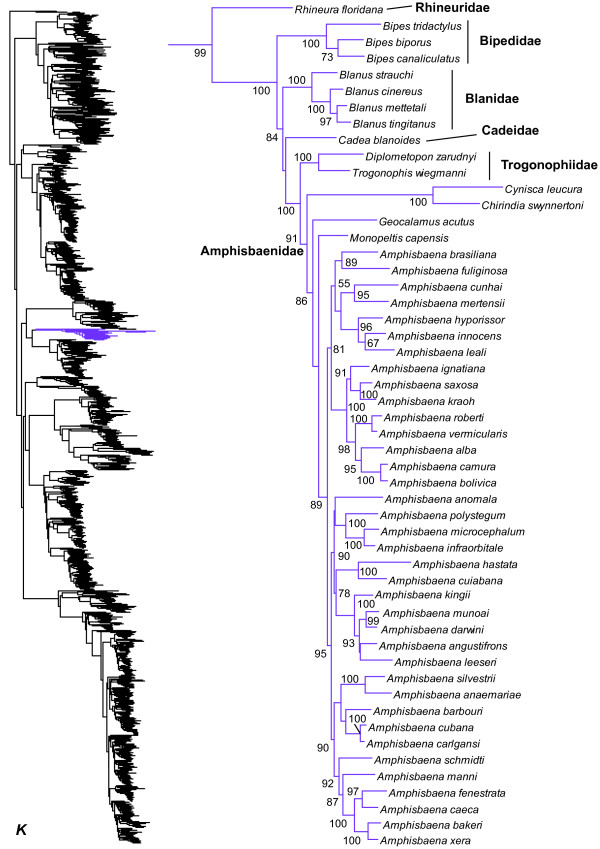
Species-level squamate phylogeny continued (K).

**Figure 13:**
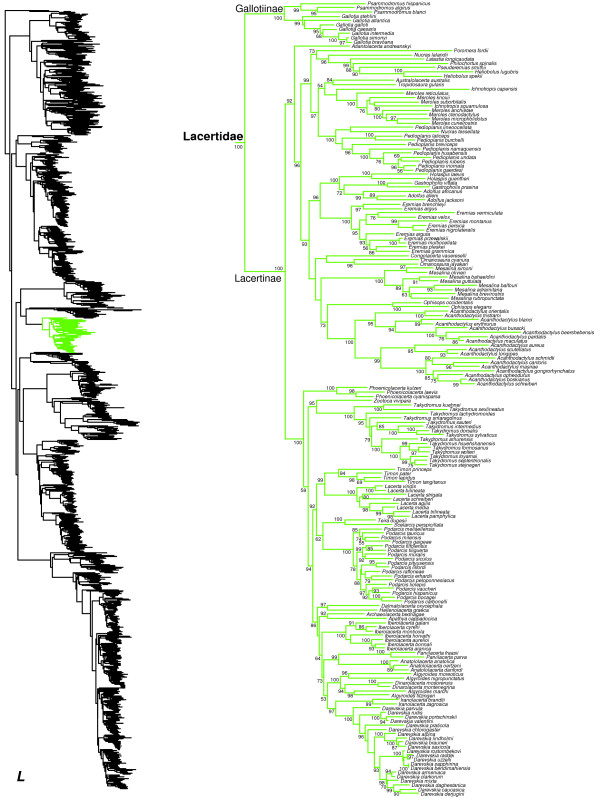
Species-level squamate phylogeny continued (L).

**Figure 14:**
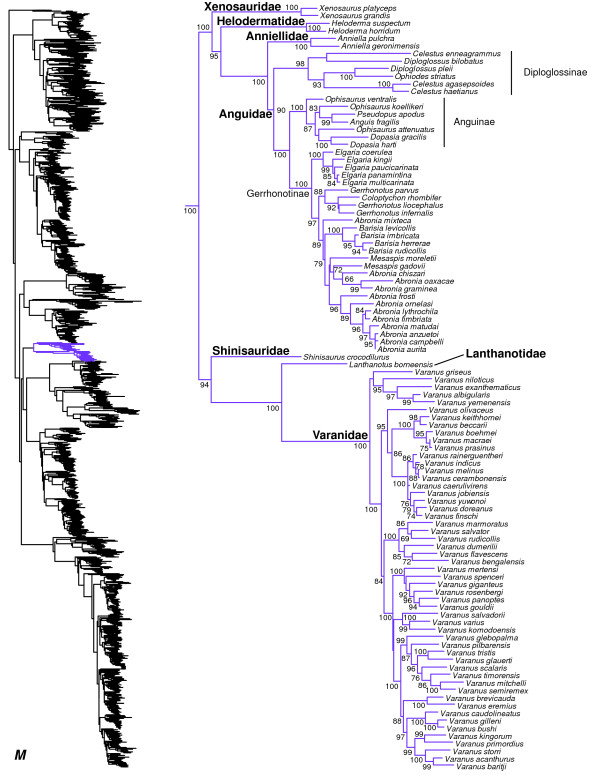
Species-level squamate phylogeny continued (M).

**Figure 15:**
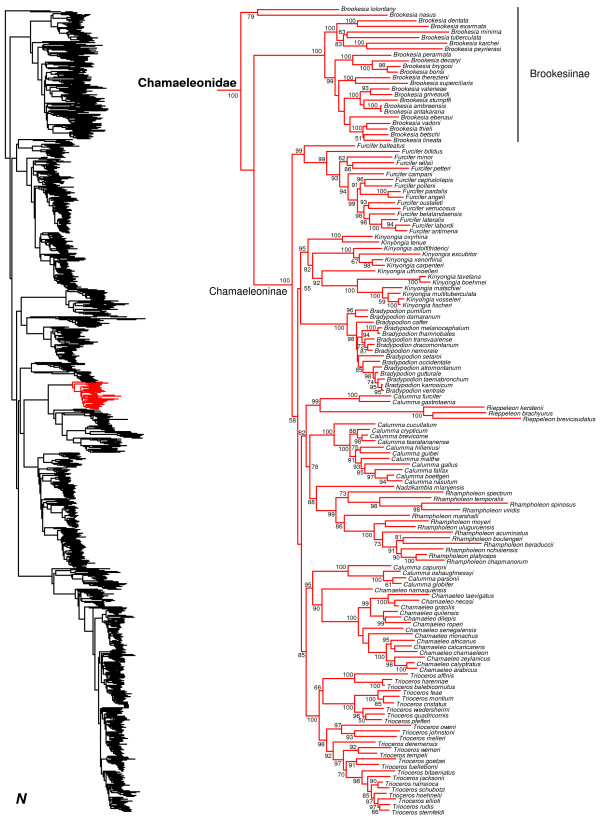
Species-level squamate phylogeny continued (N).

**Figure 16:**
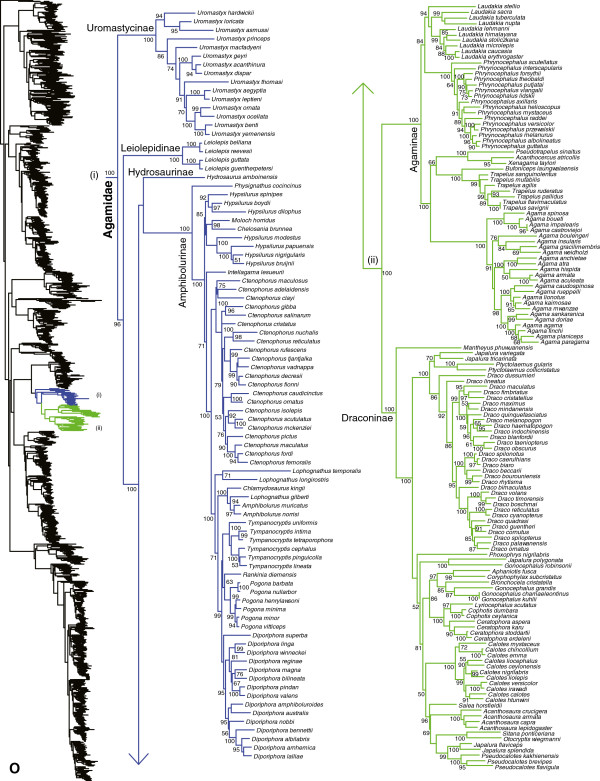
Species-level squamate phylogeny continued (O).

**Figure 17:**
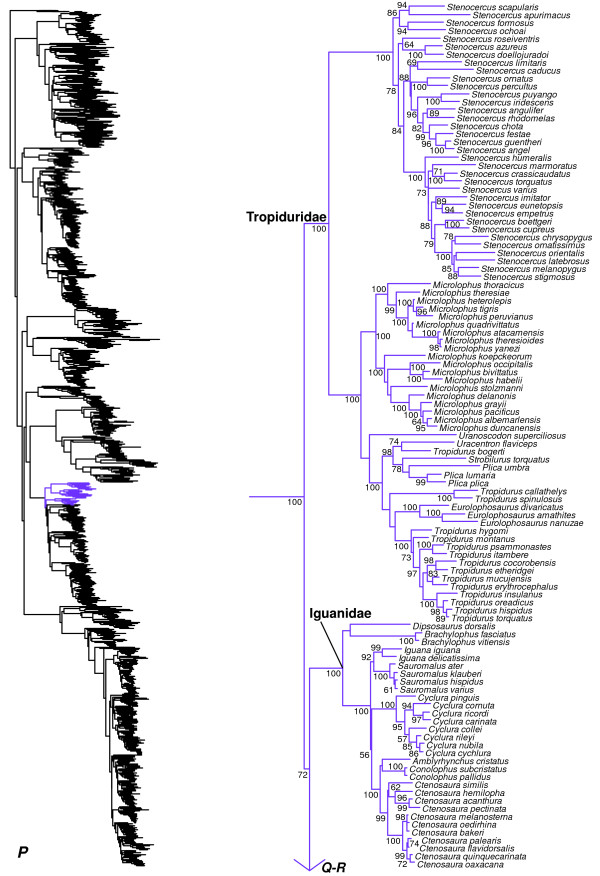
Species-level squamate phylogeny continued (P).

**Figure 18:**
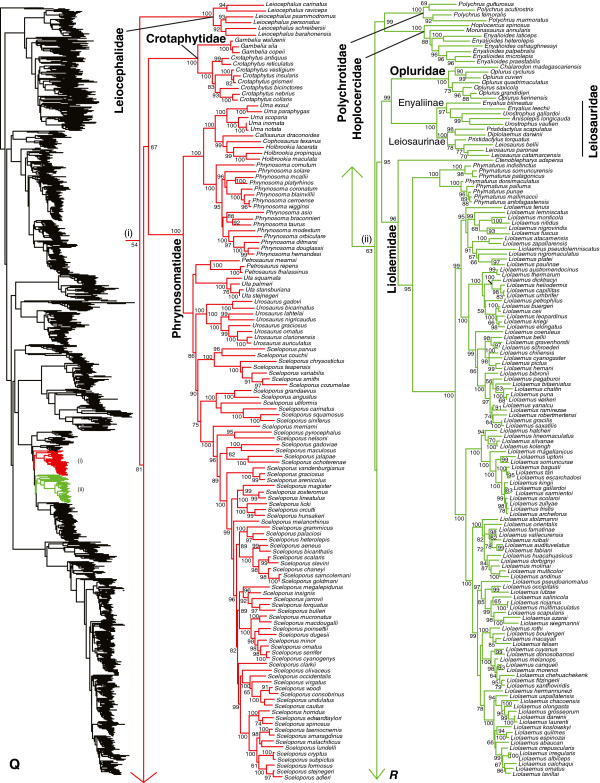
Species-level squamate phylogeny continued (Q).

**Figure 19:**
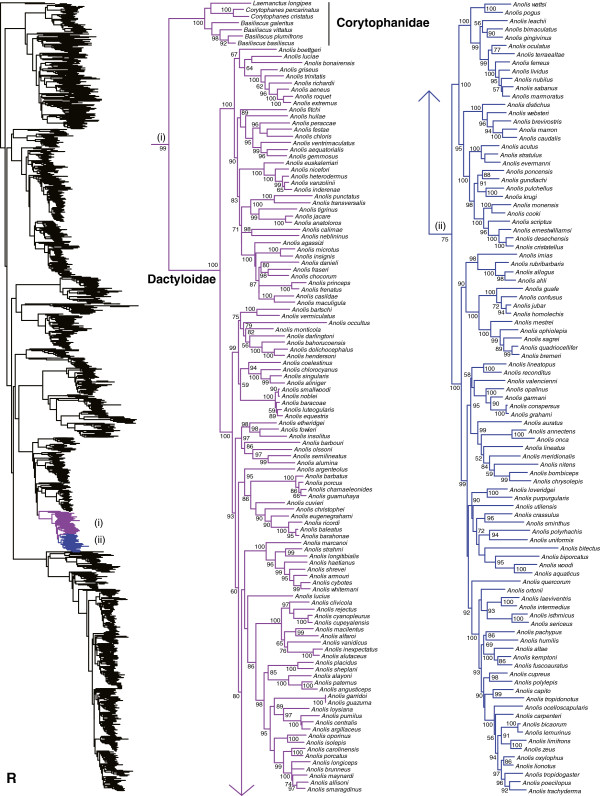
Species-level squamate phylogeny continued (R).

**Figure 20:**
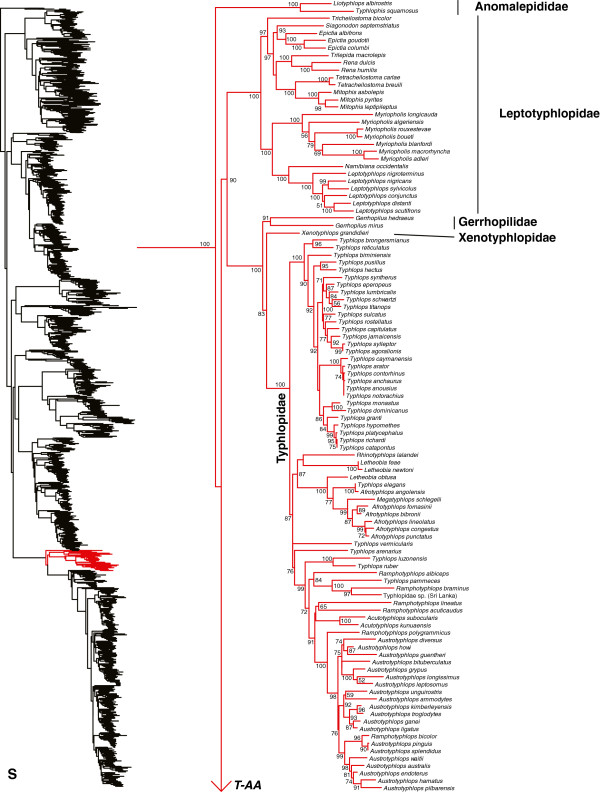
Species-level squamate phylogeny continued (S).

**Figure 21:**
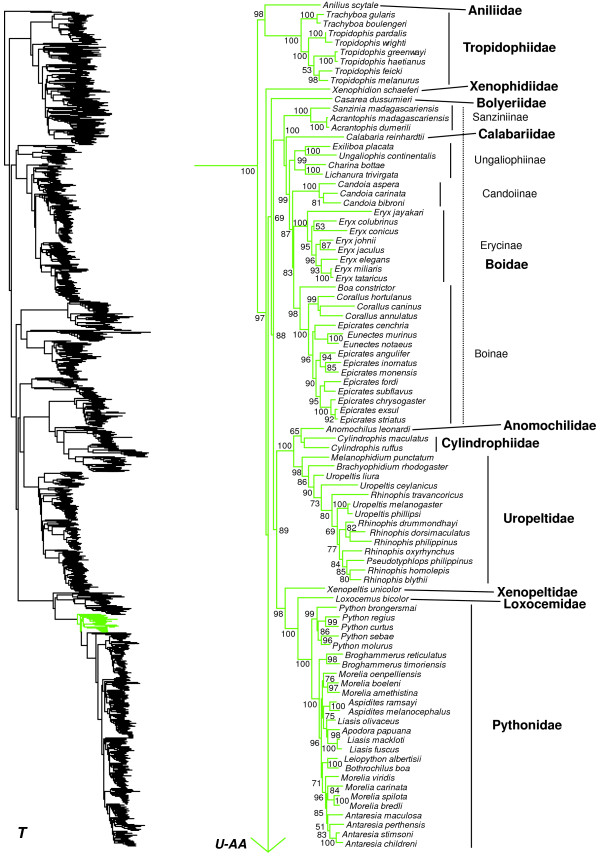
Species-level squamate phylogeny continued (T).

**Figure 22:**
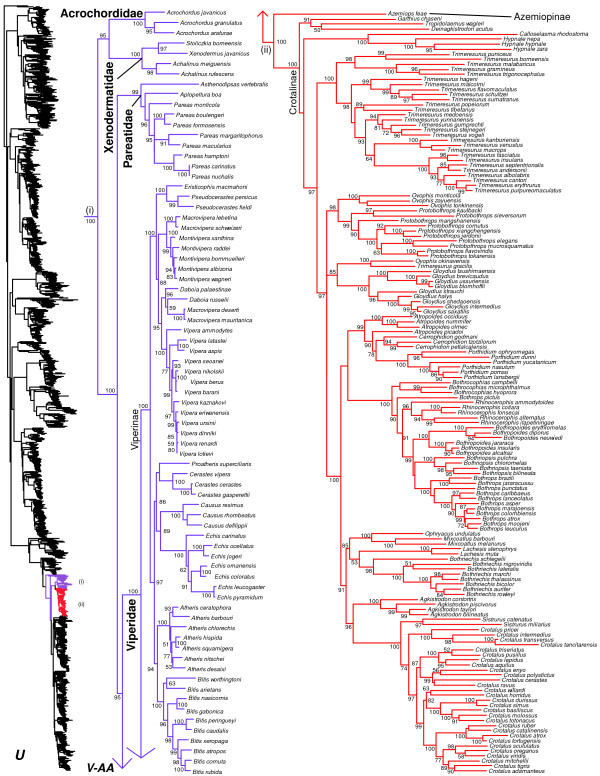
Species-level squamate phylogeny continued (U).

**Figure 23:**
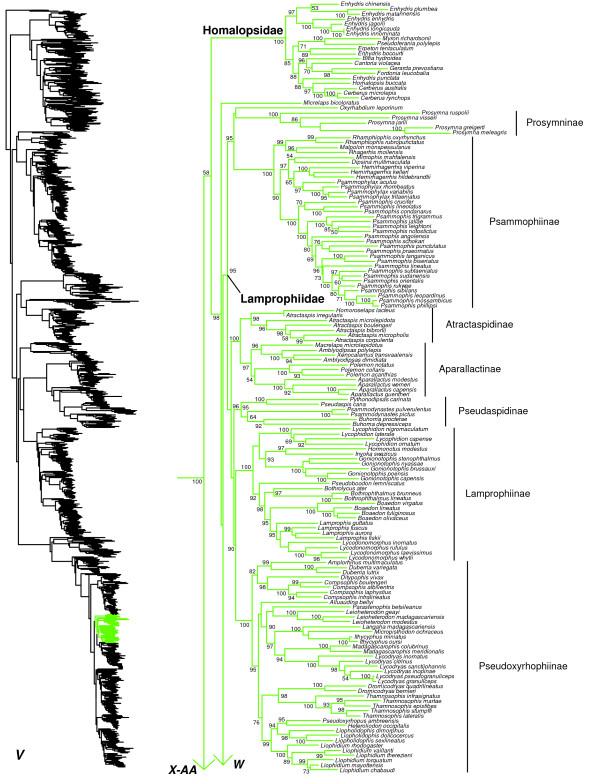
Species-level squamate phylogeny continued (V).

**Figure 24:**
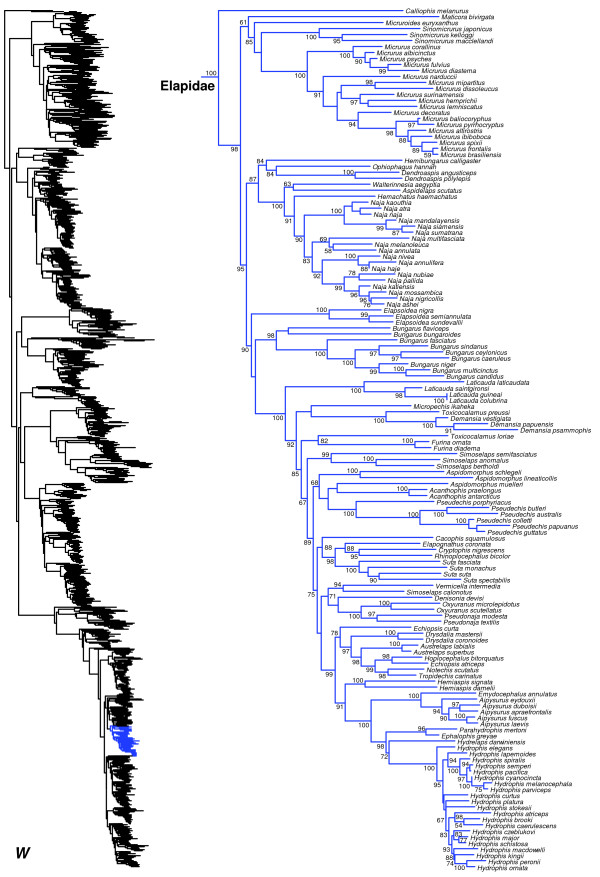
Species-level squamate phylogeny continued (W).

**Figure 25:**
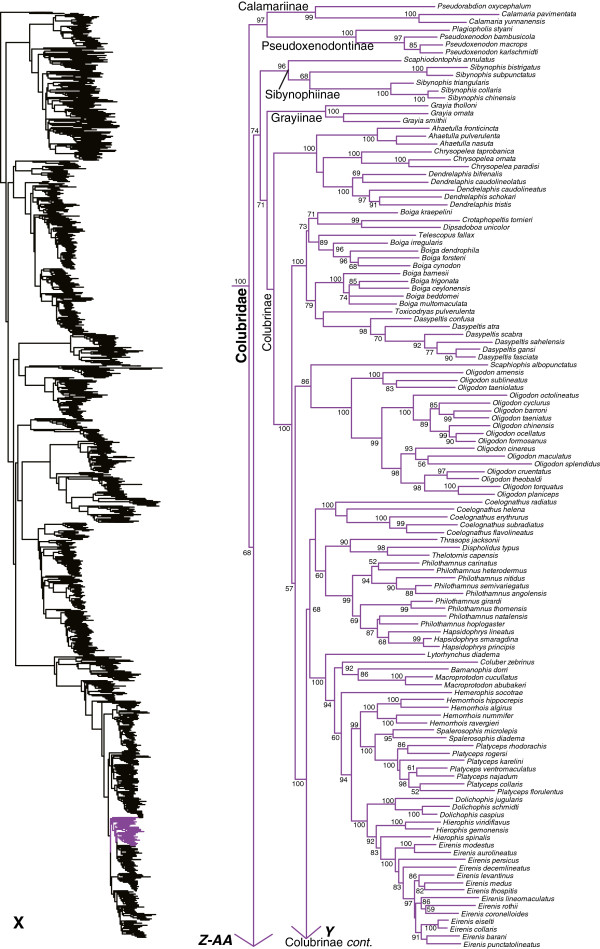
Species-level squamate phylogeny continued (X).

**Figure 26:**
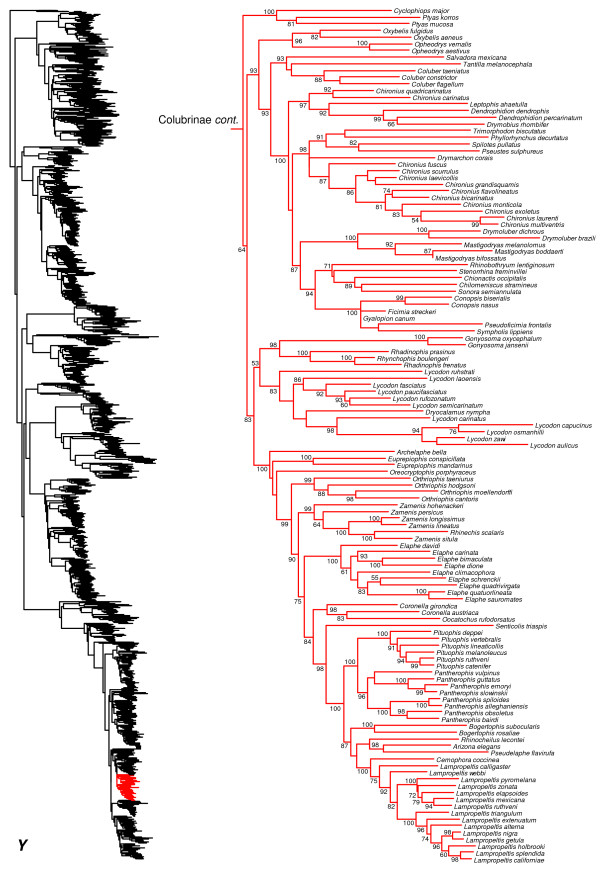
Species-level squamate phylogeny continued (Y).

**Figure 27:**
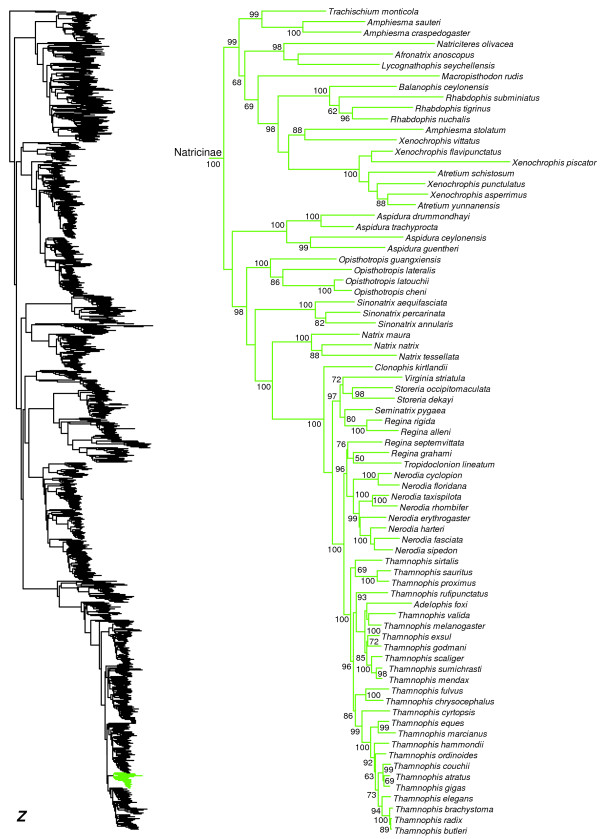
Species-level squamate phylogeny continued (Z).

**Figure 28:**
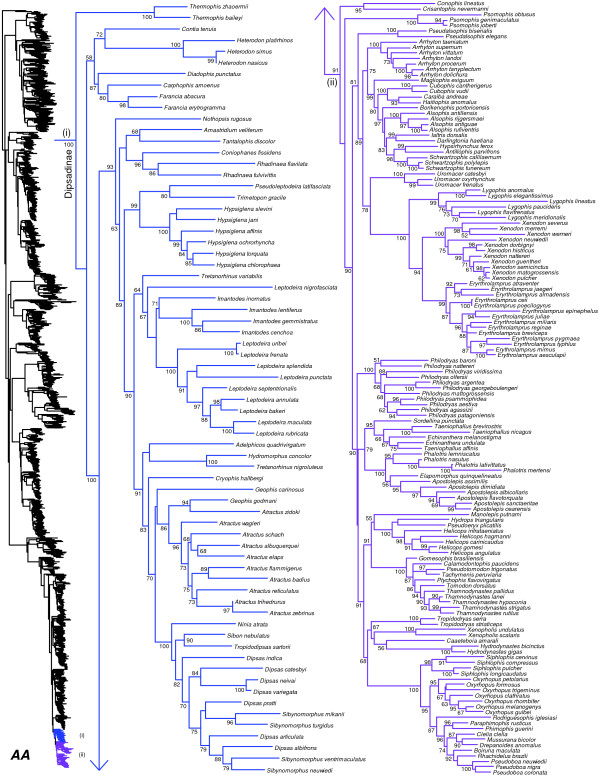
Species-level squamate phylogeny continued (AA).

We generated the final tree (lnL = −2609551.07) using Maximum Likelihood (ML) in RAxMLv7.2.8. Support was assessed using the non-parametric Shimodaira-Hasegawa-Like (SHL) implementation of the approximate likelihood-ratio test (aLRT; see [94]). The tree and data matrix are available in NEXUS format in DataDryad repository 10.5061/dryad.82h0m and as Additional file 1: Data File S1. A skeletal representation of the tree (excluding several species which are *incertae sedis*) is shown in Figure 1. The full species-level phylogeny (minus the outgroup *Sphenodon*) is shown in Figures 2, 3, 4, 5, 6, 7, 8, 9, 10, 11, 12, 13, 14, 15, 16, 17, 18, 19, 20, 21, 22, 23, 24, 25, 26, 27, 28. The analysis yields a generally well-supported phylogenetic estimate for squamates (i.e. 70% of nodes have SHL values >85, indicating they are strongly supported). There is no relationship between proportional completeness (bp of non-missing data in species / 12896 bp of complete data) and branch length (*r* = −0.29, *P* = 0.14) for terminal taxa, strongly suggesting that the estimated branch lengths are not consistently biased by missing data.

### Higher-level relationships

Our tree (Figure 1) is broadly congruent with most previous molecular studies of higher-level squamate phylogeny using both nuclear data and combined nuclear and mitochondrial data (e.g. [16-20]), providing important confirmation of previous molecular studies based on more limited taxon sampling. Specifically we support (Figure 1): (i) the placement of dibamids and gekkotans near the base of the tree (Figure 1A); (ii) a sister-group relationship between Scincoidea (scincids, cordylids, gerrhosaurids, and xantusiids; Figure 1B) and a clade (Episquamata; Figure 1C) containing the rest of the squamates excluding dibamids and gekkotans; (iii) Lacertoidea (lacertids, amphisbaenians, teiids, and gymnophthalmids; Figure 1D), and (iv) a clade (Toxicofera; Figure 1E) containing anguimorphs (Figure 1F), iguanians (Figure 1G), and snakes (Figure 1H) as the sister taxon to Lacertoidea.

These relationships are strongly supported in general (Figure 1), but differ sharply from most trees based on morphological data [13-15,19,22,95]. Nevertheless, many clades found in previous morphological taxonomies and phylogenies are also present in this tree in some form, including Amphisbaenia, Anguimorpha, Gekkota, Iguania, Lacertoidea (but including amphisbaenians), Scincoidea, Serpentes, and many families and subfamilies. In contrast, the relationships among these groups differ strongly between molecular analyses [17-20] and morphological analyses [14,15]. Our results demonstrate that this incongruence is not explained by limited taxon sampling in the molecular data sets. In fact, our species-level sampling is far more extensive than in any morphological analyses (e.g. [14,15]), by an order of magnitude.

We find that the basal squamate relationships are strongly supported in our tree. The family Dibamidae is the sister group to all other squamates, and Gekkota is the sister group to all squamates excluding Dibamidae (Figure 1), as in some previous studies (e.g. [16,18]). Other recent molecular analyses have also placed Dibamidae near the squamate root, but differed in placing it as either the sister taxon to all squamates excluding Gekkota [17], or the sister- group of Gekkota [19,20]. Our results also corroborate that the New World genus *Anelytropsis* is nested within the Old World genus *Dibamus*[96], but the associated branches are weakly supported (Figure 2).

### Gekkota

Within Gekkota, we corroborate both earlier morphological [97] and recent molecular estimates [55,56,59,98] in supporting a clade containing the Australian radiation of "diplodactylid" geckos (Carphodactylidae and Diplodactylidae) and the snakelike pygopodids (Figures 1, 2). As in previous studies [55], Carphodactylidae is the weakly supported sister group to Pygopodidae, and this clade is the sister group of Diplodactylidae (Figures 1, 2). We recover clades within the former Gekkonidae that correspond to the strongly supported families Eublepharidae, Sphaerodactylidae, Phyllodactylidae, and Gekkonidae as in previous studies, and similar relationships among these groups [55-57,59,60,98-100].

Within Gekkota, we find evidence for non-monophyly of many genera. Many relationships among the New Caledonian diplodactylids are weakly supported (Figure 2), and there is apparent non-monophyly of the genera *Rhacodactylus, Bavayia,* and *Eurydactylodes* with respect to each other and *Oedodera, Dierogekko, Paniegekko, Correlophus*, and *Mniarogekko*[101]. In the Australian diplodactylids, *Strophurus taenicauda* is strongly supported as belonging to a clade that is only distantly related to the other sampled *Strophurus* species (Figure 2). The two species of the North African sphaerodactylid genus *Saurodactylus* are divided between the two major sphaerodactylid clades (Figure 3), but the associated branches are weakly supported. The South American phyllodactylid genus *Homonota* is strongly supported as being paraphyletic with respect to *Phyllodactylus* (Figure 3).

A number of gekkonid genera (Figure 4) also appear to be non-monophyletic, including the Asian genera *Cnemaspis* (sampled species divided into two non-sister clades), *Lepidodactylus* (with respect to *Pseudogekko* and some *Luperosaurus*), *Gekko* (with respect to *Ptychozoon* and *Lu. iskandari*), *Luperosaurus* (with respect to *Lepidodactylus* and *Gekko*), *Mediodactylus* (with respect to *Pseudoceramodactylus*, *Tropiocolotes*, *Stenodactylus, Cyrtopodion, Bunopus, Crossobamon,* and *Agamura*), and *Bunopus* (with respect to *Crossobamon*), and the African *Afrogecko* (with respect to *Afroedura, Christinus, Cryptactites,* and *Matoatoa*)*, Afroedura* (with respect to *Afrogecko, Blaesodactylus, Christinus, Geckolepis*, *Pachydactylus, Rhoptropus,* and numerous other genera), *Chondrodactylus* (with respect to *Pachydactylus laevigatus*), and *Pachydactylus* (with respect to *Chondrodactylus* and *Colopus*). Many of these taxonomic problems in gekkotan families have been identified in previous studies (e.g. [59,99,102]), and extensive changes will likely be required to fix them.

### Scincoidea

We strongly support (SHL = 100; Figures 1, 5, 6, 7, 8, 9, 10) the monophyly of Scincoidea (Scincidae, Xantusiidae, Gerrhosauridae, and Cordylidae), as in other recent studies [16-20]. All four families are strongly supported (Figures 5, 6, 7, 8, 9, 10). A similar clade is also recognized in morphological phylogenies [14], though without Xantusiidae in some [13].

Within the New World family Xantusiidae, we corroborate previous analyses [103,104] that found strong support for a sister-group relationship between *Xantusia* and *Lepidophyma*, excluding *Cricosaura* (Figure 5). These relationships support the subfamily Cricosaurinae for *Cricosaura*[105]. We also recognize Xantusiinae for the North American genus *Xantusia* and Lepidophyminae for the Central American genus *Lepidophyma*[106,107].

Within the African and Madagascan family Gerrhosauridae (Figure 5), the genus *Gerrhosaurus* is weakly supported as being paraphyletic with respect to the clade comprising *Tetradactylus + Cordylosaurus,* with *G. major* placed as the sister group to all other gerrhosaurids. Within Cordylidae (Figure 5), we use the generic taxonomy from a recent phylogenetic analysis and re-classification based on multiple nuclear and mitochondrial genes [108]. This classification broke up the non-monophyletic *Cordylus*[109] into several smaller genera, and we corroborate the non-monophyly of the former *Cordylus* and support the monophyly of the newly recognized genera (Figure 5). We support the distinctiveness of *Platysaurus* (Figure 5) and recognition of the subfamily Platysaurinae [108].

We strong support (SHL = 100) for the monophyly of Scincidae (Figure 6) as in previous studies (e.g. [20,50,51]). We strongly support the basal placement of the monophyletic subfamily Acontiinae (Figure 6), as found in some previous studies (e.g. [20,51]) but not others (e.g. [50]). Similar to earlier studies, we find that the subfamily Scincinae (sensu [110]) is non-monophyletic, as Feylininae is nested within Scincinae (also found in [20,50,51,111]). Based on these results, synonymizing Feylininae with Scincinae produces a monophyletic Scincinae (SHL = 97), which is then sister to a monophyletic Lygosominae (SHL = 100 excluding *Ateuchosaurus*; see below) with 94% SHL support (Figures 6, 7, 8, 9, 10). This yields a new classification in which all three subfamilies (Acontiinae, Lygosominae, Scincinae) are strongly supported. Importantly, these definitions approxi\mate the traditional content of the three subfamilies [50,110], except for recognition of Feylininae.

We note that a recent revision of the New World genus *Mabuya* introduced a nontraditional family-level classification for Scincidae [112]. These authors divided Scincidae into seven families: Acontiidae, Egerniidae, Eugongylidae, Lygosomidae, Mabuyidae, Scincidae and Sphenomorphidae. However, there was no phylogenetic need for considering these clades as families, since the family Scincidae is clearly monophyletic, based on our results and others (see above). Thus, their new taxonomy changes the long-standing definition of Scincidae unnecessarily (see [113]). Furthermore, these changes were done without defining the full content (beyond a type genus) of any of these families other than Scincidae (the former Scincinae + Feylininae) and Acontiidae (the former Acontiinae).

Most importantly, the new taxonomy proposed by these authors [112] is at odds with the phylogeny estimated here, with respect to the familial and subfamilial classification of >1000 skink species (Figures 6, 7, 8, 9, 10). For instance, *Sphenomorphus stellatus* is found in a strongly supported clade containing *Lygosoma* (presumably Lygosomidae; Figure 10), which is separate from the other clade (presumably Sphenomorphidae) containing the other sampled *Sphenomorphus* species (Figure 7; note that these *Sphenomorphus* species are divided among several subclades within this latter clade). An additional problem is that *Egernia*, *Lygosoma*, and *Sphenomorphus* are the type genera of Egerniidae, Lygosomidae, and Sphenomorphidae, but are paraphyletic as currently defined (Figures 7, 8, 9, 10), leading to further uncertainty in the content and definition of these putative families.

Furthermore, Mabuyidae apparently refers to the clade (Figure 9) containing *Chioninia*, *Dasia*, *Mabuya*, *Trachylepis*, with each of these genera placed in its own subfamily (Chioniniinae, Dasiinae, Mabuyinae, and Trachylepidinae). However, several other genera are strongly placed in this group, such as *Eumecia, Eutropis*, *Lankaskincus*, and *Ristella* (Figure 9). These other genera cannot be readily fit into these subfamilial groups (i.e. they are not the sister group of any genera in those subfamilies), and *Trachylepis* is paraphyletic with respect to *Eumecia, Chioninia,* and *Mabuya* (Figure 9). Also, we find that *Emoia* is divided between clades containing *Lygosoma* (Lygosomidae) and *Eugongylus* (Eugongylidae), and many of these relationships have strong support (Figure 10). Finally, *Ateuchosaurus* is apparently not accounted for in their classification, and here is weakly placed as the sister-group to a clade comprising their Sphenomorphidae, Egerniidae, Mabuyidae, and Lygosomidae (i.e. Lygosominae as recognized here; Figures 7, 8, 9, 10).

These authors [112] argued that a more heavily subdivided classification for skinks may be desirable for facilitating future taxonomic revisions and species descriptions. However, this classification seems likely to only exacerbate existing taxonomic problems (e.g. placing congeneric species in different families without revising the genus-level taxonomy). Here, we retain the previous definition of *Mabuya* (restricted to the New World clade; sensu [114]), and we support the traditional definitions of Scincidae, Acontiinae, Scincinae (but including Feylininae), and Lygosominae (Figures 6, 7, 8, 9, 10; note that we leave *Ateuchosaurus* as *incertae sedis*). The other taxonomic issues in Scincidae identified here and elsewhere should be resolved in future studies. Our phylogeny provides a framework in which these analyses can take place (i.e. identifying major subclades within skinks), which we think may be more useful than a classification lacking clear taxon definitions.

Of the 133 scincid genera [1], we can assign the 113 sampled in our tree to one of the three subfamilies in our classification (Acontiinae, Lygosominae, and Scincinae; Appendix I). We place 19 of the remaining genera into one of the three subfamilies based on previous classifications (e.g. [110]), with *Ateuchosaurus* as *incertae sedis* in Scincidae. Below, we review the non-monophyletic genera in our tree. Many of these problems have been reported by previous authors [51,111,115-118], and for brevity we do not distinguish between cases reported in previous studies, and potentially new instances found here.

Within Acontiinae, we find that the two genera are both monophyletic (Figure 6). Within Scincinae, many genera are now strongly monophyletic (thanks in part to the dismantling of *Eumeces*; [49,50,119]), but some problems remain (Figure 6). The genera *Scincus* and *Scincopus* are strongly supported as being nested inside of the remaining *Eumeces*. Among Malagasy scincines (see [49,120]), *Pseudacontias* is nested inside *Madascincus*, and the genera *Androngo*, *Pygomeles*, and *Voeltzkowia* are all nested in *Amphiglossus* (Figure 6).

We also find numerous taxonomic problems within lygosomines (Figures 7, 8, 9, 10). Species of *Sphenomorphus* are widely dispersed among other lygosomine genera. The genus *Tropidophorus* is paraphyletic with respect to a clade containing many other genera (Figure 7). The sampled species of *Asymblepharus* are only distantly related to each other, including one species (*A. sikimmensis*) nested inside of *Scincella* (Figure 7). The genus *Lipinia* is polyphyletic, with one species (*L. vittigera*) strongly placed as the sister taxon to *Isopachys*, and with two other species (*L. pulchella* and *L. noctua*) placed in a well-supported clade that also includes *Papuascincus* (Figure 7).

Among Australian skinks, the genus *Eulamprus* is polyphyletic with respect to *Nangura*, *Calyptotis*, *Gnypetoscincus*, *Coggeria*, *Coeranoscincus*, *Ophioscincus*, *Saiphos*, *Anomalopus*, *Eremiascincus*, *Hemiergis*, *Glaphyromorphus*, *Notoscincus*, *Ctenotus*, and *Lerista*, and most of the relevant nodes are strongly supported (Figure 8). The genera *Coeranoscincus* and *Ophioscincus* are polyphyletic with respect to each other and to *Saiphos* and *Coggeria* (Figure 8). The genus *Glaphyromorphus* is paraphyletic with respect to a clade of *Eulamprus* (Figure 8). The genus *Egernia* is paraphyletic with respect to *Bellatorias* (which is paraphyletic with respect to *Egernia* and *Lissolepis*) and *Lissolepis*, although many of the relevant nodes are not strongly supported (Figure 9). The genera *Cyclodomorphus* and *Tiliqua* are paraphyletic with respect to each other (Figure 9).

Among other lygosomines, *Trachylepis* is non-monophyletic [121], with two species (*T. aurata* and *T. vittata*) that fall outside the strongly supported clade containing the other species (Figure 9). The latter clade is weakly supported as the sister group to a clade containing *Chioninia*, *Eumecia*, and *Mabuya*. In *Mabuya*, a few species (*M. altamazonica*, *M. bistriata*, and *M. nigropuncata*) have unorthodox placements within a monophyletic *Mabuya*, potentially due to uncertain taxonomic assignment of specimens by previous authors [51,122]. The genus *Lygosoma* is paraphyletic with respect to *Lepidothyris* and *Mochlus*, and many of the relevant nodes are strongly supported (Figure 10). Among New Caledonian skinks, the genus *Lioscincus* is polyphyletic with respect to *Marmorosphax*, *Celatiscincus,* and *Tropidoscincus*, and both *Lioscincus* and *Tropidoscincus* are paraphyletic with respect to, *Kanakysaurus, Lacertoides, Phoboscincus, Sigaloseps, Tropidoscincus, Graciliscincus, Simiscincus,* and *Caledoniscincus*, with strong support for most relevant nodes (Figure 10). The genera *Emoia* and *Bassiana* are massively polyphyletic and divided across multiple lygosomine clades (Figure 10). The genus *Lygisaurus* appears to be nested inside of *Carlia*, although many of the relevant branches are only weakly supported (Figure 10).

### Lacertoidea

Within Lacertoidea (Figure 1), we corroborate recent molecular analyses (e.g. [16,17,19,20]) and morphology-based phylogenies and classifications (e.g. [13,15]) in supporting the clade including the New World families Gymnophthalmidae and Teiidae (Figure 11). Within a weakly supported Teiidae (Figure 11), the subfamilies Tupinambinae and Teiinae are each strongly supported as monophyletic, as in previous studies [61]. In Tupinambinae, *Callopistes* is the sister group to a clade containing *Tupinambis*, *Dracaena*, and *Crocodilurus*. The clade *Dracaena + Crocodilurus* is nested within *Tupinambis*, and the associated clades have strong support (Figure 11). We find that the teiine genera *Ameiva* and *Cnemidophorus* are non-monophyletic (Figure 11), interdigitating with each other and the monophyletic genera *Aspidoscelis*, *Dicrodon* (monotypic), and *Kentropyx*, as in previous phylogenies [62].

A recent study [123] proposed a re-classification of the family Teiidae based on analysis of 137 morphological characters for 101 terminal species (with ~150 species in the family [1]). Those authors erected several new genera and subfamilies in an attempt to deal with the apparent non-monophyly of currently recognized taxa in their tree. However, in our tree, some of these new taxa conflict strongly with the phylogeny or are rendered unnecessary. First, they recognize Callopistinae as a distinct subfamily for *Callopistes*, arguing that failure to do so would produce a taxonomy inconsistent with teiid phylogeny. However, we find strong support for *Callopistes* in its traditional placement as part of Tupinambinae (Figure 11), and this change is thus not needed based on our results. The genus *Ameiva* is paraphyletic under traditional definitions [62]. In our tree, their conception of *Ameiva* is also non-monophyletic, with species found in three distinct clades (Figure 11). We also find non-monophyly of many of their species groups within *Ameiva*, including the *ameiva, bifrontata, dorsalis,* and *erythrocephala* groups (Figure 11). Their genera *Aurivela* (*Cnemidophorus longicaudus*) and *Contomastyx* (*Cnemidophorus lacertoides*) are strongly supported as sister taxa in our tree, and are nested within *Ameiva* in their *erythrocephala* species group, along with *Dicrodon* (Figure 11).

On the positive side, many of the genera they recognize are monophyletic and are not nested in other genera in our tree, including their *Ameivula* (*Cnemidophorus ocellifer*)*, Aspidoscelis* (unchanged from previous definitions), *Cnemidophorus* (excluding *C. ocellifer, C. lacertoides,* and *C. longicaudus*)*, Holcosus* (*Ameiva undulata*, *A. festiva*, and *A. quadrilineatus*)*, Kentropyx* (unchanged from previous definitions), *Salvator* (*Tupinambis rufescens, T. duseni*, and *T. merianae*), and *Teius* (unchanged from previous definitions). We did not sample *Ameiva edracantha* (their *Medopheos*).

Given our results, major taxonomic rearrangements within Teiidae seem problematic at present, especially with the extensive paraphyly of many traditional and re-defined teiid genera, the lack of strong resolution of many of these relationships based on molecular and morphological data, and incomplete taxon sampling in all studies so far. Thus, we provisionally retain the traditional taxonomy of Teiidae, pending additional data and analyses. However, we note that *Ameiva, Cnemidophorus*, and *Tupinambis* are clearly non-monophyletic based on both our results and those of recent authors [123], and will require taxonomic changes in the future. We anticipate that many of these newly proposed genera [123] will be useful in such revisions.

We find strong support (SHL = 98) for monophyly of Gymnophthalmidae (Figure 11). Within Gymnophthalmidae, we find strong support for the monophyly of the previously recognized subfamilies [63,64,124], with the exception of Cercosaurinae (Figure 11). Previous researchers considered the genus *Bachia* a distinct tribe (Bachiini) within Cercosaurinae, based on a poorly supported sister-group relationship with the tribe Cercosaurini [63,64]. Here, we find a moderately well supported relationship (SHL = 84) between *Bachia* and Gymnophthalminae + Rhachisaurinae, and we find that this clade is only distantly related to other Cercosaurinae. Therefore, we restrict Cercosaurinae to the tribe Cercosaurini, and elevate the tribe Bachiini [64] to the subfamily level. The subfamily Bachiinae contains only the genus *Bachia* (Figure 11), identical in content to the previously recognized tribe [64]. Within Cercosaurinae, we find that the genus *Petracola* is nested within *Proctoporus* (Figure 11). In Ecpleopinae, *Leposoma* is divided into two clades, separated by *Anotosaura*, *Colobosauroides*, and *Arthrosaura* (Figure 11), and many of the relevant nodes are very strongly supported. These issues should be addressed in future studies.

Our results show strong support for a clade uniting Lacertidae and Amphisbaenia (Figure 1), as in many previous studies [16-20,23]. We also find strong support for monophyly of amphisbaenians (SHL = 99), in contrast to some molecular analyses [19,20]. Relationships among amphisbaenian families are generally strongly supported and similar to those in earlier molecular studies (Figure 12), including the placement of the New World family Rhineuridae as sister group to all other amphisbaenians [70,71,125]. The family Cadeidae is placed as the sister-group to Amphisbaenidae + Trogonophiidae (Figures 1 and Figure 12) with weak support, but has been placed with Blanidae in previous studies, with strong support but less- extensive taxon sampling [125,126].

We find strong support for monophyly of the Old World family Lacertidae (Figure 13). Within Lacertidae, branch support for the monophyly of most genera and for the subfamilies Gallotiinae and Lacertinae is very high (Figure 13). However, we find that relationships among many genera are poorly supported, as in previous studies [65,67,68]. Our results (Figure 13) also indicate that several lacertid genera are non-monophyletic with strong support for the associated nodes, including *Algyroides* (paraphyletic with respect to *Dinarolacerta*), *Ichnotropis* (paraphyletic with respect to *Meroles*), *Meroles* (paraphyletic with respect to *Ichnotropis*), *Nucras* (polyphyletic with respect to several genera, including *Pedioplanis*, *Poromera*, *Latastia*, *Philocortus*, *Pseuderemias*, and *Heliobolus*), and *Pedioplanis* (paraphyletic with respect to *Nucras*).

### Higher-level phylogeny of Toxicofera

We find strong support (SHL = 96) for monophyly of Toxicofera (Anguimorpha, Iguania, and Serpentes; Figure 1), and moderate support for a sister-group relationship between Iguania and Anguimorpha (SHL = 79). Relationships among Anguimorpha, Iguania, and Serpentes were weakly supported in some Bayesian and likelihood analyses [16-19], but strongly supported in others [20]. We further corroborate previous studies in also placing Anguimorpha with Iguania [16-20]. In contrast, some other studies have placed anguimorphs with snakes as the sister group to iguanians [127,128].

### Anguimorpha

Our hypothesis for family-level anguimorphan relationships (Figures 1, 14) is generally similar to that of other recent studies [17,19,20,129], and is strongly supported. Our results differ from some analyses based only on morphology, which place Anguidae near the base of Anguimorpha [130]. Here, Shinisauridae is strongly supported as the sister taxon to a well-supported clade of Varanidae + Lanthanotidae (Figures 1, 14). Varanid relationships are similar to previous estimates (e.g. [131]). Xenosauridae is here strongly supported as the sister-group to a strongly supported clade containing Helodermatidae and the strongly supported Anniellidae + Anguidae clade (Figures 1, 14). However, previous molecular analyses have placed Helodermatidae as the sister to Xenosauridae + (Anniellidae + Anguidae), typically with strong support [16,17,19,20].

Within Anguidae (Figure 14), our phylogeny indicates non-monophyly of genera within every subfamily, including Diploglossinae (*Diploglossus* and *Celestus* are strongly supported as paraphyletic with respect to each other and to *Ophiodes*), Anguinae (*Ophisaurus* is strongly supported as paraphyletic with respect to *Anguis*, *Dopasia*, and *Pseudopus*), and Gerrhonotinae (*Abronia* and *Mesaspis* are non-monophyletic, and *Coloptychon* is nested inside *Gerrhonotus*). Some of these problems were not reported previously (e.g. *Coloptychon, Abronia*, and *Mesaspis*), due to incomplete taxon sampling in previous studies [129,132,133], but relationships within Gerrhonotinae are under detailed investigation by other researchers, so these issues are likely to be resolved in the near future.

### Iguania

We find strong support (SHL = 100) for the monophyly of Iguania (Figure 1). This clade is strongly supported by nuclear data [16,17,19,20], but an apparent episode of convergent molecular evolution in several mitochondrial genes has seemingly misled some analyses of mtDNA, leading to weak support for Iguania [134], or even separation of the acrodonts and pleurodonts [17,135] in previous studies. Within Iguania (Figure 1), we find strong support (SHL = 100) for a sister-group relationship between Chamaeleonidae and Agamidae (Acrodonta), and for a clade of mostly New World families (Pleurodonta; SHL = 100).

We find strong support for the monophyly of Chamaeleonidae and the subfamily Chamaeleoninae, and weak support for the paraphyly of Brookesiinae (Figures 1, 15). The sampled species of the *Brookesia nasus* group appear as the sister group to all other chamaeleonids (the latter clade weakly supported) as found by some previous authors [136], though other studies have recovered a monophyletic *Brookesia*[137,138]. Within Chamaeleoninae (Figure 15), we find strong support for the monophyly of most genera and species-level relationships. However, we find strong support for the non-monophyly of *Calumma*, with some species strongly placed with *Chamaeleo*, others strongly placed with *Rieppeleon*, and a third set weakly placed with *Nadzikambia* + *Rhampoleon.* While non-monophly of *Calumma* has also been found in previous studies [138], a recent study strongly supports monophyly of *Brookesia* and weakly supports monophyly of *Calumma*[139].

Monophyly of Agamidae is strongly supported (Figure 16; SHL = 100), contrary to some previous estimates [15,31]. Most relationships among agamid subfamilies and genera are strongly supported (Figure 16), and largely congruent with earlier studies [17,29,34,140]. There are some differences with earlier studies. For example, previous studies based on 29–44 loci [20,29,34] placed Hydrosaurinae as sister to Amphibolurinae + (Agaminae + Draconinae) with strong support, whereas we place Hydrosaurinae as the sister-group to Amphibolurinae with weak support. Other authors [140] placed Leiolepiedinae with Uromastycinae, but we (and most other studies) place Uromastycinae as the sister group to all other agamids.

Our phylogeny indicates several taxonomic problems within amphibolurine agamids (Figure 16). The genera *Moloch* and *Chelosania* render *Hypsilurus* paraphyletic, although the support for the relevant clades is weak. The species *Lophognathus gilberti* is placed in a strongly supported clade with *Chlamydosaurus* and *Amphibolurus*, a clade that is not closely related to the other *Lophognathus*. Many of these taxonomic problems were also noted by previous authors [141].

Within agamine agamids (Figure 16), most relationships are well supported and monophyly of all sampled genera is strongly supported. In contrast, within draconine agamids (Figure 16), many intergeneric relationships are weakly supported, and some genera are non-monophyletic (Figure 16; see also [142]), including *Gonocephalus* (*G. robinsonii* is only distantly related to other *Gonocephalus*) and *Japalura* (with species distributed among three distantly related clades, including one allied with *Ptyctolaemus*, another with *G. robinsonii,* and a third with *Pseudocalotes*).

Recent authors suggested dividing *Laudakia* into three genera (*Stellagama, Paralaudakia*, and *Laudakia*) based on a non-phylogenetic analysis of morphology [143]. Here, *Laudakia* (as previously defined) is strongly supported as monophyletic (Figure 16), and this change is not necessitated by the phylogeny. Similarly, based on genetic and morphological data, recent authors [144] suggested resurrecting the genus *Saara* for the basal clade of *Uromastyx* (*U. asmussi, U. hardwickii,* and *U. loricata*). However, *Uromastyx* (as previously defined) is strongly supported as monophyletic in our results (Figure 16) and in those of the recent revision [144], and this change is not needed. We therefore retain *Laudakia* and *Uromastyx* as previously defined, to preserve taxonomic stability in these groups [113]. We note that recent studies have also begun to revise species limits in other groups such as *Trapelus*[145], and taxa such as *T. pallidus* (Figure 16) may represent populations within other species.

Within Pleurodonta we generally confirm the monophyly and composition of the clades that were ranked as families (or subfamilies) within the group (e.g. Phrynosomatidae, Opluridae, Leiosauridae, Leiocephalidae, and Corytophanidae; Figures 1, 17, 18, 19) based on previous molecular studies [31,33,34] and earlier morphological analyses [146,147].

One important exception is the previously recognized Polychrotidae. Our results confirm that *Anolis* and *Polychrus* are not sister taxa (Figures 1, 18, 19), as also found in some previous molecular studies [31,33,34], but not others [20,29]. Our results provide strong support for non-monophyly of Polychrotidae, placing *Polychrus* with Hoplocercidae (SHL = 99) and *Anolis* with Corytophanidae (SHL = 99; the latter also found by [34]). Recent analyses placing *Anolis* with *Polychrus* showed only weak support for this relationship [20,29], despite many loci (30–44). We support continued recognition of Dactyloidae for *Anolis* and Polychrotidae for *Polychrus*[34], based on a limited number of loci but extensive taxon sampling. We note that these families are still monophlyetic, even if they prove to be sister taxa.

Interestingly, our results for relationships among pleurodont families differ from most previous studies, and are surprisingly well-supported in some cases by SHL values (but see below). In previous studies, many relationships among pleurodont families were poorly supported by Bayesian posterior probabilities and by parsimony and likelihood bootstrap values, though typically sampling fewer taxa or characters [17,31,33,148-151]. Studies including 29 nuclear loci found strong concatenated Bayesian support for many relationships but weak support from ML bootstrap analyses for many of the same relationships [34]. The latter pattern (typically weak ML support) was also found in an analysis including those same 29 loci and mitochondrial data for >150 species [29]. We also find a mixture of strongly and weakly supported clades, but with many relationships that are incongruent with these previous studies. First, we find that Tropiduridae is weakly supported as the sister group to all other pleurodonts (also found by [29]), followed successively (Figures 1, 17, 18, 19) by Iguanidae, Leiocephalidae, Crotaphytidae + Phrynosomatidae, Polychrotidae + Hoplocercidae, and Corytophanidae + Dactyloidae.

The relatively strong support for the clades Crotaphytidae + Phrynosomatidae (SHL = 87), Polychrotidae + Hoplocercidae (SHL = 99), and Corytophanidae + Dactyloidae (SHL = 99) is largely unprecedented in previous studies (although Corytophanidae + Dactyloidae is strongly supported in some Bayesian analyses [34]). As in many previous analyses, deeper relationships among the families remain weakly supported. We also find a strongly supported clade containing Liolaemidae, Opluridae, and Leiosauridae (SHL = 95), with Opluridae + Leiosauridae also strongly supported (SHL = 99). Both clades have also been found in previous studies [149,151], including studies based on 29 or more nuclear loci [20,29,34].

We note that previous studies have shown strong support for some pleurodont relationships (e.g. basal placement of phrynosomatids; see [34]), only to be strongly overturned with additional data [20,29]. Therefore, the relationships found here should be taken with some caution (even if strongly supported), with the possible exception of the recurring clade of Liolaemidae + (Opluridae + Leiosauridae).

All pleurodont families are strongly supported as monophyletic (SHL > 85). Within the pleurodont families, our results generally support the current generic-level taxonomy (Figures 17, 18, 19). However, there are some exceptions. Within Tropiduridae (Figure 17), *Tropidurus* is paraphyletic with respect to *Eurolophosaurus*, *Strobilurus*, *Uracentron*, and *Plica*. Within Opluridae (Figure 18), the monotypic genus *Chalarodon* renders *Oplurus* paraphyletic. Two leiosaurid genera are also problematic (Figure 18). In Enyaliinae, *Anisolepis* is paraphyletic with respect to *Urostrophus*, and this clade is nested within *Enyalius*. In Leiosaurinae, *Pristidactylus* is rendered paraphyletic by *Leiosaurus* and *Diplolaemus* (Figure 18).

Within Dactyloidae, a recent study re-introduced a more subdivided classification of anoles [152], an issue that has been debated extensively in the past [153-156]. Our results support the monophyly of all the genera recognized by recent authors [152], including *Anolis, Audantia*, *Chamaelinorops, Dactyloa, Deiroptyx, Norops,* and *Xiphosurus* (see Figure 19). However, since *Anolis* is monophyletic as traditionally defined, we retain that definition here (including the seven listed genera) for continuity with the recent literature [113,157].

### Serpentes

Relationships among the major serpent groups (Figure 1) are generally similar to other recent studies [20,35,36,38,41,44,47,158-160]. We find that the blindsnakes, Scolecophidia (Figures 1, 20) are not monophyletic, as in previous studies [19,20,36,44,159,160]. Similar to some previous studies [44,159], our data weakly place Anomalepididae as the sister taxon to all snakes, and the scolecophidian families Gerrhopilidae, Leptotyphlopidae, Typhlopidae, and Xenotyphlopidae as the sister-group to all other snakes excluding Anomalepididae (Figures 1, 20). Previous studies have also placed Anomalepididae as the sister-group to all non-scolecophidian snakes [19,20,35,36,158,160], in some cases with strong support [20]. Although it might appear that recent analyses of scolecophidian relationships [38] support monophyly of Scolecophidia (e.g. Figure 1 of [38]), the tree including non-snake outgroups from that study shows weak support for placing anomalepidids with alethinophidians, as in other studies [19,20,36,160].

We follow recent authors [38] in recognizing Xenotyphlopidae (strongly placed as the sister taxon of Typhlopidae) and Gerrhopilidae (strongly placed as the sister group of Xenotyphlopidae + Typhlopidae) as distinct families (Figure 20). Leptotyphlopidae is strongly supported as the sister group of a clade comprising Gerrhopilidae, Xenotyphlopidae, and Typhlopidae (Figure 20). As in previous studies [38,44], we find strong support for non-monophyly of several typhlopid genera (*Afrotyphlops, Austrotyphlops, Ramphotyphlops*, *Letheobia*, and *Typhlops*; Figure 20). There are also undescribed taxa (e.g. Typhlopidae sp. from Sri Lanka; [44]) of uncertain placement within this group (Figure 20). The systematics of typhlopoid snakes will thus require extensive revision in the future, with additional taxon and character sampling.

Within Alethinophidia (SHL = 100), Aniliidae is strongly supported (SHL = 98) as the sister taxon of Tropidophiidae (together comprising Anilioidea), and all other alethinophidians form a strongly supported sister group to this clade (SHL = 97; Figures 1, 21). The enigmatic family Xenophidiidae is weakly placed as the sister-group to all alethinophidians exclusive of Anilioidea (Figures 1, 21). The family Bolyeriidae is weakly placed as the sister-group to pythons, boas, and relatives (Booidea), which are strongly supported (SHL = 88). Relationships in this group are generally consistent with other recent molecular studies [20,35-37,47,159].

Relationships among other alethinophidians are a mixture of strongly and weakly supported nodes (Figures 1, 21). We find strong support (SHL = 100) for a clade containing Anomochilidae + Cylindrophiidae + Uropeltidae. This clade of three families is strongly supported (SHL = 89) as the sister taxon to Xenopeltidae + (Loxocemidae + Pythonidae). Together, these six families form a strongly supported clade (SHL = 89; Figures 1, 21) that is weakly supported as the sister group to the strongly supported clade of Boidae + Calabariidae. Within the clade of Anomochilidae, Cylindrophiidae, and Uropeltidae (Figure 21), we weakly place *Anomochilus* as the sister group to Cylindrophiidae [44], in contrast to previous studies which placed *Anomochilus* within *Cylindrophis*[161]. However, support for monophyly of *Cylindrophis* excluding *Anomochilus* is weak (Figure 21). As in previous studies [44,162], we find several taxonomic problems within Uropeltidae (Figure 21). Specifically, *Rhinophis* and *Uropeltis* are paraphyletic with respect to each other and to *Pseudotyphlops*. The problematic taxa are primarily Sri Lankan [44], and forthcoming analyses will address these issues.

Within Pythonidae (Figure 21), the genus *Python* is the sister group to all other genera. Some species that were traditionally referred to as *Python* (*P. reticulatus* and *P. timoriensis*) are instead sister to an Australasian clade consisting of *Antaresia*, *Apodora*, *Aspidites*, *Bothrochilus*, *Leiopython*, *Liasis*, and *Morelia* (Figure 21). These taxa (*P. reticulatus* and *P. timoriensis*) have been referred to as *Broghammerus*, a name originating from an act of "taxonomic vandalism" (i.e. an apparently intentional attempt to disrupt stable taxonomy) in a non-peer reviewed organ without data or analyses [163,164]. However, this name was, perhaps inadvertently, subsequently used by researchers in peer-reviewed work [165] and has entered into somewhat widespread usage [1]. This name should be ignored and replaced with a suitable substitute. Within the Australasian clade (Figure 21), *Morelia* is paraphyletic with respect to all other genera, and *Liasis* is non-monophyletic with respect to *Apodora*, although many of the relevant relationships are weakly supported.

Within Boidae (Figure 21), our results and those of other recent studies [20,36,47,48,150,166] have converged on estimated relationships that are generally similar to each other but which differ from traditional taxonomy [167]. However, the classification has yet to be modified to reflect this, and we rectify this situation here. We find that Calabariidae is nested within Boidae [150], but this is poorly supported, and contrary to most previous studies [47,48]. While *Calabaria* has been classified as an erycine boid in the past, this placement is strongly rejected here and in other studies [47,48]. If the current placement of *Calabaria* is supported in the future, it would require recognition as the subfamily Calabariinae in Boidae.

The Malagasy boine genera *Acrantophis* and *Sanzinia* are placed as the sister taxa to a weakly-supported clade containing Calabariidae and a strongly supported clade (SHL = 99) comprising the currently recognized subfamilies Erycinae, Ungaliophiinae, and other boines (Figure 21). Regardless of the position of Calabariidae, this placement of Malagasy boines renders Boinae paraphyletic. We therefore resurrect the subfamily Sanziniinae [168] for *Acrantophis* and *Sanzinia*. This subfamily could be recognized as a distinct family if future studies also support placement of this clade as distinct from other Boidae + Calabariidae.

The genera *Lichanura* and *Charina* are currently classified as erycines [1], but are strongly supported as the sister group to Ungaliophiinae, as in previous studies [20,36,47,166]. We expand Ungaliophiinae to include these two genera (Figure 21), rather than erect a new subfamily for these taxa. The subfamily Ungaliophiinae is placed as the sister group to a well-supported clade (SHL = 87) containing the rest of the traditionally recognized Erycinae and Boinae. We restrict Erycinae to the Old World genus *Eryx*.

The genus *Candoia* (Boinae) from Oceania and New Guinea [1], is placed as the sister taxon to a moderately supported clade (SHL = 83) consisting of Erycinae (*Eryx*) and the remaining genera of Boinae (*Boa*, *Corallus*, *Epicrates*, and *Eunectes*). To solve the non-monophyly of Boinae with respect to Erycinae (due to *Candoia*), we place *Candoia* in a new subfamily (Candoiinae, *subfam. nov*.; see Appendix I). Boinae then comprises the four Neotropical genera that have traditionally been classified in this group (*Boa*, *Corallus*, *Epicrates*, and *Eunectes*). We acknowledge that non-monophyly of Boinae could be resolved in other ways (e.g. expanding it to include Erycinae). However, our taxonomy maintains the traditionally used subfamilies Boinae, Erycinae, and Ungaliophiinae, modifies them to reflect the phylogeny, and recognizes the phylogenetically distinct boine clades as separate subfamilies (Candoiinae, Sanziniinae). Within Boinae, *Eunectes* renders *Epicrates* paraphyletic, but this is not strongly supported (see also [48]).

Our results for advanced snakes (Caenophidia) are generally similar to those of other recent studies [41,42,169], and will only be briefly described. However, in contrast to most recent studies [20,36,41,42,81,159,160], Acrochordidae is here strongly placed (SHL = 95) as the sister group to Xenodermatidae. This clade is then the sister group to the remaining Colubroidea, which form a strongly supported clade (SHL = 100; Figures 1, 22). This relationship has been found in some previous studies [169,170], and was hypothesized by early authors [171]. Further evidence will be required to resolve this conclusively. Analyses based on concatenation of 20–44 loci do not support this grouping [20,36], though preliminary species-tree analyses of >400 loci do (Pyron et al., in prep.). Relationships in Pareatidae are similar to recent studies [172], and the group is strongly placed as the sister taxon to colubroids excluding xenodermatids (SHL = 100; Figures 1 , 22), as in most recent analyses (e.g. [41,43,44]).

The family Viperidae is the sister group to all colubroids excluding xenodermatids and pareatids (Figure 1), as in other recent studies. The family Viperidae is strongly supported (Figure 22), as is the subfamily Viperinae, and the sister-group relationship between Azemiopinae and Crotalinae (SHL = 100). Our results generally support the existing generic-level taxonomy within Viperinae (Figure 22). However, we recover a strongly supported clade within Viperinae consisting of *Daboia russelii*, *D*. *palaestinae*, *Macrovipera mauritanica*, and *M. deserti* (Figure 22), as in previous studies [173]. We corroborate previous suggestions that these taxa be included in *Daboia*[174], though this has not been widely adopted [1]. The other *Macrovipera* species (including the type species) remain in that genus (Figure 22).

Within Crotalinae (Figure 22), a number of genera appear to be non-monophyletic. The species *Trimeresurus gracilis* is strongly supported as the sister taxon to *Ovophis okinavensis* and distantly related to other *Trimeresurus*, whereas the other *Ovophis* are strongly placed as the sister group to *Protobothrops*. A well-supported clade (SHL = 90) containing *Atropoides picadoi*, *Cerrophidion*, and *Porthidium* renders *Atropoides* paraphyletic (see also [175]). The species *Bothrops pictus*, considered *incertae sedis* in previous studies [176], is here strongly supported as the sister taxon to a clade containing *Rhinocerophis*, *Bothropoides*, *Bothriopsis*, and *Bothrops* (Figure 22). Most of these relationships are strongly supported.

Viperidae is strongly placed (SHL = 95) as the sister taxon to a well-supported clade (SHL = 100) containing Colubridae, Elapidae, Homalopsidae, and Lamprophiidae (Figure 1). Monophyly of Homalopsidae is also strongly supported (Figure 23). Within Homalopsidae, non-monophyly of the genus *Enhydris* is strongly supported (Figure 23), and it should likely be split into multiple genera. Homalopsidae is weakly supported (SHL = 58) as the sister group of Elapidae + Lamprophiidae (Figure 1). This same relationship was also weakly supported by previous analyses [41,44], but other studies have found strong support for placing Homalopsidae as the sister group of a strongly supported clade including Elapidae, Lamprophiidae, and Colubridae [20,36], including data from >400 loci (Pyron et al., in prep.).

Support for the monophyly of Lamprophiidae is strong (but excluding *Micrelaps*; see below), and most of its subfamilies are well-supported [40,41,177,178] including Atractaspidinae, Aparallactinae, Lamprophiinae, Prosymninae (weakly placed as the sister-group to *Oxyrhabdium*), Pseudaspidinae, Psammophiinae, and Pseudoxyrhophiinae (Figure 23). In Lamprophiidae, most genera are monophyletic based on our sampling (Figure 23). However, within Aparallactinae, *Xenocalamus* is strongly placed within *Amblyodipsas*, and in Atractaspidinae, *Homoroselaps* is weakly placed in *Atractaspis*. In Lamprophiinae, *Lamprophis* is paraphyletic with respect to *Lycodonomorphus* but support for the relevant clades is weak.

The enigmatic genera *Buhoma* from Africa and *Psammodynastes* from Asia were both previously considered *incertae sedis* within Lamprophiidae [41]. Here they are weakly placed as sister taxa, and more importantly, they form a strongly supported clade with the African genus *Pseudaspis* (Pseudaspidinae; SHL = 95; Figure 23). Therefore, we expand Pseudaspidinae to include these two genera.

The genus *Micrelaps* (putatively an aparallactine; [1]) is weakly placed as the sister taxon to Lamprophiidae + Elapidae. Along with *Oxyrhabdium* (see above) and *Montaspis*[40], this genus is treated as *incertae sedis* in our classification (Appendix I). If future studies strongly support these relationships, they may require a new family for *Micrelaps* and possibly a new subfamily for *Oxyrhabdium*, though placement of these taxa has been highly variable in previous studies [40,41,44,81].

Monophyly of Elapidae is strongly supported (Figure 24), and *Calliophis melanurus* is strongly supported as the sister group to all other elapids (see also [44]). Within Elapidae (Figure 24), relationships are generally concordant with previous taxonomy, with some exceptions. The genera *Toxicocalamus*, *Simoselaps*, and *Echiopsis* are all divided across multiple clades, with strong support for many of the relevant branches. A recent study [46] has provided a generic re-classification of the sea snakes (*Hydrophis* group) to resolve the extensive paraphyly of genera found in previous studies (e.g. [179,180]). Our results support this classification.

Monophyly of Colubridae and most of its subfamilies (sensu [41,44]) are strongly supported (Figures 1, 25, 26, 27, 28). However, relationships among many of these subfamilies are weakly supported (Figure 1), as in most previous studies [41,43,45,181]. The subfamilies Calamariinae and Pseudoxenodontinae are strongly supported as sister taxa, and weakly placed as the sister-group to the rest of Colubridae (Figure 25). There is a weakly supported clade (Figure 1) comprising Natricinae + (Dipsadinae + *Thermophis*), but the clade uniting the New World Dipsadinae with the Asian genus *Thermophis* is strongly supported (SHL = 100; Figure 28). Here, we place *Thermophis* (recently in Pseudoxenodontinae [41]) in Dipsadinae (following [182]), making it the first and only Asian member of this otherwise exclusively New World subfamily. However, despite the strong support for its placement here, placement of this taxon has been variable in previous analyses [41,182,183], and we acknowledge that future analyses may support recognition of a distinct subfamily (Thermophiinae).

The clade of Natricinae and Dipsadinae is weakly supported as the sister group (Figures 1, 25, 26, 27, 28) to a clade containing Sibynophiinae [181] + (Colubrinae + Grayiinae). The subfamily Colubrinae is weakly supported; we find that the colubrine genera *Ahaetulla*, *Chrysopelea*, and *Dendrelaphis* form a strongly supported clade that is weakly placed as the sister group to the rest of Colubrinae, which form a strongly supported clade (Figure 25). This clade was also placed with Grayiinae or Sibynophiinae in many preliminary analyses, rendering Colubrinae paraphyletic. This group of three genera has been strongly supported in the past, and only weakly placed with Colubrinae [41,44]. It is possible that future analyses will reveal that the clade of *Ahaetulla, Chrysopelea,* and *Dendrelaphis* is placed elsewhere in Colubridae with strong support, and thus merit recognition as a distinct subfamily (Ahaetuliinae). A notable feature of this clade is the presence of gliding flight in most species of *Chrysopelea*, less-developed non-flight jumping with similar locomotor origins in *Dendrelaphis*, and homologous glide-related traits in *Ahaetulla*[184].

Numerous colubroid genera are not included in our tree and are not clearly placed in subfamilies based on previous morphological evidence. In our classification, these genera are also considered *incertae sedis* within Colubridae, including *Blythia*, *Cyclocorus*, *Elapoidis*, *Gongylosoma*, *Helophis*, *Myersophis*, *Oreocalamus*, *Poecilopholis*, *Rhabdops*, and *Tetralepis*, as in previous classifications [1].

Our phylogeny reveals numerous taxonomic problems within Colubrinae (Figures 25, 26). The genus *Boiga* is paraphyletic with respect to *Crotaphopeltis, Dipsadoboa, Telescopus, Toxicodryas,* and *Dasypeltis*, with strong support (Figure 25). The genus *Philothamnus* is paraphyletic with respect to *Hapsidophrys* (Figure 25). The genus *Coluber* is split between Old World and New World clades (Figures 25, 26). The species *Hierophis spinalis* is sister to *Eirenis* to the exclusion of the other *Hierophis* species (Figure 25). The genus *Dryocalamus* is nested within *Lycodon* (Figure 26). The species *Chironius carinatus* and *C. quadricarinatus* are weakly placed in a clade of Neotropical colubrines only distantly related to the other *Chironius* species (Figure 26). The genus *Drymobius* renders *Dendrophidion* paraphyletic (Figure 26). The monotypic genus *Rhynchophis* renders the two species of *Rhadinophis* paraphyletic (Figure 26). The genus *Coronella* is rendered paraphyletic (Figure 26) by *Oocatochus* with weak support (see also [185]). Finally, the genus *Rhinechis* is nested within *Zamenis* (Figure 26).

We find numerous non-monophyletic genera within Natricinae (Figure 27), as in previous studies [41,44,78,186]. These non-monophyletic genera include the Asian genera *Amphiesma*, *Atretium*, and *Xenocrophis*. Among New World genera, we find *Regina* to be non-monophyletic with respect to most other genera, as in previous phylogenetic studies (e.g. [41,186]). Also, as in previous studies (e.g. [41,187]), we find that *Adelophis*[188] is nested deep within *Thamnophis*[189].

Finally, within a weakly supported Dipsadinae (Figure 28), we find non-monophyly of numerous genera, as in many earlier studies (e.g. [41-43,190]). These problems of non-monophyly include *Leptodeira* (with respect to *Imantodes*), *Geophis* (with respect to *Atractus*), *Atractus* (with respect to *Geophis*), *Sibynomorphus* (with respect to *Dipsas*), *Dipsas* (with respect to *Sibynomorphus*), *Taeniophallus* (with respect to *Echinanthera*), and *Echinanthera* (with respect to *Taeniophallus*). Recent revisions have begun to tackle these problems [42,43,190], but additional taxon and character sampling will be crucial to resolve relationships and taxonomy.

## Discussion

In this study, we provide a phylogenetic estimate for 4161 species of squamates based on molecular data from up to 12 genes per species, combining much of the relevant data used in previous molecular phylogenetic analyses. This tree provides a framework for future evolutionary studies, spanning from the species level to relationships among families, utilizing a common set of branch lengths. These estimated branch lengths are critically important for most phylogenetic comparative methods. To further facilitate use of this phylogeny in comparative studies, we provide the Newick version of this tree (with estimated branch lengths) in DataDryad repository 10.5061/dryad.82h0m and Additional file 1: Data File S1. Our results also suggest that the branch lengths in this tree should not generally be compromised by missing data for some genes in some taxa.

Our results also reveal many problems in squamate classification at nearly all phylogenetic levels. We make several changes to higher-level taxonomy based on this phylogeny, including changes to the traditionally recognized subfamilies of boid snakes (i.e. resurrecting Sanziniinae for the boine genera *Acrantophis* and *Sanzinia*, erecting Candoiinae for the boine genus *Candoia*, and moving *Lichanura* and *Charina* from Erycinae to Ungaliophiinae), lamprophiid snakes (expansion of Pseudaspidinae to include the formerly *incertae sedis* genera *Buhoma* and *Psammodynastes*), colubrid snakes (expansion of Dipsadinae to include the Asian pseudoxenodontine genus *Thermophis*), and gymnophthalmid lizards (recognition of Bachiinae for the tribe Bachiini, containing *Bachia*) and scincid lizards (synonymizing Feylininae with Scincinae to yield a total of three scincid subfamilies: Acontiinae, Lygosominae, and Scincinae). In Appendix I, we list the generic content of all families and subfamilies. We also find dozens of problems at the genus level, many of which have been identified previously, and which we defer the resolution of to future studies. Our results also highlight potential problems in recent proposals to modify the classification of scincid [112] and teiid lizards [123].

In addition to synthesizing existing molecular data for squamate phylogeny, our analyses also reveal several apparently novel findings (Figures 1, 2, 3, 4, 5, 6, 7, 8, 9, 10, 11, 12, 13, 14, 15, 16, 17, 18, 19, 20, 21, 22, 23, 24, 25, 26, 27, 28). Given space constraints, we cannot detail every deviation from previous phylogenetic hypotheses (especially pre-molecular studies). Nevertheless, we focus on three sets of examples. First, we find some relatively novel, strongly-supported relationships at the family level. These include the placement of Helodermatidae (as sister to Xenosauridae, Anguidae, and Anniellidae) and the placement of Xenodermatidae as the sister taxon to Acrochordidae (rendering Colubroidea paraphyletic), in contrast to most recent analyses of anguimorphs and snakes (see above). We also find some novel, strongly supported relationships among pleurodont families, but we acknowledge that these may be overturned in future studies.

The second example is the higher-level relationships within Scincidae, the largest family of lizards [1]. No previous studies examining higher-level relationships within the group included more than ~50 species [50,51]. In this study, we sample 683 skink species (Figures 6, 7, 8, 9, 10), and our phylogeny provides a unique resolution of higher-level skink relationships. Some previous researchers [51] placed acontiines as the sister group to all other skinks, but suggested that scincines and lygosomines were paraphyletic with respect to each other (with feyliniines placed with scincines). In contrast, others [50] suggested that acontiines, feyliniines, and lygosomines were all nested inside scincines (but with each of those three subfamilies as monophyletic), although many clades were only weakly supported. Here (Figures 1, 6, 7, 8, 9, 10), we find that acontiines are the sister group to a strongly supported clade consisting of a monophyletic Scincinae and a monophyletic Lygosominae (excepting the weakly supported placement of *Ateuchosaurus* and placement of Feyliniinae in Scincinae).

Third, our phylogeny reveals numerous genera that appear to be non-monophyletic, with many of these cases having strong support for the associated nodes. Our examples include genera in many families, including dibamids, diplodactylids, gekkonids, phyllodactylids, gerrhosaurids, scincids, teiids, gymnophthalmids, lacertids, anguids, chamaeleonids, agamids, tropidurids, oplurids, leiosaurids, typhlopids, pythonids, uropeltids, boids, viperids, lamprophiids, elapids, and colubrids (see Results). Although many problems noted here were found in previous studies, some seem to be new, such as placement of *Crocodilurus* and *Draceana* within *Tupinambis* (in Teiidae; Figure 11) and *Coloptychon* within *Gerrhonotus* (in Anguidae; Figure 14).

Our study also offers an important test of higher-level squamate relationships using a very different sampling strategy than that used in most previous analyses. Squamates have traditionally been divided into two clades based on morphology, Iguania and Scleroglossa (e.g. [13,21]). Despite considerable disagreement among morphology-based hypotheses, this basic division is supported by nearly all phylogenetic analyses based on morphological data [13-15,19,22,95,191]. In contrast, our results and those of most previous molecular analyses strongly support placement of iguanians with anguimorphs and snakes [16-20,23,24]. The causes of this conflict remain unclear, but may be related to morphological traits associated with different feeding strategies of iguanian and (traditional) scleroglossan squamates [3,17].

Additionally, analyses of morphology often place dibamids, amphisbaenians, snakes, and (in some cases) some scincids and anguids in a single clade [13-15]. Our analyses do not support such a clade (Figure 1), nor do other analyses of molecular data alone [17-20], or analyses of combined molecular and morphological data [19]. Instead, these morphological results seem to be explained by convergence associated with burrowing (e.g. [19]). Overall, molecular datasets have shown overwhelmingly strong support for placement of dibamids and gekkonids at the base of the tree, amphisbaenians with lacertoids, and iguanians with snakes and anguimorphs [17-20,23]. These results have now been corroborated with up to 22 genes (15794 bp) for 45 taxa [19], 25 genes (19020 bp) for 64 taxa [16], and 44 genes (33717 bp) for 161 taxa [20]. We now support this basic topology with 4161 species sampled for up to 12 genes each (up to 12896 bp).

Nevertheless, despite the overall strong support for most of the tree (i.e. 70% of all nodes have SHL > 85), certain clades remain poorly supported (e.g. relationships among many pleurodont iguanian families; Figures 1, 17, 18, 19). A potential criticism of the supermatrix approach used here is that this weak support may occur due to missing data. However, previous studies of 8 datasets have shown explicitly that there is typically little relationship between branch support for terminal taxa and the amount of missing data [85]. Instead, these patterns of weak support are more likely to reflect short underlying branch lengths [20,36,41], and may be difficult to resolve even with more complete taxonomic and genomic sampling. Indeed, as noted above, many of the weakly supported nodes in our phylogeny are also weakly supported in analyses with little missing data (<20%) and large numbers of genes (e.g. 44 genes as in [20]), such as the relationships of many pleurodont lizard families and colubroid snake families and subfamilies.

We acknowledge that the differences between our results and previous studies (noted above) do not necessarily mean that our results are right and those of previous studies are wrong. In some cases, we provide strong support for novel relationships when previous, conflicting studies showed only weak support (as in scincids, see above). In other cases, our results disagree with other studies for clades that were strongly supported (e.g. placement of xenodermatids). In the best-case scenario, these conflicts may be resolved because our results are correct, possibly reflecting the beneficial effects of adding taxa and the associated subdivision of long branches [25,26,28,87,192-194]. Furthermore, in many cases, we are including more genes than used in previous studies of particular clades, increasing sampling of characters and loci. This should generally reduce spurious results caused by sampling few characters and by incongruence between gene and species trees.

However, other explanations for incongruence between our results and previous studies are also possible. Adding taxa can potentially lead to incorrect results in some cases (e.g. [195]), such as when a long terminal branch is added that further subdivides a short internal branch. In other cases, conflicts with our results might reflect the impact of our sampling fewer nuclear genes and a correspondingly increased influence of mitochondrial data. Mitochondrial genes have relatively fast evolutionary rates (potentially exacerbating the impacts of long branches), and their phylogenetic resolution for a particular node may also reflect introgression or incomplete lineage sorting rather than the species phylogeny (review in [196]). Many taxa in the matrix are represented only by mitochondrial data, and highly variable mitochondrial genes might also overcome the influence of less variable nuclear genes in combined analyses (although this scenario does not seem to be common [196]). Such cases might explain some strongly supported conflicts between our results and those based on multiple nuclear loci. Another possibility is that some cases may represent failure to find the optimal tree (although we assume that these cases will likely show only weak support). We acknowledge that there are many reasons why our results may differ from previous studies, and the ultimate test of these novel findings will be corroboration in future studies that include more taxa and characters.

This analysis also corroborates several recent studies suggesting that the supermatrix approach is a powerful strategy for large-scale phylogenetic inference [41,72,73,75,76,197]. For example, even though each species had 81% missing data on average, we found that most species were placed in the families and genera expected based on previous taxonomy, often with very strong support. Furthermore, we found that incompleteness of terminal taxa is not related to branch lengths (at least not terminal branch lengths), suggesting that missing data are not significantly biasing branch-length estimates (see also [84,86,87]). Also, the ML models we used have been shown to be robust to missing data in large, sparse supermatrices [84].

Even though we did find some subfamilies and genera to be non-monophyletic, similar relationships were often found in previous studies based on data matrices with only limited missing data (e.g. non-monophyly of boid snake subfamilies [47], lacertid and scincid lizard genera [67,111], and scolecophidian, dipsadine, and natricine snake genera [38,43,186]). We suggest that further resolution of the squamate tree will be greatly facilitated if researchers deliberately sample mitochondrial genes and nuclear genes that include the set of genes used here and in recent phylogenomic studies (e.g. [20]), to increase overlap between genes and taxa, and decrease missing data.

With over 5000 species remaining to be included and only 12 genes sampled, our study is far from the last word on squamate phylogeny. We note that new data can easily be added to this matrix, in terms of both new taxa and new genes. Increased sampling of other nuclear genes is likely to be advantageous as well. Next-generation sequencing strategies and phylogenomic methods should help resolve difficult nodes [16,20,36,198-200], as should application of species-tree methods [201,202]. Species-tree analyses of 44 nuclear loci support many of these same clades across squamates [20], and data from >400 nuclear loci reinforces many of the relationships found here among the colubroid snake subfamilies (Pyron et al., in prep.). In addition, it would be useful to incorporate fossil taxa in future studies [15,19,22,86], utilizing the large morphological datasets that are now available [14,15]. Despite these areas for future studies, the present tree provides a framework for researchers analyzing patterns of squamate evolution at both lower and higher taxonomic levels (e.g. [10,11,203,204]), and for building a more complete picture of squamate phylogeny.

## Conclusions

In this study, we provide a phylogenetic estimate for 4161 squamate species, based on a supermatrix approach. Our results provide important confirmation for previous studies based on more limited taxon sampling, and reveal new relationships at the level of families, genera, and species. We also provide a phylogenetic framework for future comparative studies, with a large-scale tree including a common set of estimated branch lengths. Finally, we provide a revised classification for squamates based on this tree, including changes in the higher-level taxonomy of gymnophthalmid and scincid lizards and boid, colubrid, and lamprophiid snakes.

## Methods

### Initial classification

Our initial squamate classification is based on the June 2009 version of the Reptile Database [1] (http://www.reptile-database.org/), accessed in September of 2009 when this research was begun. Minor modifications to this scheme were made, primarily to update changes in colubroid snake taxonomy [41-44,205]. This initial taxonomic database consists of 8650 species (169 amphisbaenians, 5270 lizards, 3209 snakes, and 2 tuataras), against which the classification of species in the molecular sequence database was fixed. While modifications and updates (i.e. new species, revisions) have been made to squamate taxonomy subsequently, these are minor and should have no impact on our phylogenetic results. This database represents ~92% of the current estimated diversity of squamates (~9400 species as of December 2012).

Throughout the paper, we refer to the updated version of squamate taxonomy from the December 2012 update of the Reptile Database [1], incorporating major, well-accepted changes from recent studies (summarized in [1]). However, for large, taxonomic groups that have recently been broken up for reasons other than resolving paraphyly or matters of priority (e.g. in dactyloid and scincid lizards; see Results), we generally retain the older, more inclusive name in the interest of clarity, while providing references to the recent revision. We attempt to alter existing classifications as little as possible (see also [113]). Therefore, we generally only make changes when there is strong support for non-monophyly of currently recognized taxa and our proposed changes yield strongly supported monophyletic groups. Similarly, we only erect new taxa if they are strongly supported. Finally, although numerous genera are identified as being non-monophyletic in our tree, we refrain from changing genus-level taxonomy, given that our taxon sampling within many genera is limited.

### Molecular data

Preliminary literature searches were conducted to identify candidate genes for which a substantial number of squamate species were sequenced and available on GenBank (with the sampled species spread across multiple families), and which were demonstrably useful in previous phylogenetic studies of squamates (see Introduction for references). Twelve genes were identified as meeting these criteria: seven nuclear genes (brain-derived neurotrophic factor [BDNF], oocyte maturation factor [c-mos], neurotrophin-3 [NT3], phosducin [PDC], G protein-coupled receptor 35 [R35], and recombination-activating genes 1 [RAG-1] and 2 [RAG-2]); and five mitochondrial genes (12S/16S long and short subunit RNAs, cytochrome *b* [cyt-*b*], and nicotinamide adenine dehydrogenase subunits 2 [ND2] and 4 [ND4]). This sampling of genes does not include all available markers. For example, we omitted several nuclear and mitochondrial genes because they were available only for a limited subset of taxa. We also excluded tRNAs associated with the protein-coding sequences, given their short lengths and difficulty in alignment across the large time scales considered here.

To ensure maximal taxonomic coverage from the available data, searches were conducted on GenBank by family (stopping in October 2012), and the longest sequence for every species was gathered. Sequences totaling less than 250 bp for any species were not included. Only species in the taxonomic database were included in the sequence matrix, which resulted in the exclusion of numerous named taxa of ambiguous status, a few taxa described very recently, and many sequences labeled 'sp.' Some recently described phylogeographic lineages were also omitted. Species and GenBank accession numbers are available in Additional file 2: Table S1.

With respect to the December 2012 update of the Reptile Database [1], we sampled 52 of 183 amphisbaenian species (28%) from 11 of 19 (58%) genera; 2847 of 5799 lizard species (49%) from 448 of 499 genera (90%); and 1262 of 3434 snake species (39%) in 396 of 500 genera (80%). This yielded a total of 4161 species in 855 genera in the final matrix, 44% of the 9416 known, extant squamate species in 84% of 1018 genera [1]. The species-level classification of squamates is in constant flux, and the numbers of species and genera changed even as this paper was under review. The extant species of tuatara (*Sphenodon punctatus*) was included as a non-squamate outgroup taxon (see below). We acknowledge that our sampling of outgroup taxa is not extensive. However, placement of *Sphenodon* as the sister group to squamates is well-established by molecular analyses with extensive taxon sampling (e.g. [16,128,206]) and morphological data (e.g. [13]).

Alignment for protein-coding sequences was relatively straightforward. We converted them to amino acids, and then used the translation alignment algorithm in the program Geneious v4.8.4 (GeneMatters Corp.), with the default cost matrix (Blosum62) and gap penalties (open=12, extension=3). Alignments were relatively unambiguous after being trimmed for quality and maximum coverage (i.e. ambiguous end regions were removed, and most sequences began and ended at the same point).

For the ribosomal RNA sequences (12S and 16S sequences), alignment was more challenging. Preliminary global alignments using the algorithms MUSCLE [207] and CLUSTAL [208] under a variety of gap-cost parameters yielded low-quality results (i.e. alignments with large numbers of gaps and little overlap of potentially homologous characters). We subsequently employed a two-step strategy for these data. We first grouped sequences by higher taxa (i.e. Amphisbaenia, Anguimorpha, Gekkota, Iguania, Scincomorpha, and Serpentes, though these are not all monophyletic as previously defined), for which alignments were relatively straightforward under the default MUSCLE parameters.

These were then combined using the profile alignment feature of MUSCLE, and the global alignment was subsequently updated using the "refine alignment" option. Minor adjustments were then made by eye, and ambiguously aligned end-regions were trimmed for maximum coverage and quality. We did not include partitions for stems and loops for the ribosomal sequences, although this has been shown to improve model fit in previous squamate studies (e.g. [82]). Although it is possible to assign individual nucleotide positions to these partitions, this would have been challenging given the large number of sequences, and the potential for stems and loops to shift across the many species and large time scales involved.

Each species was represented by a single terminal taxon in the matrix. In many cases, sequences from multiple individuals of the same species were combined, to allow us to combine data from different genes for the same species. We acknowledge the possibility that in some cases this approach may cause us to combine genes from different species in the same terminal taxon (e.g. due to changing taxonomy or incorrect identifications). Additionally, many sequences are not from vouchered specimens, and it is possible that misidentified species are present on GenBank and in our matrix. However, most of our data came from lower-level phylogenetic studies, in which the identification of species by previous authors should be highly accurate. In addition, any such mistakes should be among closely related species, and lead to minimal phylogenetic distortion, as the grossest errors are easily identified.

Some species were removed after preliminary analyses, due either to obvious sequencing errors (e.g. high BLAST homology with unrelated families or non-squamates, excessive ambiguities) or a lack of overlap in genes sampled with other members of the same genus (leading to seemingly artificial paraphyly). We also excluded species with identical sequences between taxa across all genes, arbitrarily choosing the first taxon in alphabetical order to remain in the matrix. Additionally, we also removed a few apparent "rogue taxa" [75,77]. These were identified by their poor support and suspect placement (e.g. in a clearly incorrect family), and were typically represented in the matrix by short fragments of single genes (e.g. an ND4 fragment from the enigmatic colubroid snake *Oreocalamus hanitchsi*).

The final combined matrix contained sequence data for: 2335 species for 12S (including 56% of all 4162 taxa, 1395 bp), 2377 for 16S (57%, 1970 bp), 730 for BDNF (18%, 714 bp), 1671 for c-mos (40%, 903 bp), 1985 for cyt-b (48%, 1000 bp), 437 for NT3 (10%, 675 bp), 1860 for ND2 (45%, 960 bp), 1556 for ND4 (37%, 696 bp), 393 for PDC (9%, 395 bp), 401 for R35 (10%, 768 bp), 1379 for RAG-1 (33%, 2700 bp), and 471 for RAG2 (11%, 720 bp). The total alignment consists of 12896 bp for 4162 taxa (4161 squamates and 1 outgroup). The mean length is 2497 bp of sequence data present per species from 3.75 genes (19% of the total matrix length of 12896 bp, or 81% missing data), and ranges from 270–11153 bp (2–86% complete). The matrix and phylogeny (see below) are available in DataDryad repository 10.5061/dryad.82h0m.

Clearly, many taxa had large amounts of missing data (some >95%), and on average each species had 81% missing cells. However, several lines of evidence suggest that these missing data are not generally problematic. First, a large body of empirical and theoretical studies has shown that highly incomplete taxa can be accurately placed in model-based phylogenetic analyses (and with high levels of branch support), especially if a large number of characters have been sampled (recent reviews in [84,85]). Second, several recent empirical studies have shown that the supermatrix approach (with extensive missing data in some taxa) yields generally well-supported large-scale trees that are generally congruent with previous taxonomies and phylogenetic estimates (e.g. [41,48,72,73,75,76,197]). Third, recent studies have also shown that there is generally little relationship between the amount of missing data in individual taxa and the support for their placement on the tree [41,73,85]. Finally, we note that some highly incomplete taxa were unstable in their placement (“rogue taxa;" [75]), but these were removed prior to the analysis of the final matrix (see above).

Our sampling design should be especially robust to the impacts of missing data for several reasons. Most importantly, most terminal taxon (species) had substantial data present (mean of 2497 bp per species) regardless of the number of missing data cells. Simulations (see reviews in [84,85]) suggest that the amount of data present is a key parameter in determining the accuracy with which incomplete taxa are placed in phylogenies, not the amount of data absent. Additionally, several genes (e.g. 12S/16S, cyt-*b*, and c-mos) were shared by many (>40%) of taxa. Thus, there was typically extensive overlap among the genes present for each taxon (as also indicated by the mean bp per species being much greater than the length of most genes). Limited overlap in gene sampling among taxa could be highly problematic, irrespective of the amount of missing data *per se*, but this does not appear to be a problem in our dataset. Finally, several nuclear genes (e.g. BDNF, c-mos, R35, and RAG-1) were congruently sampled in previous studies to represent most (>80%) squamate families and subfamilies (e.g. [20]), providing a scaffold of well-sampled taxa spanning all major clades, as recommended by recent authors [84].

### Phylogenetic analyses

We performed phylogenetic analyses of the 12-gene concatenated matrix using Maximum Likelihood (ML). We assessed node support using the non-parametric Shimodaira-Hasegawa-Like (SHL) implementation of the approximate likelihood-ratio test (aLRT; [94]). This involved a two-stage strategy. We first performed initial ML tree- inference using the program RAxML-Light v1.0.7 [209], a modification of the original RAxML algorithm [210]. This program uses the GTRCAT strategy for all genes and partitions, a high-speed approximation of the GTR+Γ model (general time-reversible with gamma-distribution of rate heterogeneity among sites). The GTR model is the only substitution model implemented in RAxML [210], and all other substitution models are simply special cases of the GTR model [211]. Previous analyses suggest that GTR is generally the best-fitting model for these genes and that they should be partitioned by gene and codon position [16,17,19,20,36,81].

To generate an initial ML estimate for final optimization and support estimation, we performed 11 ML searches from 11 parsimony starting trees generated under the default parsimony model in RAxMLv7.2.8. This number is likely to be sufficient when datasets contain many characters that have strong phylogenetic signal (A. Stamatakis, *pers. comm.*). Additionally, the dataset was analyzed with these settings (GTRCAT search from a randomized parsimony starting tree) numerous times (>20) as the final matrix was assembled and tested, representing hundreds of independent searches from random starting points. All of the estimated trees from these various analyses showed high overall congruence with the final topology. The concordance between the preliminary and final results suggests that the tree was not strongly impacted by searches stuck on local optima, and that it should be a good approximation of the ML tree.

We then performed a final topology optimization and assessed support. We passed our best ML estimate of the phylogeny (based on GTRCAT) from RAxML-Light to RAxMLv7.2.8, which does an additional search (using the GTRGAMMA model) to produce a nearest-neighbor interchange (NNI)-optimized estimate of the ML tree. This optimization is needed to calculate the SHL version of the aLRT for estimating support values [94]. The SHL-aLRT strategy approximates a test of the null hypothesis that the branch length subtending each node equals 0 (i.e. that the node can be resolved, rather than estimated as a polytomy) with a test of the more general null hypothesis that "the branch is incorrect" relative to the four next suboptimal arrangements of that node relative to the NNI-optimal arrangement [94]. Based on initial analyses, generating sufficient ML bootstrap replicates for a tree of this size proved computationally intractable, so we rely on SHL values alone to assess support.

The SHL approach has at least two major advantages over non-parametric bootstrapping for large ML trees: (i) values are apparently robust to many potential model violations and have the same properties as bootstrap proportions for all but the shortest branches [41,94,212], and (ii) values for short branches may be more accurate than bootstrap proportions, as support is evaluated based on whole-alignment likelihoods, rather than the frequency of re-sampled characters [94,213]. Additionally, the SHL approach is orders of magnitude faster than traditional bootstrapping [94,212,213], and it appears to be similarly robust to matrices with extensive missing data [41]. As in previous studies, we take a conservative view, considering SHL values of 85 or greater (i.e. a 15% chance that a branch is "incorrect") as strong support [41,212,213].

These analyses were performed on a 360-core SGI ICE supercomputing system ("ANDY") at the High Performance Computing Center at the City University of New York (CUNY). The final analysis was completed in 8.8 days of computer time using 188 nodes of the CUNY supercomputing cluster.

Finally, we assessed the potential impact of missing data on our branch-length estimates. We performed linear regression (in R) of the proportional completeness of each terminal taxa (non-missing data in bp / maximum amount of non-missing data, 12896 bp) against the length of its terminal branch. This test addresses whether incomplete taxa have branch-length estimates that are consistently biased in one direction (shorter vs. longer) relative to more complete terminals. However, it does not directly test whether branch length estimates are correct or not, nor how branch length estimates are impacted by replacing non-missing data with missing data (see [87] for results suggesting that such replacements have little effect in real data sets).

## Appendix I

Note that we only provide here an account for the one subfamily newly erected in this study. We do not provide accounts for subfamilies with changes in content (Boinae, Erycinae, Dipsadinae, Pseudaspidinae, Scincinae, Ungaliophiinae), that have been resurrrected (Sanziniinae), or that represent elevation of lower-ranked taxa (tribe Bachiini here recognized as Bachiinae).

### Candoiinae *subfam. nov.* (family Boidae)

*Type:* genus and species *Candoia carinata*[214].

*Content:* one genus, 4 species; *C. aspera*, *C. bibroni*, *C. carinata*, *C. paulsoni.*

*Definition:* this subfamily consists of the most recent common ancestor of the extant species of *Candoia*, and all its descendants. These species are morphologically distinguished in part from other boid snakes by a flattened rostrum leading to an angular snout [215] and a wide premaxillary floor [167].

*Distribution:* these snakes are primarily restricted to the South Pacific islands of New Guinea and Melanesia, and the eastern Indonesian archipelago [150].

*Remarks:* the three species from this subfamily that are sampled in our tree are strongly supported as monophyletic (SHL = 100), and are well supported (SHL = 87) as the sister taxon to a moderately supported clade consisting of Erycinae + Boinae (SHL = 83).

### Proposed Generic Composition of Higher Taxa

Below, we list the familial and subfamilial assignment of all squamate genera from the December, 2012 update of the Reptile Database [1], updated to reflect some recent changes and the proposed subfamily level changes listed above. As this classification includes numerous taxa not sampled in our tree, we deal with them conservatively. For traditionally recognized families and subfamilies that we found to be monophyletic, we include all taxa traditionally assigned to them. Taxa are denoted *incertae sedis* if they are of ambiguous familial or subfamilial assignment due to uncertain placement in our tree, or due to absence from our tree and lack of assignment by previous authors. This classification includes 67 families and 56 subfamilies, and accounts for >9400 squamate species in 1018 genera [1]. Higher taxa are listed (more-or-less) phylogenetically (starting closest to the root; Figure 1), families are listed alphabetically within higher taxa, and subfamilies and genera are listed alphabetically within families.

## Squamata

### Dibamidae (*Anelytropsis, Dibamus*)

### Gekkota

**Carphodactylidae** (*Carphodactylus, Nephrurus, Orraya, Phyllurus, Saltuarius, Underwoodisaurus, Uvidicolus*); **Diplodactylidae** (*Amalosia, Bavayia, Correlophus, Crenadactylus, Dactylocnemis, Dierogekko, Diplodactylus, Eurydactylodes, Hesperoedura, Hoplodactylus, Lucasium, Mniarogekko, Mokopirirakau, Naultinus, Nebulifera, Oedodera, Oedura, Paniegekko, Pseudothecadactylus, Rhacodactylus, Rhynchoedura, Strophurus, Toropuku, Tukutuku, Woodworthia*); **Eublepharidae** (*Aeluroscalabotes, Coleonyx, Eublepharis, Goniurosaurus, Hemitheconyx, Holodactylus*); **Gekkonidae** (*Afroedura, Afrogecko, Agamura, Ailuronyx, Alsophylax, Asiocolotes, Blaesodactylus, Bunopus, Calodactylodes, Chondrodactylus, Christinus, Cnemaspis, Colopus, Crossobamon, Cryptactites, Cyrtodactylus, Cyrtopodion, Dixonius, Ebenavia, Elasmodactylus, Geckolepis, Gehyra, Gekko, Goggia, Hemidactylus, Hemiphyllodactylus, Heteronotia, Homopholis, Lepidodactylus, Luperosaurus, Lygodactylus, Matoatoa, Mediodactylus, Nactus, Narudasia, Pachydactylus, Paragehyra, Paroedura, Perochirus, Phelsuma, Pseudoceramodactylus, Pseudogekko, Ptenopus, Ptychozoon, Rhinogecko, Rhoptropella, Rhoptropus, Stenodactylus, Tropiocolotes, Urocotyledon, Uroplatus*); **Phyllodactylidae** (*Asaccus, Gymnodactylus, Haemodracon, Homonota, Phyllodactylus, Phyllopezus, Ptyodactylus, Tarentola, Thecadactylus*); **Pygopodidae** (*Aprasia, Delma, Lialis, Ophidiocephalus, Paradelma, Pletholax, Pygopus*); **Sphaerodactylidae** (*Aristelliger, Chatogekko, Coleodactylus, Euleptes, Gonatodes, Lepidoblepharis, Pristurus, Pseudogonatodes, Quedenfeldtia, Saurodactylus, Sphaerodactylus, Teratoscincus*)

### Scincoidea

**Cordylidae,** Cordylinae (*Chamaesaura, Cordylus, Hemicordylus, Karusasaurus, Namazonurus, Ninurta, Ouroborus, Pseudocordylus, Smaug*), Platysaurinae (*Platysaurus*); **Gerrhosauridae**, Gerrhosaurinae (*Cordylosaurus*, *Gerrhosaurus*, *Tetradactylus*), Zonosaurinae (*Tracheloptychus*, *Zonosaurus*); **Scincidae**, Acontiinae (*Acontias*, *Typhlosaurus*), Lygosominae (*Ablepharus, Afroablepharus, Anomalopus, Asymblepharus, Ateuchosaurus, Bartleia, Bassiana, Bellatorias, Caledoniscincus, Calyptotis, Carlia, Cautula, Celatiscincus, Chioninia, Coeranoscincus, Coggeria, Cophoscincopus, Corucia, Cryptoblepharus, Ctenotus, Cyclodomorphus, Dasia, Egernia, Emoia, Eremiascincus, Eroticoscincus, Eugongylus, Eulamprus, Eumecia, Eutropis, Fojia, Geomyersia, Geoscincus, Glaphyromorphus, Gnypetoscincus, Graciliscincus, Haackgreerius, Hemiergis, Hemisphaeriodon, Insulasaurus, Isopachys, Kaestlea, Kanakysaurus, Lacertaspis, Lacertoides, Lamprolepis, Lampropholis, Lankascincus, Larutia, Leiolopisma, Lepidothyris, Leptoseps, Leptosiaphos, Lerista, Liburnascincus, Liopholis, Lioscincus, Lipinia, Lissolepis, Lobulia, Lygisaurus, Lygosoma, Mabuya, Marmorosphax, Menetia, Mochlus, Morethia, Nangura, Nannoscincus, Niveoscincus, Notoscincus, Oligosoma, Ophioscincus, Otosaurus, Panaspis, Papuascincus, Parvoscincus, Phoboscincus, Pinoyscincus, Prasinohaema, Proablepharus, Pseudemoia, Ristella, Saiphos, Saproscincus, Scincella, Sigaloseps, Simiscincus, Sphenomorphus, Tachygyia, Tiliqua, Trachylepis, Tribolonotus, Tropidophorus, Tropidoscincus, Tytthoscincus, Vietnascincus*), Scincinae (*Amphiglossus, Androngo, Barkudia, Brachymeles, Chabanaudia, Chalcides, Chalcidoseps, Eumeces, Eurylepis, Feylinia, Gongylomorphus, Hakaria, Janetaescincus, Jarujinia, Madascincus, Melanoseps, Mesoscincus, Nessia, Ophiomorus, Pamelaescincus, Paracontias, Plestiodon, Proscelotes, Pseudoacontias, Pygomeles, Scelotes, Scincopus, Scincus, Scolecoseps, Sepsina, Sepsophis, Sirenoscincus, Typhlacontias, Voeltzkowia*); **Xantusiidae**, Cricosaurinae (*Cricosaura*), Lepidophyminae (*Lepidophyma*), Xantusiinae (*Xantusia*)

### Lacertoidea (including Amphisbaenia)

**Amphisbaenidae** (*Amphisbaena, Ancylocranium, Baikia, Chirindia, Cynisca, Dalophia, Geocalamus, Loveridgea, Mesobaena, Monopeltis, Zygaspis*); **Bipedidae** (*Bipes*); **Blanidae** (*Blanus*); **Cadeidae** (*Cadea*); **Gymnophthalmidae**, Alopoglossinae (*Alopoglossus*, *Ptychoglossus*), Bachiinae (*Bachia*), Cercosaurinae (*Anadia, Cercosaura, Echinosaura, Euspondylus, Macropholidus, Neusticurus, Opipeuter, Petracola, Pholidobolus, Placosoma, Potamites, Proctoporus, Riama, Riolama, Teuchocercus*), Ecpleopinae (*Adercosaurus, Amapasaurus, Anotosaura, Arthrosaura, Colobosauroides, Dryadosaura, Ecpleopus, Kaieteurosaurus, Leposoma, Marinussaurus, Pantepuisaurus*), Gymnophthalminae (*Acratosaura, Alexandresaurus, Calyptommatus, Caparaonia, Colobodactylus, Colobosaura, Gymnophthalmus, Heterodactylus, Iphisa, Micrablepharus, Nothobachia, Procellosaurinus, Psilophthalmus, Scriptosaura, Stenolepis, Tretioscincus, Vanzosaura*), Rhachisaurinae (*Rhachisaurus*); **Lacertidae**, Gallotiinae (*Gallotia*, *Psammodromus*), Lacertinae (*Acanthodactylus, Adolfus, Algyroides, Anatololacerta, Apathya, Archaeolacerta, Atlantolacerta, Australolacerta, Congolacerta, Dalmatolacerta, Darevskia, Dinarolacerta, Eremias, Gastropholis, Heliobolus, Hellenolacerta, Holaspis, Iberolacerta, Ichnotropis, Iranolacerta, Lacerta, Latastia, Meroles, Mesalina, Nucras, Omanosaura, Ophisops, Parvilacerta, Pedioplanis, Philochortus, Phoenicolacerta, Podarcis, Poromera, Pseuderemias, Scelarcis, Takydromus, Teira, Timon, Tropidosaura, Zootoca*); **Rhineuridae** (*Rhineura*); **Teiidae**, Teiinae (*Ameiva*, *Aspidoscelis*, *Cnemidophorus*, *Dicrodon*, *Kentropyx*, *Teius*), Tupinambinae (*Callopistes*, *Crocodilurus*, *Dracaena*, *Tupinambis*); **Trogonophiidae** (*Agamodon*, *Diplometopon*, *Pachycalamus*, *Trogonophis*)

### Iguania

**Agamidae**, Agaminae (*Acanthocercus, Agama, Brachysaura, Bufoniceps, Laudakia, Phrynocephalus, Pseudotrapelus, Trapelus, Xenagama*), Amphibolurinae (*Amphibolurus, Chelosania, Chlamydosaurus, Cryptagama, Ctenophorus, Diporiphora, Hypsilurus, Intellagama, Lophognathus, Moloch, Physignathus, Pogona, Rankinia, Tympanocryptis*), Draconinae (*Acanthosaura, Aphaniotis, Bronchocela, Calotes, Ceratophora, Complicitus, Cophotis, Coryphophylax, Dendragama, Draco, Gonocephalus, Harpesaurus, Hypsicalotes, Japalura, Lophocalotes, Lyriocephalus, Mantheyus, Oriocalotes, Otocryptis, Phoxophrys, Psammophilus, Pseudocalotes, Pseudocophotis, Ptyctolaemus, Salea, Sitana, Thaumatorhynchus*), Hydrosaurinae (*Hydrosaurus*), Leiolepidinae (*Leiolepis*), Uromastycinae (*Uromastyx*); **Chamaeleonidae**, Brookesiinae (*Brookesia*), Chamaeleoninae (*Archaius, Bradypodion, Calumma, Chamaeleo, Furcifer, Kinyongia, Nadzikambia, Rhampholeon, Rieppeleon, Trioceros*); **Corytophanidae** (*Basiliscus*, *Corytophanes*, *Laemanctus*); **Crotaphytidae** (*Crotaphytus*, *Gambelia*); **Dactyloidae** (*Anolis*); **Hoplocercidae** (*Enyalioides*, *Hoplocercus*, *Morunasaurus*); **Iguanidae** (*Amblyrhynchus*, *Brachylophus*, *Conolophus*, *Ctenosaura*, *Cyclura*, *Dipsosaurus*, *Iguana*, *Sauromalus*); **Leiocephalidae** (*Leiocephalus*); **Leiosauridae**, Enyaliinae (*Anisolepis*, *Enyalius*, *Urostrophus*), Leiosaurinae (*Diplolaemus*, *Leiosaurus*, *Pristidactylus*); **Liolaemidae** (*Ctenoblepharys*, *Liolaemus*, *Phymaturus*); **Opluridae** (*Chalarodon*, *Oplurus*); **Phrynosomatidae** (*Callisaurus*, *Cophosaurus*, *Holbrookia*, *Petrosaurus*, *Phrynosoma*, *Sceloporus*, *Uma*, *Urosaurus*, *Uta*); **Polychrotidae** (*Polychrus*); **Tropiduridae** (*Eurolophosaurus*, *Microlophus*, *Plica*, *Stenocercus*, *Strobilurus*, *Tropidurus*, *Uracentron*, *Uranoscodon*)

### Anguimorpha

**Anguidae**, Anguinae (*Anguis*, *Dopasia*, *Ophisaurus*, *Pseudopus*), Diploglossinae (*Celestus*, *Diploglossus*, *Ophiodes*), Gerrhonotinae (*Abronia*, *Barisia*, *Coloptychon*, *Elgaria*, *Gerrhonotus*, *Mesaspis*); **Anniellidae** (*Anniella*); **Helodermatidae** (*Heloderma*); **Lanthanotidae** (*Lanthanotus*); **Shinisauridae** (*Shinisaurus*); **Varanidae** (*Varanus*); **Xenosauridae** (*Xenosaurus*)

### Serpentes

**Acrochordidae** (*Acrochordus*); **Aniliidae** (*Anilius*); **Anomalepididae** (*Anomalepis*, *Helminthophis*, *Liotyphlops*, *Typhlophis*); **Anomochilidae** (*Anomochilus*); **Boidae**, Boinae (*Boa*, *Corallus*, *Epicrates*, *Eunectes*), Candoiinae (*Candoia*), Erycinae (*Eryx*), Sanziniinae (*Acrantophis*, *Sanzinia*), Ungaliophiinae (*Charina*, *Exiliboa*, *Lichanura*, *Ungaliophis*); **Bolyeriidae** (*Bolyeria*, *Casarea*); **Calabariidae** (*Calabaria*); **Colubridae***incertae sedis* (*Blythia*, *Cyclocorus*, *Elapoidis*, *Gongylosoma*, *Helophis*, *Myersophis*, *Oreocalamus, Poecilopholis*, *Rhabdops*, *Tetralepis*), Calamariinae (*Calamaria*, *Calamorhabdium*, *Collorhabdium*, *Etheridgeum*, *Macrocalamus*, *Pseudorabdion*, *Rabdion*), Colubrinae (*Aeluroglena, Ahaetulla, Aprosdoketophis, Archelaphe, Argyrogena, Arizona, Bamanophis, Bogertophis, Boiga, Cemophora, Chilomeniscus, Chionactis, Chironius, Chrysopelea, Coelognathus, Coluber, Colubroelaps, Conopsis, Coronella, Crotaphopeltis, Cyclophiops, Dasypeltis, Dendrelaphis, Dendrophidion, Dipsadoboa, Dispholidus, Dolichophis, Drymarchon, Drymobius, Drymoluber, Dryocalamus, Dryophiops, Eirenis, Elachistodon, Elaphe, Euprepiophis, Ficimia, Geagras, Gonyophis, Gonyosoma, Gyalopion, Hapsidophrys, Hemerophis, Hemorrhois, Hierophis, Lampropeltis, Leptodrymus, Leptophis, Lepturophis, Limnophis, Liopeltis, Lycodon, Lytorhynchus, Macroprotodon, Mastigodryas, Meizodon, Oligodon, Oocatochus, Opheodrys, Oreocryptophis, Orthriophis, Oxybelis, Pantherophis, Philothamnus, Phyllorhynchus, Pituophis, Platyceps, Pseudelaphe, Pseudoficimia, Pseustes, Ptyas, Rhadinophis, Rhamnophis, Rhinechis, Rhinobothryum, Rhinocheilus, Rhynchocalamus, Rhynchophis, Salvadora, Scaphiophis, Scolecophis, Senticolis, Simophis, Sonora, Spalerosophis, Spilotes, Stegonotus, Stenorrhina, Symphimus, Sympholis, Tantilla, Tantillita, Telescopus, Thelotornis, Thrasops, Toxicodryas, Trimorphodon, Xenelaphis, Xyelodontophis, Zamenis*), Dipsadinae (*Adelphicos, Alsophis, Amastridium, Amnesteophis, Antillophis, Apostolepis, Arrhyton, Atractus, Boiruna, Borikenophis, Caaeteboia, Calamodontophis, Caraiba, Carphophis, Cercophis, Chapinophis, Chersodromus, Clelia, Coniophanes, Conophis, Contia, Coronelaps, Crisantophis, Cryophis, Cubophis, Darlingtonia, Diadophis, Diaphorolepis, Dipsas, Ditaxodon, Drepanoides, Echinanthera, Elapomorphus, Emmochliophis, Enuliophis, Enulius, Erythrolamprus, Farancia, Geophis, Gomesophis, Haitiophis, Helicops, Heterodon, Hydrodynastes, Hydromorphus, Hydrops, Hypsiglena, Hypsirhynchus, Ialtris, Imantodes, Leptodeira, Lioheterophis, Lygophis, Magliophis, Manolepis, Mussurana, Ninia, Nothopsis, Ocyophis, Omoadiphas, Oxyrhopus, Paraphimophis, Phalotris, Philodryas, Phimophis, Plesiodipsas, Pliocercus, Pseudalsophis, Pseudoboa, Pseudoeryx, Pseudoleptodeira, Pseudotomodon, Psomophis, Ptychophis, Rhachidelus, Rhadinaea, Rhadinella, Rhadinophanes, Rodriguesophis, Saphenophis, Schwartzophis, Sibon, Sibynomorphus, Siphlophis, Sordellina, Synophis, Tachymenis, Taeniophallus, Tantalophis, Thamnodynastes, Thermophis, Tomodon, Tretanorhinus, Trimetopon, Tropidodipsas, Tropidodryas, Uromacer, Uromacerina, Urotheca, Xenodon, Xenopholis*), Grayiinae (*Grayia*), Natricinae (*Adelophis, Afronatrix, Amphiesma, Amphiesmoides, Anoplohydrus, Aspidura, Atretium, Balanophis, Clonophis, Hologerrhum, Hydrablabes, Hydraethiops, Iguanognathus, Lycognathophis, Macropisthodon, Natriciteres, Natrix, Nerodia, Opisthotropis, Parahelicops, Pararhabdophis, Paratapinophis, Regina, Rhabdophis, Seminatrix, Sinonatrix, Storeria, Thamnophis, Trachischium, Tropidoclonion, Tropidonophis, Virginia, Xenochrophis*), Pseudoxenodontinae (*Plagiopholis*, *Pseudoxenodon*), Sibynophiinae (*Scaphiodontophis*, *Sibynophis*); **Cylindrophiidae** (*Cylindrophis*); **Elapidae** (*Acanthophis, Aipysurus, Aspidelaps, Aspidomorphus, Austrelaps, Bungarus, Cacophis, Calliophis, Cryptophis, Demansia, Dendroaspis, Denisonia, Drysdalia, Echiopsis, Elapognathus, Elapsoidea, Emydocephalus, Ephalophis, Furina, Hemachatus, Hemiaspis, Hemibungarus, Hoplocephalus, Hydrelaps, Hydrophis, Kolpophis, Laticauda, Loveridgelaps, Maticora, Micropechis, Micruroides, Micrurus, Naja, Notechis, Ogmodon, Ophiophagus, Oxyuranus, Parahydrophis, Parapistocalamus, Parasuta, Pseudechis, Pseudohaje, Pseudolaticauda, Pseudonaja, Rhinoplocephalus, Salomonelaps, Simoselaps, Sinomicrurus, Suta, Thalassophis, Toxicocalamus, Tropidechis, Vermicella, Walterinnesia*); **Gerrhopilidae** (*Gerrhopilus*); **Homalopsidae** (*Bitia*, *Brachyorrhos*, *Cantoria*, *Cerberus*, *Djokoiskandarus*, *Enhydris*, *Erpeton*, *Fordonia*, *Gerarda*, *Heurnia*, *Homalopsis*, *Myron*, *Pseudoferania*); **Lamprophiidae***incertae sedis* (*Micrelaps*, *Montaspis*, *Oxyrhabdium*), Aparallactinae (*Amblyodipsas*, *Aparallactus*, *Brachyophis*, *Chilorhinophis*, *Elapotinus*, *Hypoptophis*, *Macrelaps*, *Polemon*, *Xenocalamus*), Atractaspidinae (*Atractaspis*, *Homoroselaps*), Lamprophiinae (*Boaedon*, *Bothrophthalmus*, *Chamaelycus*, *Dendrolycus*, *Gonionotophis*, *Hormonotus*, *Inyoka*, *Lamprophis*, *Lycodonomorphus*, *Lycophidion*, *Pseudoboodon*), Prosymninae (*Prosymna*), Psammophiinae (*Dipsina*, *Hemirhagerrhis*, *Malpolon*, *Mimophis*, *Psammophis*, *Psammophylax*, *Rhagerhis*, *Rhamphiophis*), Pseudaspidinae (*Buhoma, Psammodynastes*, *Pseudaspis*, *Pythonodipsas*), Pseudoxyrhophiine (*Alluaudina*, *Amplorhinus*, *Bothrolycus*, *Brygophis*, *Compsophis*, *Ditypophis*, *Dromicodryas*, *Duberria*, *Exallodontophis*, *Heteroliodon*, *Ithycyphus*, *Langaha*, *Leioheterodon*, *Liophidium*, *Liopholidophis*, *Lycodryas*, *Madagascarophis*, *Micropisthodon*, *Pararhadinaea*, *Parastenophis*, *Phisalixella*, *Pseudoxyrhopus*, *Thamnosophis*); **Leptotyphlopidae** (*Epacrophis*, *Epictia*, *Leptotyphlops*, *Mitophis*, *Myriopholis*, *Namibiana*, *Rena*, *Rhinoleptus*, *Siagonodon*, *Tetracheilostoma*, *Tricheilostoma*, *Trilepida*); **Loxocemidae** (*Loxocemus*); **Pareatidae** (*Aplopeltura*, *Asthenodipsas*, *Pareas*); **Pythonidae** (*Antaresia*, *Apodora*, *Aspidites*, *Bothrochilus*, *Broghammerus*, *Leiopython*, *Liasis*, *Morelia*, *Python*); **Tropidophiidae** (*Trachyboa*, *Tropidophis*); **Typhlopidae** (*Acutotyphlops*, *Afrotyphlops*, *Austrotyphlops*, *Cyclotyphlops*, *Grypotyphlops*, *Letheobia*, *Megatyphlops*, *Ramphotyphlops*, *Rhinotyphlops*, *Typhlops*); **Uropeltidae** (*Brachyophidium*, *Melanophidium*, *Platyplectrurus*, *Plectrurus*, *Pseudotyphlops*, *Rhinophis*, *Teretrurus*, *Uropeltis*); **Viperidae**, Azemiopinae (*Azemiops*), Crotalinae (*Agkistrodon*, *Atropoides*, *Bothriechis*, *Bothriopsis*, *Bothrocophias*, *Bothropoides*, *Bothrops*, *Calloselasma*, *Cerrophidion*, *Crotalus*, *Deinagkistrodon*, *Garthius*, *Gloydius*, *Hypnale*, *Lachesis*, *Mixcoatlus*, *Ophryacus*, *Ovophis*, *Porthidium*, *Protobothrops*, *Rhinocerophis*, *Sistrurus*, *Trimeresurus*, *Tropidolaemus*), Viperinae (*Atheris*, *Bitis*, *Causus*, *Cerastes*, *Daboia*, *Echis*, *Eristicophis*, *Macrovipera*, *Montatheris*, *Montivipera*, *Proatheris*, *Pseudocerastes*, *Vipera*); **Xenodermatidae** (*Achalinus*, *Fimbrios*, *Stoliczkia*, *Xenodermus*, *Xylophis*); **Xenopeltidae** (*Xenopeltis*); **Xenophidiidae** (*Xenophidion*); **Xenotyphlopidae** (*Xenotyphlops*)

## Competing interests

The authors declare that they have no competing interests.

## Authors’ contributions

RAP and JJW conceived the study. RAP, FTB, and JJW conducted analyses and checked results. RAP, FTB, and JJW wrote the MS. All authors read and approved the final manuscript.

## Supplementary Material

Additional file 1: Data File S1 The 4162-species ML phylogeny in Newick format; taxonomic changes are given in Additional file 2: Table S1. Click here for file

Additional file 2: Table S1 GenBank accession numbers for all taxa included in this analysis. Click here for file
