# Supplementary material for: A phylogeny and revised classification of Squamata, including 4161 species of lizards and snakes
Source: BMC Evol Biol. 2013 Apr 29;13:93. doi: 10.1186/1471-2148-13-93 (PMC3682911; doi:10.1186/1471-2148-13-93)
Supplement: Additional file 2: Table S1 — GenBank accession numbers for all taxa included in this analysis. [file 1471-2148-13-93-S2.docx]

**Appendix S1.** GenBank accession numbers for 4161 sampled squamate taxa and the outgroup *Sphenodon punctatus*. Numerous taxa underwent name changes between the 2009 and 2012 updates of the Reptile Database; the differences between the tree (Fig. 2) and the matrix (and Newick tree file) are given at the end of this document, with the matrix name (2009) first, and the tree name (2012) last.

| **Species** | **12S** | **16S** | **BDNF** | **CMOS** | **cytb** | **ND2** | **ND4** | **NT3** | **PDC** | **R35** | **RAG1** | **RAG2** |
| --- | --- | --- | --- | --- | --- | --- | --- | --- | --- | --- | --- | --- |
| *Ablepharus budaki* | -- | AY561432 | -- | -- | -- | -- | -- | -- | -- | -- | -- | -- |
| *Ablepharus chernovi* | -- | AY561433 | -- | -- | -- | -- | -- | -- | -- | -- | -- | -- |
| *Ablepharus kitaibelii* | AY308325 | AY561419 | -- | -- | -- | -- | -- | -- | -- | -- | -- | -- |
| *Ablepharus pannonicus* | -- | -- | -- | AF039466 | -- | AY607280 | -- | -- | -- | -- | -- | -- |
| *Abronia anzuetoi* | AF056602 | -- | -- | -- | AF056587 | -- | -- | -- | -- | -- | -- | -- |
| *Abronia aurita* | AF056616 | -- | -- | -- | AF056588 | -- | -- | -- | -- | -- | -- | -- |
| *Abronia campbelli* | AF056605 | -- | -- | -- | AF056590 | -- | -- | -- | -- | -- | -- | -- |
| *Abronia chiszari* | -- | -- | -- | -- | -- | -- | AY605101 | -- | -- | -- | -- | -- |
| *Abronia fimbriata* | AF056606 | -- | -- | -- | AF056591 | -- | -- | -- | -- | -- | -- | -- |
| *Abronia frosti* | AF056607 | -- | -- | -- | AF056592 | -- | -- | -- | -- | -- | -- | -- |
| *Abronia graminea* | NC_005958 | AB080273 | -- | -- | NC_005958 | NC_005958 | AY605102 | JQ844947 | -- | -- | -- | -- |
| *Abronia lythrochila* | AF056608 | -- | -- | -- | AF056593 | -- | -- | -- | -- | -- | -- | -- |
| *Abronia matudai* | AF056609 | -- | -- | -- | AF056594 | -- | -- | -- | -- | -- | -- | -- |
| *Abronia mixteca* | AF056610 | -- | -- | -- | AF056595 | -- | -- | -- | -- | -- | -- | -- |
| *Abronia oaxacae* | -- | -- | -- | -- | -- | AF085615 | -- | -- | -- | -- | -- | -- |
| *Abronia ornelasi* | AF056611 | -- | -- | -- | AF056596 | -- | -- | -- | -- | -- | -- | -- |
| *Acalyptophis peronii* | -- | DQ234006 | -- | FJ587180 | DQ233925 | -- | FJ593204 | -- | -- | -- | -- | -- |
| *Acanthocercus atricollis* | -- | GU128463 | -- | -- | -- | -- | -- | -- | -- | -- | -- | -- |
| *Acanthodactylus aureus* | AF197485 | AF197486 | -- | -- | -- | -- | -- | -- | -- | -- | -- | -- |
| *Acanthodactylus beershebensis* | JF912449 | JF912448 | -- | -- | -- | -- | -- | -- | -- | -- | -- | -- |
| *Acanthodactylus blanci* | AY633406 | AY633431 | -- | -- | -- | -- | -- | -- | -- | -- | -- | -- |
| *Acanthodactylus boskianus* | GU225705 | GU433291 | -- | EF632251 | HM778107 | -- | HM778100 | -- | -- | -- | EF632206 | -- |
| *Acanthodactylus busacki* | EU086869 | EU086903 | -- | -- | -- | -- | -- | -- | -- | -- | -- | -- |
| *Acanthodactylus cantoris* | AF080344 | AF080346 | -- | -- | -- | -- | -- | -- | -- | -- | -- | -- |
| *Acanthodactylus erythrurus* | AF206607 | AY633436 | -- | HQ605874 | HQ605832 | -- | -- | -- | -- | -- | HQ616540 | -- |
| *Acanthodactylus gongrorhynchatus* | AF080341 | AF080343 | -- | -- | -- | -- | -- | -- | -- | -- | -- | -- |
| *Acanthodactylus longipes* | AF197489 | AF197490 | -- | -- | -- | -- | -- | -- | -- | -- | -- | -- |
| *Acanthodactylus maculatus* | EU086871 | EU086895 | -- | -- | -- | -- | -- | -- | -- | -- | -- | -- |
| *Acanthodactylus masirae* | AF197503 | AF197504 | -- | -- | -- | -- | -- | -- | -- | -- | -- | -- |
| *Acanthodactylus opheodurus* | AF197501 | AF197502 | -- | -- | -- | -- | -- | -- | -- | -- | -- | -- |
| *Acanthodactylus orientalis* | AF197491 | AF197492 | -- | -- | -- | -- | -- | -- | -- | -- | -- | -- |
| *Acanthodactylus pardalis* | EU086877 | EU086905 | -- | -- | -- | -- | -- | -- | -- | -- | -- | -- |
| *Acanthodactylus schmidti* | AF080375 | AF080377 | -- | -- | -- | -- | -- | -- | -- | -- | -- | -- |
| *Acanthodactylus schreiberi* | GU433283 | GU433292 | -- | -- | -- | -- | -- | -- | -- | -- | -- | -- |
| *Acanthodactylus scutellatus* | GU225707 | AF197488 | -- | EF632252 | -- | -- | -- | -- | -- | -- | EF632207 | -- |
| *Acanthodactylus tristrami* | AF197493 | AF197494 | -- | -- | -- | -- | -- | -- | -- | -- | -- | -- |
| *Acanthophis antarcticus* | -- | -- | -- | -- | AY340134 | -- | AY340162 | -- | -- | -- | -- | -- |
| *Acanthophis praelongus* | EU547112 | EU547161 | -- | EU546926 | AY340135 | -- | AY340164 | -- | -- | -- | EU546887 | -- |
| *Acanthosaura armata* | NC_014175 | NC_014175 | -- | -- | AY572872 | NC_014175 | NC_014175 | -- | -- | -- | -- | -- |
| *Acanthosaura capra* | -- | -- | -- | -- | AY572879 | AF128498 | -- | -- | -- | -- | -- | -- |
| *Acanthosaura crucigera* | AB031963 | AB031980 | -- | -- | AY572893 | -- | -- | -- | -- | -- | -- | -- |
| *Acanthosaura lepidogaster* | -- | -- | JF806003 | -- | AY572923 | AF128499 | -- | JF804531 | -- | JF804578 | JF806187 | -- |
| *Achalinus meiguensis* | FJ424614 | NC_11576 | -- | -- | FJ424614 | FJ424614 | FJ424614 | -- | -- | -- | -- | -- |
| *Achalinus rufescens* | -- | -- | -- | -- | -- | -- | U49319 | -- | -- | -- | -- | -- |
| *Acontias breviceps* | DQ249017 | HQ180036 | -- | DQ249062 | DQ249094 | -- | -- | -- | -- | -- | DQ249126 | -- |
| *Acontias gracilicauda* | AY683643 | HQ180035 | -- | DQ249049 | AY683790 | -- | -- | -- | -- | -- | DQ249113 | -- |
| *Acontias meleagris* | AY169563 | HQ180038 | GU457870 | AY662572 | FJ972224 | AY662553 | AY169637 | GU456026 | -- | HQ876348 | AY662639 | -- |
| *Acontias percivali* | AY217997 | HQ180034 | -- | AY217844 | AY217792 | -- | -- | -- | -- | -- | DQ249127 | -- |
| *Acontias plumbeus* | DQ249006 | HQ180041 | -- | DQ249040 | DQ249085 | -- | -- | -- | -- | -- | DQ249105 | -- |
| *Acontias poecilus* | -- | HQ180042 | -- | -- | -- | -- | -- | -- | -- | -- | -- | -- |
| *Acontophiops lineatus* | DQ249005 | AY649142 | -- | DQ249039 | DQ249084 | -- | -- | -- | -- | -- | DQ249104 | -- |
| *Acrantophis dumerili* | EU403569 | AF215276 | AY988032 | EU403581 | EU403574 | -- | -- | -- | -- | -- | AY988066 | -- |
| *Acrantophis madagascariensis* | EU403566 | AY336071 | FJ433973 | EU403578 | U69736 | -- | -- | -- | -- | FJ433927 | AY487401 | FJ433903 |
| *Acrochordus arafurae* | -- | -- | -- | HM234059 | -- | -- | HM234056 | -- | -- | -- | HM234062 | -- |
| *Acrochordus granulatus* | AB177879 | AB177879 | FJ433981 | AF471124 | AF217841 | AB177879 | U49296 | EU390905 | -- | EF144065 | HM234060 | EF144093 |
| *Acrochordus javanicus* | AF512745 | Z46502 | AY988036 | HM234058 | -- | -- | HM234055 | -- | -- | -- | HM234061 | -- |
| *Acutotyphlops kunuaensis* | -- | -- | GU902419 | -- | -- | -- | -- | GU902590 | -- | -- | GU902669 | -- |
| *Acutotyphlops subocularis* | -- | -- | GU902418 | -- | -- | -- | -- | GU902589 | -- | -- | GU902668 | -- |
| *Adelophis foxi* | -- | -- | -- | -- | AF420069 | AF420071 | AF420072 | -- | -- | -- | -- | -- |
| *Adelphicos quadrivirgatus* | -- | -- | -- | GQ895796 | GQ895853 | -- | -- | -- | -- | -- | -- | -- |
| *Adolfus africanus* | AF080385 | AF080386 | -- | AY234226 | HQ605870 | -- | -- | -- | -- | -- | HQ616576 | -- |
| *Adolfus alleni* | -- | HQ605779 | -- | HQ605882 | HQ605840 | -- | -- | -- | -- | -- | -- | -- |
| *Adolfus jacksoni* | AF206615 | AF206615 | -- | EF632253 | AF206539 | -- | -- | -- | -- | -- | EF632208 | -- |
| *Adolfus vauereselli* | AF206614 | AF206614 | -- | HQ616591 | AF206548 | -- | -- | -- | -- | -- | HQ616571 | -- |
| *Aeluroscalabotes felinus* | -- | -- | HQ876229 | HQ426517 | -- | AB308463 | -- | -- | HQ426171 | HQ876369 | JN654855 | HQ426432 |
| *Afroablepharus africanus* | EU164439 | AY308286 | -- | EU164501 | EU164518 | DQ675257 | -- | -- | -- | -- | DQ675337 | -- |
| *Afroablepharus annobonensis* | EU164454 | EU164494 | -- | EU164498 | EU164530 | -- | -- | -- | -- | -- | -- | -- |
| *Afroablepharus wahlbergi* | AY308327 | AY308178 | -- | -- | -- | -- | -- | -- | -- | -- | -- | -- |
| *Afroedura karroica* | -- | -- | -- | JQ945523 | -- | JX041302 | -- | -- | JQ945345 | -- | JQ945277 | JQ945415 |
| *Afroedura pondolia* | -- | -- | -- | -- | -- | EU054231 | EU054159 | -- | -- | -- | EU054207 | -- |
| *Afrogecko porphyreus* | DQ852696 | -- | JQ073120 | AY172919 | EF490750 | EF490776 | -- | -- | EF490697 | -- | EF490723 | JQ945418 |
| *Afrogecko swartbergensis* | -- | -- | -- | JQ945527 | -- | JX041305 | -- | -- | JQ945348 | -- | JQ945280 | JQ945419 |
| *Afronatrix anoscopus* | -- | -- | EU402622 | AF471123 | AF420073 | -- | -- | EU390906 | -- | JN703082 | EU402832 | -- |
| *Agama aculeata* | -- | AF355520 | -- | -- | AF355564 | -- | GU128467 | -- | -- | -- | -- | -- |
| *Agama agama* | -- | GU133310 | EU402615 | AF137530 | -- | AF128504 | AF443225 | EU390899 | -- | HQ876321 | EU402825 | -- |
| *Agama anchietae* | -- | AF355524 | -- | -- | AF355566 | GQ242169 | GU128483 | -- | -- | -- | GQ242239 | -- |
| *Agama armata* | -- | GU128447 | -- | -- | -- | -- | GU128484 | -- | -- | -- | -- | -- |
| *Agama atra* | -- | AF355515 | -- | -- | AF355557 | AF128505 | GU128485 | -- | -- | -- | -- | -- |
| *Agama boueti* | -- | GU133313 | -- | -- | -- | -- | -- | -- | -- | -- | -- | -- |
| *Agama boulengeri* | -- | GU133324 | -- | -- | -- | -- | GU128486 | -- | -- | -- | -- | -- |
| *Agama castroviejoi* | -- | AY522929 | -- | -- | -- | -- | -- | -- | -- | -- | -- | -- |
| *Agama caudospinosa* | -- | GU128450 | -- | -- | -- | -- | GU128487 | -- | -- | -- | -- | -- |
| *Agama doriae* | -- | GU128451 | -- | -- | -- | -- | GU128488 | -- | -- | -- | -- | -- |
| *Agama finchi* | -- | GU133314 | -- | -- | -- | -- | GU128489 | -- | -- | -- | -- | -- |
| *Agama gracilimembris* | -- | JF520721 | -- | -- | -- | -- | -- | -- | -- | -- | -- | -- |
| *Agama hispida* | -- | GU128453 | -- | -- | -- | -- | GU128490 | -- | -- | -- | -- | -- |
| *Agama impalearis* | -- | GU128454 | -- | -- | AB271239 | AB201526 | GU128491 | -- | -- | -- | -- | -- |
| *Agama insularis* | -- | GU133326 | -- | -- | -- | -- | -- | -- | -- | -- | -- | -- |
| *Agama kaimosae* | -- | GU128455 | -- | -- | -- | -- | GU128492 | -- | -- | -- | -- | -- |
| *Agama lionotus* | -- | GU128456 | -- | -- | -- | GQ242168 | GU128493 | -- | -- | -- | GQ242234 | -- |
| *Agama mwanzae* | -- | GU128457 | -- | -- | -- | -- | -- | -- | -- | -- | -- | -- |
| *Agama paragama* | -- | GU133321 | -- | -- | -- | -- | -- | -- | -- | -- | -- | -- |
| *Agama planiceps* | -- | AF355476 | -- | -- | AF355525 | -- | GU128494 | -- | -- | -- | -- | -- |
| *Agama rueppelli* | -- | GU128459 | -- | -- | -- | -- | GU128495 | -- | -- | -- | -- | -- |
| *Agama sankaranica* | -- | GU133327 | -- | -- | -- | -- | GU128496 | -- | -- | -- | -- | -- |
| *Agama spinosa* | -- | GU128461 | -- | -- | -- | -- | GU128497 | -- | -- | -- | -- | -- |
| *Agama weidholzi* | -- | GU133328 | -- | -- | -- | -- | GU128498 | -- | -- | -- | -- | -- |
| *Agamura persica* | DQ852726 | -- | -- | DQ852728 | EU589176 | JX041306 | -- | -- | JQ945349 | -- | JQ945281 | JQ945420 |
| *Agkistrodon bilineatus* | AF057230 | AF057277 | -- | -- | AY223613 | -- | AF156583 | -- | -- | -- | -- | -- |
| *Agkistrodon contortrix* | AF057229 | AF156566 | EU402623 | -- | U96022 | -- | U41868 | JN703027 | -- | JN703068 | EU402833 | -- |
| *Agkistrodon piscivorus* | NC_009768 | AF057278 | JQ599004 | AF471096 | AF471074 | EF669477 | U41870 | -- | -- | -- | -- | -- |
| *Agkistrodon taylori* | AF156590 | AF156569 | -- | -- | AY223614 | -- | AF156580 | -- | -- | -- | -- | -- |
| *Ahaetulla fronticincta* | -- | -- | -- | AF471161 | AF471072 | -- | -- | -- | -- | -- | -- | -- |
| *Ahaetulla nasuta* | PEA | -- | -- | KC347377 | KC347453 | -- | KC347491 | -- | -- | -- | KC347415 | -- |
| *Ahaetulla pulverulenta* | KC347304 | KC347339 | -- | KC347378 | KC347454 | -- | KC347492 | -- | -- | -- | KC347416 | -- |
| *Ailuronyx seychellensis* | AY221267 | -- | -- | AY221331 | FJ830052 | -- | -- | -- | -- | -- | -- | -- |
| *Ailuronyx tachyscopaeus* | -- | -- | -- | JQ945529 | -- | JX041307 | -- | -- | JQ945350 | -- | JQ945282 | JQ945421 |
| *Ailuronyx trachygaster* | -- | -- | -- | JQ945530 | -- | JX041308 | -- | -- | JQ945351 | -- | JQ945283 | JQ945422 |
| *Aipysurus apraefrontalis* | -- | DQ233981 | -- | -- | DQ233905 | -- | FJ593194 | -- | -- | -- | -- | -- |
| *Aipysurus duboisii* | -- | DQ233983 | -- | -- | DQ233907 | -- | EF506632 | -- | -- | -- | -- | -- |
| *Aipysurus eydouxii* | -- | DQ233986 | -- | -- | DQ233910 | -- | EF506636 | -- | -- | -- | -- | -- |
| *Aipysurus fuscus* | -- | DQ233987 | -- | -- | DQ233912 | -- | EF506634 | -- | -- | -- | -- | -- |
| *Aipysurus laevis* | EU547132 | DQ233997 | -- | -- | DQ233922 | -- | EF506638 | -- | -- | -- | EU546906 | -- |
| *Algyroides fitzingeri* | AF206598 | GQ142104 | -- | GQ142147 | GQ142134 | -- | FR821088 | -- | -- | -- | GQ142157 | -- |
| *Algyroides marchi* | GQ142080 | GQ142103 | -- | GQ142146 | GQ142133 | -- | -- | -- | -- | -- | GQ142156 | -- |
| *Algyroides moreoticus* | GQ142079 | GQ142102 | -- | EF632254 | GQ142131 | -- | -- | -- | -- | -- | EF632209 | -- |
| *Algyroides nigropunctatus* | AY942782 | AY942797 | -- | EF632255 | GQ142132 | -- | -- | -- | -- | -- | EF632210 | -- |
| *Alluaudina bellyi* | FJ404142 | AY188044 | -- | AY187966 | AY188005 | -- | FJ404345 | -- | -- | -- | -- | FJ404417 |
| *Alopoglossus angulatus* | -- | -- | HQ876226 | AF420847 | -- | -- | AF420909 | JN568329 | -- | HQ876345 | HQ876442 | -- |
| *Alopoglossus atriventris* | AF420695 | AF420746 | -- | AF420821 | -- | -- | AF420908 | -- | -- | -- | -- | -- |
| *Alopoglossus copii* | AF420692 | AF420745 | -- | AF420819 | -- | -- | AF420865 | -- | -- | -- | -- | -- |
| *Alsophis anomalus* | FJ666091 | FJ666092 | -- | -- | -- | -- | -- | -- | -- | -- | -- | -- |
| *Alsophis antiguae* | AF158455 | AF158524 | -- | -- | FJ416731 | FJ416769 | FJ416805 | -- | -- | -- | -- | FJ416842 |
| *Alsophis antillensis* | AF158459 | FJ416702 | JQ599005 | -- | FJ416726 | FJ416764 | FJ416800 | -- | -- | -- | -- | FJ416837 |
| *Alsophis biserialis* | JQ598832 | JQ598892 | -- | JQ598994 | JQ598946 | -- | -- | -- | -- | -- | -- | -- |
| *Alsophis cantherigerus* | AF158405 | AF158475 | FJ433999 | AF544694 | AF544669 | FJ416782 | FJ416818 | FJ434100 | -- | EF144082 | AY487376 | EF144109 |
| *Alsophis elegans* | AF158401 | AF158470 | JQ599042 | JQ598995 | JQ598947 | -- | -- | -- | -- | -- | -- | -- |
| *Alsophis portoricensis* | FJ416696 | AF158517 | JQ599012 | AF471126 | AF471085 | FJ416770 | U49308 | -- | -- | -- | -- | FJ416843 |
| *Alsophis rijgersmaei* | FJ416697 | FJ416708 | -- | -- | FJ416729 | FJ416767 | FJ416803 | -- | -- | -- | -- | FJ416840 |
| *Alsophis rufiventris* | FJ416698 | FJ416709 | -- | -- | FJ416730 | FJ416768 | FJ416804 | -- | -- | -- | -- | FJ416841 |
| *Alsophis vudii* | AF158443 | AF158512 | -- | JQ598976 | FJ416744 | FJ416783 | FJ416819 | -- | -- | -- | -- | FJ416855 |
| *Alsophylax pipiens* | -- | -- | -- | JQ945531 | -- | JX041309 | -- | -- | JQ945352 | -- | JQ945284 | JQ945423 |
| *Amastridium veliferum* | -- | -- | -- | GQ895797 | GQ334479 | -- | GQ334580 | GQ334663 | -- | -- | -- | -- |
| *Amblyodipsas dimidiata* | -- | -- | -- | DQ486170 | DQ486346 | -- | DQ486322 | -- | -- | -- | -- | -- |
| *Amblyodipsas polylepis* | FJ404128 | AY611844 | -- | FJ404233 | AY612027 | -- | -- | -- | -- | -- | -- | FJ404403 |
| *Amblyrhynchus cristatus* | -- | -- | -- | -- | FJ536151 | -- | U66234 | -- | -- | -- | -- | -- |
| *Ameiva ameiva* | AF206586 | AF206586 | -- | -- | AF151192 | -- | AF151206 | -- | -- | -- | -- | -- |
| *Ameiva auberi* | AY359474 | AY046466 | -- | -- | M65117 | -- | -- | -- | -- | -- | -- | DQ119629 |
| *Ameiva bifrontata* | AY046454 | AY046496 | -- | -- | -- | -- | -- | -- | -- | -- | -- | -- |
| *Ameiva chrysolaema* | AY046425 | AY046467 | -- | -- | -- | EU781100 | -- | -- | -- | -- | -- | -- |
| *Ameiva corax* | AY359477 | AY359497 | -- | -- | -- | -- | -- | -- | -- | -- | -- | -- |
| *Ameiva dorsalis* | AY359478 | AY359498 | -- | -- | -- | -- | -- | -- | -- | -- | -- | -- |
| *Ameiva erythrocephala* | AY359479 | AY359499 | -- | -- | -- | -- | -- | -- | -- | -- | -- | -- |
| *Ameiva exsul* | AY359480 | AY359500 | -- | -- | -- | -- | -- | -- | -- | -- | -- | -- |
| *Ameiva festiva* | AY359481 | AY359501 | -- | -- | -- | -- | -- | -- | -- | -- | -- | -- |
| *Ameiva fuscata* | AY359482 | AY359502 | -- | -- | -- | -- | -- | -- | -- | -- | -- | -- |
| *Ameiva griswoldi* | AY359483 | AY359503 | -- | -- | -- | -- | -- | -- | -- | -- | -- | -- |
| *Ameiva leberi* | AY292302 | AY292343 | -- | -- | -- | AY561663 | -- | -- | -- | -- | -- | -- |
| *Ameiva lineolata* | AY292296 | AY292333 | -- | -- | -- | AY561638 | -- | -- | -- | -- | -- | -- |
| *Ameiva maynardi* | AY359486 | AY359506 | -- | -- | -- | -- | -- | -- | -- | -- | -- | -- |
| *Ameiva plei* | AY359487 | AY359507 | -- | -- | -- | -- | -- | -- | -- | -- | -- | -- |
| *Ameiva pluvianotata* | AY359488 | AY359508 | -- | -- | -- | -- | -- | -- | -- | -- | -- | -- |
| *Ameiva polops* | -- | -- | -- | -- | -- | JQ240643 | -- | -- | -- | -- | -- | -- |
| *Ameiva quadrilineata* | AY046426 | AY046468 | -- | -- | -- | -- | -- | -- | -- | -- | -- | -- |
| *Ameiva taeniura* | AY359489 | AY359509 | -- | -- | -- | -- | -- | -- | -- | -- | -- | -- |
| *Ameiva undulata* | AY359491 | AY359511 | -- | JN090144 | -- | -- | -- | -- | -- | -- | -- | -- |
| *Ameiva wetmorei* | AY359492 | AY359512 | -- | -- | -- | -- | -- | -- | -- | -- | -- | -- |
| *Amphibolurus muricatus* | -- | -- | DQ340701 | AF137523 | -- | AF128468 | -- | -- | -- | -- | HQ662519 | -- |
| *Amphibolurus nobbi* | -- | -- | DQ340702 | DQ340661 | -- | AY132999 | AY281164 | -- | -- | -- | -- | -- |
| *Amphibolurus norrisi* | -- | -- | -- | -- | -- | AY133001 | -- | -- | -- | -- | HQ662434 | -- |
| *Amphiesma craspedogaster* | -- | -- | -- | JQ687437 | JQ687429 | JQ687459 | JQ687412 | -- | -- | -- | -- | -- |
| *Amphiesma sauteri* | AF402622 | -- | -- | -- | AF402905 | AF384824 | -- | -- | -- | -- | -- | -- |
| *Amphiesma stolatum* | -- | -- | EU402624 | JQ687450 | JQ687432 | JQ687464 | JQ687425 | -- | -- | JN703076 | -- | -- |
| *Amphiglossus anosyensis* | FJ667609 | FJ667621 | FJ667634 | FJ667663 | -- | -- | -- | -- | FJ667692 | -- | -- | FJ667721 |
| *Amphiglossus astrolabi* | AY391125 | AY391143 | FJ667635 | FJ667664 | -- | -- | -- | -- | FJ667693 | -- | -- | FJ667722 |
| *Amphiglossus frontoparietalis* | FJ667610 | FJ667622 | FJ667636 | FJ667665 | -- | -- | -- | -- | FJ667694 | -- | -- | FJ667723 |
| *Amphiglossus macrocercus* | AY315480 | AY391146 | FJ667637 | FJ667666 | AY391164 | -- | -- | -- | FJ667695 | -- | JQ073195 | FJ667724 |
| *Amphiglossus mandokava* | FJ667611 | FJ667623 | FJ667638 | FJ667667 | AY391165 | -- | -- | -- | FJ667696 | -- | -- | FJ667725 |
| *Amphiglossus melanurus* | AY315502 | AY391148 | FJ667639 | FJ667668 | AY391166 | -- | -- | -- | FJ667697 | -- | -- | FJ667726 |
| *Amphiglossus nanus* | AY315493 | AY315542 | -- | -- | -- | -- | -- | -- | -- | -- | -- | -- |
| *Amphiglossus ornaticeps* | AY315488 | AY391150 | -- | AY391186 | AY391168 | -- | -- | -- | -- | -- | -- | -- |
| *Amphiglossus polleni* | -- | -- | FJ667651 | FJ667680 | -- | -- | -- | -- | FJ667709 | -- | -- | -- |
| *Amphiglossus punctatus* | AY391133 | FJ667624 | FJ667640 | FJ667669 | AY391169 | -- | -- | -- | FJ667698 | -- | -- | FJ667727 |
| *Amphiglossus reticulatus* | AY315490 | AY315539 | FJ667641 | FJ667670 | AY391172 | -- | -- | -- | FJ667699 | -- | -- | FJ667728 |
| *Amphiglossus splendidus* | AY315495 | AY315544 | JN614211 | -- | -- | -- | -- | JN568316 | -- | JN568489 | -- | -- |
| *Amphiglossus tanysoma* | AY315498 | AY315547 | FJ667642 | FJ667671 | -- | -- | -- | -- | FJ667700 | -- | -- | FJ667729 |
| *Amphiglossus tsaratananensis* | AY315499 | AY391153 | -- | AY391189 | AY391171 | -- | -- | -- | -- | -- | -- | -- |
| *Amphisbaena alba* | EU203644 | FJ441706 | FJ441888 | FJ441766 | -- | FJ441948 | -- | -- | HQ426249 | -- | FJ441826 | -- |
| *Amphisbaena anaemariae* | -- | FJ441668 | FJ441851 | FJ441728 | -- | FJ441911 | -- | -- | -- | -- | FJ441788 | -- |
| *Amphisbaena angustifrons* | -- | FJ441707 | FJ441890 | FJ441767 | -- | FJ441950 | -- | -- | -- | -- | FJ441827 | -- |
| *Amphisbaena bakeri* | EU203645 | EU203645 | -- | -- | -- | -- | -- | -- | -- | -- | -- | -- |
| *Amphisbaena barbouri* | EU203646 | EU203646 | -- | -- | -- | -- | -- | -- | -- | -- | -- | -- |
| *Amphisbaena bolivica* | -- | FJ441670 | FJ441853 | FJ441730 | -- | FJ441913 | -- | -- | -- | -- | FJ441790 | -- |
| *Amphisbaena caeca* | EU203647 | FJ441671 | FJ441854 | FJ441731 | -- | FJ441914 | -- | -- | -- | -- | FJ441791 | -- |
| *Amphisbaena camura* | -- | FJ441672 | FJ441855 | FJ441732 | -- | FJ441915 | -- | -- | -- | -- | FJ441792 | -- |
| *Amphisbaena carlgansi* | EU203648 | EU203648 | -- | -- | -- | -- | -- | -- | -- | -- | -- | -- |
| *Amphisbaena cubana* | EU203649 | EU203649 | EU203609 | AY487346 | -- | -- | -- | EU203626 | -- | DQ119615 | AY487365 | DQ119633 |
| *Amphisbaena cunhai* | -- | FJ441673 | FJ441856 | FJ441733 | -- | FJ441916 | -- | -- | -- | -- | FJ441793 | -- |
| *Amphisbaena darwini* | -- | FJ441693 | FJ441876 | FJ441753 | -- | FJ441936 | -- | -- | -- | -- | FJ441813 | -- |
| *Amphisbaena fenestrata* | EU203650 | EU203650 | -- | -- | -- | -- | -- | -- | -- | -- | -- | -- |
| *Amphisbaena fuliginosa* | -- | FJ441684 | FJ441865 | FJ441744 | -- | FJ441927 | -- | GU456032 | -- | HQ876352 | FJ441804 | -- |
| *Amphisbaena hastata* | -- | FJ441678 | FJ441860 | FJ441738 | -- | FJ441921 | -- | -- | -- | -- | FJ441798 | -- |
| *Amphisbaena hyporissor* | EU203651 | EU203651 | -- | -- | -- | -- | -- | -- | -- | -- | -- | -- |
| *Amphisbaena ignatiana* | -- | FJ441680 | FJ441863 | FJ441740 | -- | FJ441922 | -- | -- | -- | -- | FJ441800 | -- |
| *Amphisbaena innocens* | EU203652 | EU203652 | -- | -- | -- | -- | -- | -- | -- | -- | -- | -- |
| *Amphisbaena leali* | EU203653 | EU203653 | -- | -- | -- | -- | -- | -- | -- | -- | -- | -- |
| *Amphisbaena leeseri* | -- | FJ441694 | FJ441877 | FJ441754 | -- | FJ441937 | -- | -- | -- | -- | FJ441814 | -- |
| *Amphisbaena manni* | EU203654 | EU203654 | -- | -- | -- | -- | -- | -- | -- | -- | -- | -- |
| *Amphisbaena mertensii* | -- | FJ441676 | FJ441858 | FJ441736 | -- | FJ441918 | -- | -- | -- | -- | FJ441796 | -- |
| *Amphisbaena munoai* | -- | FJ441687 | FJ441870 | FJ441747 | -- | FJ441930 | -- | -- | -- | -- | FJ441807 | -- |
| *Amphisbaena schmidti* | EU203655 | NC_006284 | FJ441864 | FJ441741 | NC_006284 | FJ441924 | NC_006284 | -- | -- | -- | FJ441801 | -- |
| *Amphisbaena silvestrii* | -- | FJ441689 | FJ441871 | FJ441749 | -- | FJ441932 | -- | -- | -- | -- | FJ441809 | -- |
| *Amphisbaena vermicularis* | -- | FJ441686 | FJ441869 | FJ441746 | -- | FJ441929 | -- | -- | -- | -- | FJ441806 | -- |
| *Amphisbaena xera* | EU203656 | EU203656 | -- | AY662568 | -- | AY662541 | -- | -- | -- | -- | AY662619 | -- |
| *Amplorhinus multimaculatus* | FJ404143 | AY611880 | -- | DQ486176 | DQ486352 | -- | FJ404346 | -- | -- | -- | -- | FJ404418 |
| *Anatololacerta anatolica* | GQ142086 | GQ142109 | -- | GQ142150 | GQ142138 | -- | -- | -- | -- | -- | -- | -- |
| *Anatololacerta danfordi* | AJ238188 | GQ142107 | -- | DQ461743 | DQ461765 | -- | -- | -- | -- | -- | EF632224 | -- |
| *Anatololacerta oertzeni* | GQ142085 | GQ142108 | -- | GQ142149 | GQ142137 | -- | -- | -- | -- | -- | GQ142159 | -- |
| *Androngo trivittatus* | FJ667615 | AY151444 | FJ667646 | FJ667675 | AF280126 | -- | -- | -- | FJ667704 | -- | -- | FJ667733 |
| *Anelytropsis papillosus* | -- | DQ990972 | GU457862 | HQ876394 | -- | HQ876244 | -- | GU456018 | -- | HQ876383 | GU457985 | -- |
| *Anguis fragilis* | EU443256 | EU443256 | -- | AY099972 | AY099996 | AF085622 | NC_012431 | JQ844948 | -- | -- | -- | -- |
| *Anilius scytale* | AF512729 | NC_14343 | EU402625 | AF544722 | U69738 | FJ755180 | FJ755180 | FJ434066 | -- | HQ876355 | AY988072 | FJ433891 |
| *Anisolepis longicauda* | AF338336 | -- | -- | -- | -- | AF528736 | -- | -- | -- | -- | -- | -- |
| *Anniella geronimensis* | -- | -- | EU445907 | -- | AF195091 | AF085605 | -- | -- | -- | -- | -- | -- |
| *Anniella pulchra* | -- | -- | EU445901 | AY487350 | AF195090 | AF407537 | AY620747 | GU456008 | -- | HM161053 | AY662605 | DQ119636 |
| *Anolis acutus* | AY534654 | -- | EU544058 | -- | AY534670 | AF055926 | -- | EU544098 | -- | EU544118 | EU544034 | -- |
| *Anolis aeneus* | -- | -- | -- | -- | EU557103 | AF055950 | -- | -- | -- | -- | JN112592 | -- |
| *Anolis aequatorialis* | -- | -- | -- | -- | -- | JN112662 | -- | -- | -- | -- | JN112593 | -- |
| *Anolis agassizi* | -- | -- | -- | -- | -- | AF055952 | -- | -- | -- | -- | JN112595 | -- |
| *Anolis ahli* | -- | -- | -- | -- | -- | AY296148 | -- | -- | -- | -- | -- | -- |
| *Anolis alayoni* | -- | -- | -- | -- | -- | AY296149 | -- | -- | -- | -- | -- | -- |
| *Anolis alfaroi* | -- | -- | -- | -- | -- | AY296150 | -- | -- | -- | -- | -- | -- |
| *Anolis aliniger* | -- | -- | -- | -- | -- | EF531488 | -- | -- | -- | -- | -- | -- |
| *Anolis allisoni* | -- | -- | -- | -- | -- | AY296151 | -- | -- | -- | -- | -- | -- |
| *Anolis allogus* | -- | -- | -- | -- | -- | AY296152 | -- | -- | -- | -- | -- | -- |
| *Anolis altae* | -- | -- | -- | -- | -- | AY909735 | -- | -- | -- | -- | -- | -- |
| *Anolis alumina* | -- | -- | -- | -- | -- | AY296153 | -- | -- | -- | -- | -- | -- |
| *Anolis alutaceus* | -- | Z48641 | -- | -- | -- | AF055971 | -- | -- | -- | -- | -- | -- |
| *Anolis anatoloros* | -- | -- | -- | -- | -- | JN112668 | -- | -- | -- | -- | JN112596 | -- |
| *Anolis angusticeps* | -- | -- | -- | -- | -- | AF055967 | -- | -- | -- | -- | -- | -- |
| *Anolis annectens* | -- | -- | -- | -- | -- | AY909736 | -- | -- | -- | -- | -- | -- |
| *Anolis aquaticus* | -- | -- | -- | -- | -- | AY909738 | -- | -- | -- | -- | -- | -- |
| *Anolis argenteolus* | -- | -- | -- | -- | -- | AY296154 | -- | -- | -- | -- | -- | -- |
| *Anolis argillaceus* | -- | Z48642 | -- | -- | -- | AY909739 | -- | -- | -- | -- | -- | -- |
| *Anolis armouri* | -- | -- | -- | -- | -- | AY263014 | -- | -- | -- | -- | -- | -- |
| *Anolis auratus* | -- | -- | -- | -- | -- | AY909740 | -- | -- | -- | -- | -- | -- |
| *Anolis bahorucoensis* | -- | -- | -- | -- | -- | AF055932 | -- | -- | -- | -- | -- | -- |
| *Anolis baleatus* | -- | -- | -- | -- | -- | AY296155 | -- | -- | -- | -- | -- | -- |
| *Anolis baracoae* | -- | -- | -- | -- | -- | AY296156 | -- | -- | -- | -- | -- | -- |
| *Anolis barahonae* | -- | -- | -- | -- | -- | AF055972 | -- | -- | -- | -- | -- | -- |
| *Anolis barbatus* | -- | -- | -- | -- | -- | AY296146 | -- | -- | -- | -- | -- | -- |
| *Anolis barbouri* | -- | Z48643 | -- | -- | -- | AF055946 | -- | -- | -- | -- | -- | -- |
| *Anolis bartschi* | -- | -- | -- | -- | -- | AF055960 | -- | -- | -- | -- | -- | -- |
| *Anolis bicaorum* | -- | -- | -- | -- | -- | AY909741 | -- | -- | -- | -- | -- | -- |
| *Anolis bimaculatus* | -- | Z48644 | -- | -- | AF212123 | AF055930 | -- | -- | -- | -- | JN112650 | -- |
| *Anolis biporcatus* | -- | -- | -- | -- | -- | AF294286 | -- | -- | -- | -- | -- | -- |
| *Anolis bitectus* | -- | -- | -- | -- | -- | AY909743 | -- | -- | -- | -- | -- | -- |
| *Anolis boettgeri* | -- | Z48645 | -- | -- | -- | -- | -- | -- | -- | -- | -- | -- |
| *Anolis bombiceps* | -- | -- | -- | -- | -- | JN191570 | -- | -- | -- | -- | -- | -- |
| *Anolis bonairensis* | -- | -- | -- | -- | -- | AF317070 | -- | -- | -- | -- | -- | -- |
| *Anolis bremeri* | -- | -- | -- | -- | -- | AY296157 | -- | -- | -- | -- | -- | -- |
| *Anolis brevirostris* | AY534650 | -- | -- | -- | AY534667 | AY296158 | -- | -- | -- | -- | -- | -- |
| *Anolis brunneus* | -- | -- | -- | -- | -- | AY296159 | -- | -- | -- | -- | -- | -- |
| *Anolis calimae* | -- | -- | -- | -- | -- | JN112670 | -- | -- | -- | -- | JN112597 | -- |
| *Anolis capito* | -- | -- | -- | -- | -- | AY909744 | -- | -- | -- | -- | -- | -- |
| *Anolis carolinensis* | NC_010972 | AB031978 | EU402616 | -- | NC_010972 | AB473620 | EU747728 | EU390900 | XM_003221561 | HQ876334 | FJ356739 | XM_003214648 |
| *Anolis carpenteri* | -- | -- | -- | -- | -- | AY296160 | -- | -- | -- | -- | -- | -- |
| *Anolis casildae* | -- | -- | -- | -- | -- | AY909745 | -- | -- | -- | -- | JN112599 | -- |
| *Anolis caudalis* | -- | -- | -- | -- | -- | AY296161 | -- | -- | -- | -- | -- | -- |
| *Anolis centralis* | -- | -- | -- | -- | -- | AY296162 | -- | -- | -- | -- | -- | -- |
| *Anolis chamaeleonides* | -- | Z48646 | -- | -- | -- | AF055975 | -- | -- | -- | -- | -- | -- |
| *Anolis chloris* | -- | -- | -- | -- | -- | JN112672 | -- | -- | -- | -- | JN112601 | -- |
| *Anolis chlorocyanus* | -- | -- | -- | -- | -- | AY296163 | -- | -- | -- | -- | -- | -- |
| *Anolis chocorum* | -- | -- | -- | -- | -- | JN112674 | -- | -- | -- | -- | JN112603 | -- |
| *Anolis christophei* | -- | Z48647 | -- | -- | -- | AF055957 | -- | -- | -- | -- | -- | -- |
| *Anolis chrysolepis* | -- | -- | -- | -- | -- | AF294281 | -- | -- | -- | -- | -- | -- |
| *Anolis clivicola* | -- | -- | -- | -- | -- | AY909746 | -- | -- | -- | -- | -- | -- |
| *Anolis coelestinus* | -- | -- | -- | -- | -- | AY296164 | -- | -- | -- | -- | -- | -- |
| *Anolis confusus* | -- | -- | -- | -- | -- | AY909787 | -- | -- | -- | -- | -- | -- |
| *Anolis conspersus* | -- | -- | -- | -- | -- | AF294304 | -- | -- | -- | -- | -- | -- |
| *Anolis cooki* | AY662313 | -- | EU544066 | -- | EU095689 | AY296165 | -- | EU544104 | -- | EU544126 | EU544042 | -- |
| *Anolis crassulus* | -- | -- | -- | -- | -- | AY909748 | -- | -- | -- | -- | -- | -- |
| *Anolis cristatellus* | AY534658 | Z48648 | EU544071 | -- | EF553583 | AY296166 | -- | EU544114 | -- | EU544139 | EU544054 | -- |
| *Anolis cupeyalensis* | -- | -- | -- | -- | -- | AY909749 | -- | -- | -- | -- | -- | -- |
| *Anolis cupreus* | -- | -- | -- | -- | -- | AY909750 | -- | -- | -- | -- | -- | -- |
| *Anolis cuvieri* | -- | Z48649 | -- | -- | -- | AF055973 | -- | -- | -- | -- | JN112652 | -- |
| *Anolis cyanopleurus* | -- | -- | -- | -- | -- | AY909751 | -- | -- | -- | -- | -- | -- |
| *Anolis cybotes* | AB218960 | Z48650 | -- | -- | AB218960 | EU107838 | AB218960 | -- | -- | -- | -- | -- |
| *Anolis danieli* | -- | -- | -- | -- | -- | JN112676 | -- | -- | -- | -- | JN112605 | -- |
| *Anolis darlingtoni* | -- | Z48651 | -- | -- | -- | AY367137 | -- | -- | -- | -- | -- | -- |
| *Anolis desechensis* | AY662316 | -- | EU544068 | -- | EF553600 | AY296167 | -- | EU544106 | -- | EU544128 | EU544044 | -- |
| *Anolis distichus* | AY534651 | Z48652 | EU544057 | -- | -- | AY296168 | -- | EU544097 | -- | EU544117 | EU544033 | -- |
| *Anolis dolichocephalus* | -- | -- | -- | -- | -- | AY296169 | -- | -- | -- | -- | -- | -- |
| *Anolis equestris* | -- | -- | -- | -- | -- | EU107892 | -- | -- | -- | -- | JN112653 | -- |
| *Anolis ernestwilliamsi* | AY534659 | -- | EU544069 | -- | AY534675 | AY296170 | -- | EU544107 | -- | EU544129 | EU544045 | -- |
| *Anolis etheridgei* | -- | -- | -- | -- | -- | AF055934 | -- | -- | -- | -- | -- | -- |
| *Anolis eugenegrahami* | -- | -- | -- | -- | -- | AY296171 | -- | -- | -- | -- | -- | -- |
| *Anolis euskalerriari* | -- | -- | -- | -- | -- | JN112678 | -- | -- | -- | -- | JN112611 | -- |
| *Anolis evermanni* | AY534653 | -- | EU544059 | -- | AY662319 | AY296172 | -- | EU544099 | -- | EU544119 | EU544035 | -- |
| *Anolis extremus* | -- | -- | -- | -- | EU557123 | AF317065 | -- | -- | -- | -- | JN112612 | -- |
| *Anolis ferreus* | -- | -- | -- | -- | AF426908 | AY296173 | -- | -- | -- | -- | -- | -- |
| *Anolis festae* | -- | -- | -- | -- | -- | JN112680 | -- | -- | -- | -- | JN112613 | -- |
| *Anolis fitchi* | -- | -- | -- | -- | -- | JN112682 | -- | -- | -- | -- | JN112615 | -- |
| *Anolis fowleri* | -- | -- | -- | -- | -- | AY296174 | -- | -- | -- | -- | -- | -- |
| *Anolis fraseri* | -- | -- | -- | -- | -- | JN112684 | -- | -- | -- | -- | JN112617 | -- |
| *Anolis frenatus* | -- | -- | -- | -- | -- | AY909752 | -- | -- | -- | -- | JN112618 | -- |
| *Anolis fuscoauratus* | AF338337 | L41440 | -- | -- | -- | AF294284 | -- | -- | -- | -- | -- | -- |
| *Anolis garmani* | -- | -- | -- | -- | -- | EU107961 | -- | -- | -- | -- | -- | -- |
| *Anolis garridoi* | -- | -- | -- | -- | -- | AY296175 | -- | -- | -- | -- | -- | -- |
| *Anolis gemmosus* | -- | -- | -- | -- | -- | JN112686 | -- | -- | -- | -- | JN112620 | -- |
| *Anolis gingivinus* | AY534648 | -- | -- | -- | AF426893 | AY909753 | -- | -- | -- | -- | -- | -- |
| *Anolis grahami* | -- | Z48653 | -- | -- | -- | DQ353945 | -- | -- | -- | -- | -- | -- |
| *Anolis griseus* | -- | Z48654 | -- | -- | EU557102 | AY296176 | -- | -- | -- | -- | JN112622 | -- |
| *Anolis guafe* | -- | -- | -- | -- | -- | AY909788 | -- | -- | -- | -- | -- | -- |
| *Anolis guamuhaya* | -- | -- | -- | -- | -- | AF055974 | -- | -- | -- | -- | -- | -- |
| *Anolis guazuma* | -- | -- | -- | -- | -- | AY909754 | -- | -- | -- | -- | -- | -- |
| *Anolis gundlachi* | AY534662 | -- | EU544060 | -- | EU095730 | AY296177 | -- | EU544100 | -- | EU544120 | EU544036 | -- |
| *Anolis haetianus* | -- | -- | -- | -- | -- | AY263042 | -- | -- | -- | -- | -- | -- |
| *Anolis hendersoni* | -- | Z48655 | -- | -- | -- | AY296178 | -- | -- | -- | -- | -- | -- |
| *Anolis heterodermus* | -- | -- | -- | -- | -- | AY296144 | -- | -- | -- | -- | JN112624 | -- |
| *Anolis homolechis* | -- | -- | -- | -- | -- | AY296179 | -- | -- | -- | -- | -- | -- |
| *Anolis huilae* | -- | -- | -- | -- | -- | JN112692 | -- | -- | -- | -- | JN112626 | -- |
| *Anolis humilis* | -- | -- | -- | -- | -- | AF055944 | -- | -- | -- | -- | -- | -- |
| *Anolis imias* | -- | -- | -- | -- | -- | AF294314 | -- | -- | -- | -- | -- | -- |
| *Anolis inderenae* | -- | -- | -- | -- | -- | AY296145 | -- | -- | -- | -- | JN112628 | -- |
| *Anolis inexpectatus* | -- | -- | -- | -- | -- | AY296180 | -- | -- | -- | -- | -- | -- |
| *Anolis insignis* | -- | -- | -- | -- | -- | JN112693 | -- | -- | -- | -- | -- | -- |
| *Anolis insolitus* | -- | -- | -- | -- | -- | AF055933 | -- | -- | -- | -- | -- | -- |
| *Anolis intermedius* | -- | -- | -- | -- | -- | AY909755 | -- | -- | -- | -- | -- | -- |
| *Anolis isolepis* | -- | -- | -- | -- | -- | AY296181 | -- | -- | -- | -- | -- | -- |
| *Anolis isthmicus* | -- | -- | -- | -- | -- | AY909762 | -- | -- | -- | -- | -- | -- |
| *Anolis jacare* | -- | -- | -- | -- | EU557099 | JN112696 | -- | -- | -- | -- | JN112630 | -- |
| *Anolis jubar* | -- | -- | -- | -- | -- | AY296182 | -- | -- | -- | -- | -- | -- |
| *Anolis kemptoni* | -- | -- | -- | -- | -- | AY909770 | -- | -- | -- | -- | -- | -- |
| *Anolis krugi* | AY534663 | -- | EU544061 | -- | EU095731 | EU095783 | -- | EU544101 | -- | EU544121 | EU544037 | -- |
| *Anolis laeviventris* | -- | -- | -- | -- | -- | AY909756 | -- | -- | -- | -- | -- | -- |
| *Anolis leachii* | -- | -- | -- | -- | AF426896 | AY296183 | -- | -- | -- | -- | -- | -- |
| *Anolis lemurinus* | -- | -- | -- | -- | -- | AF294283 | -- | -- | -- | -- | -- | -- |
| *Anolis limifrons* | -- | Z48657 | -- | -- | -- | AF055943 | -- | -- | -- | -- | -- | -- |
| *Anolis lineatopus* | -- | -- | -- | -- | -- | AF055937 | -- | -- | -- | -- | -- | -- |
| *Anolis lineatus* | -- | -- | -- | -- | -- | AF055935 | -- | -- | -- | -- | -- | -- |
| *Anolis lionotus* | -- | -- | -- | -- | -- | AY909757 | -- | -- | -- | -- | -- | -- |
| *Anolis lividus* | -- | -- | -- | -- | AF426909 | AY909758 | -- | -- | -- | -- | -- | -- |
| *Anolis longiceps* | -- | -- | -- | -- | -- | AY296184 | -- | -- | -- | -- | -- | -- |
| *Anolis longitibialis* | -- | -- | -- | -- | -- | AY263010 | -- | -- | -- | -- | -- | -- |
| *Anolis loveridgei* | -- | -- | -- | -- | -- | AY909759 | -- | -- | -- | -- | -- | -- |
| *Anolis loysiana* | -- | -- | -- | -- | -- | AF055964 | -- | -- | -- | -- | -- | -- |
| *Anolis luciae* | -- | -- | -- | -- | EU557100 | AF055951 | -- | -- | -- | -- | JN112631 | -- |
| *Anolis lucius* | -- | Z48659 | -- | -- | -- | -- | -- | -- | -- | -- | JN112654 | -- |
| *Anolis luteogularis* | -- | -- | -- | -- | -- | AF055977 | -- | -- | -- | -- | -- | -- |
| *Anolis macilentus* | -- | -- | -- | -- | -- | AY296185 | -- | -- | -- | -- | -- | -- |
| *Anolis maculigula* | -- | -- | -- | -- | -- | JN112698 | -- | -- | -- | -- | JN112632 | -- |
| *Anolis marcanoi* | -- | -- | -- | -- | -- | AF055955 | -- | -- | -- | -- | JN112655 | -- |
| *Anolis marmoratus* | -- | -- | -- | -- | AF426906 | AY296186 | -- | -- | -- | -- | -- | -- |
| *Anolis marron* | -- | -- | -- | -- | -- | AY296187 | -- | -- | -- | -- | -- | -- |
| *Anolis maynardi* | -- | -- | -- | -- | -- | AY902411 | -- | -- | -- | -- | -- | -- |
| *Anolis meridionalis* | AF338332 | -- | -- | -- | -- | AY909760 | -- | -- | -- | -- | -- | -- |
| *Anolis mestrei* | -- | -- | -- | -- | -- | AF337779 | -- | -- | -- | -- | -- | -- |
| *Anolis microtus* | -- | -- | -- | -- | -- | AF055947 | -- | -- | -- | -- | -- | -- |
| *Anolis monensis* | AY534656 | -- | EU544067 | -- | EF553631 | AY296188 | -- | EU544105 | -- | EU544127 | EU544043 | -- |
| *Anolis monticola* | -- | -- | -- | -- | -- | AY296189 | -- | -- | -- | -- | -- | -- |
| *Anolis neblininus* | -- | -- | -- | -- | -- | JN112700 | -- | -- | -- | -- | JN112634 | -- |
| *Anolis nicefori* | -- | -- | -- | -- | -- | AF055948 | -- | -- | -- | -- | -- | -- |
| *Anolis nitens* | -- | -- | GQ853274 | GQ853276 | -- | AF337807 | -- | -- | -- | -- | -- | -- |
| *Anolis noblei* | -- | -- | -- | -- | -- | AY296190 | -- | -- | -- | -- | -- | -- |
| *Anolis nubilis* | -- | -- | -- | -- | AF426894 | AY909764 | -- | -- | -- | -- | -- | -- |
| *Anolis occultus* | -- | Z48660 | -- | -- | -- | AF055976 | -- | -- | -- | -- | JN112656 | -- |
| *Anolis ocelloscapularis* | -- | -- | -- | -- | -- | AY909767 | -- | -- | -- | -- | -- | -- |
| *Anolis oculatus* | -- | -- | -- | -- | EU557098 | AY296191 | -- | -- | -- | -- | -- | -- |
| *Anolis olssoni* | -- | -- | -- | -- | -- | AF055945 | -- | -- | -- | -- | -- | -- |
| *Anolis onca* | -- | -- | -- | -- | -- | AY909765 | -- | -- | -- | -- | -- | -- |
| *Anolis opalinus* | -- | -- | -- | -- | -- | AF294309 | -- | -- | -- | -- | -- | -- |
| *Anolis ophiolepis* | -- | Z48661 | -- | -- | -- | AF055942 | -- | -- | -- | -- | -- | -- |
| *Anolis oporinus* | -- | -- | -- | -- | -- | AY909766 | -- | -- | -- | -- | -- | -- |
| *Anolis ortonii* | U39561 | -- | -- | -- | -- | AF294288 | -- | -- | -- | -- | -- | -- |
| *Anolis oxylophus* | -- | -- | -- | -- | -- | AY909768 | -- | -- | -- | -- | -- | -- |
| *Anolis pachypus* | -- | -- | -- | -- | -- | AY909769 | -- | -- | -- | -- | -- | -- |
| *Anolis paternus* | -- | -- | -- | -- | -- | AF055965 | -- | -- | -- | -- | AY662589 | -- |
| *Anolis peraccae* | -- | -- | -- | -- | -- | JN112701 | -- | -- | -- | -- | JN112636 | -- |
| *Anolis placidus* | -- | -- | -- | -- | -- | AY296192 | -- | -- | -- | -- | -- | -- |
| *Anolis poecilopus* | -- | -- | -- | -- | -- | AY909771 | -- | -- | -- | -- | -- | -- |
| *Anolis pogus* | -- | -- | EU544056 | -- | AF212126 | AY296193 | -- | EU544096 | -- | EU544116 | EU544032 | -- |
| *Anolis polylepis* | -- | -- | -- | -- | HQ641742 | AY909772 | -- | -- | -- | -- | -- | -- |
| *Anolis polyrhachis* | -- | -- | -- | -- | -- | AY909773 | -- | -- | -- | -- | -- | -- |
| *Anolis poncensis* | AY534661 | -- | EU544062 | -- | AB377062 | AY296194 | -- | EU544102 | -- | EU544122 | EU544038 | -- |
| *Anolis porcatus* | -- | Z48668 | -- | -- | -- | AY296195 | -- | -- | -- | -- | -- | -- |
| *Anolis porcus* | -- | Z48663 | -- | -- | -- | AY296147 | -- | -- | -- | -- | -- | -- |
| *Anolis princeps* | -- | -- | -- | -- | -- | JN112705 | -- | -- | -- | -- | JN112638 | -- |
| *Anolis pulchellus* | AY534664 | -- | EU544063 | -- | EF553634 | AY296196 | -- | -- | -- | EU544123 | EU544039 | -- |
| *Anolis pumilus* | -- | -- | -- | -- | -- | AF055963 | -- | -- | -- | -- | -- | -- |
| *Anolis punctatus* | -- | -- | -- | -- | -- | AF528726 | -- | -- | -- | -- | JN112640 | -- |
| *Anolis purpurgularis* | -- | -- | -- | -- | -- | AY909774 | -- | -- | -- | -- | -- | -- |
| *Anolis quadriocellifer* | -- | -- | -- | -- | -- | AY296197 | -- | -- | -- | -- | -- | -- |
| *Anolis quercorum* | -- | -- | -- | -- | -- | AY909775 | -- | -- | -- | -- | -- | -- |
| *Anolis reconditus* | -- | -- | -- | -- | -- | AY296198 | -- | -- | -- | -- | -- | -- |
| *Anolis rejectus* | -- | -- | -- | -- | -- | AY909761 | -- | -- | -- | -- | -- | -- |
| *Anolis richardii* | -- | -- | -- | -- | EU557101 | AF055949 | -- | -- | -- | -- | JN112641 | -- |
| *Anolis ricordi* | -- | Z48664 | -- | -- | -- | AY367138 | -- | -- | -- | -- | -- | -- |
| *Anolis roquet* | AF338339 | -- | -- | -- | EU557193 | AF317063 | -- | -- | -- | -- | JN112642 | -- |
| *Anolis rubribarbaris* | -- | -- | -- | -- | -- | AY909789 | -- | -- | -- | -- | -- | -- |
| *Anolis sabanus* | -- | -- | -- | -- | AF426895 | AY909776 | -- | -- | -- | -- | -- | -- |
| *Anolis sagrei* | -- | Z48665 | -- | -- | -- | AF055940 | -- | -- | -- | -- | JN112657 | -- |
| *Anolis scriptus* | AY534660 | -- | EU544065 | -- | AY534676 | AY296200 | -- | EU544103 | -- | EU544125 | EU544041 | -- |
| *Anolis semilineatus* | -- | Z48666 | -- | -- | -- | AY296201 | -- | -- | -- | -- | -- | -- |
| *Anolis sericeus* | -- | -- | -- | -- | -- | AY909778 | -- | -- | -- | -- | -- | -- |
| *Anolis sheplani* | -- | -- | -- | -- | -- | AF055966 | -- | -- | -- | -- | -- | -- |
| *Anolis shrevei* | -- | -- | -- | -- | -- | AY263041 | -- | -- | -- | -- | -- | -- |
| *Anolis singularis* | -- | -- | -- | -- | -- | AY296202 | -- | -- | -- | -- | -- | -- |
| *Anolis smallwoodi* | -- | Z48667 | -- | -- | -- | AY296203 | -- | -- | -- | -- | -- | -- |
| *Anolis smaragdinus* | -- | -- | -- | -- | -- | AY902424 | -- | -- | -- | -- | JN112658 | -- |
| *Anolis sminthus* | -- | -- | -- | -- | -- | AY909779 | -- | -- | -- | -- | -- | -- |
| *Anolis strahmi* | -- | -- | -- | -- | -- | AF055956 | -- | -- | -- | -- | -- | -- |
| *Anolis stratulus* | AY534652 | -- | EU544064 | -- | AY662322 | AF055929 | -- | -- | -- | EU544124 | EU544040 | -- |
| *Anolis terraealtae* | -- | -- | -- | -- | AF212118 | -- | -- | -- | -- | -- | -- | -- |
| *Anolis tigrinus* | -- | -- | -- | -- | -- | JN112710 | -- | -- | -- | -- | JN112643 | -- |
| *Anolis trachyderma* | -- | -- | -- | -- | -- | AF294285 | -- | -- | -- | -- | -- | -- |
| *Anolis transversalis* | -- | -- | -- | -- | -- | AF337769 | -- | -- | -- | -- | JN112644 | -- |
| *Anolis trinitatis* | -- | -- | -- | -- | AF493603 | AY296204 | -- | -- | -- | -- | JN112645 | -- |
| *Anolis tropidogaster* | -- | -- | -- | -- | -- | AY909782 | -- | -- | -- | -- | -- | -- |
| *Anolis tropidonotus* | -- | -- | -- | -- | -- | AY909783 | -- | -- | -- | -- | -- | -- |
| *Anolis uniformis* | -- | -- | -- | -- | -- | AY909784 | -- | -- | -- | -- | -- | -- |
| *Anolis utilensis* | -- | -- | -- | -- | -- | AY909785 | -- | -- | -- | -- | -- | -- |
| *Anolis valencienni* | -- | -- | -- | -- | -- | AF055939 | -- | -- | -- | -- | -- | -- |
| *Anolis vanidicus* | -- | -- | -- | -- | -- | AF055970 | -- | -- | -- | -- | -- | -- |
| *Anolis vanzolinii* | -- | -- | -- | -- | -- | JN112712 | -- | -- | -- | -- | -- | -- |
| *Anolis ventrimaculatus* | -- | -- | -- | -- | -- | JN112713 | -- | -- | -- | -- | JN112646 | -- |
| *Anolis vermiculatus* | -- | -- | -- | -- | -- | AF055961 | -- | -- | -- | -- | -- | -- |
| *Anolis wattsi* | AY534649 | -- | -- | -- | AY100211 | AF055931 | -- | -- | -- | -- | -- | -- |
| *Anolis websteri* | -- | -- | -- | -- | -- | AY296205 | -- | -- | -- | -- | -- | -- |
| *Anolis whitemani* | -- | -- | -- | -- | -- | AY263035 | -- | -- | -- | -- | -- | -- |
| *Anolis woodi* | -- | -- | -- | -- | -- | AF337780 | -- | -- | -- | -- | -- | -- |
| *Anolis zeus* | -- | -- | -- | -- | -- | AY909786 | -- | -- | -- | -- | -- | -- |
| *Anomalopus leuckartii* | HM852474 | HM852501 | -- | -- | -- | -- | DQ915342 | -- | -- | -- | -- | -- |
| *Anomalopus mackayi* | AY169575 | AY169612 | -- | -- | -- | -- | AY169650 | -- | -- | -- | -- | -- |
| *Anomalopus swansoni* | AY169576 | AY169613 | HQ655159 | HQ655196 | -- | -- | AY169651 | -- | -- | -- | -- | -- |
| *Anomalopus verreauxi* | AY308328 | AY308179 | -- | -- | -- | -- | -- | -- | -- | -- | -- | -- |
| *Anomochilus leonardi* | AY953430 | AY953431 | -- | -- | -- | -- | -- | -- | -- | -- | -- | -- |
| *Anops kingii* | -- | FJ441726 | FJ441908 | FJ441786 | -- | FJ441969 | -- | -- | -- | -- | FJ441846 | -- |
| *Anotosaura collaris* | -- | -- | -- | -- | -- | -- | AF420910 | -- | -- | -- | -- | -- |
| *Antaresia childreni* | EF545031 | EF545058 | -- | AY099967 | AY099994 | -- | -- | -- | -- | -- | -- | -- |
| *Antaresia maculosa* | EF545029 | EF545056 | -- | -- | EF545102 | -- | -- | -- | -- | -- | -- | -- |
| *Antaresia perthensis* | EF545032 | EF545059 | -- | -- | EF545104 | -- | -- | -- | -- | -- | -- | -- |
| *Antaresia stimsoni* | EF545030 | EF545057 | -- | -- | EF545103 | -- | -- | -- | -- | -- | -- | -- |
| *Antillophis andreae* | AF158442 | AF158511 | -- | -- | FJ416743 | FJ416781 | FJ416817 | -- | -- | -- | -- | FJ416854 |
| *Antillophis parvifrons* | AF158441 | AF158510 | JQ599006 | -- | FJ416740 | FJ416778 | FJ416814 | -- | -- | -- | -- | FJ416851 |
| *Aparallactus capensis* | FJ404129 | AY188045 | -- | AY187967 | AY188006 | -- | FJ404331 | -- | -- | -- | -- | FJ404404 |
| *Aparallactus guentheri* | -- | -- | -- | -- | AY235730 | -- | -- | -- | -- | -- | -- | -- |
| *Aparallactus modestus* | FJ404130 | AY611824 | -- | AY611916 | AY612007 | -- | FJ404332 | -- | -- | -- | -- | FJ404405 |
| *Aparallactus werneri* | -- | -- | EU402626 | AF471116 | AF471035 | -- | U49315 | EU390907 | -- | JN703077 | -- | -- |
| *Apathya cappadocica* | AF080328 | GQ142099 | -- | EF632268 | GQ142127 | -- | -- | -- | -- | -- | EF632223 | -- |
| *Aphaniotis fusca* | AB023749 | AB023771 | -- | -- | -- | AF288228 | -- | -- | -- | -- | -- | -- |
| *Aplopeltura boa* | AF544761 | AF544787 | FJ433984 | AF544715 | JF827673 | -- | U49312 | FJ434085 | -- | EF144068 | -- | -- |
| *Apodora papuana* | EF545027 | EF545054 | FJ433971 | AF544720 | AF241405 | -- | -- | FJ434076 | -- | FJ433925 | AY487405 | FJ433901 |
| *Apostolepis albicollaris* | JQ598793 | JQ598856 | -- | JQ598965 | -- | -- | -- | -- | -- | -- | -- | -- |
| *Apostolepis assimilis* | GQ457781 | GQ457724 | JQ599007 | GQ457843 | -- | -- | -- | -- | -- | -- | -- | -- |
| *Apostolepis cearensis* | JQ598794 | JQ598857 | -- | JQ598966 | -- | -- | -- | -- | -- | -- | -- | -- |
| *Apostolepis dimidiata* | GQ457782 | GQ457725 | JQ599008 | GQ457844 | JQ598917 | -- | -- | -- | -- | -- | -- | -- |
| *Apostolepis flavotorquata* | JQ598795 | JQ598858 | -- | GQ895798 | GQ895854 | -- | -- | -- | -- | -- | -- | -- |
| *Apostolepis sanctaeritae* | JQ598797 | JQ598859 | -- | JQ598969 | -- | -- | -- | -- | -- | -- | -- | -- |
| *Aprasia aurita* | -- | AY134500 | -- | AY134536 | -- | AY134572 | -- | -- | -- | -- | -- | -- |
| *Aprasia fusca* | -- | AY134501 | -- | AY134537 | -- | AY134573 | -- | -- | -- | -- | -- | -- |
| *Aprasia inaurita* | -- | AY134502 | -- | FJ571646 | -- | AY134574 | -- | -- | -- | -- | FJ571632 | -- |
| *Aprasia parapulchella* | -- | GU460139 | -- | AY134539 | -- | AY134575 | -- | -- | GU459741 | -- | HQ426260 | HQ426433 |
| *Aprasia picturata* | -- | AY134504 | -- | AY134540 | -- | AY134576 | -- | -- | -- | -- | -- | -- |
| *Aprasia pseudopulchella* | -- | AY134505 | -- | AY134541 | -- | AY134577 | -- | -- | -- | -- | -- | -- |
| *Aprasia pulchella* | -- | AY134506 | -- | AY134542 | -- | AY134578 | -- | -- | -- | -- | -- | -- |
| *Aprasia repens* | -- | AY134507 | -- | AY134543 | -- | AY134579 | -- | -- | -- | -- | -- | -- |
| *Aprasia smithi* | -- | AY134508 | -- | AY134544 | -- | AY134580 | -- | -- | -- | -- | -- | -- |
| *Aprasia striolata* | -- | AY134509 | -- | AY134545 | -- | AY134581 | -- | -- | -- | -- | -- | -- |
| *Apterygodon vittatus* | AB028771 | AB028782 | -- | DQ675393 | -- | -- | -- | -- | -- | -- | DQ675338 | -- |
| *Archaeolacerta bedriagae* | -- | -- | -- | EF632256 | GQ142126 | -- | -- | -- | -- | -- | EF632211 | -- |
| *Aristelliger georgeensis* | -- | -- | -- | HQ426518 | -- | JX041310 | -- | -- | HQ426173 | -- | HQ426261 | HQ426434 |
| *Aristelliger lar* | -- | -- | -- | EF534931 | -- | JX041311 | -- | -- | EF534847 | -- | EF534805 | EF534973 |
| *Aristelliger praesignis* | -- | -- | -- | HQ426520 | -- | JX041312 | -- | -- | HQ426175 | -- | HQ426262 | HQ426436 |
| *Arizona elegans* | AY122810 | -- | -- | DQ902058 | DQ902101 | DQ902204 | DQ902279 | -- | -- | -- | -- | -- |
| *Arrhyton callilaemum* | -- | AF158509 | -- | -- | FJ416737 | FJ416775 | FJ416811 | -- | -- | -- | -- | FJ416848 |
| *Arrhyton dolichura* | AF158438 | AF158507 | -- | -- | FJ416721 | FJ416759 | FJ416795 | -- | -- | -- | -- | FJ416832 |
| *Arrhyton exiguum* | AF158457 | AF158526 | -- | AF471117 | AF471071 | FJ416762 | FJ416798 | -- | -- | -- | -- | FJ416835 |
| *Arrhyton funereum* | AF158451 | AF158520 | -- | -- | FJ416739 | FJ416777 | FJ416813 | -- | -- | -- | -- | FJ416850 |
| *Arrhyton landoi* | AF158439 | AF158508 | -- | -- | FJ416720 | FJ416758 | FJ416794 | -- | -- | -- | -- | FJ416831 |
| *Arrhyton polylepis* | AF158450 | AF158519 | -- | -- | FJ416738 | FJ416776 | FJ416812 | -- | -- | -- | -- | FJ416849 |
| *Arrhyton procerum* | AF158452 | AF158521 | -- | -- | FJ416723 | FJ416761 | FJ416797 | -- | -- | -- | -- | FJ416834 |
| *Arrhyton supernum* | AF158436 | AF158505 | -- | -- | FJ416718 | FJ416756 | FJ416792 | -- | -- | -- | -- | FJ416829 |
| *Arrhyton taeniatum* | AF158453 | AF158522 | -- | -- | FJ416717 | FJ416755 | FJ416791 | -- | -- | -- | -- | FJ416828 |
| *Arrhyton tanyplectum* | AF158446 | AF158516 | -- | -- | FJ416722 | FJ416760 | FJ416796 | -- | -- | -- | -- | FJ416833 |
| *Arrhyton vittatum* | AF158437 | AF158506 | -- | -- | FJ416719 | FJ416757 | FJ416793 | -- | -- | -- | -- | FJ416830 |
| *Arthrosaura kockii* | AF420680 | AF420721 | EU108350 | -- | -- | -- | AF420866 | EU108016 | -- | -- | -- | -- |
| *Arthrosaura reticulata* | AF420676 | AF420722 | -- | -- | -- | -- | AF420894 | -- | -- | -- | -- | -- |
| *Asaccus platyrhynchus* | -- | -- | -- | EU293670 | -- | HM212529 | -- | -- | EU293693 | -- | EU293625 | EU293715 |
| *Aspidelaps scutatus* | U96790 | AY188046 | -- | AY187968 | AY188007 | -- | -- | -- | -- | -- | -- | -- |
| *Aspidites melanocephalus* | EF545033 | EF545060 | EU402627 | DQ465557 | U69741 | -- | -- | EU390908 | -- | HQ876365 | EU402835 | -- |
| *Aspidites ramsayi* | EF545034 | EF545061 | -- | -- | EF545105 | -- | -- | -- | -- | -- | -- | -- |
| *Aspidomorphus lineaticollis* | GQ397247 | GQ397239 | -- | -- | -- | -- | -- | -- | -- | -- | -- | -- |
| *Aspidomorphus muelleri* | -- | EU547139 | -- | -- | AF217814 | -- | EU546999 | -- | -- | -- | EU366434 | -- |
| *Aspidomorphus schlegeli* | GQ397246 | GQ397238 | -- | -- | -- | -- | -- | -- | -- | -- | -- | -- |
| *Aspidoscelis burti* | AY046428 | AY046470 | -- | -- | -- | -- | -- | -- | -- | -- | -- | -- |
| *Aspidoscelis ceralbensis* | -- | -- | -- | -- | AF006293 | -- | -- | -- | -- | -- | -- | -- |
| *Aspidoscelis communis* | -- | HM012702 | -- | -- | -- | -- | -- | -- | -- | -- | -- | -- |
| *Aspidoscelis costatus* | AY046429 | AY046471 | -- | -- | -- | -- | -- | -- | -- | -- | -- | -- |
| *Aspidoscelis deppei* | AY046431 | AY046473 | -- | -- | AF006303 | -- | -- | -- | -- | -- | -- | -- |
| *Aspidoscelis gularis* | AY046433 | AY046475 | -- | -- | -- | -- | AF026195 | -- | -- | -- | -- | -- |
| *Aspidoscelis guttatus* | AY046434 | AY046476 | -- | -- | AF006302 | -- | -- | -- | -- | -- | -- | -- |
| *Aspidoscelis hyperythrus* | AY046435 | AY046477 | -- | -- | AF006301 | -- | -- | -- | -- | -- | -- | -- |
| *Aspidoscelis inornatus* | AY046436 | AY046478 | -- | -- | -- | -- | DQ113993 | -- | -- | -- | -- | -- |
| *Aspidoscelis laredoensis* | -- | -- | -- | -- | -- | -- | AF026196 | -- | -- | -- | -- | -- |
| *Aspidoscelis lineattissimus* | -- | HM012703 | -- | -- | -- | -- | -- | -- | -- | -- | -- | -- |
| *Aspidoscelis marmoratus* | AY046449 | AY046491 | -- | -- | -- | -- | -- | -- | -- | -- | -- | -- |
| *Aspidoscelis sexlineatus* | AF236047 | AY046486 | -- | -- | -- | GQ374464 | AF026178 | -- | -- | -- | -- | -- |
| *Aspidoscelis tigris* | AF206585 | AY046489 | EU402619 | AF039481 | AF006288 | U71332 | AF026173 | EU390903 | HQ426250 | HM161054 | AY662620 | HQ426512 |
| *Aspidoscelis velox* | -- | -- | EU108351 | EU116675 | EU116504 | -- | -- | EU108017 | -- | -- | EU108525 | -- |
| *Aspidura drummondhayi* | KC347305 | KC347340 | -- | KC347379 | KC347455 | -- | KC347493 | -- | -- | -- | KC347417 | -- |
| *Aspidura guentheri* | KC347306 | KC347341 | -- | KC347380 | KC347456 | -- | KC347494 | -- | -- | -- | KC347418 | -- |
| *Aspidura trachyprocta* | KC347308 | KC347343 | -- | KC347382 | KC347458 | -- | KC347496 | -- | -- | -- | KC347420 | -- |
| *Asthenodipsas vertebralis* | -- | -- | -- | -- | AY425808 | -- | -- | -- | -- | -- | -- | -- |
| *Astrotia stokesii* | -- | DQ234010 | -- | FJ587182 | DQ233929 | -- | FJ593206 | -- | -- | -- | FJ587104 | -- |
| *Asymblepharus alaicus* | -- | -- | -- | -- | -- | AY607281 | -- | -- | -- | -- | -- | -- |
| *Asymblepharus sikimmensis* | -- | -- | -- | -- | -- | AY662549 | -- | -- | -- | -- | AY662631 | -- |
| *Ateuchosaurus pellopleurus* | AY818756 | -- | -- | AY818790 | FJ972226 | -- | -- | -- | -- | -- | -- | -- |
| *Atheris barbouri* | -- | -- | -- | -- | AJ275686 | -- | -- | -- | -- | -- | -- | -- |
| *Atheris ceratophora* | DQ305410 | DQ305433 | -- | -- | DQ305456 | -- | DQ305474 | -- | -- | -- | -- | -- |
| *Atheris chlorechis* | EU624244 | EU624278 | -- | -- | AJ275679 | -- | EU624211 | -- | -- | -- | -- | -- |
| *Atheris desaixi* | -- | AJ275733 | -- | -- | AJ275680 | -- | -- | -- | -- | -- | -- | -- |
| *Atheris hispida* | -- | AJ275734 | -- | -- | AJ275681 | -- | -- | -- | -- | -- | -- | -- |
| *Atheris nitschei* | AY223650 | AY223663 | -- | AF471125 | AF471070 | -- | AY223618 | -- | -- | -- | -- | -- |
| *Atheris squamigera* | AF544762 | EU624279 | -- | AF544734 | EU624303 | -- | EU624212 | -- | -- | -- | -- | -- |
| *Atlantolacerta andreanskyi* | AF206603 | GQ142093 | -- | GQ142144 | GQ142117 | -- | -- | -- | -- | -- | GQ142154 | -- |
| *Atractaspis bibronii* | FJ404131 | AY188047 | -- | AY187969 | AY188008 | -- | U49314 | -- | -- | -- | -- | FJ404406 |
| *Atractaspis boulengeri* | FJ404132 | AY611833 | -- | AY611925 | AY612016 | -- | FJ404334 | -- | -- | -- | -- | FJ404407 |
| *Atractaspis corpulenta* | Z46597 | AY611837 | -- | AY611929 | AY612020 | -- | FJ404335 | -- | -- | -- | DQ993174 | FJ404408 |
| *Atractaspis irregularis* | -- | -- | JN614212 | -- | -- | -- | -- | -- | -- | JN703063 | -- | -- |
| *Atractaspis microlepidota* | -- | -- | -- | AF471127 | AF471046 | -- | -- | -- | -- | -- | -- | -- |
| *Atractaspis micropholis* | AF544740 | AY611823 | FJ433994 | AF544677 | AF039261 | -- | FJ404336 | FJ434095 | -- | EF144078 | -- | EF144105 |
| *Atractus albuquerquei* | GQ457783 | GQ457726 | JQ599009 | GQ457845 | JQ598918 | -- | -- | -- | -- | -- | -- | -- |
| *Atractus badius* | AF158425 | AF158485 | -- | -- | -- | -- | -- | -- | -- | -- | -- | -- |
| *Atractus elaps* | -- | -- | -- | -- | EF078536 | -- | EF078584 | GU353273 | -- | -- | -- | -- |
| *Atractus flammigerus* | AF158402 | AF158471 | -- | -- | -- | -- | -- | -- | -- | -- | -- | -- |
| *Atractus reticulatus* | JQ598798 | -- | -- | JQ598970 | -- | -- | -- | -- | -- | -- | -- | -- |
| *Atractus schach* | JQ598799 | JQ598860 | -- | JQ598971 | -- | -- | -- | -- | -- | -- | -- | -- |
| *Atractus trihedrurus* | GQ457784 | GQ457727 | JQ599010 | GQ457846 | JQ598919 | -- | -- | -- | -- | -- | -- | -- |
| *Atractus wagleri* | -- | -- | -- | -- | GQ334480 | -- | GQ334581 | GQ334664 | -- | -- | -- | -- |
| *Atractus zebrinus* | JQ598800 | JQ598861 | -- | JQ598972 | -- | -- | -- | -- | -- | -- | -- | -- |
| *Atractus zidoki* | AF158426 | AF158487 | -- | -- | -- | -- | -- | -- | -- | -- | -- | -- |
| *Atretium schistosum* | KC347309 | PEA | -- | KC347383 | KC347459 | -- | KC347497 | -- | -- | -- | KC347421 | -- |
| *Atretium yunnanensis* | -- | -- | -- | JQ687448 | GQ281787 | JQ687463 | JQ687423 | -- | -- | -- | -- | -- |
| *Atropoides nummifer* | AF057207 | DQ305445 | -- | -- | DQ061195 | -- | AY220336 | -- | -- | -- | -- | -- |
| *Atropoides occiduus* | DQ305423 | DQ305446 | -- | -- | DQ061194 | -- | DQ061219 | -- | -- | -- | -- | -- |
| *Atropoides olmec* | AY223656 | AY223669 | -- | -- | DQ061196 | -- | AY220345 | -- | -- | -- | -- | -- |
| *Atropoides picadoi* | AF057208 | AF057255 | -- | -- | DQ061197 | -- | U41872 | -- | -- | -- | -- | -- |
| *Aulura anomala* | -- | FJ441713 | FJ441895 | FJ441773 | -- | FJ441956 | -- | -- | -- | -- | FJ441833 | -- |
| *Australolacerta australis* | FR751395 | DQ871152 | -- | -- | FR751398 | DQ871094 | -- | -- | -- | -- | DQ871208 | -- |
| *Austrelaps labialis* | EU547126 | EU547175 | -- | EU546939 | -- | -- | EU547029 | -- | -- | -- | EU546900 | -- |
| *Austrelaps superbus* | EU547127 | EU547176 | -- | -- | AF217815 | -- | EU547030 | -- | -- | -- | EU546901 | -- |
| *Austrotyphlops ammodytes* | AY442890 | AY442842 | -- | -- | -- | -- | -- | -- | -- | -- | -- | -- |
| *Austrotyphlops australis* | AY442891 | AM236346 | GU902409 | AF039474 | AM236346 | AM236346 | AM236346 | GU902580 | -- | -- | GU902659 | -- |
| *Austrotyphlops bituberculatus* | AY442893 | AY442831 | GU902403 | -- | -- | -- | -- | GU902574 | -- | -- | GU902653 | -- |
| *Austrotyphlops diversus* | -- | -- | GU902411 | -- | -- | -- | -- | GU902582 | -- | -- | GU902661 | -- |
| *Austrotyphlops endoterus* | -- | -- | GU902399 | -- | -- | -- | -- | GU902570 | -- | -- | GU902649 | -- |
| *Austrotyphlops ganei* | -- | -- | GU902412 | -- | -- | -- | -- | GU902583 | -- | -- | GU902662 | -- |
| *Austrotyphlops grypus* | AY442898 | AY442835 | GU902413 | -- | -- | -- | -- | GU902584 | -- | -- | -- | -- |
| *Austrotyphlops guentheri* | -- | -- | GU902404 | -- | -- | -- | -- | GU902575 | -- | -- | GU902654 | -- |
| *Austrotyphlops hamatus* | AY442894 | AY442832 | GU902401 | -- | -- | -- | -- | GU902572 | -- | -- | GU902651 | -- |
| *Austrotyphlops howi* | -- | -- | GU902414 | -- | -- | -- | -- | GU902585 | -- | -- | GU902664 | -- |
| *Austrotyphlops kimberleyensis* | -- | -- | GU902406 | -- | -- | -- | -- | GU902577 | -- | -- | GU902656 | -- |
| *Austrotyphlops leptosomus* | AY442889 | AY442830 | -- | -- | -- | -- | -- | -- | -- | -- | -- | -- |
| *Austrotyphlops ligatus* | -- | -- | GU902405 | -- | -- | -- | -- | GU902576 | -- | -- | GU902655 | -- |
| *Austrotyphlops longissimus* | AY442901 | AY442838 | GU902408 | -- | -- | -- | -- | GU902579 | -- | -- | -- | -- |
| *Austrotyphlops pilbarensis* | AY442896 | AY442834 | GU902400 | -- | -- | -- | -- | GU902571 | -- | -- | GU902650 | -- |
| *Austrotyphlops pinguis* | -- | -- | GU902415 | -- | -- | -- | -- | GU902586 | -- | -- | GU902665 | -- |
| *Austrotyphlops splendidus* | -- | -- | GU902416 | -- | -- | -- | -- | GU902587 | -- | -- | GU902666 | -- |
| *Austrotyphlops troglodytes* | -- | -- | GU902417 | -- | -- | -- | -- | GU902588 | -- | -- | GU902667 | -- |
| *Austrotyphlops unguirostris* | AY442902 | AY442837 | GU902407 | -- | -- | -- | -- | GU902578 | -- | -- | GU902657 | -- |
| *Austrotyphlops waitii* | AY442892 | AY442840 | GU902402 | -- | -- | -- | -- | GU902573 | -- | -- | GU902652 | -- |
| *Azemiops feae* | AF512748 | AY352713 | EU402628 | AF544695 | AY352747 | -- | AY352808 | EU390909 | -- | JN703086 | EU402836 | -- |
| *Bachia barbouri* | DQ383205 | DQ383216 | -- | DQ383193 | -- | -- | -- | -- | -- | -- | -- | -- |
| *Bachia bicolor* | -- | DQ383217 | -- | DQ383194 | -- | -- | -- | -- | -- | -- | -- | -- |
| *Bachia bresslaui* | -- | AF420755 | -- | AF420860 | -- | -- | AF420876 | -- | -- | -- | -- | -- |
| *Bachia dorbignyi* | AF420688 | AF420754 | -- | -- | -- | -- | AF420892 | -- | -- | -- | -- | -- |
| *Bachia flavescens* | AF420705 | AF420753 | -- | AF420859 | -- | -- | AF420869 | -- | -- | -- | -- | -- |
| *Bachia heteropa* | DQ383210 | DQ383213 | -- | -- | -- | -- | -- | -- | -- | -- | -- | -- |
| *Bachia huallagana* | DQ383212 | DQ383224 | -- | DQ383201 | -- | -- | -- | -- | -- | -- | -- | -- |
| *Bachia intermedia* | DQ383204 | DQ383215 | -- | -- | -- | -- | -- | -- | -- | -- | -- | -- |
| *Bachia panoplia* | DQ383207 | DQ383219 | -- | -- | -- | -- | -- | -- | -- | -- | -- | -- |
| *Bachia peruana* | DQ383206 | DQ383218 | -- | DQ383195 | -- | -- | -- | -- | -- | -- | -- | -- |
| *Bachia scolecoides* | DQ383211 | DQ383223 | -- | DQ383200 | -- | -- | -- | -- | -- | -- | -- | -- |
| *Bachia trisanale* | DQ383209 | DQ383221 | -- | DQ383198 | -- | -- | -- | -- | -- | -- | -- | -- |
| *Balanophis ceylonensis* | KC347310 | KC347344 | -- | KC347384 | KC347460 | -- | KC347498 | -- | -- | -- | KC347422 | -- |
| *Barisia herrerae* | -- | -- | -- | -- | -- | -- | AY605120 | -- | -- | -- | -- | -- |
| *Barisia imbricata* | AF056612 | -- | -- | -- | AF056597 | AF085613 | AY660760 | JQ844944 | -- | -- | -- | -- |
| *Barisia levicollis* | -- | -- | -- | -- | -- | AY742922 | -- | -- | -- | -- | -- | -- |
| *Barisia rudicollis* | -- | -- | -- | -- | -- | -- | AY660761 | -- | -- | -- | -- | -- |
| *Bartleia jigurru* | -- | -- | HQ655160 | HQ655197 | -- | -- | -- | -- | -- | -- | -- | -- |
| *Basiliscus basiliscus* | AF338330 | -- | JF806017 | -- | -- | -- | -- | JF804532 | -- | HQ876326 | JF806202 | -- |
| *Basiliscus galeritus* | -- | -- | -- | -- | -- | AF528714 | -- | -- | -- | -- | FJ356743 | -- |
| *Basiliscus plumifrons* | -- | -- | AY987969 | AY987986 | -- | U82680 | -- | -- | -- | HM161055 | AY662599 | -- |
| *Basiliscus vittatus* | AB218883 | NC_012829 | -- | -- | NC_012829 | AF528715 | NC_012829 | -- | -- | -- | -- | -- |
| *Bassiana duperreyi* | AY818752 | -- | -- | AY818784 | AY818822 | -- | -- | -- | -- | -- | -- | -- |
| *Bassiana trilineata* | -- | -- | HQ655161 | HQ655198 | -- | -- | -- | -- | -- | -- | -- | -- |
| *Bavayia cyclura* | -- | AF215246 | -- | HQ426521 | -- | JX041315 | -- | -- | HQ426176 | -- | HQ426264 | HQ426437 |
| *Bavayia geitaina* | -- | -- | -- | JQ945532 | -- | JF972432 | -- | -- | JQ945353 | -- | JQ945285 | JQ945424 |
| *Bavayia goroensis* | -- | -- | -- | -- | -- | EU054304 | -- | -- | -- | -- | -- | -- |
| *Bavayia madjo* | -- | GU460149 | -- | JQ945533 | -- | GU459950 | -- | -- | GU459751 | -- | GU459549 | JQ945425 |
| *Bavayia montana* | -- | -- | -- | -- | -- | DQ533738 | -- | -- | -- | -- | -- | -- |
| *Bavayia ornata* | -- | -- | -- | -- | -- | DQ533737 | -- | -- | -- | -- | -- | -- |
| *Bavayia pulchella* | -- | -- | -- | -- | -- | DQ533739 | -- | -- | -- | -- | -- | -- |
| *Bavayia sauvagii* | -- | -- | -- | FJ855468 | -- | DQ533740 | -- | -- | -- | -- | FJ855448 | -- |
| *Bellatorias frerei* | -- | -- | -- | -- | -- | -- | AF530264 | -- | -- | -- | -- | -- |
| *Bellatorias major* | -- | -- | HQ655162 | HQ655199 | -- | -- | -- | -- | -- | -- | -- | -- |
| *Bibilava epistibes* | -- | DQ979965 | -- | DQ979972 | DQ979979 | -- | -- | -- | -- | -- | -- | -- |
| *Bibilava infrasignatus* | -- | -- | -- | -- | DQ979994 | -- | -- | -- | -- | -- | -- | -- |
| *Bibilava lateralis* | -- | AF215270 | -- | DQ979970 | DQ979977 | -- | -- | -- | -- | -- | -- | -- |
| *Bibilava martae* | -- | DQ979967 | -- | DQ979974 | DQ979981 | -- | -- | -- | -- | -- | -- | -- |
| *Bibilava stumpffi* | -- | DQ979969 | -- | DQ979976 | DQ979986 | -- | -- | -- | -- | -- | -- | -- |
| *Bipes biporus* | -- | -- | HM160582 | AF039482 | AF013251 | U71335 | NC_006287 | JN568335 | -- | HM161056 | AY662616 | DQ119632 |
| *Bipes canaliculatus* | EU203658 | EU203658 | FJ441849 | FJ518700 | AY605484 | NC_006288 | NC_006288 | GU456033 | -- | DQ119614 | FJ518701 | -- |
| *Bipes tridactylus* | -- | -- | -- | -- | AY605479 | NC_006286 | NC_006286 | -- | -- | -- | -- | -- |
| *Bitia hydroides* | EF395872 | EF395847 | -- | EF395921 | EF395896 | -- | -- | -- | -- | -- | -- | -- |
| *Bitis arietans* | EU852316 | JF357953 | -- | -- | AY235728 | -- | EU852304 | -- | -- | -- | EU852328 | -- |
| *Bitis atropos* | EU624246 | EU624281 | -- | -- | AJ275691 | -- | EU624214 | -- | -- | -- | -- | -- |
| *Bitis caudalis* | EU624247 | EU624282 | -- | -- | AJ275693 | -- | EU624215 | -- | -- | -- | -- | -- |
| *Bitis cornuta* | EU624248 | EU624283 | -- | -- | AJ275694 | -- | EU624216 | -- | -- | -- | -- | -- |
| *Bitis gabonica* | EU624249 | EU624284 | -- | -- | AJ275695 | -- | EU624217 | -- | -- | -- | -- | -- |
| *Bitis nasicornis* | DQ305411 | DQ305434 | -- | AY187970 | AY188009 | -- | DQ305475 | -- | -- | -- | -- | -- |
| *Bitis peringueyi* | DQ305412 | DQ305435 | -- | -- | DQ305458 | -- | DQ305476 | -- | -- | -- | -- | -- |
| *Bitis rubida* | EU624251 | EU624286 | -- | -- | EU624306 | -- | EU624219 | -- | -- | -- | -- | -- |
| *Bitis worthingtoni* | EU624252 | AJ275745 | -- | -- | AJ275692 | -- | EU624220 | -- | -- | -- | -- | -- |
| *Bitis xeropaga* | EU624253 | EU624287 | -- | -- | EU624307 | -- | EU624221 | -- | -- | -- | -- | -- |
| *Blaesodactylus antongilensis* | -- | -- | JQ073117 | JQ945534 | -- | EU054254 | EU054182 | -- | EU054206 | -- | EU054230 | JQ945426 |
| *Blaesodactylus boivini* | EU596607 | -- | EU596841 | -- | -- | EU054252 | EU054180 | -- | EU054204 | -- | EU054228 | -- |
| *Blaesodactylus sakalava* | -- | -- | -- | -- | -- | EU054251 | EU054179 | -- | EU054203 | -- | EU054227 | -- |
| *Blanus cinereus* | NC_012433 | EF036388 | EU108349 | DQ324864 | NC_012433 | NC_012433 | EF036460 | EU108015 | -- | -- | EU108523 | -- |
| *Blanus mettetali* | -- | EF036461 | -- | -- | -- | -- | EF036462 | -- | -- | -- | -- | -- |
| *Blanus strauchi* | EU203660 | FJ518702 | FJ441847 | AY444024 | -- | FJ518703 | EF036463 | EU203628 | -- | EU203630 | AY444050 | EU203634 |
| *Blanus tingitanus* | -- | EF036466 | -- | DQ324866 | -- | DQ902269 | DQ902331 | -- | -- | -- | -- | -- |
| *Boa constrictor* | AB177354 | AF215275 | EU402629 | AF471115 | AF471036 | AB177354 | AB177354 | -- | -- | HQ876357 | AY487351 | FJ433905 |
| *Bogertia lutzae* | -- | JN935549 | -- | HQ426522 | -- | JX041415 | -- | -- | HQ426177 | -- | HQ426265 | HQ426438 |
| *Bogertophis rosaliae* | AY122815 | -- | -- | DQ902059 | DQ902102 | DQ902205 | DQ902280 | -- | -- | -- | -- | -- |
| *Bogertophis subocularis* | AY122845 | -- | -- | DQ902060 | DQ902103 | DQ902206 | DQ902281 | -- | -- | -- | -- | -- |
| *Boiga barnesii* | KC347311 | KC347345 | -- | KC347385 | KC347461 | -- | KC347499 | -- | -- | -- | KC347423 | -- |
| *Boiga beddomei* | KC347312 | KC347346 | -- | KC347386 | KC347462 | -- | KC347500 | -- | -- | -- | KC347424 | -- |
| *Boiga ceylonensis* | KC347313 | KC347347 | -- | KC347387 | KC347463 | -- | KC347501 | -- | -- | -- | KC347425 | -- |
| *Boiga cynodon* | Z46468 | AF139568 | -- | -- | -- | -- | -- | -- | -- | -- | -- | -- |
| *Boiga dendrophila* | -- | -- | -- | AF471128 | AF471089 | -- | U49303 | -- | -- | -- | -- | -- |
| *Boiga forsteni* | KC347314 | KC347348 | -- | KC347388 | KC347464 | -- | KC347502 | -- | -- | -- | KC347426 | -- |
| *Boiga irregularis* | -- | AF139564 | -- | FJ710793 | FJ710794 | -- | -- | -- | -- | -- | -- | -- |
| *Boiga kraepelini* | -- | -- | -- | FJ710795 | FJ710796 | -- | -- | -- | -- | -- | -- | -- |
| *Boiga multomaculata* | -- | -- | -- | FJ710797 | FJ710798 | -- | -- | -- | -- | -- | -- | -- |
| *Boiga pulverulenta* | -- | -- | -- | AF471118 | AF471047 | -- | -- | -- | -- | -- | -- | -- |
| *Boiga trigonata* | KC347315 | KC347349 | -- | KC347389 | KC347465 | -- | KC347503 | -- | -- | -- | KC347427 | -- |
| *Boiruna maculata* | GQ457785 | JQ598862 | JQ599011 | GQ895799 | JQ598920 | -- | -- | -- | -- | -- | -- | -- |
| *Bothriechis aurifer* | DQ305425 | DQ305448 | -- | -- | DQ305466 | -- | DQ305483 | -- | -- | -- | -- | -- |
| *Bothriechis bicolor* | DQ305426 | DQ305449 | -- | -- | DQ305467 | -- | DQ305484 | -- | -- | -- | -- | -- |
| *Bothriechis lateralis* | AF057211 | AF057258 | -- | -- | AF292572 | -- | U41873 | -- | -- | -- | -- | -- |
| *Bothriechis marchi* | DQ305428 | DQ305451 | -- | -- | DQ305469 | -- | DQ305486 | -- | -- | -- | -- | -- |
| *Bothriechis nigroviridis* | AF057212 | AF057259 | -- | -- | AY223589 | -- | AY223635 | -- | -- | -- | -- | -- |
| *Bothriechis rowleyi* | DQ305427 | DQ305450 | -- | -- | DQ305468 | -- | DQ305485 | -- | -- | -- | -- | -- |
| *Bothriechis schlegelii* | AF057213 | AF057260 | FJ433983 | AF544680 | AF292573 | -- | U41874 | FJ434084 | -- | EF144067 | AY487374 | EF144095 |
| *Bothriechis thalassinus* | DQ305424 | DQ305447 | -- | -- | DQ305465 | -- | DQ305482 | -- | -- | -- | -- | -- |
| *Bothriopsis bilineata* | AF057214 | AF057261 | -- | -- | AY223591 | -- | U41875 | -- | -- | -- | -- | -- |
| *Bothriopsis chloromelas* | DQ305430 | DQ305453 | -- | -- | DQ305471 | -- | DQ305488 | -- | -- | -- | -- | -- |
| *Bothriopsis pulchra* | JN870179 | -- | -- | -- | AF292593 | -- | AF292631 | -- | -- | -- | -- | -- |
| *Bothriopsis taeniata* | AF057215 | AF057262 | -- | -- | AY223592 | -- | AY223637 | -- | -- | -- | -- | -- |
| *Bothrochilus boa* | EF545028 | EF545055 | -- | -- | EF545101 | -- | -- | -- | -- | -- | -- | -- |
| *Bothrocophias campbelli* | -- | -- | -- | -- | AF292584 | -- | AF292622 | -- | -- | -- | -- | -- |
| *Bothrocophias hyoprora* | -- | -- | -- | -- | AF292576 | -- | AF292614 | -- | -- | -- | -- | -- |
| *Bothrocophias microphthalmus* | AY223657 | AY223670 | -- | -- | AF292577 | -- | AF292615 | -- | -- | -- | -- | -- |
| *Bothrolycus ater* | FJ404144 | AY611859 | -- | AY611950 | AY612041 | -- | FJ404347 | -- | -- | -- | -- | FJ404419 |
| *Bothrophthalmus brunneus* | FJ404145 | AY611874 | -- | AY611965 | AY612056 | -- | FJ404348 | -- | -- | -- | -- | FJ404420 |
| *Bothrophthalmus lineatus* | FJ404146 | FJ404198 | -- | AF471129 | AF471090 | -- | FJ404349 | -- | -- | -- | -- | FJ404421 |
| *Bothrops alcatraz* | -- | -- | -- | -- | AY865824 | -- | -- | -- | -- | -- | -- | -- |
| *Bothrops alternatus* | AY027777 | EU867263 | -- | -- | AY223601 | -- | AF292617 | -- | -- | -- | -- | -- |
| *Bothrops ammodytoides* | AY223658 | AY223671 | -- | -- | AY223595 | -- | AY223639 | -- | -- | -- | -- | -- |
| *Bothrops asper* | EU624239 | GQ372868 | EU402630 | -- | AF292600 | -- | U41876 | EU390910 | -- | JN703092 | EU402838 | -- |
| *Bothrops atrox* | GQ428494 | AY223672 | DQ469794 | -- | AF292604 | -- | AF246278 | DQ469792 | -- | -- | DQ469790 | -- |
| *Bothrops brazili* | EU867252 | EU867264 | -- | -- | AF292597 | -- | AF292635 | -- | -- | -- | -- | -- |
| *Bothrops caribbaeus* | -- | -- | -- | -- | AF292598 | -- | AF292636 | -- | -- | -- | -- | -- |
| *Bothrops colombiensis* | -- | -- | -- | -- | AF292602 | -- | AF292640 | -- | -- | -- | -- | -- |
| *Bothrops cotiara* | AF057217 | AF057264 | -- | -- | AF292581 | -- | AF292619 | -- | -- | -- | -- | -- |
| *Bothrops diporus* | DQ305431 | DQ305454 | -- | -- | DQ305472 | -- | DQ305489 | -- | -- | -- | -- | -- |
| *Bothrops erythromelas* | AF057219 | AF057266 | -- | -- | AY865653 | -- | U41877 | -- | -- | -- | -- | -- |
| *Bothrops fonsecai* | -- | -- | -- | -- | AF292580 | -- | AF292618 | -- | -- | -- | -- | -- |
| *Bothrops insularis* | AF057216 | AF057263 | -- | -- | AY865660 | -- | AF188705 | -- | -- | -- | -- | -- |
| *Bothrops itapetiningae* | EU867253 | EU867265 | -- | -- | AF292582 | -- | AF292620 | -- | -- | -- | -- | -- |
| *Bothrops jararaca* | EU867254 | EU867266 | -- | -- | AY865819 | -- | AF292627 | -- | -- | -- | -- | -- |
| *Bothrops jararacussu* | AY223661 | AY223674 | -- | -- | AF292596 | -- | AF292634 | -- | -- | -- | -- | -- |
| *Bothrops lanceolatus* | -- | -- | -- | -- | AF292599 | -- | AF292637 | -- | -- | -- | -- | -- |
| *Bothrops leucurus* | EU867255 | EU867267 | -- | -- | AF246276 | -- | AF246283 | -- | -- | -- | -- | -- |
| *Bothrops marajoensis* | -- | -- | -- | -- | AF292605 | -- | AF292643 | -- | -- | -- | -- | -- |
| *Bothrops moojeni* | EU867257 | EU867271 | -- | -- | AF292606 | -- | AF292644 | -- | -- | -- | -- | -- |
| *Bothrops neuwiedi* | -- | JQ627282 | -- | -- | AF292587 | -- | AF292625 | -- | -- | -- | -- | -- |
| *Bothrops pictus* | -- | -- | -- | -- | AF292583 | -- | AF292621 | -- | -- | -- | -- | -- |
| *Bothrops punctata* | -- | -- | -- | -- | AF292594 | -- | AF292632 | -- | -- | -- | -- | -- |
| *Brachylophus fasciatus* | -- | -- | AY987980 | AY987993 | AF020257 | AF528721 | U66238 | JF804533 | -- | JF804579 | AY988026 | -- |
| *Brachylophus vitiensis* | -- | -- | -- | -- | AF459039 | -- | -- | -- | -- | -- | -- | -- |
| *Brachymeles apus* | -- | -- | HQ907234 | -- | -- | -- | -- | -- | -- | HQ907641 | -- | -- |
| *Brachymeles bicolor* | AY818761 | -- | HQ907281 | AY818797 | FJ972227 | -- | -- | -- | -- | HQ907689 | -- | -- |
| *Brachymeles bonitae* | -- | -- | HQ907250 | -- | -- | JN981990 | -- | -- | -- | HM161057 | HM161152 | -- |
| *Brachymeles boulengeri* | -- | -- | HQ907284 | -- | -- | -- | -- | -- | -- | HQ907692 | -- | -- |
| *Brachymeles cebuensis* | -- | -- | HQ907316 | -- | -- | -- | -- | -- | -- | HQ907724 | -- | -- |
| *Brachymeles elerae* | -- | -- | HQ907275 | -- | -- | -- | -- | -- | -- | HQ907683 | -- | -- |
| *Brachymeles gracilis* | AY649103 | AY649143 | HQ907295 | -- | -- | -- | -- | JN568322 | -- | HQ907703 | JN654856 | -- |
| *Brachymeles minimus* | -- | -- | HQ907310 | -- | -- | -- | -- | -- | -- | HQ907718 | -- | -- |
| *Brachymeles pathfinderi* | -- | -- | HQ907291 | -- | -- | -- | -- | -- | -- | HQ907699 | -- | -- |
| *Brachymeles samarensis* | -- | -- | HQ907252 | -- | -- | -- | -- | -- | -- | HQ907731 | -- | -- |
| *Brachymeles schadenbergi* | -- | -- | HQ907307 | -- | -- | -- | -- | -- | -- | HQ907715 | -- | -- |
| *Brachymeles talinis* | AY649104 | AY649144 | HQ907266 | -- | -- | -- | -- | -- | -- | HQ907674 | -- | -- |
| *Brachymeles tridactylus* | -- | -- | HQ907239 | -- | -- | -- | -- | -- | -- | HQ907646 | -- | -- |
| *Brachyophidium rhodogaster* | AY700992 | AY701023 | -- | -- | -- | -- | -- | -- | -- | -- | -- | -- |
| *Bradypodion atromontanum* | -- | AY649401 | -- | -- | -- | -- | -- | -- | -- | -- | -- | -- |
| *Bradypodion caffrum* | -- | -- | -- | -- | -- | AY289864 | -- | -- | -- | -- | -- | -- |
| *Bradypodion damaranum* | -- | AY555196 | JQ073092 | -- | -- | AY555220 | -- | -- | -- | -- | DQ996646 | -- |
| *Bradypodion dracomontanum* | -- | AY555217 | -- | -- | -- | AY289892 | -- | -- | -- | -- | -- | -- |
| *Bradypodion gutturale* | -- | AY555218 | -- | -- | -- | AY289915 | -- | -- | -- | -- | -- | -- |
| *Bradypodion karrooicum* | -- | AY289839 | -- | -- | -- | AY289896 | -- | -- | -- | -- | -- | -- |
| *Bradypodion melanocephalum* | -- | EU268208 | -- | -- | -- | AY289881 | -- | -- | -- | -- | DQ996647 | -- |
| *Bradypodion nemorale* | -- | EU877680 | -- | -- | -- | -- | -- | -- | -- | -- | -- | -- |
| *Bradypodion occidentale* | -- | HQ130519 | -- | FJ984260 | -- | AY289911 | -- | -- | -- | -- | -- | -- |
| *Bradypodion pumilum* | -- | DQ234630 | -- | -- | -- | AY289914 | AF443237 | -- | -- | -- | DQ996648 | -- |
| *Bradypodion setaroi* | -- | DQ234637 | -- | -- | -- | AY289877 | -- | -- | -- | -- | -- | -- |
| *Bradypodion taeniabronchum* | -- | AY555204 | -- | -- | -- | AY289884 | -- | -- | -- | -- | -- | -- |
| *Bradypodion thamnobates* | -- | EU268210 | -- | -- | -- | AY289872 | -- | -- | -- | -- | -- | -- |
| *Bradypodion transvaalense* | -- | EU268211 | -- | -- | -- | AY289875 | -- | -- | -- | -- | -- | -- |
| *Bradypodion ventrale* | -- | DQ234636 | -- | -- | -- | AY289882 | -- | -- | -- | -- | DQ996649 | -- |
| *Broghammerus reticulatus* | Z46448 | EF545062 | FJ433969 | AF039472 | U69860 | -- | -- | FJ434074 | -- | FJ433923 | EU624119 | -- |
| *Broghammerus timoriensis* | EF545036 | EF545063 | -- | -- | EF545106 | -- | -- | -- | -- | -- | -- | -- |
| *Bronchocela cristatella* | -- | EU503024 | -- | -- | -- | AF288229 | -- | -- | -- | -- | -- | -- |
| *Bronia brasiliana* | -- | FJ441708 | FJ441891 | FJ441768 | -- | FJ441951 | -- | -- | -- | -- | FJ441828 | -- |
| *Bronia kraoh* | -- | FJ441692 | FJ441875 | FJ441752 | -- | FJ441935 | -- | -- | -- | -- | FJ441812 | -- |
| *Bronia saxosa* | -- | FJ441710 | FJ441892 | FJ441770 | -- | FJ441953 | -- | -- | -- | -- | FJ441830 | -- |
| *Brookesia ambreensis* | -- | -- | -- | FJ984313 | -- | FJ975197 | FJ981807 | -- | -- | -- | FJ984243 | -- |
| *Brookesia antakarana* | -- | -- | -- | FJ984312 | -- | FJ975196 | FJ981806 | -- | -- | -- | FJ984242 | -- |
| *Brookesia betschi* | -- | -- | -- | FJ984296 | -- | FJ975180 | FJ981787 | -- | -- | -- | FJ984226 | -- |
| *Brookesia bonsi* | -- | -- | -- | -- | -- | -- | AF443248 | -- | -- | -- | -- | -- |
| *Brookesia brygooi* | -- | JN674044 | JF806004 | FJ984306 | -- | AF448774 | FJ981800 | JF804534 | -- | JF804580 | FJ984236 | -- |
| *Brookesia decaryi* | AB474914 | HQ130512 | -- | FJ984308 | -- | FJ975192 | FJ981802 | -- | -- | -- | FJ984238 | -- |
| *Brookesia dentata* | -- | JN674046 | -- | FJ984289 | -- | FJ975174 | FJ981778 | -- | -- | -- | FJ984218 | -- |
| *Brookesia ebenaui* | -- | -- | -- | FJ984300 | -- | FJ975184 | FJ981791 | -- | -- | -- | FJ984230 | -- |
| *Brookesia exarmata* | -- | JN674048 | -- | FJ984291 | -- | JN674004 | FJ981780 | -- | -- | -- | FJ984220 | -- |
| *Brookesia griveaudi* | -- | -- | -- | FJ984321 | -- | FJ975204 | FJ981815 | -- | -- | -- | FJ984251 | -- |
| *Brookesia karchei* | -- | JN674049 | -- | FJ984285 | -- | FJ975170 | FJ981773 | -- | -- | -- | FJ984214 | -- |
| *Brookesia lineata* | -- | -- | -- | FJ984322 | -- | FJ975206 | FJ981816 | -- | -- | -- | FJ984252 | -- |
| *Brookesia lolontany* | -- | -- | -- | -- | -- | -- | AF443234 | -- | -- | -- | -- | -- |
| *Brookesia minima* | -- | HQ130511 | -- | FJ984280 | -- | JN674023 | FJ981767 | -- | -- | -- | FJ984209 | -- |
| *Brookesia nasus* | -- | HQ130509 | JQ073088 | FJ984276 | -- | AF448775 | FJ981764 | -- | -- | -- | FJ984205 | -- |
| *Brookesia perarmata* | -- | -- | -- | FJ984309 | -- | AF448776 | FJ981803 | -- | -- | -- | FJ984239 | -- |
| *Brookesia peyrierasi* | AF215137 | AF121954 | -- | FJ984287 | -- | AF448777 | FJ981776 | -- | -- | -- | FJ984216 | -- |
| *Brookesia stumpffi* | -- | -- | -- | FJ984318 | -- | FJ975202 | FJ981812 | -- | -- | -- | FJ984247 | -- |
| *Brookesia superciliaris* | GQ921686 | HQ130513 | -- | FJ984303 | EF222187 | AF448778 | FJ981796 | -- | GQ921702 | -- | FJ984233 | -- |
| *Brookesia therezieni* | GQ921673 | -- | -- | FJ984301 | -- | AF448779 | FJ981793 | -- | GQ921716 | -- | AY662577 | -- |
| *Brookesia thieli* | -- | -- | JQ073089 | FJ984294 | -- | AF448780 | FJ981785 | -- | -- | -- | AY662577 | -- |
| *Brookesia tuberculata* | -- | JN674056 | -- | FJ984281 | -- | AF448781 | FJ981769 | -- | -- | -- | FJ984210 | -- |
| *Brookesia vadoni* | -- | -- | -- | FJ984297 | -- | FJ975182 | FJ981788 | -- | -- | -- | FJ984227 | -- |
| *Brookesia valerieae* | -- | -- | -- | -- | -- | -- | AF443243 | -- | -- | -- | -- | -- |
| *Bufoniceps laungwalaensis* | -- | -- | -- | -- | -- | DQ008214 | -- | -- | -- | -- | -- | -- |
| *Buhoma depressiceps* | FJ404147 | AY611860 | -- | AY611951 | AY612042 | -- | -- | -- | -- | -- | -- | FJ404422 |
| *Buhoma procterae* | FJ404148 | AY611818 | -- | DQ486177 | DQ486353 | -- | FJ404350 | -- | -- | -- | -- | FJ404423 |
| *Bungarus bungaroides* | -- | -- | -- | -- | AY973270 | -- | -- | -- | -- | -- | -- | -- |
| *Bungarus caeruleus* | -- | -- | -- | -- | AJ749305 | -- | AJ830220 | -- | -- | -- | -- | -- |
| *Bungarus candidus* | JN687932 | JN687933 | -- | -- | AJ749343 | JN123449 | AJ830255 | -- | -- | -- | -- | -- |
| *Bungarus ceylonicus* | KC347316 | KC347350 | -- | KC347390 | KC347466 | -- | KC347504 | -- | -- | -- | KC347428 | -- |
| *Bungarus fasciatus* | NC_011393 | JF357944 | FJ433989 | AF544732 | AJ749350 | NC_011393 | U49297 | FJ434090 | -- | EF144073 | EU366438 | EF144100 |
| *Bungarus flaviceps* | -- | -- | -- | -- | AJ749351 | -- | AJ830251 | -- | -- | -- | -- | -- |
| *Bungarus multicinctus* | EU579522 | EF520682 | -- | AF435021 | AJ749345 | NC_011392 | NC 011392 | -- | -- | -- | -- | -- |
| *Bungarus niger* | -- | -- | -- | -- | AJ749304 | -- | AJ830241 | -- | -- | -- | -- | -- |
| *Bungarus sindanus* | -- | -- | -- | -- | AJ749346 | -- | AJ830242 | -- | -- | -- | -- | -- |
| *Bunopus crassicauda* | EU589154 | -- | -- | -- | EU589177 | -- | -- | -- | -- | -- | -- | -- |
| *Bunopus tuberculatus* | EU589160 | -- | HQ443613 | AF148706 | EU589181 | HQ443541 | -- | -- | JQ945355 | -- | JQ945287 | JQ945427 |
| *Cacophis squamulosus* | EU547101 | EU547150 | -- | EU366451 | -- | -- | EU547007 | -- | -- | -- | EU366440 | -- |
| *Cadea blanoides* | EU203661 | EU203661 | EU203612 | EU203613 | -- | -- | -- | EU203629 | -- | EU203631 | EU203662 | EU203635 |
| *Caimanops amphiboluroides* | -- | -- | DQ340704 | DQ340663 | -- | AF128472 | -- | -- | -- | -- | -- | -- |
| *Calabaria reinhardtii* | Z46464 | Z46494 | AY988041 | AY099978 | AY099985 | -- | AF302943 | EU390911 | -- | HQ876359 | EU402839 | FJ433902 |
| *Calamaria pavimentata* | -- | -- | FJ434005 | AF471103 | AF471081 | -- | -- | FJ434106 | -- | EF144089 | EF144092 | EF144116 |
| *Calamaria yunnanensis* | JQ598801 | JQ598863 | -- | -- | JQ598922 | -- | -- | -- | -- | -- | -- | -- |
| *Calamodontophis paucidens* | GQ457786 | GQ457728 | -- | GQ457848 | -- | -- | -- | -- | -- | -- | -- | -- |
| *Caledoniscincus aquilonius* | -- | -- | -- | DQ675399 | -- | DQ675194 | -- | -- | -- | -- | DQ675274 | -- |
| *Caledoniscincus atropunctatus* | -- | -- | -- | DQ675401 | -- | DQ675196 | -- | -- | -- | -- | DQ675276 | -- |
| *Caledoniscincus auratus* | -- | -- | -- | DQ675402 | -- | DQ675197 | -- | -- | -- | -- | DQ675277 | -- |
| *Caledoniscincus austrocaledonicus* | EU567931 | EU567922 | HQ655163 | DQ675404 | EU567835 | EU567727 | EU567766 | -- | -- | -- | EU568024 | -- |
| *Caledoniscincus chazeaui* | -- | -- | -- | -- | -- | DQ675272 | -- | -- | -- | -- | -- | -- |
| *Caledoniscincus festivus* | -- | -- | -- | DQ675405 | -- | DQ675200 | -- | -- | -- | -- | DQ675280 | -- |
| *Caledoniscincus haplorhinus* | -- | -- | -- | DQ675406 | -- | DQ675201 | -- | -- | -- | -- | DQ675281 | -- |
| *Caledoniscincus orestes* | -- | -- | -- | DQ675407 | -- | DQ675202 | -- | -- | -- | -- | DQ675282 | -- |
| *Caledoniscincus renevieri* | -- | -- | -- | -- | -- | DQ675268 | -- | -- | -- | -- | DQ675348 | -- |
| *Caledoniscincus terma* | -- | -- | -- | -- | -- | DQ675271 | -- | -- | -- | -- | -- | -- |
| *Calliophis bivirgata* | U96800 | -- | -- | AY058934 | AF217812 | AY059007 | AY058979 | -- | -- | -- | -- | -- |
| *Calliophis melanurus* | KC347317 | KC347351 | -- | KC347391 | KC347467 | -- | KC347505 | -- | -- | -- | KC347429 | -- |
| *Callisaurus draconoides* | AF194251 | L41441 | -- | AF315401 | AY141104 | EU543789 | AY141061 | -- | -- | -- | -- | -- |
| *Callopistes flavipunctatus* | EF029873 | EF029880 | -- | -- | -- | -- | -- | -- | -- | -- | -- | -- |
| *Callopistes maculatus* | EF029875 | EF029882 | JN654796 | -- | -- | -- | -- | JN568318 | -- | JN568490 | JN654857 | -- |
| *Calloselasma rhodostoma* | AY352779 | AY352718 | -- | -- | AY223562 | -- | AY352813 | -- | -- | -- | -- | -- |
| *Calodactylodes aureus* | DQ852697 | -- | -- | AY172921 | -- | -- | -- | -- | -- | -- | -- | -- |
| *Calodactylodes illingworthorum* | -- | -- | -- | JQ945536 | -- | JX041318 | -- | -- | JQ945356 | -- | JQ945288 | JQ945428 |
| *Calotes calotes* | -- | -- | -- | -- | AB263941 | AF128482 | -- | -- | -- | -- | AY662584 | -- |
| *Calotes ceylonensis* | -- | -- | -- | -- | -- | AF128483 | -- | -- | -- | -- | -- | -- |
| *Calotes chincollium* | -- | -- | -- | -- | -- | DQ289459 | -- | -- | -- | -- | -- | -- |
| *Calotes emma* | -- | -- | JF806005 | -- | AB263942 | DQ289460 | -- | JF804535 | -- | JF804581 | JF806189 | -- |
| *Calotes htunwini* | -- | -- | -- | -- | -- | DQ289464 | -- | -- | -- | -- | -- | -- |
| *Calotes irawadi* | -- | -- | -- | -- | -- | DQ289468 | -- | -- | -- | -- | -- | -- |
| *Calotes liocephalus* | -- | -- | -- | -- | -- | AF128484 | -- | -- | -- | -- | -- | -- |
| *Calotes liolepis* | -- | -- | -- | -- | -- | AF128485 | -- | -- | -- | -- | -- | -- |
| *Calotes mystaceus* | -- | -- | -- | -- | -- | AF128487 | -- | -- | -- | -- | -- | -- |
| *Calotes nigrilabris* | -- | -- | -- | -- | -- | AF128486 | -- | -- | -- | -- | -- | -- |
| *Calotes versicolor* | AB183287 | AB031981 | DQ340705 | AF137525 | AY572870 | DQ289478 | NC_009683 | -- | -- | -- | -- | -- |
| *Calumma boettgeri* | -- | -- | -- | -- | -- | AF448733 | -- | -- | -- | -- | -- | -- |
| *Calumma brevicorne* | -- | -- | -- | FJ984261 | -- | AF448734 | AF443255 | -- | -- | -- | AY662579 | -- |
| *Calumma capuroni* | -- | HQ130521 | -- | HQ130543 | -- | HQ130645 | HQ130567 | -- | -- | -- | HQ130630 | -- |
| *Calumma crypticum* | -- | HQ130527 | -- | -- | -- | HQ130651 | HQ130573 | -- | -- | -- | HQ130636 | -- |
| *Calumma cucullatum* | -- | HQ130523 | -- | HQ130545 | -- | AF448735 | AF443256 | -- | -- | -- | HQ130632 | -- |
| *Calumma fallax* | -- | -- | -- | -- | -- | JN030486 | AF443258 | -- | -- | -- | -- | -- |
| *Calumma furcifer* | -- | HQ130524 | -- | HQ130546 | -- | AF448736 | HQ130570 | -- | -- | -- | HQ130633 | -- |
| *Calumma gallus* | -- | -- | -- | -- | -- | JN030487 | -- | -- | -- | -- | -- | -- |
| *Calumma gastrotaenia* | -- | HQ130525 | -- | FJ984263 | -- | AF448737 | AF443262 | -- | -- | -- | FJ984191 | -- |
| *Calumma globifer* | -- | -- | -- | -- | -- | AF448738 | -- | -- | -- | -- | -- | -- |
| *Calumma guibei* | -- | -- | -- | -- | -- | -- | AF443259 | -- | -- | -- | -- | -- |
| *Calumma hilleniusi* | AF215135 | AF215263 | -- | -- | -- | AF448739 | AF443250 | -- | -- | -- | -- | -- |
| *Calumma malthe* | -- | HQ130526 | -- | HQ130548 | -- | HQ130650 | AF443257 | -- | -- | -- | HQ130635 | -- |
| *Calumma nasutum* | -- | HQ130528 | -- | JN030463 | -- | AF448740 | AF443254 | -- | -- | -- | HQ130637 | -- |
| *Calumma oshaughnessyi* | -- | -- | -- | -- | -- | AF448741 | AF443252 | -- | -- | -- | -- | -- |
| *Calumma parsonii* | AB474915 | AB474915 | -- | FJ984262 | -- | AF448742 | AF443253 | -- | -- | -- | -- | -- |
| *Calumma tsaratananense* | -- | -- | -- | -- | -- | -- | AF443260 | -- | -- | -- | -- | -- |
| *Calyptommatus confusionibus* | HM015561 | HM015583 | -- | -- | -- | -- | -- | -- | -- | -- | -- | -- |
| *Calyptommatus leiolepis* | AF420683 | HM015582 | -- | AF420858 | -- | -- | AF420874 | -- | -- | -- | -- | -- |
| *Calyptommatus nicterus* | AF420684 | AF420747 | -- | AF420822 | -- | -- | AF420903 | -- | -- | -- | -- | -- |
| *Calyptommatus sinebrachiatus* | AF420685 | AF420720 | -- | AF420832 | -- | -- | AF420873 | -- | -- | -- | -- | -- |
| *Calyptotis lepidorostrum* | DQ915293 | DQ915317 | -- | -- | -- | -- | DQ915341 | -- | -- | -- | -- | -- |
| *Calyptotis ruficauda* | DQ915303 | DQ915327 | -- | -- | -- | -- | DQ915351 | -- | -- | -- | -- | -- |
| *Calyptotis scutirostrum* | AY169577 | AY308181 | -- | -- | AF373235 | AF373265 | AY169652 | -- | -- | -- | -- | -- |
| *Candoia aspera* | EF545041 | EF545068 | -- | -- | U69751 | -- | -- | -- | -- | -- | -- | -- |
| *Candoia bibroni* | -- | -- | -- | -- | AF153077 | -- | -- | -- | -- | -- | -- | -- |
| *Candoia carinata* | AF544741 | EU419850 | FJ433974 | AY099961 | AY099984 | -- | -- | FJ434077 | -- | FJ433928 | AY988065 | FJ433904 |
| *Cantoria violacea* | EF395873 | EF395848 | -- | EF395922 | EF395897 | -- | -- | -- | -- | -- | -- | -- |
| *Carinatogecko heteropholis* | GQ354886 | -- | -- | -- | HM140839 | -- | -- | -- | -- | -- | -- | -- |
| *Carlia amax* | -- | FJ379465 | -- | -- | -- | -- | AJ290505 | -- | -- | -- | -- | -- |
| *Carlia bicarinata* | -- | FJ379466 | -- | -- | -- | -- | AJ290506 | -- | -- | -- | -- | -- |
| *Carlia coensis* | -- | FJ379468 | -- | -- | -- | -- | FJ379452 | -- | -- | -- | -- | -- |
| *Carlia dogare* | -- | FJ379470 | -- | -- | -- | -- | AJ290509 | -- | -- | -- | -- | -- |
| *Carlia fusca* | AY308331 | AY308182 | -- | -- | -- | -- | FJ379453 | -- | -- | -- | -- | -- |
| *Carlia gracilis* | -- | FJ379474 | -- | -- | -- | -- | AJ290512 | -- | -- | -- | -- | -- |
| *Carlia jarnoldae* | -- | FJ379478 | -- | -- | -- | -- | AJ290515 | -- | -- | -- | -- | -- |
| *Carlia johnstonei* | -- | FJ379480 | -- | -- | -- | -- | AJ290517 | -- | -- | -- | -- | -- |
| *Carlia longipes* | -- | FJ379483 | -- | -- | -- | -- | GQ403650 | -- | -- | -- | -- | -- |
| *Carlia munda* | -- | FJ379485 | -- | -- | -- | -- | AJ290521 | -- | -- | -- | -- | -- |
| *Carlia mundivensis* | -- | FJ379486 | -- | -- | -- | -- | AJ290522 | -- | -- | -- | -- | -- |
| *Carlia mysi* | -- | FJ379471 | -- | -- | -- | -- | -- | -- | -- | -- | -- | -- |
| *Carlia pectoralis* | -- | FJ379490 | -- | -- | -- | -- | FJ379454 | -- | -- | -- | -- | -- |
| *Carlia rhomboidalis* | -- | FJ379492 | -- | -- | AJ406216 | -- | AJ290528 | -- | -- | -- | -- | -- |
| *Carlia rostralis* | -- | FJ379494 | -- | -- | -- | -- | GQ403643 | -- | -- | -- | -- | -- |
| *Carlia rubrigularis* | -- | FJ379497 | -- | AY818787 | AF181068 | -- | AJ290533 | -- | -- | -- | -- | -- |
| *Carlia rufilatus* | -- | FJ379500 | -- | -- | -- | -- | FJ379456 | -- | -- | -- | -- | -- |
| *Carlia schmeltzii* | -- | FJ379501 | -- | -- | -- | -- | GQ403644 | -- | -- | -- | -- | -- |
| *Carlia scirtetis* | -- | FJ379502 | -- | -- | -- | -- | FJ379457 | -- | -- | -- | -- | -- |
| *Carlia storri* | -- | FJ379505 | -- | -- | -- | -- | FJ379458 | -- | -- | -- | -- | -- |
| *Carlia tetradactyla* | -- | FJ379507 | -- | -- | -- | -- | AJ290541 | -- | -- | -- | -- | -- |
| *Carlia triacantha* | -- | FJ379509 | -- | -- | -- | -- | AJ290543 | -- | -- | -- | -- | -- |
| *Carlia vivax* | -- | FJ379511 | -- | -- | -- | -- | FJ379460 | -- | -- | -- | -- | -- |
| *Carphodactylus laevis* | AF090175 | GU460142 | -- | EF534905 | AF109565 | AY369017 | -- | -- | GU459744 | -- | EF534781 | EF534947 |
| *Carphophis amoenus* | AY577013 | AY577022 | -- | DQ112082 | AF471067 | -- | -- | -- | -- | -- | -- | -- |
| *Casarea dussumieri* | AF544754 | AF544827 | EU402632 | AF471114 | U69755 | -- | -- | EU390912 | -- | FJ433918 | AY487408 | FJ433894 |
| *Causus defilippii* | AF057186 | AF057233 | EU402633 | -- | AY223556 | GU045452 | AY223617 | EU390913 | -- | JN703088 | EU402840 | -- |
| *Causus resimus* | AF544763 | AY223662 | -- | AF544696 | AY223555 | -- | AY223616 | -- | -- | -- | -- | -- |
| *Causus rhombeatus* | DQ305409 | DQ305432 | -- | -- | DQ305455 | -- | DQ305473 | -- | -- | -- | -- | -- |
| *Cautula zia* | -- | -- | -- | -- | -- | DQ675264 | AY626666 | -- | -- | -- | DQ675344 | -- |
| *Celatiscincus euryotis* | -- | -- | -- | DQ675409 | -- | DQ675204 | -- | -- | -- | -- | DQ675284 | -- |
| *Celatiscincus similis* | -- | -- | -- | DQ675408 | -- | DQ675203 | -- | -- | -- | -- | DQ675283 | -- |
| *Celestus agasepsoides* | -- | -- | -- | -- | -- | AF085611 | -- | -- | -- | -- | -- | -- |
| *Celestus enneagrammus* | -- | -- | GU457853 | -- | -- | AF085607 | -- | GU456009 | -- | JN703087 | AY662604 | -- |
| *Celestus haetianus* | -- | -- | -- | -- | -- | AF085612 | -- | -- | -- | -- | -- | -- |
| *Cemophora coccinea* | FJ623960 | -- | -- | AF471132 | AF471091 | DQ902249 | DQ902282 | -- | -- | -- | -- | -- |
| *Cerastes cerastes* | EU852317 | HQ267801 | -- | AF471131 | AF471028 | -- | EU852305 | -- | -- | -- | EU852329 | -- |
| *Cerastes gasperettii* | JN870181 | HQ658451 | -- | -- | -- | -- | -- | -- | -- | -- | -- | -- |
| *Cerastes vipera* | -- | AJ275757 | -- | -- | AJ275705 | -- | -- | -- | -- | -- | -- | -- |
| *Ceratophora aspera* | -- | -- | -- | -- | -- | AF128491 | -- | -- | -- | -- | -- | -- |
| *Ceratophora erdeleni* | -- | -- | -- | -- | -- | AF128522 | -- | -- | -- | -- | -- | -- |
| *Ceratophora karu* | -- | -- | -- | -- | -- | AF128520 | -- | -- | -- | -- | -- | -- |
| *Ceratophora stoddartii* | -- | -- | -- | -- | -- | AF364054 | -- | -- | -- | -- | -- | -- |
| *Cerberus australis* | EF395874 | EF395849 | -- | EF395923 | EF395898 | -- | -- | -- | -- | -- | -- | -- |
| *Cerberus microlepis* | EF395875 | EF395850 | -- | EF395924 | EF395899 | -- | -- | -- | -- | -- | -- | -- |
| *Cerberus rynchops* | AF499289 | EF395852 | -- | EF395926 | EF395901 | -- | U49327 | -- | -- | -- | EU366441 | -- |
| *Cercaspis carinatus* | -- | KC347352 | -- | KC347392 | KC347468 | -- | -- | -- | -- | -- | KC347430 | -- |
| *Cercolophia cuiabana* | -- | FJ441696 | FJ441879 | FJ441756 | -- | FJ441939 | -- | -- | -- | -- | FJ441816 | -- |
| *Cercolophia roberti* | -- | FJ441711 | FJ441894 | FJ441771 | -- | FJ441954 | -- | -- | -- | -- | FJ441831 | -- |
| *Cercosaura argulus* | -- | -- | -- | AF420838 | -- | -- | AF420896 | -- | -- | -- | -- | -- |
| *Cercosaura eigenmanni* | -- | -- | -- | AF420828 | -- | -- | AF420895 | -- | -- | -- | -- | -- |
| *Cercosaura ocellata* | AF420677 | AF420731 | -- | AF420834 | -- | -- | AF420883 | -- | -- | -- | -- | -- |
| *Cercosaura oshaughnessyi* | -- | -- | -- | AF420852 | -- | -- | AF420893 | -- | -- | -- | -- | -- |
| *Cercosaura quadrilineata* | -- | -- | -- | AF420830 | -- | -- | AF420880 | -- | -- | -- | -- | -- |
| *Cercosaura schreibersii* | -- | -- | -- | AF420856 | -- | -- | AF420882 | -- | -- | -- | -- | -- |
| *Cerrophidion barbouri* | HM363639 | HM363640 | -- | -- | HM363641 | -- | HM363642 | -- | -- | -- | -- | -- |
| *Cerrophidion godmani* | AF057203 | DQ305442 | -- | -- | DQ061201 | -- | AY220351 | -- | -- | -- | -- | -- |
| *Cerrophidion petlalcalensis* | DQ305420 | DQ305443 | -- | -- | DQ061202 | -- | DQ061227 | -- | -- | -- | -- | -- |
| *Cerrophidion tzotzilorum* | JN870182 | JN870193 | -- | -- | DQ061204 | -- | DQ061228 | -- | -- | -- | -- | -- |
| *Chalarodon madagascariensis* | AB266748 | EU099714 | AY987972 | AY987987 | AB266748 | AF528722 | AB266748 | JF804536 | -- | JF804582 | FJ356745 | -- |
| *Chalcides bedriagai* | EU277909 | EU278041 | -- | -- | EU278148 | -- | -- | -- | -- | -- | -- | -- |
| *Chalcides boulengeri* | EU277924 | EU278045 | -- | -- | EU278156 | -- | -- | -- | -- | -- | -- | -- |
| *Chalcides chalcides* | AJ416936 | AY649145 | -- | -- | EU278211 | -- | -- | -- | -- | -- | -- | -- |
| *Chalcides coeruleopunctatus* | EU277891 | EU278038 | -- | -- | EU278123 | -- | -- | -- | -- | -- | -- | -- |
| *Chalcides colosii* | EU277930 | EU278049 | -- | -- | EU278161 | -- | -- | -- | -- | -- | -- | -- |
| *Chalcides guentheri* | EU278001 | -- | -- | -- | EU278233 | -- | -- | -- | -- | -- | -- | -- |
| *Chalcides lanzai* | EU277920 | EU278042 | -- | -- | EU278152 | -- | -- | -- | -- | -- | -- | -- |
| *Chalcides manueli* | EU277856 | EU278023 | -- | -- | EU278089 | -- | -- | -- | -- | -- | -- | -- |
| *Chalcides mauritanicus* | EU277970 | EU278060 | -- | -- | EU278203 | -- | -- | -- | -- | -- | -- | -- |
| *Chalcides minutus* | EU277974 | EU278063 | -- | -- | EU278205 | -- | -- | -- | -- | -- | -- | -- |
| *Chalcides mionecton* | FJ980116 | AY649146 | -- | -- | EU278099 | -- | -- | -- | -- | -- | -- | -- |
| *Chalcides montanus* | FJ980114 | -- | -- | -- | EU278090 | -- | -- | -- | -- | -- | -- | -- |
| *Chalcides ocellatus* | FJ980091 | AY649147 | HM160584 | AY818798 | FJ972228 | AY662557 | -- | -- | -- | HM161058 | AY662638 | -- |
| *Chalcides parallelus* | EU277921 | EU278043 | -- | -- | EU278153 | -- | -- | -- | -- | -- | -- | -- |
| *Chalcides polylepis* | EU277861 | AY649148 | -- | -- | AF054556 | -- | -- | -- | -- | -- | -- | -- |
| *Chalcides pseudostriatus* | EU277985 | EU278066 | -- | -- | EU278218 | -- | -- | -- | -- | -- | -- | -- |
| *Chalcides sepsoides* | EU277925 | EU278047 | -- | -- | EU278158 | -- | -- | -- | -- | -- | -- | -- |
| *Chalcides sexlineatus* | AF054532 | AF054547 | JQ073128 | -- | AF054561 | -- | -- | -- | -- | -- | JQ073251 | -- |
| *Chalcides sphenopsiformis* | EU277878 | EU278032 | -- | -- | EU278110 | -- | -- | -- | -- | -- | -- | -- |
| *Chalcides striatus* | EU277987 | EU278068 | -- | AY234232 | EU278232 | -- | -- | -- | -- | -- | -- | -- |
| *Chalcides viridanus* | AF232630 | EU278036 | JQ073129 | -- | AF054566 | -- | -- | -- | -- | -- | JQ073252 | -- |
| *Chamaeleo affinis* | -- | FJ717757 | -- | HQ130542 | -- | FJ717788 | HQ130566 | -- | -- | -- | FJ746595 | -- |
| *Chamaeleo africanus* | NC_012422 | DQ397217 | -- | -- | NC_012422 | AF448743 | NC_012422 | -- | -- | -- | -- | -- |
| *Chamaeleo arabicus* | NC_012445 | NC_012445 | -- | -- | NC_012445 | -- | NC_012445 | -- | -- | -- | -- | -- |
| *Chamaeleo balebicornutus* | -- | FJ717758 | -- | -- | -- | FJ717789 | -- | -- | -- | -- | FJ746596 | -- |
| *Chamaeleo bitaeniatus* | DQ397276 | FJ717760 | -- | -- | -- | -- | -- | -- | -- | -- | FJ746597 | -- |
| *Chamaeleo calcaricarens* | NC_012452 | NC_012452 | -- | -- | NC_012452 | -- | NC_012452 | -- | -- | -- | -- | -- |
| *Chamaeleo calyptratus* | NC_012420 | NC_012420 | GU457847 | -- | NC_012420 | AF448744 | AF443227 | GU456003 | -- | HQ876323 | HQ876433 | -- |
| *Chamaeleo chamaeleon* | NC_012427 | HQ130518 | -- | -- | NC_012427 | AF448745 | AF443228 | -- | -- | -- | -- | -- |
| *Chamaeleo cristatus* | -- | -- | -- | -- | -- | AF448746 | -- | -- | -- | -- | -- | -- |
| *Chamaeleo deremensis* | DQ397273 | FJ717762 | -- | -- | -- | AF448747 | -- | -- | -- | -- | FJ746599 | -- |
| *Chamaeleo dilepis* | NC_012436 | HQ130517 | -- | -- | NC_012436 | AY289916 | NC_012436 | -- | -- | -- | DQ996655 | -- |
| *Chamaeleo ellioti* | DQ397239 | FJ717766 | -- | -- | -- | AF448748 | -- | -- | -- | -- | FJ746601 | -- |
| *Chamaeleo feae* | -- | FJ717767 | -- | HQ130541 | -- | AF448749 | HQ130565 | -- | -- | -- | -- | -- |
| *Chamaeleo fuelleborni* | -- | -- | -- | -- | -- | AF448750 | -- | -- | -- | -- | -- | -- |
| *Chamaeleo goetzei* | -- | FJ717770 | -- | -- | -- | FJ717791 | -- | -- | -- | -- | FJ746603 | -- |
| *Chamaeleo gracilis* | AY927255 | FJ717748 | -- | -- | -- | AF448751 | -- | -- | -- | -- | DQ996658 | -- |
| *Chamaeleo harennae* | -- | FJ717772 | -- | -- | -- | FJ717790 | -- | -- | -- | -- | FJ746606 | -- |
| *Chamaeleo hoehnelii* | DQ397238 | FJ717773 | -- | -- | -- | AF448752 | -- | -- | -- | -- | FJ746607 | -- |
| *Chamaeleo jacksonii* | DQ397240 | DQ397226 | AY987977 | FJ984258 | -- | AF448753 | AF443229 | -- | -- | -- | AY988023 | -- |
| *Chamaeleo johnstoni* | DQ397275 | FJ717776 | -- | -- | -- | AF448754 | -- | -- | -- | -- | DQ996650 | -- |
| *Chamaeleo laevigatus* | DQ397210 | DQ397212 | -- | -- | -- | -- | -- | -- | -- | -- | -- | -- |
| *Chamaeleo melleri* | NC_014176 | FJ717777 | -- | JN090149 | NC_014176 | AF448755 | AF443232 | -- | -- | -- | DQ996652 | -- |
| *Chamaeleo monachus* | NC_012443 | NC_012443 | -- | -- | NC_012443 | -- | NC_012443 | -- | -- | -- | -- | -- |
| *Chamaeleo montium* | -- | -- | -- | -- | -- | AF448756 | -- | -- | -- | -- | -- | -- |
| *Chamaeleo namaquensis* | -- | HQ130516 | -- | FJ984256 | -- | AF448757 | FJ981754 | -- | -- | -- | -- | -- |
| *Chamaeleo narraioca* | DQ397268 | DQ397298 | -- | -- | -- | -- | -- | -- | -- | -- | -- | -- |
| *Chamaeleo necasi* | DQ397211 | DQ397213 | -- | -- | -- | -- | -- | -- | -- | -- | -- | -- |
| *Chamaeleo oweni* | HQ337864 | HQ337816 | -- | -- | -- | -- | -- | -- | -- | -- | HQ130629 | -- |
| *Chamaeleo pfefferi* | -- | -- | -- | -- | -- | AF448758 | -- | -- | -- | -- | -- | -- |
| *Chamaeleo quadricornis* | -- | AF121959 | -- | FJ984259 | -- | AF448759 | AF443230 | -- | -- | -- | -- | -- |
| *Chamaeleo quilensis* | -- | FJ717749 | -- | -- | -- | AF448760 | -- | -- | -- | -- | DQ996657 | -- |
| *Chamaeleo roperi* | AY927240 | FJ717750 | -- | -- | -- | -- | -- | -- | -- | -- | -- | -- |
| *Chamaeleo rudis* | DQ397277 | DQ923811 | -- | -- | -- | AF448761 | -- | -- | -- | -- | AY662578 | -- |
| *Chamaeleo schubotzi* | -- | FJ717783 | -- | -- | -- | FJ717806 | -- | -- | -- | -- | FJ746618 | -- |
| *Chamaeleo senegalensis* | -- | FJ717752 | -- | -- | -- | -- | AF443231 | -- | -- | -- | AY487370 | -- |
| *Chamaeleo sternfeldi* | AM422444 | FJ717780 | -- | -- | -- | AF448762 | -- | -- | -- | -- | FJ746615 | -- |
| *Chamaeleo tempeli* | -- | FJ717786 | -- | -- | -- | -- | -- | -- | -- | -- | FJ746619 | -- |
| *Chamaeleo werneri* | DQ397274 | AJ609596 | -- | -- | -- | -- | -- | -- | -- | -- | -- | -- |
| *Chamaeleo wiedersheimi* | -- | -- | -- | -- | -- | AF448763 | -- | -- | -- | -- | -- | -- |
| *Chamaeleo zeylanicus* | NC_012444 | NC_012444 | -- | -- | NC_012444 | -- | NC_012444 | -- | -- | -- | -- | -- |
| *Chamaesaura aenea* | HQ167051 | HQ167162 | -- | -- | -- | HQ166952 | -- | -- | -- | -- | -- | -- |
| *Chamaesaura anguina* | HQ167053 | HQ167165 | -- | -- | -- | HQ166955 | -- | -- | -- | -- | -- | -- |
| *Charina bottae* | AF544743 | -- | AY988042 | AY099971 | U69757 | -- | AF302976 | FJ434079 | -- | FJ433932 | AY988076 | FJ433908 |
| *Chelosania brunnea* | -- | -- | DQ340707 | DQ340664 | -- | AF128465 | -- | JF804537 | -- | JF804583 | JF806190 | -- |
| *Chilomeniscus stramineus* | -- | -- | -- | GQ895800 | GQ895856 | -- | U49305 | -- | -- | -- | -- | -- |
| *Chionactis occipitalis* | -- | -- | -- | GQ895801 | GQ895857 | -- | -- | -- | -- | -- | -- | -- |
| *Chioninia delalandii* | AF280185 | AY151482 | -- | AF335081 | AF335044 | -- | -- | -- | -- | -- | -- | HQ316448 |
| *Chioninia fogoensis* | AF335027 | AY151480 | -- | AF335084 | AF280312 | -- | -- | -- | -- | -- | -- | HQ316461 |
| *Chioninia spinalis* | AF335069 | AY151478 | -- | AF335087 | AF335072 | -- | -- | -- | -- | -- | -- | HQ316475 |
| *Chioninia stangeri* | AF280162 | AY151479 | -- | AF335086 | AF335078 | -- | -- | -- | -- | -- | -- | HQ316455 |
| *Chioninia vaillantii* | AF280201 | AY151483 | -- | AF335088 | AF335076 | -- | -- | -- | -- | -- | -- | -- |
| *Chirindia swynnertoni* | -- | -- | -- | AY444010 | -- | -- | -- | -- | -- | -- | AY444037 | -- |
| *Chironius bicarinatus* | HM565744 | -- | -- | -- | -- | -- | -- | -- | -- | -- | -- | -- |
| *Chironius carinatus* | HM565745 | -- | -- | HQ529281 | HQ529280 | -- | -- | -- | -- | -- | -- | -- |
| *Chironius exoletus* | HM565746 | -- | -- | -- | -- | -- | -- | -- | -- | -- | -- | -- |
| *Chironius flavolineatus* | HM565747 | -- | -- | -- | -- | -- | -- | -- | -- | -- | -- | -- |
| *Chironius fuscus* | HM565749 | -- | -- | -- | -- | -- | -- | -- | -- | -- | -- | -- |
| *Chironius grandisquamis* | HM565750 | -- | -- | -- | -- | -- | -- | -- | -- | -- | -- | -- |
| *Chironius laevicollis* | HM565751 | -- | -- | -- | -- | -- | -- | -- | -- | -- | -- | -- |
| *Chironius laurenti* | HM565752 | -- | -- | -- | -- | -- | -- | -- | -- | -- | -- | -- |
| *Chironius monticola* | HM565753 | -- | -- | -- | -- | -- | -- | -- | -- | -- | -- | -- |
| *Chironius multiventris* | HM565754 | -- | -- | -- | -- | -- | -- | -- | -- | -- | -- | -- |
| *Chironius quadricarinatus* | HM565755 | -- | -- | -- | -- | -- | -- | -- | -- | -- | -- | -- |
| *Chironius scurrulus* | HM565756 | -- | -- | -- | -- | -- | -- | -- | -- | -- | -- | -- |
| *Chlamydosaurus kingii* | EF090423 | EF090423 | DQ340708 | DQ340665 | NC_009421 | EF090489 | EF090423 | JF804538 | -- | JF804584 | JF806191 | -- |
| *Chondrodactylus angulifer* | DQ275403 | AY123372 | JQ073121 | JQ945537 | AY123394 | JX041320 | -- | -- | JQ945357 | -- | JQ073242 | JQ945429 |
| *Chondrodactylus bibronii* | -- | -- | -- | EU293690 | -- | -- | -- | -- | EU293712 | -- | EU293645 | EU293735 |
| *Chondrodactylus fitzsimonsi* | DQ275404 | AF449109 | -- | -- | AF449125 | JN393945 | -- | -- | -- | -- | DQ275448 | -- |
| *Chondrodactylus turneri* | DQ852708 | AF449108 | -- | AY172938 | AF449124 | -- | -- | -- | -- | -- | -- | -- |
| *Christinus marmoratus* | DQ852698 | -- | -- | FJ855461 | -- | JX041322 | -- | -- | JQ945358 | -- | FJ855440 | JQ945430 |
| *Chrysopelea ornata* | KC347318 | KC347353 | -- | KC347393 | KC347469 | -- | KC347506 | -- | -- | -- | KC347431 | -- |
| *Chrysopelea paradisi* | -- | -- | -- | GQ895802 | GQ895858 | -- | -- | -- | -- | -- | -- | -- |
| *Chrysopelea taprobanica* | KC347319 | KC347354 | -- | KC347394 | KC347470 | -- | KC347507 | -- | -- | -- | KC347432 | -- |
| *Clelia bicolor* | GQ457787 | GQ457729 | -- | GQ457849 | -- | -- | -- | -- | -- | -- | -- | -- |
| *Clelia clelia* | AF158403 | AF158472 | -- | JQ598973 | -- | -- | -- | -- | -- | -- | -- | -- |
| *Clelia rustica* | JQ598802 | JQ598864 | -- | JQ598974 | JQ598923 | -- | -- | -- | -- | -- | -- | -- |
| *Clonophis kirtlandii* | AF402625 | -- | -- | -- | AF402908 | AF384827 | -- | -- | -- | -- | -- | -- |
| *Cnemaspis africana* | -- | -- | -- | JQ945539 | -- | JX041323 | -- | -- | JQ945359 | -- | JQ945291 | JQ945431 |
| *Cnemaspis dickersoni* | -- | -- | -- | JQ945540 | -- | JX041324 | -- | -- | JQ945360 | -- | JQ945292 | JQ945432 |
| *Cnemaspis kandiana* | -- | -- | -- | JQ945541 | -- | JX041325 | -- | -- | JQ945361 | -- | JQ945293 | JQ945433 |
| *Cnemaspis kendallii* | -- | -- | -- | AY172923 | -- | JX041326 | -- | -- | JQ945362 | -- | JQ945294 | JQ945434 |
| *Cnemaspis limi* | -- | -- | -- | EF534935 | -- | JX041327 | -- | -- | EF534851 | -- | EF534809 | EF534977 |
| *Cnemaspis podihuna* | -- | -- | -- | JQ945543 | -- | JX041328 | -- | -- | JQ945363 | -- | JQ945295 | JQ945435 |
| *Cnemaspis tropidogaster* | DQ852716 | -- | -- | DQ852729 | -- | -- | -- | -- | -- | -- | -- | -- |
| *Cnemaspis uzungwae* | -- | -- | -- | JQ945544 | -- | JX041329 | -- | -- | JQ945364 | -- | JQ945296 | JQ945436 |
| *Cnemidophorus arenivagus* | AY046441 | AY046483 | -- | -- | -- | -- | -- | -- | -- | -- | -- | -- |
| *Cnemidophorus gramivagus* | AY046432 | AY046474 | -- | -- | -- | -- | -- | -- | -- | -- | -- | -- |
| *Cnemidophorus lacertoides* | AY046437 | AY046479 | -- | -- | -- | -- | -- | -- | -- | -- | -- | -- |
| *Cnemidophorus lemniscatus* | AY046438 | AY046480 | -- | -- | -- | -- | -- | -- | -- | -- | -- | -- |
| *Cnemidophorus longicaudus* | AY046439 | AY046481 | -- | -- | -- | -- | -- | -- | -- | -- | -- | -- |
| *Cnemidophorus ocellifer* | AF420706 | AY217992 | -- | AF420862 | AY217839 | -- | AF420914 | -- | -- | -- | -- | -- |
| *Cnemidophorus vanzoi* | DQ168985 | DQ168987 | -- | -- | -- | -- | -- | -- | -- | -- | -- | -- |
| *Coelognathus erythrurus* | AY122665 | -- | -- | DQ902067 | DQ902108 | DQ902215 | DQ902288 | -- | -- | -- | -- | -- |
| *Coelognathus flavolineatus* | AY122666 | -- | -- | DQ902090 | DQ902128 | DQ902240 | U49301 | -- | -- | -- | -- | -- |
| *Coelognathus helena* | AY122674 | -- | -- | DQ902071 | DQ902112 | DQ902219 | DQ902292 | -- | -- | -- | -- | -- |
| *Coelognathus radiata* | AY122677 | -- | -- | DQ902079 | DQ902121 | DQ902230 | DQ902317 | -- | -- | -- | -- | -- |
| *Coelognathus subradiata* | AY122673 | -- | -- | DQ902084 | DQ902126 | DQ902235 | DQ902304 | -- | -- | -- | -- | -- |
| *Coeranoscincus frontalis* | DQ915288 | DQ915312 | -- | -- | -- | -- | DQ915336 | -- | -- | -- | -- | -- |
| *Coeranoscincus reticulatus* | AY169578 | AY169615 | -- | -- | -- | -- | AY169653 | -- | -- | -- | -- | -- |
| *Coggeria naufragus* | DQ915297 | DQ915321 | -- | -- | -- | -- | DQ915345 | -- | -- | -- | -- | -- |
| *Coleodactylus amazonicus* | -- | GQ140420 | -- | EU435231 | DQ110596 | JX041319 | DQ110776 | -- | HQ426180 | -- | HQ426267 | HQ426442 |
| *Coleodactylus brachystoma* | -- | DQ104116 | -- | EU435238 | DQ110709 | JX041330 | DQ104175 | -- | EF534833 | -- | EF534792 | EF534959 |
| *Coleodactylus meridionalis* | -- | DQ110514 | -- | EU435247 | DQ110663 | -- | DQ110819 | -- | -- | -- | -- | -- |
| *Coleodactylus natalensis* | -- | DQ104127 | -- | EU435253 | DQ110712 | -- | DQ110868 | -- | -- | -- | -- | -- |
| *Coleodactylus septentrionalis* | -- | DQ110555 | -- | EU435258 | DQ110706 | JX041332 | DQ110823 | -- | EF534832 | -- | EF534791 | EF534958 |
| *Coleonyx brevis* | AB308451 | AB308462 | -- | HQ426528 | -- | AB308464 | -- | -- | HQ426182 | -- | HQ426271 | HQ426444 |
| *Coleonyx elegans* | AB308452 | HM012688 | -- | -- | -- | AB308465 | -- | -- | -- | -- | -- | -- |
| *Coleonyx mitratus* | AB028747 | AB028761 | HQ876230 | HQ426529 | -- | AB308466 | -- | -- | HQ426183 | HQ876370 | HQ426272 | HQ426445 |
| *Coleonyx variegatus* | AB114446 | AY140999 | EU108352 | EF534901 | EU116505 | AB114446 | NC_008774 | EU108018 | EF534817 | HQ876371 | EU108526 | EF534943 |
| *Colobodactylus dalcyanus* | AF420663 | AF420736 | -- | AF420844 | -- | -- | AF420881 | -- | -- | -- | -- | -- |
| *Colobodactylus taunayi* | AF420662 | AF420741 | -- | AF420831 | -- | -- | -- | -- | -- | -- | -- | -- |
| *Colobosaura mentalis* | AF420694 | AF420726 | -- | AF420842 | -- | -- | AF420899 | -- | -- | -- | -- | -- |
| *Colobosaura modesta* | AY218003 | -- | EU108353 | EU116677 | EU116506 | -- | AF420887 | JN568324 | -- | JN568502 | EU108527 | -- |
| *Colobosauroides cearensis* | AF420659 | AF420727 | -- | AF420849 | JN588656 | -- | AF420886 | -- | -- | -- | -- | -- |
| *Coloptychon rhombifer* | AY525105 | -- | -- | -- | AY525102 | -- | -- | -- | -- | -- | -- | -- |
| *Colopus kochii* | -- | -- | -- | JQ945545 | AY123398 | JX041336 | -- | -- | JQ945365 | -- | DQ275418 | JQ945437 |
| *Colopus wahlbergii* | DQ275375 | AY123373 | -- | JQ945546 | AY123395 | JX041337 | -- | -- | JQ945366 | -- | DQ275419 | JQ945438 |
| *Coluber constrictor* | U96794 | L01770 | EU402634 | AY486938 | AY122649 | AY487002 | AY487041 | EU390914 | -- | JN703078 | EU402841 | -- |
| *Coluber dorri* | -- | AY188081 | -- | AY188001 | AY188040 | AY487003 | AY487042 | -- | -- | -- | -- | -- |
| *Coluber zebrinus* | -- | AY188084 | -- | AY188004 | AY188043 | AY487019 | AY487058 | -- | -- | -- | -- | -- |
| *Compsophis albiventris* | FJ404149 | AY188050 | -- | AY187972 | AY188011 | -- | FJ404351 | -- | -- | -- | -- | -- |
| *Compsophis boulengeri* | -- | EF204007 | -- | EF204001 | EF203995 | -- | -- | -- | -- | -- | -- | -- |
| *Compsophis infralineatus* | FJ404157 | EF204006 | -- | EF204000 | EF203994 | -- | FJ404359 | -- | -- | -- | -- | FJ404431 |
| *Compsophis laphystius* | -- | EF204004 | -- | EF203998 | EF203992 | -- | -- | -- | -- | -- | -- | -- |
| *Coniophanes fissidens* | -- | -- | -- | -- | EF078538 | -- | EF078586 | GU353274 | -- | -- | -- | -- |
| *Conolophus pallidus* | -- | -- | -- | HM352531 | FJ535930 | -- | HM352517 | HM352521 | -- | -- | -- | -- |
| *Conolophus subcristatus* | -- | -- | -- | -- | FJ535984 | -- | U66235 | HM352522 | -- | -- | -- | -- |
| *Conophis lineatus* | GQ457788 | GU018161 | JQ599016 | JQ598975 | JQ598924 | -- | -- | -- | -- | -- | -- | -- |
| *Conopsis biserialis* | -- | -- | -- | GQ895804 | GQ895860 | -- | -- | -- | -- | -- | -- | -- |
| *Conopsis nasus* | -- | -- | -- | GQ895805 | GQ895861 | -- | -- | -- | -- | -- | -- | -- |
| *Contia tenuis* | AY577021 | AY577030 | GU112342 | AF471134 | AF471095 | -- | AF402656 | -- | -- | -- | -- | -- |
| *Cophosaurus texanus* | -- | L41442 | -- | -- | AY141099 | AY297489 | AY141062 | -- | -- | -- | -- | -- |
| *Cophotis ceylanica* | -- | -- | -- | -- | -- | AF128493 | -- | -- | -- | -- | -- | -- |
| *Cophotis dumbara* | -- | -- | -- | -- | -- | GQ502785 | -- | -- | -- | -- | -- | -- |
| *Corallus annulatus* | -- | -- | -- | -- | U69763 | -- | -- | -- | -- | -- | -- | -- |
| *Corallus caninus* | -- | -- | JQ073072 | AY987964 | U69768 | -- | -- | -- | -- | -- | JQ073193 | -- |
| *Corallus hortulanus* | -- | -- | -- | HQ399544 | HQ399515 | -- | -- | -- | -- | -- | HQ399525 | -- |
| *Cordylosaurus subtessellatus* | AY218001 | AY217951 | HQ876225 | AY217849 | AY217797 | AY167393 | -- | JF804540 | -- | HQ876344 | HQ876441 | -- |
| *Cordylus aridus* | HQ167059 | HQ167170 | -- | -- | -- | HQ166958 | -- | -- | -- | -- | -- | -- |
| *Cordylus beraduccii* | HQ167061 | HQ167172 | -- | -- | -- | -- | -- | -- | -- | -- | -- | -- |
| *Cordylus campbelli* | HQ167064 | HQ167175 | -- | -- | -- | HQ166962 | -- | -- | -- | -- | -- | -- |
| *Cordylus capensis* | HQ167143 | HQ167255 | -- | AY818770 | -- | HQ167033 | -- | -- | -- | -- | -- | -- |
| *Cordylus cataphractus* | HQ167066 | HQ167177 | -- | -- | AY167391 | AY167408 | -- | -- | -- | -- | -- | -- |
| *Cordylus coeruleopunctatus* | HQ167070 | -- | -- | -- | -- | HQ166969 | -- | -- | -- | -- | -- | -- |
| *Cordylus cordylus* | HQ167077 | HQ167190 | -- | AF148711 | -- | AY519716 | -- | -- | -- | -- | -- | -- |
| *Cordylus giganteus* | HQ167085 | HQ167196 | -- | -- | -- | HQ166984 | -- | -- | -- | DQ119610 | AY487371 | -- |
| *Cordylus imkeae* | HQ167087 | HQ167198 | -- | -- | -- | HQ166986 | -- | -- | -- | -- | -- | -- |
| *Cordylus jonesii* | HQ167088 | HQ167200 | -- | -- | -- | HQ166988 | -- | -- | -- | -- | -- | -- |
| *Cordylus jordani* | HQ167091 | HQ167202 | -- | -- | -- | HQ166990 | -- | -- | -- | -- | -- | -- |
| *Cordylus langi* | HQ167146 | HQ167257 | -- | -- | -- | HQ167035 | -- | -- | -- | -- | -- | -- |
| *Cordylus lawrenci* | HQ167092 | HQ167203 | -- | -- | -- | -- | -- | -- | -- | -- | -- | -- |
| *Cordylus macropholis* | AF236045 | HQ167207 | -- | -- | -- | AY519719 | -- | -- | -- | -- | -- | -- |
| *Cordylus mclachlani* | HQ167097 | HQ167209 | -- | -- | -- | HQ166996 | -- | -- | -- | -- | -- | -- |
| *Cordylus meculae* | HQ167100 | HQ167234 | -- | -- | -- | -- | -- | -- | -- | -- | -- | -- |
| *Cordylus melanotus* | HQ167147 | HQ167258 | -- | -- | -- | HQ167036 | -- | -- | -- | -- | -- | -- |
| *Cordylus microlepidotus* | HQ167149 | HQ167260 | -- | DQ249071 | -- | HQ167038 | -- | -- | -- | -- | -- | -- |
| *Cordylus minor* | AF236032 | HQ167212 | -- | -- | -- | HQ166997 | -- | -- | -- | -- | -- | -- |
| *Cordylus namaquensis* | HQ167104 | HQ167215 | AY987981 | AY217848 | EU116507 | -- | -- | EU108020 | -- | -- | EU108528 | -- |
| *Cordylus nebulosus* | HQ167151 | HQ167262 | -- | -- | -- | HQ167040 | -- | -- | -- | -- | -- | -- |
| *Cordylus niger* | HQ167105 | AY519758 | -- | -- | -- | AY519699 | -- | -- | -- | -- | -- | -- |
| *Cordylus oelofseni* | HQ167107 | HQ167221 | -- | -- | -- | AY519707 | -- | -- | -- | -- | -- | -- |
| *Cordylus peersi* | HQ167112 | HQ167223 | -- | -- | -- | HQ167006 | -- | -- | -- | -- | -- | -- |
| *Cordylus polyzonus* | HQ167116 | JF834012 | -- | -- | -- | AY662561 | -- | -- | -- | -- | AY662643 | -- |
| *Cordylus pustulatus* | HQ167117 | HQ167228 | -- | -- | -- | HQ167011 | -- | -- | -- | -- | -- | -- |
| *Cordylus rhodesianus* | HQ167120 | HQ167231 | -- | -- | -- | HQ167014 | -- | -- | -- | -- | -- | -- |
| *Cordylus spinosus* | HQ167153 | HQ167266 | -- | -- | -- | HQ167044 | -- | -- | -- | -- | -- | -- |
| *Cordylus tasmani* | HQ167121 | HQ167232 | -- | -- | -- | HQ167015 | -- | -- | -- | -- | -- | -- |
| *Cordylus tropidosternum* | HQ167125 | HQ167236 | -- | DQ100148 | DQ090880 | -- | -- | -- | -- | -- | -- | -- |
| *Cordylus ukingensis* | HQ167126 | HQ167237 | -- | -- | -- | -- | -- | -- | -- | -- | -- | -- |
| *Cordylus vittifer* | HQ167131 | HQ167243 | -- | -- | -- | HQ167021 | -- | -- | -- | -- | -- | -- |
| *Cordylus warreni* | HQ167133 | HQ167244 | -- | DQ249072 | DQ249103 | NC_005962 | AB079613 | -- | -- | -- | -- | -- |
| *Coronella austriaca* | AY122836 | EU078988 | -- | AY486954 | AY122752 | AY487026 | AY487065 | -- | -- | -- | -- | -- |
| *Coronella girondica* | AY122835 | EU022641 | -- | AF471113 | AF471088 | AY487027 | AY487066 | -- | -- | -- | -- | -- |
| *Corucia zebrata* | AB028793 | AY308185 | HQ655164 | HQ655201 | -- | -- | -- | -- | -- | -- | -- | -- |
| *Coryphophylax subcristatus* | -- | EU503017 | -- | -- | -- | -- | -- | -- | -- | -- | -- | -- |
| *Corytophanes cristatus* | -- | -- | JF806020 | AF315390 | -- | AF528717 | -- | JF804541 | -- | JF804585 | JF806205 | -- |
| *Corytophanes percarinatus* | -- | -- | -- | -- | -- | AF528718 | -- | -- | -- | -- | -- | -- |
| *Crenadactylus ocellatus* | -- | -- | -- | FJ571641 | -- | AY369016 | -- | -- | JQ945367 | -- | AY662627 | JQ945439 |
| *Cricosaura typica* | M65111 | -- | EU108368 | EU116690 | EU116520 | -- | -- | EU108034 | -- | JN703084 | EU108542 | -- |
| *Crisantophis nevermanni* | GU018152 | GU018169 | -- | -- | -- | -- | -- | -- | -- | -- | -- | -- |
| *Crocodilurus amazonicus* | EF029877 | EF029884 | -- | -- | -- | -- | -- | -- | -- | -- | -- | -- |
| *Crossobamon orientalis* | DQ852715 | -- | HQ443611 | DQ852730 | HM921200 | HQ443529 | -- | -- | JQ945368 | -- | JQ945299 | JQ945440 |
| *Crotalus adamanteus* | AF259255 | AF057269 | -- | -- | AY223605 | -- | U41880 | -- | -- | -- | -- | -- |
| *Crotalus aquilus* | HQ257624 | AF259125 | -- | -- | -- | -- | HQ257787 | -- | -- | -- | -- | -- |
| *Crotalus atrox* | AF259258 | AF057272 | -- | JN090135 | AY223608 | AY016239 | DQ679856 | -- | -- | -- | -- | -- |
| *Crotalus basiliscus* | AF259244 | AF259136 | -- | -- | AY704845 | AY704796 | AY704895 | -- | -- | -- | -- | -- |
| *Crotalus catalinensis* | AF259259 | AF259151 | -- | -- | -- | -- | -- | -- | -- | -- | -- | -- |
| *Crotalus cerastes* | AF259235 | AF259128 | -- | -- | AF259165 | AY016245 | -- | -- | -- | -- | -- | -- |
| *Crotalus durissus* | AF259248 | AF259140 | -- | -- | DQ899736 | AY704776 | AY704869 | -- | -- | -- | -- | -- |
| *Crotalus enyo* | AF259245 | AF259137 | -- | -- | AF259175 | AY016246 | -- | -- | -- | -- | -- | -- |
| *Crotalus horridus* | HM641837 | AF259143 | -- | -- | AF327893 | -- | JN870207 | -- | -- | -- | -- | -- |
| *Crotalus intermedius* | AF259238 | AF259131 | -- | -- | -- | -- | JN870208 | -- | -- | -- | -- | -- |
| *Crotalus lepidus* | HQ257545 | AF259123 | -- | -- | -- | -- | HQ257791 | -- | -- | -- | -- | -- |
| *Crotalus mitchellii* | AF259250 | AF259142 | -- | -- | AF259180 | AY016241 | -- | -- | -- | -- | -- | -- |
| *Crotalus molossus* | AF057224 | AF259135 | -- | -- | AY704849 | AY704798 | AY704897 | -- | -- | -- | -- | -- |
| *Crotalus oreganus* | AY016037 | -- | -- | AF471135 | AF147857 | AY016227 | AF194148 | -- | -- | -- | -- | -- |
| *Crotalus polystictus* | AF512747 | AF259129 | -- | -- | -- | -- | -- | -- | -- | -- | -- | -- |
| *Crotalus pricei* | AF259237 | AF259130 | -- | -- | -- | -- | -- | -- | -- | -- | -- | -- |
| *Crotalus pusillus* | HQ257572 | AF259122 | -- | -- | AF259159 | -- | HQ257774 | -- | -- | -- | -- | -- |
| *Crotalus ravus* | HQ257587 | HQ257578 | -- | -- | AY223609 | -- | HQ257881 | -- | -- | -- | -- | -- |
| *Crotalus ruber* | AF259261 | AF259153 | -- | -- | -- | -- | HQ316632 | -- | -- | -- | -- | -- |
| *Crotalus scutulatus* | AY016047 | AF259146 | -- | -- | AF147876 | AY016238 | AF156574 | -- | -- | -- | -- | -- |
| *Crotalus simus* | EU624240 | GQ372869 | -- | -- | -- | -- | -- | -- | -- | -- | -- | -- |
| *Crotalus tancitarensis* | JN022897 | JN022897 | -- | -- | -- | -- | JN022851 | -- | -- | -- | -- | -- |
| *Crotalus tigris* | AF057223 | AF057270 | -- | -- | AY223606 | AY016240 | AF194166 | GQ334665 | -- | -- | -- | -- |
| *Crotalus tortugensis* | AF259257 | AF259149 | -- | -- | AF259187 | -- | DQ679839 | -- | -- | -- | -- | -- |
| *Crotalus totonacus* | -- | -- | -- | -- | AY704839 | AY704795 | -- | -- | -- | -- | -- | -- |
| *Crotalus transversus* | AF259239 | JN022895 | -- | -- | AF259169 | -- | JN022875 | -- | -- | -- | -- | -- |
| *Crotalus triseriatus* | AF259233 | AF259127 | -- | -- | -- | -- | HQ257775 | -- | -- | -- | -- | -- |
| *Crotalus viridis* | DQ020029 | AF259145 | -- | AF471135 | AF471066 | AY016218 | AF194157 | -- | -- | -- | -- | -- |
| *Crotalus willardi* | AF259242 | AF259134 | -- | -- | AF259171 | -- | JN870209 | -- | -- | -- | -- | -- |
| *Crotaphopeltis tornieri* | -- | -- | -- | AF471112 | AF471093 | -- | -- | -- | -- | -- | -- | -- |
| *Crotaphytus antiquus* | -- | -- | -- | -- | EU037436 | EU038469 | -- | -- | -- | -- | -- | -- |
| *Crotaphytus bicinctores* | -- | -- | -- | -- | EU037685 | EU038720 | -- | -- | -- | -- | -- | -- |
| *Crotaphytus collaris* | -- | L41443 | AY987968 | AY987985 | EU037576 | EU038620 | -- | JF804542 | -- | JF804586 | FJ356749 | -- |
| *Crotaphytus grismeri* | -- | -- | -- | -- | EU037702 | EU038730 | -- | -- | -- | -- | -- | -- |
| *Crotaphytus insularis* | -- | -- | -- | -- | EU037705 | EU038733 | -- | -- | -- | -- | -- | -- |
| *Crotaphytus nebrius* | -- | -- | -- | -- | EU037731 | EU038771 | -- | -- | -- | -- | -- | -- |
| *Crotaphytus reticulatus* | -- | -- | -- | -- | EU037743 | EU038786 | -- | -- | -- | -- | -- | -- |
| *Crotaphytus vestigium* | -- | -- | -- | -- | EU037771 | EU038799 | -- | -- | -- | -- | -- | -- |
| *Cryophis hallbergi* | -- | -- | -- | GQ895807 | GQ895863 | -- | GQ334582 | GU353276 | -- | -- | -- | -- |
| *Cryptactites peringueyi* | DQ852718 | -- | -- | DQ852731 | -- | JX041339 | -- | -- | JQ945369 | -- | JQ945300 | JQ945441 |
| *Cryptelytrops albolabris* | AY352803 | AY059560 | -- | -- | AF517190 | -- | AY352837 | -- | -- | -- | -- | -- |
| *Cryptelytrops andersonii* | AY352801 | AY352740 | -- | -- | -- | -- | -- | -- | -- | -- | -- | -- |
| *Cryptelytrops cantori* | AF057196 | AY352741 | -- | -- | AF171899 | -- | AY352836 | -- | -- | -- | -- | -- |
| *Cryptelytrops erythrurus* | AF517161 | AY352739 | -- | -- | AY352768 | -- | AF517217 | -- | -- | -- | -- | -- |
| *Cryptelytrops fasciatus* | GQ428492 | GQ428466 | -- | -- | -- | -- | -- | -- | -- | -- | -- | -- |
| *Cryptelytrops insularis* | AY059534 | AY059550 | -- | -- | AY059568 | -- | AY352833 | -- | -- | -- | -- | -- |
| *Cryptelytrops kanburiensis* | AY289219 | AY352737 | -- | -- | -- | -- | -- | -- | -- | -- | -- | -- |
| *Cryptelytrops macrops* | GQ428493 | AF517176 | -- | -- | AF517184 | -- | AF517219 | -- | -- | -- | -- | -- |
| *Cryptelytrops purpureomaculatus* | AY352806 | AY352746 | -- | -- | AF517188 | -- | AF517218 | -- | -- | -- | -- | -- |
| *Cryptelytrops septentrionalis* | AY059543 | AY059559 | -- | -- | AY352755 | -- | AY059592 | -- | -- | -- | -- | -- |
| *Cryptelytrops venustus* | AY293931 | AY352723 | -- | -- | AY289234 | -- | AY289230 | -- | -- | -- | -- | -- |
| *Cryptoblepharus boutonii* | AF280116 | AY151445 | -- | AY818775 | AY818808 | -- | -- | -- | -- | -- | -- | -- |
| *Cryptoblepharus nigropunctatus* | -- | AB474011 | -- | -- | -- | -- | -- | -- | -- | -- | -- | -- |
| *Cryptoblepharus novocaledonicus* | DQ118058 | DQ118080 | -- | DQ675410 | -- | DQ675205 | -- | -- | -- | -- | DQ675285 | -- |
| *Ctenoblepharys adspersa* | -- | -- | -- | -- | -- | AF305784 | -- | -- | -- | -- | -- | -- |
| *Ctenophorus adelaidensis* | -- | -- | DQ340740 | DQ340692 | -- | FJ200009 | -- | JF804566 | -- | JF804602 | JF806192 | -- |
| *Ctenophorus caudicinctus* | -- | -- | DQ340709 | DQ340666 | -- | AF375623 | -- | -- | -- | -- | GQ244462 | -- |
| *Ctenophorus clayi* | -- | -- | DQ340710 | DQ340667 | -- | AF375620 | -- | -- | -- | -- | -- | -- |
| *Ctenophorus cristatus* | -- | -- | DQ340711 | DQ340696 | -- | AF375622 | -- | -- | -- | -- | -- | -- |
| *Ctenophorus decresii* | -- | -- | DQ340712 | AF039475 | -- | AF128470 | -- | -- | -- | -- | -- | -- |
| *Ctenophorus femoralis* | -- | -- | -- | -- | -- | AF375627 | -- | -- | -- | -- | -- | -- |
| *Ctenophorus fionni* | -- | -- | -- | -- | -- | AF375638 | -- | -- | -- | -- | -- | -- |
| *Ctenophorus fordi* | -- | -- | DQ340713 | DQ340669 | -- | AF375626 | -- | -- | -- | -- | -- | -- |
| *Ctenophorus gibba* | -- | -- | DQ340714 | DQ340670 | -- | AF375625 | -- | -- | -- | -- | -- | -- |
| *Ctenophorus isolepis* | -- | -- | DQ340715 | DQ340671 | -- | AF375629 | -- | JF804543 | -- | JF804587 | JF806193 | -- |
| *Ctenophorus maculatus* | -- | -- | -- | -- | -- | AF375628 | -- | -- | -- | -- | -- | -- |
| *Ctenophorus maculosus* | -- | -- | -- | -- | -- | AF375621 | -- | -- | -- | -- | -- | -- |
| *Ctenophorus mckenziei* | -- | -- | DQ340716 | DQ340672 | -- | AF375631 | -- | -- | -- | -- | -- | -- |
| *Ctenophorus nuchalis* | -- | -- | DQ340717 | AF137521 | -- | AF375633 | -- | -- | -- | -- | -- | -- |
| *Ctenophorus ornatus* | -- | -- | -- | -- | -- | AF375624 | -- | -- | -- | -- | GQ244461 | -- |
| *Ctenophorus pictus* | -- | -- | DQ340718 | DQ340673 | -- | AF375635 | -- | -- | -- | -- | -- | -- |
| *Ctenophorus reticulatus* | -- | -- | -- | -- | -- | AF375634 | -- | -- | -- | -- | -- | -- |
| *Ctenophorus rufescens* | -- | -- | -- | -- | -- | AF375636 | -- | -- | -- | -- | -- | -- |
| *Ctenophorus salinarum* | -- | -- | -- | -- | -- | DQ683731 | -- | -- | -- | -- | AY662580 | -- |
| *Ctenophorus scutulatus* | -- | -- | -- | -- | -- | AF375632 | -- | -- | -- | -- | -- | -- |
| *Ctenophorus tjantjalka* | -- | -- | -- | -- | -- | AF375637 | -- | -- | -- | -- | -- | -- |
| *Ctenophorus vadnappa* | -- | -- | DQ340719 | DQ340674 | -- | AF375639 | -- | -- | -- | -- | -- | -- |
| *Ctenosaura acanthura* | -- | -- | -- | -- | -- | -- | EU246733 | -- | -- | -- | -- | -- |
| *Ctenosaura bakeri* | -- | -- | -- | GU332023 | GU331979 | -- | EU271879 | -- | -- | -- | -- | -- |
| *Ctenosaura flavidorsalis* | -- | -- | -- | -- | -- | -- | AF417075 | -- | -- | -- | -- | -- |
| *Ctenosaura hemilopha* | -- | -- | -- | -- | AF020254 | -- | EU246696 | -- | -- | -- | -- | -- |
| *Ctenosaura melanosterna* | -- | -- | -- | GU332027 | GU331992 | -- | AY730661 | -- | -- | -- | -- | -- |
| *Ctenosaura oaxacana* | -- | -- | -- | -- | -- | -- | AY730658 | -- | -- | -- | -- | -- |
| *Ctenosaura oedirhina* | -- | -- | -- | GU332029 | GU331982 | -- | EU407504 | -- | -- | -- | -- | -- |
| *Ctenosaura palearis* | -- | -- | -- | AF315392 | GU331984 | -- | U66229 | -- | -- | -- | -- | -- |
| *Ctenosaura pectinata* | -- | -- | -- | -- | HQ141270 | -- | EU246780 | -- | -- | -- | -- | -- |
| *Ctenosaura quinquecarinata* | -- | -- | -- | -- | -- | -- | AY730659 | -- | -- | -- | -- | -- |
| *Ctenosaura similis* | -- | -- | -- | GU332022 | AF020252 | -- | EU407524 | -- | -- | -- | -- | -- |
| *Ctenotus angusticeps* | -- | -- | -- | -- | -- | -- | EU109200 | -- | -- | -- | -- | -- |
| *Ctenotus astarte* | -- | -- | -- | -- | -- | -- | EU109201 | -- | -- | -- | -- | -- |
| *Ctenotus atlas* | -- | -- | -- | -- | GQ241585 | -- | EU109202 | -- | -- | -- | -- | -- |
| *Ctenotus australis* | -- | -- | -- | -- | -- | -- | EU109203 | -- | -- | -- | -- | -- |
| *Ctenotus brooksi* | -- | -- | -- | -- | -- | -- | EU109204 | -- | -- | -- | -- | -- |
| *Ctenotus calurus* | -- | -- | -- | -- | -- | -- | EU109205 | -- | -- | -- | -- | -- |
| *Ctenotus essingtonii* | -- | -- | -- | -- | -- | -- | EU109206 | -- | -- | -- | -- | -- |
| *Ctenotus fallens* | -- | -- | -- | -- | -- | -- | EU109207 | -- | -- | -- | -- | -- |
| *Ctenotus gagudju* | -- | -- | -- | -- | GQ241606 | -- | EU109208 | -- | -- | -- | -- | -- |
| *Ctenotus grandis* | -- | -- | -- | -- | -- | -- | EU109209 | -- | -- | -- | -- | -- |
| *Ctenotus greeri* | -- | -- | -- | -- | GQ241596 | -- | -- | -- | -- | -- | -- | -- |
| *Ctenotus hanloni* | -- | -- | -- | -- | -- | -- | EU109210 | -- | -- | -- | -- | -- |
| *Ctenotus hebetior* | -- | -- | -- | -- | GQ241608 | -- | EU109211 | -- | -- | -- | -- | -- |
| *Ctenotus hilli* | -- | -- | -- | -- | -- | -- | EU109212 | -- | -- | -- | -- | -- |
| *Ctenotus inornatus* | AY308337 | AY308188 | -- | -- | -- | -- | -- | -- | -- | -- | -- | -- |
| *Ctenotus labillardieri* | -- | -- | -- | -- | GQ241602 | -- | EU109213 | -- | -- | -- | -- | -- |
| *Ctenotus leae* | -- | -- | -- | -- | -- | -- | EU109214 | -- | -- | -- | -- | -- |
| *Ctenotus leonhardii* | AY169579 | AY169616 | -- | -- | GQ241562 | -- | AY169654 | -- | -- | -- | -- | -- |
| *Ctenotus maryani* | -- | -- | -- | -- | GQ241615 | -- | EU109217 | -- | -- | -- | -- | -- |
| *Ctenotus mimetes* | -- | -- | -- | -- | GQ241609 | -- | -- | -- | -- | -- | -- | -- |
| *Ctenotus nasutus* | -- | -- | -- | -- | -- | -- | EU109218 | -- | -- | -- | -- | -- |
| *Ctenotus olympicus* | -- | -- | -- | -- | GQ241619 | -- | -- | -- | -- | -- | -- | -- |
| *Ctenotus pantherinus* | DQ915298 | AY169617 | -- | -- | -- | -- | AY169655 | -- | -- | -- | -- | -- |
| *Ctenotus piankai* | -- | -- | -- | -- | -- | -- | EU109221 | -- | -- | -- | -- | -- |
| *Ctenotus pulchellus* | -- | -- | -- | -- | GQ241614 | -- | -- | -- | -- | -- | -- | -- |
| *Ctenotus quattuordecimlineatus* | -- | -- | -- | -- | GQ241583 | -- | EU109222 | -- | -- | -- | -- | -- |
| *Ctenotus rawlinsoni* | -- | AF530226 | -- | -- | -- | -- | AF530263 | -- | -- | -- | -- | -- |
| *Ctenotus regius* | -- | -- | -- | -- | GQ241592 | -- | -- | -- | -- | -- | -- | -- |
| *Ctenotus robustus* | AY169581 | AY169618 | -- | -- | -- | AY662548 | AY169656 | -- | -- | -- | AY662630 | -- |
| *Ctenotus rubicundus* | -- | -- | -- | -- | -- | -- | EU109224 | -- | -- | -- | -- | -- |
| *Ctenotus rutilans* | -- | -- | -- | -- | GQ241611 | -- | -- | -- | -- | -- | -- | -- |
| *Ctenotus saxatilis* | -- | -- | -- | -- | GQ241603 | -- | EU109225 | -- | -- | -- | -- | -- |
| *Ctenotus schomburgkii* | -- | -- | -- | -- | -- | -- | EU109226 | -- | -- | -- | -- | -- |
| *Ctenotus septenarius* | -- | -- | -- | -- | GQ241595 | -- | EU109227 | -- | -- | -- | -- | -- |
| *Ctenotus serventyi* | -- | -- | -- | -- | GQ241605 | -- | -- | -- | -- | -- | -- | -- |
| *Ctenotus spaldingi* | -- | -- | -- | -- | -- | -- | EU109228 | -- | -- | -- | -- | -- |
| *Ctenotus strauchii* | -- | -- | -- | -- | -- | -- | EU109229 | -- | -- | -- | -- | -- |
| *Ctenotus taeniolatus* | AY818758 | -- | -- | AY818792 | -- | -- | EU109230 | -- | -- | -- | -- | -- |
| *Ctenotus tanamiensis* | -- | -- | -- | -- | GQ241597 | -- | -- | -- | -- | -- | -- | -- |
| *Ctenotus uber* | -- | -- | -- | -- | GQ241601 | -- | EU109219 | -- | -- | -- | -- | -- |
| *Ctenotus youngsoni* | -- | -- | -- | -- | -- | -- | EU109231 | -- | -- | -- | -- | -- |
| *Cyclodina aenea* | AF194088 | EU567866 | -- | DQ675384 | EU567771 | EF567164 | EU567745 | -- | -- | -- | EU568058 | -- |
| *Cyclodina alani* | EU567972 | EU567844 | -- | -- | EU567775 | EF567168 | EF043169 | -- | -- | -- | EU568070 | -- |
| *Cyclodina hardyi* | -- | EU567872 | -- | -- | EU567780 | EF567125 | EU567747 | -- | -- | -- | EU568060 | -- |
| *Cyclodina levidensa* | -- | -- | -- | -- | -- | EF567121 | -- | -- | -- | -- | -- | -- |
| *Cyclodina lichenigera* | EU567966 | EU567870 | -- | DQ675385 | EU567829 | EU567704 | EU567763 | -- | -- | -- | EU568108 | -- |
| *Cyclodina macgregori* | EU567974 | EU567846 | -- | -- | EU567777 | EF567174 | EU567731 | -- | -- | -- | EU568064 | -- |
| *Cyclodina oliveri* | EU567979 | EU567856 | -- | -- | EF081246 | EF033045 | EF081208 | -- | -- | -- | EU568077 | -- |
| *Cyclodina ornata* | EU567976 | EU567864 | -- | -- | EF104030 | EF567202 | EU567734 | -- | -- | -- | EU568079 | -- |
| *Cyclodina townsi* | EU567983 | EU567860 | -- | -- | EF081231 | EF081194 | EF081218 | -- | -- | -- | EU568080 | -- |
| *Cyclodina whitakeri* | EU567986 | EU567862 | -- | -- | EF081248 | EU852578 | EF081210 | -- | -- | -- | EU568084 | -- |
| *Cyclodomorphus branchialis* | AY308338 | AY308189 | -- | -- | -- | -- | -- | -- | -- | -- | -- | -- |
| *Cyclodomorphus casuarinae* | AF280118 | AY151447 | -- | AY818773 | AF280129 | -- | -- | -- | -- | -- | -- | -- |
| *Cyclodomorphus michaeli* | -- | -- | HQ655165 | HQ655202 | -- | -- | -- | -- | -- | -- | -- | -- |
| *Cyclophiops major* | -- | -- | -- | KC347395 | KC347471 | KC347452 | -- | -- | -- | -- | -- | -- |
| *Cyclura carinata* | -- | -- | -- | HM352534 | -- | -- | DQ397520 | HM352524 | -- | -- | -- | -- |
| *Cyclura collei* | -- | -- | -- | -- | -- | -- | AF217773 | -- | -- | -- | -- | -- |
| *Cyclura cornuta* | -- | -- | -- | -- | U88955 | -- | AF217770 | -- | -- | -- | -- | -- |
| *Cyclura cychlura* | -- | -- | -- | -- | -- | -- | AF217781 | -- | -- | -- | -- | -- |
| *Cyclura nubila* | -- | -- | FJ433956 | DQ119595 | AF020255 | -- | U66236 | FJ434062 | -- | DQ119623 | DQ119625 | DQ119641 |
| *Cyclura pinguis* | EU793996 | -- | GQ853272 | -- | -- | -- | AF217772 | GQ853278 | -- | -- | GQ853279 | -- |
| *Cyclura ricordi* | -- | -- | -- | HM352533 | -- | -- | U66237 | HM352523 | -- | -- | -- | -- |
| *Cyclura rileyi* | -- | -- | -- | -- | -- | -- | AF217777 | -- | -- | -- | -- | -- |
| *Cylindrophis maculatus* | KC347320 | KC347355 | -- | KC347396 | KC347472 | -- | KC347508 | -- | -- | -- | KC347433 | -- |
| *Cylindrophis ruffus* | AB179619 | AF512738 | AY988037 | AF471133 | AF471032 | AB179619 | EF056500 | EU390915 | -- | JF804588 | AY662613 | -- |
| *Cynisca leucura* | -- | -- | -- | AY444011 | -- | -- | -- | -- | -- | -- | AY444038 | -- |
| *Cyrtodactylus agusanensis* | -- | -- | -- | -- | -- | HQ154532 | -- | -- | -- | -- | -- | -- |
| *Cyrtodactylus angularis* | -- | -- | -- | JQ945549 | -- | HQ401212 | -- | -- | JQ945370 | -- | JQ945301 | JQ945442 |
| *Cyrtodactylus annulatus* | -- | -- | -- | -- | -- | GU550763 | -- | -- | -- | -- | -- | -- |
| *Cyrtodactylus ayeyarwadyensis* | -- | -- | -- | JQ945550 | EU268380 | EU268348 | EU268411 | -- | EU268317 | -- | -- | JQ945443 |
| *Cyrtodactylus consobrinus* | -- | -- | -- | -- | EU268381 | EU268349 | EU268412 | -- | EU268318 | -- | -- | -- |
| *Cyrtodactylus epiroticus* | -- | -- | -- | -- | -- | HQ401196 | -- | -- | -- | -- | -- | -- |
| *Cyrtodactylus intermedius* | AB028743 | AB028757 | -- | -- | -- | GU550712 | -- | -- | -- | -- | -- | -- |
| *Cyrtodactylus irregularis* | -- | -- | -- | JQ945551 | -- | JX041341 | -- | -- | JQ945371 | -- | JQ945302 | JQ945444 |
| *Cyrtodactylus jarujini* | -- | -- | -- | JQ945552 | -- | HQ401213 | -- | -- | JQ945372 | -- | JQ945303 | JQ945445 |
| *Cyrtodactylus klugei* | -- | -- | -- | -- | -- | HQ401198 | -- | -- | -- | -- | -- | -- |
| *Cyrtodactylus loriae* | -- | -- | -- | -- | EU268382 | EU268350 | EU268413 | -- | EU268319 | -- | -- | -- |
| *Cyrtodactylus louisiadensis* | -- | -- | -- | -- | -- | HQ401190 | -- | -- | -- | -- | -- | -- |
| *Cyrtodactylus marmoratus* | -- | -- | -- | -- | -- | GQ257747 | -- | -- | -- | -- | -- | -- |
| *Cyrtodactylus novaeguineae* | -- | -- | -- | HQ426531 | -- | HQ401210 | -- | -- | HQ426185 | -- | HQ426274 | HQ426447 |
| *Cyrtodactylus oldhami* | -- | JF799758 | -- | -- | -- | -- | -- | -- | -- | -- | -- | -- |
| *Cyrtodactylus philippinicus* | -- | -- | -- | JQ945553 | -- | JX041344 | -- | -- | JQ945373 | -- | JQ945304 | JQ945446 |
| *Cyrtodactylus pulchellus* | DQ852717 | -- | -- | DQ852732 | -- | -- | -- | -- | -- | -- | -- | -- |
| *Cyrtodactylus robustus* | -- | -- | -- | -- | -- | HQ401204 | -- | -- | -- | -- | -- | -- |
| *Cyrtodactylus sermowaiensis* | -- | -- | -- | -- | -- | HQ401211 | -- | -- | -- | -- | -- | -- |
| *Cyrtodactylus tripartitus* | -- | -- | -- | -- | -- | HQ401203 | -- | -- | -- | -- | -- | -- |
| *Cyrtodactylus tuberculatus* | -- | -- | -- | -- | -- | HQ401109 | -- | -- | -- | -- | -- | -- |
| *Cyrtopodion agamuroides* | EU589161 | -- | -- | -- | EU589185 | -- | -- | -- | -- | -- | -- | -- |
| *Cyrtopodion caspium* | EU589163 | -- | -- | JQ945620 | EU589187 | JX041448 | -- | -- | JQ945409 | -- | JQ945340 | JQ945514 |
| *Cyrtopodion gastrophole* | EU589165 | -- | -- | -- | EU589189 | -- | -- | -- | -- | -- | -- | -- |
| *Cyrtopodion heterocercum* | EU589167 | -- | -- | -- | -- | -- | -- | -- | -- | -- | -- | -- |
| *Cyrtopodion kotschyi* | EU589168 | -- | -- | -- | EU589192 | -- | -- | -- | -- | -- | -- | -- |
| *Cyrtopodion longipes* | EU589170 | -- | -- | JQ945621 | EU589193 | JX041449 | -- | -- | JQ945410 | -- | JQ945341 | JQ945515 |
| *Cyrtopodion russowii* | -- | -- | -- | JQ945588 | -- | JX041384 | -- | -- | JQ945383 | -- | JQ945315 | JQ945481 |
| *Cyrtopodion sagittiferum* | EU589171 | -- | -- | -- | EU589194 | -- | -- | -- | -- | -- | -- | -- |
| *Cyrtopodion scabrum* | EU589172 | -- | -- | HQ426532 | EU589195 | JX041345 | -- | -- | HQ426186 | -- | HQ426275 | HQ426448 |
| *Cyrtopodion sistanensis* | EU589175 | -- | -- | -- | EU589198 | -- | -- | -- | -- | -- | -- | -- |
| *Cyrtopodion spinicaudum* | -- | -- | -- | JQ945589 | -- | JX041385 | -- | -- | JQ945384 | -- | JQ945316 | JQ945482 |
| *Daboia russelii* | GQ225676 | GQ398147 | EU402636 | AF471156 | AF471076 | NC_011391 | DQ305477 | EU390916 | -- | HQ876367 | EU402843 | -- |
| *Dalmatolacerta oxycephala* | AY256656 | -- | -- | EF632271 | GQ142129 | -- | -- | -- | -- | -- | EF632228 | -- |
| *Darevskia alpina* | -- | AF206185 | -- | -- | U88601 | -- | -- | -- | -- | -- | -- | -- |
| *Darevskia armeniaca* | -- | -- | -- | -- | AF147799 | -- | -- | -- | -- | -- | -- | -- |
| *Darevskia bendimahiensis* | -- | -- | -- | -- | AF164084 | -- | -- | -- | -- | -- | -- | -- |
| *Darevskia brauneri* | -- | AF206183 | -- | -- | AF206179 | -- | -- | -- | -- | -- | -- | -- |
| *Darevskia caucasica* | -- | AF206187 | -- | -- | U88616 | -- | -- | -- | -- | -- | -- | -- |
| *Darevskia chlorogaster* | AF080284 | AF080286 | -- | -- | AF080285 | -- | -- | -- | -- | -- | -- | -- |
| *Darevskia clarkorum* | -- | AF206190 | -- | -- | U88605 | -- | -- | -- | -- | -- | -- | -- |
| *Darevskia daghestanica* | -- | AF206188 | -- | -- | AF206171 | -- | -- | -- | -- | -- | -- | -- |
| *Darevskia derjugini* | EF422431 | AF206191 | -- | -- | AF164073 | -- | -- | -- | -- | -- | -- | -- |
| *Darevskia lindholmi* | -- | AF206182 | -- | -- | AF206177 | -- | -- | -- | -- | -- | -- | -- |
| *Darevskia mixta* | -- | AF206189 | -- | -- | -- | -- | -- | -- | -- | -- | -- | -- |
| *Darevskia parvula* | -- | AF206195 | -- | -- | U88609 | -- | -- | -- | -- | -- | -- | -- |
| *Darevskia portschinskii* | -- | AF206194 | -- | -- | U88615 | -- | -- | -- | -- | -- | -- | -- |
| *Darevskia praticola* | -- | AF206186 | -- | -- | U88612 | -- | -- | -- | -- | -- | -- | -- |
| *Darevskia raddei* | -- | AF206192 | -- | -- | AF164074 | -- | -- | -- | -- | -- | -- | -- |
| *Darevskia rostombekovi* | -- | -- | -- | -- | AF164091 | -- | -- | -- | -- | -- | -- | -- |
| *Darevskia rudis* | AJ238180 | AF206193 | -- | -- | U88614 | -- | -- | -- | -- | -- | -- | -- |
| *Darevskia sapphirina* | -- | -- | -- | -- | AF164083 | -- | -- | -- | -- | -- | -- | -- |
| *Darevskia saxicola* | AF080281 | AF080283 | -- | -- | AF206180 | -- | -- | -- | -- | -- | -- | -- |
| *Darevskia uzzelli* | -- | -- | -- | -- | AF164082 | -- | -- | -- | -- | -- | -- | -- |
| *Darevskia valentini* | AF206597 | GQ142098 | -- | EF632257 | GQ142123 | -- | -- | -- | -- | -- | EF632212 | -- |
| *Darlingtonia haetiana* | AF158458 | AF158527 | -- | -- | FJ416736 | FJ416774 | FJ416810 | -- | -- | -- | -- | FJ416847 |
| *Dasia grisea* | AB028773 | AB028784 | HQ907226 | -- | -- | -- | JF498460 | -- | -- | HQ907631 | -- | -- |
| *Dasia olivacea* | AB028772 | AB028783 | -- | -- | -- | -- | -- | -- | -- | -- | -- | -- |
| *Dasypeltis atra* | -- | -- | -- | AF471136 | AF471065 | -- | -- | -- | -- | -- | -- | -- |
| *Dasypeltis confusa* | -- | JQ801322 | -- | -- | -- | -- | -- | -- | -- | -- | -- | -- |
| *Dasypeltis fasciata* | -- | JQ801334 | -- | -- | -- | -- | -- | -- | -- | -- | -- | -- |
| *Dasypeltis gansi* | -- | JQ801320 | -- | -- | -- | -- | -- | -- | -- | -- | -- | -- |
| *Dasypeltis sahelensis* | -- | JQ801328 | -- | -- | -- | -- | -- | -- | -- | -- | -- | -- |
| *Dasypeltis scabra* | -- | JQ801324 | -- | -- | AY235729 | -- | -- | -- | -- | -- | -- | -- |
| *Davewakeum miriamae* | -- | -- | HQ907236 | -- | -- | HQ907436 | -- | -- | -- | HQ907643 | -- | -- |
| *Deinagkistrodon acutus* | DQ343647 | EU729429 | -- | -- | AF171919 | DQ836216 | AY352811 | -- | -- | -- | -- | -- |
| *Delma australis* | -- | AY134510 | -- | FJ571647 | -- | AY134582 | -- | -- | -- | -- | FJ571633 | -- |
| *Delma borea* | -- | AY134511 | GU457867 | AY134547 | -- | AY134583 | -- | GU456023 | -- | HQ876381 | GU457990 | -- |
| *Delma butleri* | -- | GU460138 | -- | AY134548 | -- | AY134584 | -- | -- | GU459740 | -- | HQ426276 | HQ426449 |
| *Delma concinna* | -- | AY134514 | -- | FJ571648 | -- | AY134586 | -- | -- | -- | -- | FJ571634 | -- |
| *Delma fraseri* | AF090182 | AY134515 | -- | AY134551 | -- | AY134587 | -- | -- | -- | -- | FJ571636 | -- |
| *Delma grayii* | -- | AY134516 | -- | AY134552 | -- | AY134588 | -- | -- | -- | -- | -- | -- |
| *Delma impar* | -- | AY134517 | -- | AY134553 | -- | AY134589 | -- | -- | -- | -- | -- | -- |
| *Delma inornata* | -- | AY134518 | -- | AY134554 | -- | AY134590 | -- | -- | -- | -- | -- | -- |
| *Delma labialis* | -- | AY134519 | -- | AY134555 | -- | AY134591 | -- | -- | -- | -- | -- | -- |
| *Delma mitella* | -- | AY134520 | -- | AY134556 | -- | AY134592 | -- | -- | -- | -- | -- | -- |
| *Delma molleri* | -- | AY134521 | -- | FJ571649 | -- | AY134593 | -- | -- | -- | -- | FJ571635 | -- |
| *Delma nasuta* | -- | AY134522 | -- | AY134558 | -- | AY134594 | -- | -- | -- | -- | -- | -- |
| *Delma pax* | -- | AY134523 | -- | AY134559 | -- | AY134595 | -- | -- | -- | -- | -- | -- |
| *Delma tincta* | DQ852694 | AY134525 | -- | AY134561 | -- | AY134597 | -- | -- | HQ426188 | -- | HQ426277 | HQ426450 |
| *Delma torquata* | -- | AY134526 | -- | AY134562 | -- | AY134598 | -- | -- | -- | -- | -- | -- |
| *Demansia papuensis* | EU547093 | EU547142 | -- | EU546910 | -- | -- | EU547002 | -- | -- | -- | EU546871 | -- |
| *Demansia psammophis* | GQ397248 | GQ397240 | -- | -- | -- | -- | -- | -- | -- | -- | -- | -- |
| *Demansia vestigiata* | EU547094 | EU547143 | -- | -- | -- | -- | EU547003 | -- | -- | -- | EU546872 | -- |
| *Dendrelaphis bifrenalis* | KC347321 | KC347356 | -- | KC347397 | KC347473 | -- | KC347509 | -- | -- | -- | KC347434 | -- |
| *Dendrelaphis caudolineatus* | AF544782 | AF544811 | -- | GQ895808 | GQ895864 | -- | -- | -- | -- | -- | -- | -- |
| *Dendrelaphis caudolineolatus* | -- | KC347357 | -- | -- | KC347474 | -- | KC347510 | -- | -- | -- | -- | -- |
| *Dendrelaphis schokari* | -- | KC347358 | -- | KC347398 | KC347475 | -- | KC347511 | -- | -- | -- | KC347435 | -- |
| *Dendrelaphis tristis* | KC347322 | KC347359 | -- | KC347399 | KC347476 | -- | KC347512 | -- | -- | -- | KC347436 | -- |
| *Dendroaspis angusticeps* | AF544764 | JF357945 | FJ433988 | AF544735 | JF357936 | -- | JF357927 | FJ434089 | -- | EF144072 | AY487395 | EF144099 |
| *Dendroaspis polylepis* | -- | -- | -- | AY058928 | AF217832 | AY059003 | AY058974 | -- | -- | -- | -- | -- |
| *Dendrophidion dendrophis* | -- | -- | -- | GQ895809 | GQ895865 | -- | -- | -- | -- | -- | -- | -- |
| *Dendrophidion percarinatus* | HM565757 | -- | -- | -- | -- | -- | -- | -- | -- | -- | -- | -- |
| *Denisonia devisi* | EU547120 | EU547169 | -- | EU546933 | -- | -- | EU547023 | -- | -- | -- | EU546894 | -- |
| *Diadophis punctatus* | AY577014 | AY577024 | GU112373 | AF471122 | AF471094 | -- | DQ364667 | FJ434098 | -- | EF144083 | AY487403 | EF144110 |
| *Dibamus bourreti* | -- | -- | HQ876238 | HQ876395 | -- | HQ876245 | -- | -- | HQ426251 | HQ876385 | HQ876454 | HQ426513 |
| *Dibamus celebensis* | -- | -- | HQ876239 | HQ876396 | -- | HQ876246 | -- | -- | -- | HQ876386 | HQ876455 | -- |
| *Dibamus greeri* | -- | -- | HQ876240 | HQ876397 | -- | HQ876247 | -- | -- | -- | HQ876387 | HQ876455 | -- |
| *Dibamus montanus* | -- | -- | HQ876241 | AY444027 | -- | HQ876248 | -- | -- | -- | HQ876388 | AY444053 | -- |
| *Dibamus novaeguineae* | -- | -- | EU108355 | EF450999 | EU116508 | FJ195390 | FJ195390 | EU108021 | -- | -- | EU108529 | -- |
| *Dibamus seramensis* | -- | -- | HQ876242 | HQ876399 | -- | HQ876250 | -- | -- | -- | HQ876389 | HQ876458 | -- |
| *Dibamus tiomanensis* | -- | -- | HQ876243 | HQ876400 | -- | HQ876251 | -- | -- | -- | HQ876390 | HQ876459 | -- |
| *Dicrodon guttulatum* | AY046453 | AY046495 | -- | -- | -- | -- | -- | -- | -- | -- | -- | -- |
| *Dierogekko inexpectatus* | -- | -- | -- | -- | -- | JF972441 | -- | -- | -- | -- | -- | -- |
| *Dierogekko insularis* | -- | -- | -- | JQ945555 | -- | JF972459 | -- | -- | JQ945375 | -- | JQ945306 | JQ945448 |
| *Dierogekko kaalaensis* | -- | -- | -- | -- | -- | JF972455 | -- | -- | -- | -- | -- | -- |
| *Dierogekko koniambo* | -- | -- | -- | -- | -- | JF972453 | -- | -- | -- | -- | -- | -- |
| *Dierogekko nehoueensis* | -- | -- | -- | -- | -- | JF972437 | -- | -- | -- | -- | -- | -- |
| *Dierogekko poumensis* | -- | -- | -- | -- | -- | JF972447 | -- | -- | -- | -- | -- | -- |
| *Dierogekko thomaswhitei* | -- | -- | -- | -- | -- | JF972449 | -- | -- | -- | -- | -- | -- |
| *Dierogekko validiclavis* | -- | -- | -- | -- | -- | JF972461 | -- | -- | -- | -- | -- | -- |
| *Dinarolacerta montenegrina* | GQ142078 | GQ142101 | -- | GQ142153 | GQ142141 | -- | -- | -- | -- | -- | GQ142163 | -- |
| *Dinarolacerta mosorensis* | AF440600 | AF440615 | -- | AY151995 | GQ142130 | -- | -- | -- | -- | -- | EF632227 | -- |
| *Dinodon rufozonatum* | AF233939 | HM439982 | JQ599018 | AF471163 | AF471063 | -- | JF827649 | -- | -- | -- | AY662611 | -- |
| *Dinodon semicarinatum* | -- | -- | -- | -- | NC_001945 | NC_001945 | NC_001945 | -- | -- | -- | -- | -- |
| *Diplodactylus capensis* | -- | -- | -- | -- | -- | FJ665567 | -- | -- | -- | -- | -- | -- |
| *Diplodactylus conspicillatus* | -- | -- | -- | HQ426533 | -- | EF681786 | -- | -- | HQ426189 | -- | HQ426278 | HQ426451 |
| *Diplodactylus fulleri* | -- | -- | -- | -- | -- | EF681772 | -- | -- | -- | -- | -- | -- |
| *Diplodactylus galeatus* | -- | -- | -- | -- | -- | FJ665572 | -- | -- | -- | -- | -- | -- |
| *Diplodactylus granariensis* | -- | -- | -- | FJ855473 | -- | EF532897 | JQ398450 | -- | JQ173674 | -- | FJ855452 | -- |
| *Diplodactylus klugei* | -- | -- | -- | -- | -- | EF681788 | -- | -- | -- | -- | -- | -- |
| *Diplodactylus mitchelli* | -- | -- | -- | -- | -- | FJ665565 | -- | -- | -- | -- | -- | -- |
| *Diplodactylus ornatus* | -- | -- | -- | -- | -- | FJ665561 | JQ398449 | -- | JQ173675 | -- | JQ173723 | -- |
| *Diplodactylus polyophthalmus* | -- | -- | -- | -- | -- | EF681793 | -- | -- | -- | -- | -- | -- |
| *Diplodactylus pulcher* | -- | -- | -- | -- | -- | EF681789 | -- | -- | -- | -- | -- | -- |
| *Diplodactylus savagei* | -- | -- | -- | -- | -- | EF681791 | -- | -- | -- | -- | -- | -- |
| *Diplodactylus tessellatus* | -- | AY134535 | -- | FJ571639 | -- | FJ665606 | JQ398452 | -- | JQ173677 | -- | FJ571624 | JQ945449 |
| *Diplodactylus vittatus* | AF090178 | -- | -- | AF090847 | -- | EF532893 | -- | -- | -- | -- | -- | -- |
| *Diploglossus bilobatus* | -- | -- | -- | -- | -- | AF085608 | -- | -- | -- | -- | -- | -- |
| *Diploglossus pleii* | -- | -- | -- | -- | -- | AF085609 | -- | -- | -- | -- | -- | -- |
| *Diplolaemus darwinii* | AF338326 | -- | AY987973 | AY987988 | -- | AF055924 | -- | -- | -- | -- | AY988019 | -- |
| *Diplometopon zarudnyi* | -- | -- | JN654798 | AY444023 | NC_006283 | NC_006283 | NC_006283 | JN568326 | -- | JN568505 | JN654858 | -- |
| *Diporiphora albilabris* | -- | -- | DQ340720 | DQ340675 | -- | AY133003 | -- | -- | -- | -- | HQ681196 | -- |
| *Diporiphora arnhemica* | -- | -- | -- | -- | -- | AY133004 | -- | -- | -- | -- | -- | -- |
| *Diporiphora australis* | -- | -- | -- | -- | -- | AY133005 | -- | -- | -- | -- | JN815263 | -- |
| *Diporiphora bennettii* | -- | -- | DQ340721 | DQ340676 | -- | AY133006 | -- | -- | -- | -- | HQ681197 | -- |
| *Diporiphora bilineata* | -- | -- | DQ340722 | DQ340677 | -- | -- | -- | -- | -- | -- | HQ681238 | -- |
| *Diporiphora lalliae* | -- | -- | DQ340723 | DQ340678 | -- | AY133007 | -- | -- | -- | -- | HQ681217 | -- |
| *Diporiphora linga* | -- | -- | -- | -- | -- | AY133008 | -- | -- | -- | -- | -- | -- |
| *Diporiphora magna* | -- | -- | -- | -- | -- | AY133009 | -- | -- | -- | -- | HQ681239 | -- |
| *Diporiphora pindan* | -- | -- | -- | -- | -- | AY133010 | -- | -- | -- | -- | HQ681198 | -- |
| *Diporiphora reginae* | -- | -- | DQ340724 | DQ340679 | -- | AY133011 | -- | -- | -- | -- | -- | -- |
| *Diporiphora superba* | -- | -- | -- | -- | -- | HQ699066 | -- | -- | -- | -- | HQ681205 | -- |
| *Diporiphora valens* | -- | -- | -- | -- | -- | HQ699062 | -- | -- | -- | -- | HQ681201 | -- |
| *Diporiphora winneckei* | -- | -- | DQ340725 | DQ340680 | -- | AY133012 | -- | -- | -- | -- | HQ681204 | -- |
| *Dipsadoboa unicolor* | -- | -- | -- | AF471139 | AF471062 | -- | -- | -- | -- | -- | -- | -- |
| *Dipsas albifrons* | JQ598803 | JQ598866 | JQ599019 | -- | JQ598925 | -- | -- | -- | -- | -- | -- | -- |
| *Dipsas articulata* | JQ598804 | JQ598867 | JQ599020 | -- | -- | -- | -- | -- | -- | -- | -- | -- |
| *Dipsas catesbyi* | JQ598805 | JQ598868 | JQ599021 | JQ598977 | JQ598926 | -- | EF078585 | GU353277 | -- | -- | -- | -- |
| *Dipsas indica* | GQ457789 | GQ457730 | -- | GQ457850 | -- | -- | -- | -- | -- | -- | -- | -- |
| *Dipsas neivai* | GQ457790 | GQ457731 | -- | GQ457851 | -- | -- | -- | -- | -- | -- | -- | -- |
| *Dipsas pratti* | -- | -- | -- | -- | GQ334482 | -- | GQ334583 | GQ334667 | -- | -- | -- | -- |
| *Dipsas variegata* | AF158406 | AF158476 | -- | -- | -- | -- | -- | -- | -- | -- | -- | -- |
| *Dipsina multimaculata* | -- | -- | -- | DQ486181 | DQ486357 | -- | -- | -- | -- | -- | -- | -- |
| *Dipsosaurus dorsalis* | -- | -- | GQ853275 | AF148705 | EU116509 | AF049857 | U66239 | JF804545 | -- | HQ876329 | FJ356747 | -- |
| *Dispholidus typus* | -- | AY188051 | -- | AY187973 | AY188012 | -- | U49302 | -- | -- | -- | -- | -- |
| *Disteira kingii* | -- | DQ234014 | -- | FJ587184 | DQ233933 | -- | FJ593208 | -- | -- | -- | FJ587106 | -- |
| *Disteira major* | -- | DQ234018 | -- | FJ587186 | DQ233937 | -- | FJ593209 | -- | -- | -- | FJ587108 | -- |
| *Ditypophis vivax* | FJ404150 | AY188052 | -- | AY187974 | AY188013 | -- | FJ404352 | -- | -- | -- | -- | FJ404424 |
| *Dixonius melanostictus* | -- | -- | -- | -- | -- | HM997153 | -- | -- | HM997177 | -- | HM997165 | -- |
| *Dixonius siamensis* | -- | -- | -- | JQ945557 | -- | EU054299 | -- | -- | EU054267 | -- | EU054283 | JQ945450 |
| *Dixonius vietnamensis* | -- | -- | -- | JQ945558 | -- | EU054298 | -- | -- | EU054266 | -- | EU054282 | JQ945451 |
| *Dolichophis caspius* | AY039135 | AY376768 | -- | AY376797 | AY039173 | AY487000 | AY487039 | -- | -- | -- | -- | -- |
| *Dolichophis jugularis* | -- | -- | -- | AY486941 | AY486917 | AY487007 | AY487046 | -- | -- | -- | -- | -- |
| *Dolichophis schmidti* | -- | -- | -- | AY486947 | AY486923 | AY487015 | AY487054 | -- | -- | -- | -- | -- |
| *Dracaena guianensis* | EF029878 | EF029886 | -- | -- | -- | -- | -- | -- | -- | -- | -- | -- |
| *Draco beccarii* | -- | -- | -- | -- | -- | AF288276 | -- | -- | -- | -- | -- | -- |
| *Draco biaro* | -- | -- | -- | -- | -- | AF288277 | -- | -- | -- | -- | -- | -- |
| *Draco bimaculatus* | -- | -- | -- | -- | -- | AF288241 | -- | -- | -- | -- | -- | -- |
| *Draco blanfordii* | AB023733 | AB023751 | JF806010 | -- | -- | AF288242 | -- | JF804546 | -- | JF804589 | JF806194 | -- |
| *Draco boschmai* | -- | -- | -- | -- | -- | AF288273 | -- | -- | -- | -- | -- | -- |
| *Draco bourouniensis* | -- | -- | -- | -- | -- | AF288279 | -- | -- | -- | -- | -- | -- |
| *Draco caerulhians* | -- | -- | -- | -- | -- | AF288281 | -- | -- | -- | -- | -- | -- |
| *Draco cornutus* | AB023729 | AB023752 | -- | -- | -- | AF288244 | -- | -- | -- | -- | -- | -- |
| *Draco cristatellus* | -- | -- | -- | -- | -- | AF288255 | -- | -- | -- | -- | -- | -- |
| *Draco cyanopterus* | -- | -- | -- | -- | -- | AF288245 | -- | -- | -- | -- | -- | -- |
| *Draco dussumieri* | AB023734 | AB023753 | -- | -- | -- | -- | -- | -- | -- | -- | -- | -- |
| *Draco fimbriatus* | -- | -- | -- | -- | -- | AF288257 | -- | -- | -- | -- | -- | -- |
| *Draco guentheri* | -- | -- | -- | -- | -- | AF288260 | -- | -- | -- | -- | -- | -- |
| *Draco haematopogon* | AB023736 | AB023755 | -- | -- | -- | AF288259 | -- | -- | -- | -- | -- | -- |
| *Draco indochinensis* | -- | -- | -- | -- | -- | AF288243 | -- | -- | -- | -- | -- | -- |
| *Draco lineatus* | AB023738 | AB023757 | -- | -- | -- | -- | -- | -- | -- | -- | -- | -- |
| *Draco maculatus* | AB023739 | AB023758 | -- | -- | -- | AF288248 | -- | -- | -- | -- | -- | -- |
| *Draco maximus* | AB023740 | AB023760 | -- | -- | -- | AF288231 | -- | -- | -- | -- | -- | -- |
| *Draco melanopogon* | AB023742 | AB023762 | -- | -- | -- | AF288258 | -- | -- | -- | -- | -- | -- |
| *Draco mindanensis* | -- | -- | -- | -- | -- | AF288249 | -- | -- | -- | -- | -- | -- |
| *Draco obscurus* | AB023744 | AB023764 | -- | -- | -- | AF288250 | -- | -- | -- | -- | -- | -- |
| *Draco ornatus* | -- | -- | -- | -- | -- | AF288253 | -- | -- | -- | -- | -- | -- |
| *Draco palawanensis* | -- | -- | -- | -- | -- | AF288262 | -- | -- | -- | -- | -- | -- |
| *Draco quadrasi* | -- | -- | -- | -- | -- | AF288268 | -- | -- | -- | -- | -- | -- |
| *Draco quinquefasciatus* | AB023745 | AB023766 | -- | -- | -- | AF288232 | -- | -- | -- | -- | -- | -- |
| *Draco reticulatus* | -- | -- | -- | -- | -- | AF288247 | -- | -- | -- | -- | -- | -- |
| *Draco rhytisma* | -- | -- | -- | -- | -- | AF288280 | -- | -- | -- | -- | -- | -- |
| *Draco spilonotus* | -- | -- | -- | -- | -- | AF288282 | -- | -- | -- | -- | -- | -- |
| *Draco spilopterus* | -- | -- | -- | -- | -- | AF288240 | -- | -- | -- | -- | -- | -- |
| *Draco taeniopterus* | AB023747 | AB023767 | -- | -- | -- | AF288251 | -- | -- | -- | -- | -- | -- |
| *Draco timorensis* | -- | -- | -- | -- | -- | AF288275 | -- | -- | -- | -- | -- | -- |
| *Draco volans* | AB023731 | AB023770 | -- | -- | -- | AF288267 | AF443226 | -- | -- | -- | -- | -- |
| *Drepanoides anomalus* | GQ457791 | GQ457732 | -- | GQ895810 | GQ895866 | -- | -- | -- | -- | -- | -- | -- |
| *Dromicodryas bernieri* | FJ404151 | AF215268 | -- | AY187975 | AY188014 | -- | -- | -- | -- | -- | -- | FJ404425 |
| *Dromicodryas quadrilineatus* | -- | AY188054 | -- | AY187976 | AY188015 | -- | -- | -- | -- | -- | -- | -- |
| *Drymarchon corais* | HM565758 | -- | -- | AF471137 | AF471064 | DQ902207 | DQ902314 | -- | -- | -- | -- | -- |
| *Drymobius rhombifer* | HM565761 | -- | -- | GQ927313 | GQ927320 | -- | -- | -- | -- | -- | -- | -- |
| *Drymoluber brazili* | HM565760 | -- | -- | -- | -- | -- | -- | -- | -- | -- | -- | -- |
| *Drymoluber dichrous* | HM565759 | -- | -- | GQ895812 | GQ895869 | -- | -- | -- | -- | -- | -- | -- |
| *Dryocalamus nympha* | KC347323 | KC347360 | -- | KC347400 | KC347477 | -- | KC347513 | -- | -- | -- | KC347437 | -- |
| *Drysdalia coronoides* | EU547124 | EU547173 | -- | -- | -- | -- | EU547027 | -- | -- | -- | EU546898 | -- |
| *Drysdalia mastersii* | EU547125 | EU547174 | -- | EU546938 | -- | -- | EU547028 | -- | -- | -- | EU546899 | -- |
| *Duberria lutrix* | FJ404154 | FJ404207 | -- | DQ486161 | DQ486337 | -- | FJ404356 | -- | -- | -- | -- | FJ404428 |
| *Duberria variegata* | FJ404155 | FJ404208 | -- | DQ486165 | DQ486341 | -- | FJ404357 | -- | -- | -- | -- | FJ404429 |
| *Ebenavia inunguis* | EU596608 | GU129011 | EU596842 | FJ830144 | FJ830053 | EF536191 | EF536215 | -- | HQ426191 | -- | EF536143 | HQ426453 |
| *Echinanthera melanostigma* | JQ598806 | JQ598869 | -- | -- | JQ598928 | -- | -- | -- | -- | -- | -- | -- |
| *Echinanthera undulata* | JQ598807 | JQ598870 | JQ599022 | JQ598978 | JQ598929 | -- | -- | -- | -- | -- | -- | -- |
| *Echiopsis atriceps* | EU547129 | EU547178 | -- | EU546942 | -- | -- | EU547032 | -- | -- | -- | EU546903 | -- |
| *Echiopsis curta* | EU547121 | EU547170 | -- | -- | -- | -- | EU547024 | -- | -- | -- | EU546895 | -- |
| *Echis carinatus* | GQ359605 | GQ359690 | -- | -- | -- | -- | EU852301 | -- | -- | -- | EU852325 | -- |
| *Echis coloratus* | EU852315 | HQ658452 | -- | -- | -- | -- | EU852303 | -- | -- | -- | EU852327 | -- |
| *Echis jogeri* | GQ359648 | GQ359732 | -- | -- | GQ359476 | -- | GQ359560 | -- | -- | -- | -- | -- |
| *Echis leucogaster* | GQ359624 | EU642577 | -- | -- | -- | -- | -- | -- | -- | -- | -- | -- |
| *Echis ocellatus* | GQ359589 | GQ359681 | -- | -- | AF292568 | -- | AF292607 | -- | -- | -- | EU852324 | -- |
| *Echis omanensis* | GQ359637 | EU642581 | -- | -- | -- | -- | -- | -- | -- | -- | -- | -- |
| *Echis pyramidum* | GQ359609 | GQ359724 | -- | -- | -- | -- | EU624226 | -- | -- | -- | EU852326 | -- |
| *Ecpleopus gaudichaudii* | AF420660 | AF420738 | -- | AF420855 | -- | -- | AF420901 | -- | -- | -- | -- | -- |
| *Egernia depressa* | -- | -- | -- | -- | -- | -- | JF813065 | -- | -- | -- | -- | -- |
| *Egernia guthega* | -- | AY520503 | -- | -- | -- | -- | AY612998 | -- | -- | -- | -- | -- |
| *Egernia hosmeri* | -- | -- | -- | -- | -- | -- | JF813076 | -- | -- | -- | -- | -- |
| *Egernia inornata* | -- | AY520498 | -- | -- | -- | -- | AY612897 | -- | -- | -- | -- | -- |
| *Egernia kingii* | -- | -- | -- | -- | -- | -- | JF813008 | -- | -- | -- | -- | -- |
| *Egernia kintorei* | -- | AY520500 | -- | -- | -- | -- | AY520468 | -- | -- | -- | -- | -- |
| *Egernia luctuosa* | -- | -- | -- | AF039463 | -- | -- | -- | -- | -- | -- | -- | -- |
| *Egernia margaretae* | -- | AY520507 | -- | -- | -- | -- | AY520483 | -- | -- | -- | -- | -- |
| *Egernia modesta* | -- | AY520509 | -- | -- | -- | -- | AY613068 | -- | -- | -- | -- | -- |
| *Egernia montana* | -- | AY520502 | -- | -- | -- | -- | AY612993 | -- | -- | -- | -- | -- |
| *Egernia multiscutata* | -- | AY520505 | -- | -- | -- | -- | AY612985 | -- | -- | -- | -- | -- |
| *Egernia napoleonis* | -- | -- | -- | -- | -- | -- | JF813075 | -- | -- | -- | -- | -- |
| *Egernia pulchra* | -- | AY520499 | -- | -- | -- | -- | AY520467 | -- | -- | -- | -- | -- |
| *Egernia richardi* | -- | -- | -- | -- | -- | -- | JF813069 | -- | -- | -- | -- | -- |
| *Egernia saxatilis* | -- | AY520495 | -- | -- | -- | -- | AY520463 | -- | -- | -- | -- | -- |
| *Egernia stokesii* | -- | -- | HQ655166 | HQ655203 | -- | -- | JF813095 | -- | -- | -- | -- | -- |
| *Egernia striata* | -- | AY520501 | -- | -- | -- | -- | -- | -- | -- | -- | -- | -- |
| *Egernia whitii* | AY649109 | AY649150 | -- | AY818774 | AF280130 | -- | AY169640 | -- | -- | -- | -- | -- |
| *Eirenis aurolineatus* | -- | AY376778 | -- | AY376807 | AY376749 | AY487031 | AY487070 | -- | -- | -- | -- | -- |
| *Eirenis barani* | -- | AY376785 | -- | AY376822 | AY376764 | -- | -- | -- | -- | -- | -- | -- |
| *Eirenis collaris* | -- | AY376795 | -- | AY376824 | AY376766 | -- | -- | -- | -- | -- | -- | -- |
| *Eirenis coronelloides* | -- | AY376787 | -- | AY376816 | AY376758 | -- | -- | -- | -- | -- | -- | -- |
| *Eirenis decemlineatus* | -- | AY376789 | -- | AY376818 | AY376760 | -- | -- | -- | -- | -- | -- | -- |
| *Eirenis eiselti* | -- | AY376776 | -- | AY376805 | AY376747 | AY487030 | AY487069 | -- | -- | -- | -- | -- |
| *Eirenis levantinus* | -- | AY376794 | -- | AY376823 | AY376765 | AY487032 | AY487071 | -- | -- | -- | -- | -- |
| *Eirenis lineomaculatus* | -- | AY376791 | -- | AY376820 | AY376762 | -- | -- | -- | -- | -- | -- | -- |
| *Eirenis medus* | AY647226 | AY376796 | -- | AY376825 | AY376767 | -- | -- | -- | -- | -- | -- | -- |
| *Eirenis modestus* | AY039143 | AY376792 | -- | AY486957 | AY486933 | AY487033 | AY487072 | -- | -- | -- | -- | -- |
| *Eirenis punctatolineatus* | AY647227 | AY376781 | -- | AY376813 | AY376755 | AY487034 | AY487073 | -- | -- | -- | -- | -- |
| *Eirenis rothii* | -- | -- | -- | AY376817 | AY376759 | -- | -- | -- | -- | -- | -- | -- |
| *Eirenis thospitis* | -- | AY376790 | -- | AY376819 | AY376761 | -- | -- | -- | -- | -- | -- | -- |
| *Elaphe bimaculata* | AY122767 | -- | -- | DQ902062 | DQ902104 | DQ902210 | DQ902283 | -- | -- | -- | -- | -- |
| *Elaphe carinata* | AY122839 | HM439983 | -- | DQ902063 | DQ902133 | DQ902211 | DQ902284 | -- | -- | -- | -- | -- |
| *Elaphe climacophora* | AY122772 | -- | -- | DQ902064 | DQ902105 | DQ902212 | DQ902285 | -- | -- | -- | -- | -- |
| *Elaphe davidi* | AY122775 | -- | -- | -- | -- | -- | -- | -- | -- | -- | -- | -- |
| *Elaphe dione* | AF236673 | -- | -- | DQ902066 | DQ902107 | DQ902214 | DQ902287 | -- | -- | -- | -- | -- |
| *Elaphe quadrivirgata* | AY122793 | -- | -- | DQ902078 | DQ902120 | DQ902228 | DQ902300 | -- | -- | -- | -- | -- |
| *Elaphe quatuorlineata* | AY122796 | AF215267 | -- | AY486955 | AY122714 | AY487028 | AY487067 | -- | -- | -- | -- | -- |
| *Elaphe rufodorsata* | -- | -- | -- | DQ902081 | DQ902123 | DQ902232 | DQ902301 | -- | -- | -- | -- | -- |
| *Elaphe sauromates* | AY122797 | -- | -- | -- | -- | -- | -- | -- | -- | -- | -- | -- |
| *Elaphe schrenckii* | AF236672 | -- | -- | DQ902082 | AY122720 | DQ902233 | DQ902302 | -- | -- | -- | -- | -- |
| *Elapognathus coronata* | EU547118 | EU547167 | -- | EU546931 | -- | -- | EU547021 | -- | -- | -- | EU546892 | -- |
| *Elapomorphus quinquelineatus* | GQ457794 | GQ457735 | JQ599023 | GQ457855 | JQ598930 | -- | -- | -- | -- | -- | -- | -- |
| *Elapsoidea nigra* | U96804 | -- | -- | AY058930 | AF217820 | -- | -- | -- | -- | -- | -- | -- |
| *Elapsoidea semiannulata* | AF544745 | JF357946 | FJ433987 | AF544678 | AF039260 | -- | JF357928 | FJ434088 | -- | EF144071 | AY487373 | EF144098 |
| *Elapsoidea sundevallii* | -- | AY188055 | -- | AY187977 | AY188016 | -- | -- | -- | -- | -- | -- | -- |
| *Elasmodactylus tetensis* | DQ275407 | AF449118 | -- | JQ945559 | AY026926 | JX041349 | -- | -- | JQ945376 | -- | DQ275451 | JQ945452 |
| *Elasmodactylus tuberculosus* | DQ275408 | AF449119 | -- | -- | AF449134 | -- | -- | -- | -- | -- | DQ275452 | -- |
| *Elgaria coerulea* | -- | -- | JQ845042 | -- | -- | AF085617 | -- | JQ844949 | -- | -- | -- | -- |
| *Elgaria kingii* | AY525103 | -- | -- | -- | AY525100 | AY742921 | AY605103 | -- | HQ426252 | -- | -- | HQ426514 |
| *Elgaria multicarinata* | AY649110 | AY649151 | GU457854 | AF039479 | AF361522 | AF085620 | DQ364661 | GU456010 | -- | HQ876338 | GU457977 | -- |
| *Elgaria panamintina* | -- | -- | -- | -- | -- | U82692 | -- | -- | -- | -- | AY662603 | -- |
| *Elgaria paucicarinata* | -- | -- | -- | -- | -- | AF085619 | DQ364662 | -- | -- | -- | -- | -- |
| *Emoia atrocostata* | JF497856 | JF497979 | HQ907222 | -- | -- | -- | JF498461 | -- | -- | HQ907627 | -- | -- |
| *Emoia caeruleocauda* | AY218012 | AY217962 | HM160585 | AY217859 | U20454 | JF498109 | JF498462 | -- | -- | HM161059 | HM161154 | -- |
| *Emoia concolor* | -- | -- | -- | -- | AF151657 | -- | -- | -- | -- | -- | -- | -- |
| *Emoia cyanogaster* | JF497858 | JF497981 | -- | -- | -- | JF498110 | JF498463 | -- | -- | JF498337 | -- | -- |
| *Emoia cyanura* | AY218018 | AY308191 | -- | AY217865 | U49345 | DQ675263 | JF498464 | -- | -- | JF498338 | DQ675343 | -- |
| *Emoia impar* | AY818740 | -- | -- | AY818777 | U49340 | -- | -- | -- | -- | -- | -- | -- |
| *Emoia isolata* | -- | -- | -- | -- | U49333 | -- | -- | -- | -- | -- | -- | -- |
| *Emoia jakati* | AY218008 | AY217958 | -- | AY217855 | AY217804 | -- | -- | -- | -- | -- | -- | -- |
| *Emoia loyaltiensis* | -- | -- | -- | DQ675411 | -- | DQ675206 | -- | -- | -- | -- | DQ675286 | -- |
| *Emoia physicae* | AY169566 | AY169603 | -- | AY818778 | AY818811 | -- | AY169641 | -- | -- | -- | -- | -- |
| *Emoia pseudocyanura* | -- | -- | -- | -- | U49334 | -- | -- | -- | -- | -- | -- | -- |
| *Emoia schmidti* | JF497860 | JF497983 | -- | -- | -- | -- | JF498465 | -- | -- | JF498339 | -- | -- |
| *Emoia tongana* | -- | -- | -- | -- | AF151662 | -- | -- | -- | -- | -- | -- | -- |
| *Emydocephalus annulatus* | EU547136 | EU547185 | -- | -- | DQ233942 | -- | EU547038 | -- | -- | -- | EU546908 | -- |
| *Enhydrina schistosa* | -- | FJ587210 | -- | -- | -- | -- | -- | -- | -- | -- | -- | -- |
| *Enhydris bocourti* | AF499279 | EF395853 | -- | EF395927 | EF395902 | -- | -- | -- | -- | -- | -- | -- |
| *Enhydris chinensis* | AF499280 | EF395854 | -- | EF395928 | EF395903 | -- | GU997184 | -- | -- | -- | -- | -- |
| *Enhydris enhydris* | EF395879 | EF395855 | -- | EF395929 | EF395904 | -- | GU997191 | -- | -- | -- | -- | -- |
| *Enhydris innominata* | EF395880 | EF395856 | -- | EF395930 | GU997199 | -- | GU997185 | -- | -- | -- | -- | -- |
| *Enhydris jagorii* | AF499284 | AF499298 | -- | -- | GU997207 | -- | GU997193 | -- | -- | -- | -- | -- |
| *Enhydris longicauda* | EF395882 | EF395858 | -- | EF395932 | EF395907 | -- | GU997189 | -- | -- | -- | -- | -- |
| *Enhydris matannensis* | AF499281 | EF395859 | -- | EF473654 | EF395908 | -- | -- | -- | -- | -- | -- | -- |
| *Enhydris plumbea* | NC_010200 | EF395861 | -- | EF395934 | EF395910 | NC_010200 | U49328 | -- | -- | -- | -- | -- |
| *Enhydris polylepis* | EF395886 | EF395862 | -- | EF473655 | EF395911 | -- | -- | -- | -- | -- | -- | -- |
| *Enhydris punctata* | EF395887 | EF395863 | -- | EF395935 | EF395912 | -- | -- | -- | -- | -- | -- | -- |
| *Enyalioides heterolepis* | -- | -- | EU586759 | -- | -- | EU586746 | -- | -- | -- | -- | EU586771 | -- |
| *Enyalioides laticeps* | -- | -- | EU586761 | -- | -- | EU586748 | U66226 | GU456004 | -- | JF804590 | EU586773 | -- |
| *Enyalioides microlepis* | -- | -- | EU586763 | -- | -- | EU586750 | -- | -- | -- | -- | EU586774 | -- |
| *Enyalioides oshaughnessyi* | -- | -- | EU586764 | -- | -- | EU586753 | -- | -- | -- | -- | EU586775 | -- |
| *Enyalioides palpebralis* | -- | -- | EU586765 | -- | -- | EU586754 | -- | -- | -- | -- | EU586776 | -- |
| *Enyalioides praestabilis* | -- | -- | EU586766 | -- | -- | EU586756 | -- | -- | -- | -- | EU586777 | -- |
| *Enyalius bilineatus* | AF338340 | -- | -- | -- | -- | -- | -- | -- | -- | -- | -- | -- |
| *Enyalius leechii* | AF338342 | -- | -- | -- | -- | AF528733 | -- | -- | -- | -- | -- | -- |
| *Ephalophis greyae* | -- | FJ587209 | -- | -- | -- | -- | -- | -- | -- | -- | -- | -- |
| *Epicrates angulifer* | -- | -- | -- | HQ399542 | HQ399513 | -- | -- | -- | -- | -- | HQ399523 | -- |
| *Epicrates cenchria* | AF368059 | AF215273 | AY988028 | HQ399538 | U69777 | -- | -- | -- | -- | -- | AY988062 | -- |
| *Epicrates chrysogaster* | -- | -- | -- | -- | U69780 | -- | -- | JF812171 | -- | -- | -- | -- |
| *Epicrates exsul* | -- | -- | -- | -- | U69782 | -- | -- | -- | -- | -- | -- | -- |
| *Epicrates fordi* | -- | -- | -- | -- | U69784 | -- | -- | -- | -- | -- | -- | -- |
| *Epicrates inornatus* | -- | -- | -- | -- | U69787 | -- | -- | -- | -- | -- | -- | -- |
| *Epicrates monensis* | -- | -- | -- | -- | U69792 | -- | -- | -- | -- | -- | -- | -- |
| *Epicrates striatus* | -- | -- | DQ465555 | DQ465553 | U69791 | -- | -- | EU390918 | -- | HQ876358 | EU402844 | -- |
| *Epicrates subflavus* | -- | -- | -- | -- | U69803 | -- | L08098 | -- | -- | -- | -- | -- |
| *Eremias argus* | AF217082 | DQ658831 | -- | -- | HM120813 | -- | -- | -- | -- | -- | -- | -- |
| *Eremias arguta* | DQ658805 | DQ658824 | -- | EF632258 | GQ142115 | -- | -- | -- | -- | -- | EF632213 | -- |
| *Eremias brenchleyi* | EF490071 | DQ658833 | -- | -- | NC_011764 | NC_011764 | NC_011764 | -- | -- | -- | -- | -- |
| *Eremias grammica* | -- | DQ494822 | -- | -- | -- | U71331 | -- | -- | -- | -- | -- | -- |
| *Eremias montanus* | FJ445368 | -- | -- | -- | -- | -- | -- | -- | -- | -- | -- | -- |
| *Eremias multiocellata* | DQ658793 | DQ658839 | -- | -- | -- | -- | -- | -- | -- | -- | -- | -- |
| *Eremias nigrolateralis* | FJ445374 | -- | -- | -- | FJ416291 | -- | -- | -- | -- | -- | -- | -- |
| *Eremias persica* | FJ445308 | -- | -- | -- | FJ416281 | -- | -- | -- | -- | -- | -- | -- |
| *Eremias pleskei* | AY035828 | AY035838 | -- | EF632259 | -- | -- | -- | -- | -- | -- | EF632214 | -- |
| *Eremias przewalskii* | -- | DQ494825 | -- | -- | -- | -- | -- | -- | -- | -- | -- | -- |
| *Eremias velox* | DQ658801 | DQ658845 | -- | -- | FJ416175 | -- | -- | -- | -- | -- | -- | -- |
| *Eremias vermiculata* | -- | DQ494831 | -- | -- | -- | -- | -- | -- | -- | -- | -- | -- |
| *Eremiascincus fasciolatus* | AY308341 | AY308192 | -- | -- | -- | -- | -- | -- | -- | -- | -- | -- |
| *Eremiascincus richardsonii* | AY308342 | AY308193 | HQ655167 | HQ655204 | -- | -- | AY169657 | -- | -- | -- | -- | -- |
| *Eristicophis macmahoni* | EU624259 | EU624293 | -- | -- | AJ275711 | -- | EU624227 | -- | -- | -- | -- | -- |
| *Erpeton tentaculatum* | AF544766 | EF395864 | -- | EF395936 | EF395913 | -- | -- | -- | -- | -- | -- | -- |
| *Erythrolamprus aesculapii* | GQ457795 | GQ457736 | -- | GQ895814 | GQ895871 | -- | -- | -- | -- | -- | -- | -- |
| *Erythrolamprus mimus* | GU018157 | GU018175 | -- | -- | -- | -- | -- | -- | -- | -- | -- | -- |
| *Eryx elegans* | -- | -- | -- | -- | U69818 | -- | -- | -- | -- | -- | -- | -- |
| *Eryx jaculus* | -- | -- | -- | -- | U69820 | -- | -- | -- | -- | -- | -- | -- |
| *Eryx jayakari* | HQ658419 | -- | DQ465566 | DQ465565 | -- | -- | -- | -- | -- | -- | DQ465567 | -- |
| *Eryx johnii* | -- | -- | DQ465576 | AY099975 | U69823 | -- | -- | DQ465575 | -- | -- | DQ465577 | -- |
| *Eryx miliaris* | AF544746 | -- | FJ433977 | AF544683 | U69824 | -- | AF302942 | -- | -- | FJ433931 | AY487393 | FJ433907 |
| *Eryx tataricus* | AF236681 | -- | -- | AF435018 | U69830 | -- | -- | -- | -- | -- | -- | -- |
| *Eublepharis macularius* | AF090186 | AB028762 | GU457864 | EU366458 | -- | AB308467 | -- | GU456020 | EF534816 | HQ876372 | EF534776 | EF534942 |
| *Eublepharis turcmenicus* | -- | -- | -- | -- | -- | AF114248 | -- | -- | -- | -- | AY662622 | -- |
| *Eugongylus albofasciolatus* | -- | -- | -- | -- | AF373232 | AF373262 | -- | -- | -- | -- | -- | -- |
| *Eugongylus rufescens* | AY169567 | AY649152 | HQ655168 | AY818779 | AY217807 | DQ675253 | AY169642 | JN568336 | -- | JN568485 | JN654859 | -- |
| *Eulamprus amplus* | AY169583 | AY169620 | -- | AY818791 | AJ406248 | -- | AY169658 | -- | -- | -- | -- | -- |
| *Eulamprus brachyosoma* | -- | -- | -- | -- | -- | -- | AF530236 | -- | -- | -- | -- | -- |
| *Eulamprus frerei* | -- | AF530204 | -- | -- | -- | -- | AF530241 | -- | -- | -- | -- | -- |
| *Eulamprus heatwolei* | -- | AF530222 | -- | -- | -- | -- | AF530259 | -- | -- | -- | -- | -- |
| *Eulamprus kosciuskoi* | DQ915292 | DQ915316 | -- | -- | -- | -- | DQ915340 | -- | -- | -- | -- | -- |
| *Eulamprus leuraensis* | -- | AF530225 | -- | -- | -- | -- | AF530262 | -- | -- | -- | -- | -- |
| *Eulamprus luteilateralis* | DQ915295 | DQ915319 | -- | -- | -- | -- | DQ915343 | -- | -- | -- | -- | -- |
| *Eulamprus martini* | -- | AF530192 | -- | -- | -- | -- | AF530229 | -- | -- | -- | -- | -- |
| *Eulamprus murrayi* | AY169584 | AY169621 | -- | -- | -- | -- | AY169659 | -- | -- | -- | -- | -- |
| *Eulamprus quoyii* | AY169585 | AY169622 | -- | -- | -- | -- | AY169660 | -- | -- | -- | -- | -- |
| *Eulamprus sokosoma* | -- | AF530196 | -- | -- | -- | -- | AF530233 | -- | -- | -- | -- | -- |
| *Eulamprus tenuis* | DQ915305 | AY308194 | -- | -- | -- | -- | DQ915353 | -- | -- | -- | -- | -- |
| *Eulamprus tigrinus* | -- | AF530194 | -- | -- | -- | -- | AF530231 | -- | -- | -- | -- | -- |
| *Eulamprus tryoni* | -- | AF530206 | -- | -- | -- | -- | AF530243 | -- | -- | -- | -- | -- |
| *Eulamprus tympanum* | -- | AF530217 | -- | -- | -- | -- | AF530254 | -- | -- | -- | -- | -- |
| *Euleptes europaea* | -- | -- | -- | EF534932 | -- | JN393941 | -- | -- | EF534848 | -- | EF534806 | EF534974 |
| *Eumeces algeriensis* | AY308345 | AY308196 | -- | -- | -- | -- | -- | -- | -- | -- | -- | -- |
| *Eumeces schneideri* | AY315506 | AY308213 | HM160586 | -- | -- | -- | -- | -- | -- | HM161060 | HM161155 | -- |
| *Eumecia anchietae* | AY308367 | AY308218 | -- | -- | -- | -- | -- | -- | -- | -- | -- | -- |
| *Eunectes murinus* | -- | -- | -- | AY099964 | U69808 | -- | -- | -- | -- | -- | -- | -- |
| *Eunectes notaeus* | AM236347 | AF215274 | AY988029 | -- | U69810 | AM236347 | AM236347 | -- | -- | -- | AY988063 | -- |
| *Euprepiophis conspicillata* | -- | -- | -- | DQ902065 | DQ902106 | DQ902213 | DQ902286 | -- | -- | -- | -- | -- |
| *Euprepiophis mandarina* | -- | -- | -- | DQ902073 | DQ902115 | DQ902222 | DQ902294 | -- | -- | -- | -- | -- |
| *Eurolophosaurus amathites* | AF362525 | DQ848778 | -- | -- | DQ848731 | -- | -- | -- | -- | -- | -- | -- |
| *Eurolophosaurus divaricatus* | AF362529 | DQ848777 | -- | -- | -- | -- | -- | -- | -- | -- | -- | -- |
| *Eurolophosaurus nanuzae* | AF362537 | DQ848746 | -- | -- | -- | -- | -- | -- | -- | -- | -- | -- |
| *Eurydactylodes agricolae* | -- | GU460147 | -- | JQ945560 | -- | DQ533758 | -- | -- | GU459749 | -- | GU459547 | JQ945453 |
| *Eurydactylodes occidentalis* | -- | -- | -- | -- | -- | DQ533776 | -- | -- | -- | -- | -- | -- |
| *Eurydactylodes symmetricus* | -- | -- | -- | -- | -- | DQ533743 | -- | -- | -- | -- | -- | -- |
| *Eurydactylodes vieillardi* | -- | -- | -- | -- | -- | DQ533774 | -- | -- | -- | -- | -- | -- |
| *Eurylepis taeniolatus* | -- | -- | HM160587 | -- | HM921206 | -- | -- | -- | -- | HM161061 | HM161156 | -- |
| *Eutropis beddomii* | JQ767970 | JQ767965 | -- | JQ767946 | -- | -- | -- | -- | -- | -- | -- | -- |
| *Eutropis bibronii* | JQ767979 | JQ767963 | -- | JQ767947 | -- | -- | -- | -- | -- | -- | -- | -- |
| *Eutropis clivicola* | JQ767978 | JQ767956 | -- | JQ767945 | -- | -- | -- | -- | -- | -- | -- | -- |
| *Eutropis cumingi* | DQ239218 | DQ238896 | -- | DQ238977 | DQ239137 | -- | -- | -- | -- | -- | -- | -- |
| *Eutropis longicaudata* | -- | -- | -- | EU366457 | DQ239139 | -- | AY169645 | -- | -- | -- | -- | -- |
| *Eutropis macularia* | AF153557 | AF153573 | -- | DQ238976 | AF153590 | -- | AF228556 | -- | -- | -- | -- | -- |
| *Eutropis multicarinata* | AY159052 | AY159081 | -- | DQ675394 | -- | -- | -- | -- | -- | -- | DQ675339 | -- |
| *Eutropis multifasciata* | AY159054 | AF153576 | -- | DQ238978 | AY151513 | JF498112 | JF498466 | -- | -- | JF498340 | AY444055 | -- |
| *Eutropis nagarjuni* | JQ767972 | JQ767952 | -- | JQ767949 | -- | -- | -- | -- | -- | -- | -- | -- |
| *Eutropis rudis* | -- | -- | -- | DQ238975 | DQ239135 | -- | -- | -- | -- | -- | -- | -- |
| *Eutropis trivittata* | JQ767971 | JQ767951 | -- | JQ767948 | -- | -- | -- | -- | -- | -- | -- | -- |
| *Exiliboa placata* | AF512742 | AF512742 | AY988034 | AY099973 | AY099989 | -- | -- | EU390920 | -- | JN703061 | EU402846 | -- |
| *Farancia abacura* | AY577016 | AY577025 | -- | AF471141 | U69832 | DQ902239 | U49307 | -- | -- | -- | -- | -- |
| *Farancia erytrogramma* | AY577017 | AY577026 | -- | -- | -- | -- | -- | -- | -- | -- | -- | -- |
| *Feylinia currori* | AY308369 | AY308220 | -- | -- | -- | -- | -- | -- | -- | -- | -- | -- |
| *Feylinia grandisquamis* | DQ316879 | AY217952 | -- | AY217850 | DQ316871 | -- | -- | -- | -- | -- | -- | -- |
| *Feylinia polylepis* | AY649120 | AY649161 | GU457871 | AY662571 | FJ972225 | AY662556 | -- | GU456027 | -- | JN568493 | AY662637 | -- |
| *Ficimia streckeri* | -- | -- | -- | GQ927314 | -- | -- | -- | -- | -- | -- | -- | -- |
| *Fordonia leucobalia* | EF395889 | EF395866 | -- | EF395938 | EF395915 | -- | -- | -- | -- | -- | -- | -- |
| *Furcifer angeli* | -- | -- | -- | -- | -- | -- | AF443274 | -- | -- | -- | -- | -- |
| *Furcifer antimena* | -- | FJ717754 | -- | -- | -- | -- | -- | -- | -- | -- | -- | -- |
| *Furcifer balteatus* | -- | HQ130529 | -- | FJ984264 | -- | AF448764 | FJ981757 | -- | -- | -- | HQ130638 | -- |
| *Furcifer belalandaensis* | -- | -- | -- | FJ984270 | -- | AF448765 | FJ981760 | -- | -- | -- | -- | -- |
| *Furcifer bifidus* | -- | -- | -- | -- | -- | -- | AF443272 | -- | -- | -- | -- | -- |
| *Furcifer campani* | -- | EF210578 | -- | FJ984269 | -- | AF448766 | AF443271 | -- | -- | -- | -- | -- |
| *Furcifer cephalolepis* | -- | -- | JQ073093 | FJ984266 | -- | FJ975155 | FJ981758 | -- | -- | -- | JQ073214 | -- |
| *Furcifer labordi* | AF215138 | AF215264 | -- | FJ984265 | -- | AF448767 | AF443273 | -- | -- | -- | -- | -- |
| *Furcifer lateralis* | -- | HQ130531 | JQ073087 | FJ984272 | -- | AF448768 | AF443267 | -- | -- | -- | JQ073208 | -- |
| *Furcifer minor* | -- | AF378380 | -- | -- | -- | -- | AF443268 | -- | -- | -- | -- | -- |
| *Furcifer oustaleti* | AB185326 | NC_008777 | -- | FJ984271 | NC_008777 | AF448769 | AF443269 | -- | -- | -- | -- | -- |
| *Furcifer pardalis* | -- | -- | -- | FJ984268 | -- | FJ975157 | AF443265 | -- | -- | -- | -- | -- |
| *Furcifer petteri* | -- | -- | -- | -- | -- | -- | AF443266 | -- | -- | -- | -- | -- |
| *Furcifer polleni* | -- | -- | JQ073094 | FJ984267 | -- | FJ975156 | FJ981759 | -- | -- | -- | JQ073215 | -- |
| *Furcifer verrucosus* | -- | FJ717755 | -- | -- | -- | AF448770 | AF443270 | -- | -- | -- | -- | -- |
| *Furcifer willsii* | -- | HQ130530 | -- | HQ130551 | -- | HQ130654 | HQ130577 | -- | -- | -- | HQ130640 | -- |
| *Furina diadema* | EU547102 | EU547151 | -- | EU546917 | -- | -- | EU547008 | -- | -- | -- | EU546878 | -- |
| *Furina ornata* | EU547103 | EU547152 | -- | -- | -- | -- | EU547009 | -- | -- | -- | EU546879 | -- |
| *Gallotia atlantica* | Z48040 | DQ298679 | JQ073130 | AY151999 | AY762456 | AY762534 | -- | -- | -- | -- | JQ073253 | -- |
| *Gallotia caesaris* | AF439943 | DQ298684 | -- | AY152006 | AY154903 | -- | -- | -- | -- | -- | -- | -- |
| *Gallotia galloti* | AF206587 | GQ142091 | JQ073131 | AF315394 | GQ142114 | -- | -- | -- | -- | -- | EF632215 | -- |
| *Gallotia gomerana* | AJ272395 | -- | -- | AF435103 | AJ272396 | -- | -- | -- | -- | -- | -- | -- |
| *Gallotia intermedia* | AY154913 | DQ298681 | -- | AY152007 | AY151844 | -- | -- | -- | -- | -- | -- | -- |
| *Gallotia simonyi* | Z49751 | AF101208 | -- | AY152008 | Z49752 | -- | -- | -- | -- | -- | -- | -- |
| *Gallotia stehlini* | AF439944 | DQ298688 | JQ073132 | AY152001 | AY154899 | -- | -- | -- | -- | -- | JQ073255 | -- |
| *Gambelia copeii* | -- | -- | -- | -- | EU037375 | EU038413 | -- | -- | -- | -- | -- | -- |
| *Gambelia sila* | -- | -- | -- | -- | EU037370 | EU038401 | -- | -- | -- | -- | -- | -- |
| *Gambelia wislizenii* | NC_012831 | AY217944 | EU108358 | -- | EU116511 | NC_012831 | NC_012831 | JF804548 | -- | HQ876327 | AY662600 | -- |
| *Garthius chaseni* | AY352791 | AY352729 | -- | -- | AY352760 | -- | AY352825 | -- | -- | -- | -- | -- |
| *Gastropholis prasina* | -- | HQ605781 | -- | HQ605877 | HQ605835 | -- | -- | -- | -- | -- | HQ616543 | -- |
| *Gastropholis vittata* | -- | HQ605780 | -- | HQ605876 | HQ605834 | -- | -- | -- | -- | -- | HQ616542 | -- |
| *Geckoella triedrus* | DQ852719 | -- | -- | DQ852733 | -- | JX041352 | -- | -- | JQ945377 | -- | JQ945308 | JQ945454 |
| *Geckolepis maculata* | EU596610 | -- | EU596844 | JQ945562 | -- | EU054235 | EU054163 | -- | EU054187 | -- | EU054211 | JQ945455 |
| *Geckolepis typica* | -- | -- | -- | -- | -- | EU054233 | EU054161 | -- | EU054185 | -- | EU054209 | -- |
| *Geckonia chazaliae* | AF363574 | JQ300797 | -- | AF363556 | AF364326 | JX041443 | -- | -- | EU293705 | -- | EU293638 | JQ301343 |
| *Gehyra australis* | -- | -- | -- | JQ945563 | -- | GQ257759 | -- | -- | JN019113 | -- | -- | JQ945456 |
| *Gehyra baliola* | -- | -- | -- | -- | -- | GQ257783 | -- | -- | -- | -- | -- | -- |
| *Gehyra barea* | -- | -- | -- | -- | -- | JN393915 | -- | -- | JN393993 | -- | JN393960 | -- |
| *Gehyra borroloola* | -- | -- | -- | -- | -- | GQ257755 | -- | -- | -- | -- | -- | -- |
| *Gehyra brevipalmata* | -- | -- | -- | -- | -- | JN393910 | -- | -- | JN393987 | -- | JN393955 | -- |
| *Gehyra catenata* | -- | -- | -- | -- | -- | GQ257787 | -- | -- | -- | -- | -- | -- |
| *Gehyra dubia* | -- | -- | -- | JQ945565 | -- | GQ257788 | -- | -- | JN393989 | -- | -- | JQ945458 |
| *Gehyra fehlmanni* | -- | -- | -- | -- | -- | JN393948 | -- | -- | -- | -- | JN393986 | -- |
| *Gehyra koira* | -- | -- | -- | -- | -- | GQ257786 | -- | -- | -- | -- | -- | -- |
| *Gehyra lacerata* | -- | -- | -- | -- | -- | JN393947 | -- | -- | -- | -- | JN393985 | -- |
| *Gehyra marginata* | -- | -- | -- | -- | -- | JN393931 | -- | -- | JN394009 | -- | JN393975 | -- |
| *Gehyra membranacruralis* | -- | -- | -- | -- | -- | GQ257761 | -- | -- | -- | -- | -- | -- |
| *Gehyra minuta* | -- | -- | -- | -- | -- | GQ257753 | -- | -- | -- | -- | -- | -- |
| *Gehyra montium* | -- | -- | -- | -- | -- | GQ257801 | -- | -- | -- | -- | -- | -- |
| *Gehyra mutilata* | DQ852699 | AY217956 | -- | FJ830146 | FJ830054 | GQ257784 | -- | -- | JN019114 | -- | FJ830237 | FJ830328 |
| *Gehyra nana* | -- | -- | -- | JQ945567 | -- | GQ257760 | -- | -- | JN393998 | -- | -- | JQ945460 |
| *Gehyra occidentalis* | -- | -- | -- | -- | -- | GQ257754 | -- | -- | -- | -- | -- | -- |
| *Gehyra oceanica* | -- | -- | -- | -- | -- | GQ257785 | -- | -- | JN394001 | -- | -- | -- |
| *Gehyra pamela* | -- | -- | -- | -- | -- | GQ257779 | -- | -- | -- | -- | -- | -- |
| *Gehyra pilbara* | -- | -- | -- | -- | -- | GQ257757 | -- | -- | JN394004 | -- | -- | -- |
| *Gehyra punctata* | -- | -- | -- | -- | -- | GQ257748 | -- | -- | -- | -- | -- | -- |
| *Gehyra purpurascens* | -- | -- | -- | -- | -- | GQ257800 | -- | -- | -- | -- | -- | -- |
| *Gehyra robusta* | -- | -- | -- | -- | -- | GQ257758 | -- | -- | JN394006 | -- | -- | -- |
| *Gehyra variegata* | AF090185 | -- | -- | AF090851 | -- | AY369026 | -- | -- | JN393994 | -- | -- | JQ945461 |
| *Gehyra xenopus* | -- | -- | -- | -- | -- | GQ257790 | -- | -- | JN394010 | -- | -- | -- |
| *Gekko athymus* | -- | -- | -- | -- | -- | JN019075 | -- | -- | JN019107 | -- | JN019139 | -- |
| *Gekko auriverrucosus* | -- | -- | -- | -- | EU417695 | JN019062 | -- | -- | JN019096 | -- | JN019127 | -- |
| *Gekko badenii* | -- | -- | -- | JQ945569 | -- | JN019065 | -- | -- | JN019099 | -- | JN019130 | JQ945462 |
| *Gekko chinensis* | -- | -- | -- | JQ945571 | EU417676 | JN019058 | -- | -- | JN019092 | -- | JN019123 | JQ945464 |
| *Gekko crombota* | -- | -- | -- | -- | -- | FJ487876 | -- | -- | -- | -- | -- | -- |
| *Gekko gecko* | HM370130 | HM370130 | EU402614 | EU366455 | NC_007627 | AY282753 | AY282753 | EU390898 | JN019087 | HQ876378 | AY662625 | EF534981 |
| *Gekko grossmanni* | -- | -- | -- | -- | -- | JN019064 | -- | -- | JN019098 | -- | JN019129 | -- |
| *Gekko hokouensis* | AF323511 | -- | -- | -- | EU417691 | JN019060 | -- | -- | JN019094 | -- | JN019125 | -- |
| *Gekko japonicus* | AF318271 | -- | -- | -- | EU417688 | JN019059 | -- | -- | JN019093 | -- | JN019124 | -- |
| *Gekko mindorensis* | -- | -- | -- | JQ945572 | -- | FJ487887 | -- | -- | JN019108 | -- | JN019140 | JQ945465 |
| *Gekko monarchus* | -- | -- | -- | JQ945573 | -- | FJ487870 | -- | -- | JN019110 | -- | JN019141 | JQ945466 |
| *Gekko petricolus* | -- | -- | -- | -- | -- | JN019067 | -- | -- | JN019101 | -- | JN019132 | -- |
| *Gekko porosus* | -- | -- | -- | -- | -- | FJ487881 | -- | -- | -- | -- | -- | -- |
| *Gekko romblon* | -- | -- | -- | -- | -- | JN710497 | -- | -- | JN710505 | -- | -- | -- |
| *Gekko smithii* | -- | -- | -- | -- | -- | FJ487868 | -- | -- | JN019091 | -- | JN019121 | -- |
| *Gekko swinhonis* | AF323518 | JQ906550 | -- | -- | EU417702 | JN019061 | -- | -- | JN019095 | -- | JN019126 | -- |
| *Gekko vittatus* | NC_008772 | NC_008772 | -- | JQ945575 | NC_008772 | AB178897 | NC_008772 | -- | JN019106 | -- | JN019137 | JQ945468 |
| *Geocalamus acutus* | AB162909 | FJ441724 | FJ441907 | FJ441784 | NC_006285 | FJ441967 | NC_006285 | JN568319 | -- | JN568486 | JN654860 | -- |
| *Geophis carinosus* | -- | -- | -- | GQ895815 | GQ895872 | -- | -- | -- | -- | -- | -- | -- |
| *Geophis godmani* | JQ598814 | JQ598877 | JQ599026 | -- | JQ598932 | -- | -- | -- | -- | -- | -- | -- |
| *Gerarda prevostiana* | AF499287 | EF395867 | -- | EF395939 | EF395916 | -- | -- | -- | -- | -- | -- | -- |
| *Gerrhonotus infernalis* | -- | -- | JQ845043 | -- | -- | AY742920 | -- | JQ844950 | -- | -- | -- | -- |
| *Gerrhonotus liocephalus* | AF056613 | -- | -- | -- | AF056598 | AY742924 | -- | -- | -- | -- | -- | -- |
| *Gerrhonotus parvus* | -- | -- | -- | -- | -- | AY742925 | -- | -- | -- | -- | -- | -- |
| *Gerrhosaurus flavigularis* | AJ416920 | AY167366 | -- | DQ100151 | DQ090883 | AY167400 | -- | EU636221 | -- | -- | -- | -- |
| *Gerrhosaurus major* | AJ416921 | AJ416922 | HM160588 | DQ100149 | DQ090881 | AY167396 | -- | EU636222 | -- | HM161062 | HM161157 | -- |
| *Gerrhosaurus multilineatus* | AY167351 | AY167368 | -- | -- | AY167385 | AY167402 | -- | -- | -- | -- | -- | -- |
| *Gerrhosaurus nigrolineatus* | HQ167134 | HQ167245 | EU108359 | EF632250 | EU116512 | AY167401 | -- | EU108025 | -- | -- | EF632205 | -- |
| *Gerrhosaurus skoogi* | -- | -- | -- | -- | AY167381 | AY167398 | -- | -- | -- | -- | -- | -- |
| *Gerrhosaurus typicus* | AF236039 | AY167365 | -- | -- | AY167382 | AY167399 | -- | -- | -- | -- | -- | -- |
| *Gerrhosaurus validus* | HQ167135 | HQ167246 | -- | DQ100150 | DQ090882 | AY167394 | -- | EU636223 | -- | -- | -- | -- |
| *Glaphyromorphus cracens* | DQ915285 | DQ915309 | -- | -- | -- | -- | DQ915333 | -- | -- | -- | -- | -- |
| *Glaphyromorphus darwiniensis* | DQ915286 | DQ915310 | -- | -- | -- | -- | DQ915334 | -- | -- | -- | -- | -- |
| *Glaphyromorphus douglasi* | DQ915287 | HM852502 | -- | -- | -- | -- | DQ915335 | -- | -- | -- | -- | -- |
| *Glaphyromorphus fuscicaudis* | DQ915289 | HM852504 | -- | -- | -- | -- | DQ915337 | -- | -- | -- | -- | -- |
| *Glaphyromorphus gracilipes* | AY169588 | AY169625 | -- | -- | -- | -- | AY169663 | -- | -- | -- | -- | -- |
| *Glaphyromorphus isolepis* | AY169587 | AY169624 | -- | -- | -- | -- | AY169662 | -- | -- | -- | -- | -- |
| *Glaphyromorphus mjobergi* | DQ915296 | DQ915320 | -- | -- | -- | -- | DQ915344 | -- | -- | -- | -- | -- |
| *Glaphyromorphus pardalis* | DQ915300 | DQ915324 | -- | -- | -- | -- | DQ915348 | -- | -- | -- | -- | -- |
| *Glaphyromorphus pumilus* | DQ915301 | DQ915325 | -- | -- | -- | -- | DQ915349 | -- | -- | -- | -- | -- |
| *Glaphyromorphus punctulatus* | DQ915302 | DQ915326 | -- | -- | -- | -- | DQ915350 | -- | -- | -- | -- | -- |
| *Gloydius blomhoffii* | -- | JF357952 | -- | -- | AY352752 | DQ839399 | AY352815 | -- | -- | -- | -- | -- |
| *Gloydius brevicaudus* | -- | -- | -- | JF827697 | JQ687497 | -- | JQ687485 | -- | -- | -- | -- | -- |
| *Gloydius halys* | AF057191 | AF057238 | -- | -- | AY223564 | AY662540 | -- | -- | -- | -- | AY662614 | -- |
| *Gloydius intermedius* | AF124153 | JN870194 | -- | JQ687513 | JN870201 | -- | JQ687475 | -- | -- | -- | -- | -- |
| *Gloydius saxatilis* | AF124154 | JN870195 | -- | JQ687521 | JN870202 | -- | JN870210 | -- | -- | -- | -- | -- |
| *Gloydius shedaoensis* | AF124156 | AF057241 | -- | AF435019 | AY223566 | -- | JQ687481 | -- | -- | -- | -- | -- |
| *Gloydius strauchi* | EF012814 | AF057239 | -- | JQ687509 | AY223563 | -- | EF012795 | -- | -- | -- | -- | -- |
| *Gloydius tsushimaensis* | JN870186 | JN870196 | -- | -- | JN870203 | -- | JN870211 | -- | -- | -- | -- | -- |
| *Gloydius ussuriensis* | AF124155 | AF057240 | -- | JQ687522 | AY223565 | -- | AY223622 | -- | -- | -- | -- | -- |
| *Gnypetoscincus queenslandiae* | AY308371 | AY169626 | -- | AY818794 | U21796 | AF373266 | AY169664 | -- | -- | -- | -- | -- |
| *Goggia lineata* | AY763261 | AY763274 | -- | AY172930 | -- | JX041353 | -- | -- | JQ945378 | -- | JQ945310 | JQ945469 |
| *Gomesophis brasiliensis* | GQ457796 | GQ457737 | -- | -- | -- | -- | -- | -- | -- | -- | -- | -- |
| *Gonatodes albogularis* | GU139932 | EF564024 | GU457866 | EF564078 | -- | JX041354 | -- | GU139877 | EF534839 | HQ876380 | EF534797 | EF564106 |
| *Gonatodes alexandermendesi* | GU139912 | -- | -- | GU139805 | -- | JX041355 | -- | GU139859 | -- | -- | -- | -- |
| *Gonatodes annularis* | GU139921 | EF564037 | -- | EF534919 | -- | JX041356 | -- | GU139867 | EF534835 | -- | EF534794 | EF564115 |
| *Gonatodes antillensis* | GU139933 | -- | -- | GU139847 | -- | -- | -- | GU139879 | -- | -- | -- | -- |
| *Gonatodes caudiscutatus* | -- | EF564011 | -- | EF534920 | -- | JX041357 | -- | -- | EF534836 | -- | EF534795 | EF534962 |
| *Gonatodes ceciliae* | GU139934 | EF564035 | -- | EF564088 | -- | JX041358 | -- | GU139881 | HQ426193 | -- | JF416914 | EF564114 |
| *Gonatodes concinnatus* | GU139937 | EF564013 | -- | EF564071 | -- | JX041359 | -- | GU139884 | HQ426194 | -- | HQ426282 | EF564097 |
| *Gonatodes daudini* | GU139938 | EF564034 | -- | EF534918 | -- | JX041360 | -- | GU139885 | EF534834 | -- | EF534793 | EF534960 |
| *Gonatodes eladioi* | -- | EF564025 | -- | EF564081 | -- | -- | -- | -- | HQ426195 | -- | HQ426283 | EF564107 |
| *Gonatodes falconensis* | GU139935 | -- | -- | GU139829 | -- | -- | -- | GU139882 | -- | -- | -- | -- |
| *Gonatodes hasemani* | GU139940 | EF564039 | -- | EF564091 | -- | -- | -- | GU139887 | EF534837 | -- | -- | EF564117 |
| *Gonatodes humeralis* | GU139941 | EU477069 | -- | EU435263 | DQ104133 | JX041361 | DQ104163 | GU139889 | EF534838 | -- | EF534796 | EF564118 |
| *Gonatodes infernalis* | GU139922 | -- | -- | GU139819 | -- | -- | -- | GU139871 | -- | -- | -- | -- |
| *Gonatodes ocellatus* | GU139945 | EF564014 | -- | EF564072 | -- | JX041362 | -- | GU139892 | HQ426196 | -- | HQ426284 | EF564098 |
| *Gonatodes petersi* | GU139947 | -- | -- | GU139841 | -- | -- | -- | GU139894 | -- | -- | -- | -- |
| *Gonatodes purpurogularis* | GU139936 | -- | -- | GU139830 | -- | -- | -- | GU139883 | -- | -- | -- | -- |
| *Gonatodes seigliei* | GU139943 | -- | -- | GU139837 | -- | -- | -- | GU139890 | -- | -- | -- | -- |
| *Gonatodes superciliaris* | GU139904 | -- | -- | -- | -- | -- | -- | -- | -- | -- | -- | -- |
| *Gonatodes taniae* | GU139944 | -- | -- | GU139838 | -- | -- | -- | GU139891 | -- | -- | -- | -- |
| *Gonatodes vittatus* | GU139946 | EF564033 | -- | EF564087 | -- | JX041363 | -- | GU139893 | -- | -- | HQ426285 | EF564113 |
| *Gongylomorphus bojerii* | FJ791003 | AY649162 | -- | AY818799 | FJ790976 | -- | -- | -- | -- | -- | -- | -- |
| *Gongylophis colubrinus* | AF544747 | AF544819 | FJ433976 | DQ465568 | U69811 | -- | -- | FJ434078 | -- | HQ876360 | EU402845 | FJ433906 |
| *Gongylophis conicus* | GQ225680 | AF512743 | AY988040 | DQ469787 | U69817 | -- | -- | -- | -- | -- | AY988074 | -- |
| *Gonionotophis brussauxi* | FJ404156 | AY611861 | -- | AY611952 | AY612043 | -- | FJ404358 | -- | -- | -- | -- | FJ404430 |
| *Goniurosaurus araneus* | AB308453 | AB308460 | -- | HQ426537 | -- | AB308468 | -- | -- | HQ426197 | -- | HQ426286 | HQ426455 |
| *Goniurosaurus catbaensis* | -- | EU499389 | -- | -- | -- | -- | -- | -- | -- | -- | -- | -- |
| *Goniurosaurus kuroiwae* | AB028749 | AB028766 | HQ876232 | -- | -- | AB308469 | -- | -- | -- | HQ876373 | HQ876449 | -- |
| *Goniurosaurus lichtenfelderi* | AB308455 | JF799756 | -- | -- | -- | AB308470 | -- | -- | -- | -- | -- | -- |
| *Goniurosaurus luii* | EF081253 | EU499391 | -- | HQ426538 | -- | AB308471 | -- | -- | HQ426198 | -- | HQ426287 | HQ426456 |
| *Gonocephalus chamaeleontinus* | AB070376 | AB070379 | -- | AF137526 | -- | -- | -- | -- | -- | -- | -- | -- |
| *Gonocephalus grandis* | AB031966 | AB031983 | -- | -- | AB263939 | AF128496 | -- | -- | -- | -- | -- | -- |
| *Gonocephalus kuhlii* | -- | -- | DQ340726 | DQ340681 | -- | -- | -- | -- | -- | -- | -- | -- |
| *Gonocephalus robinsonii* | AB070378 | AB070381 | -- | -- | -- | -- | -- | -- | -- | -- | -- | -- |
| *Gonyosoma jansenii* | AY122671 | -- | -- | DQ902100 | DQ902113 | DQ902220 | DQ902313 | -- | -- | -- | -- | -- |
| *Gonyosoma oxycephalum* | AY122678 | Z46490 | -- | AF471105 | AF471084 | DQ902241 | DQ902309 | -- | -- | -- | -- | -- |
| *Graciliscincus shonae* | -- | -- | -- | DQ675412 | -- | DQ675207 | -- | -- | -- | -- | DQ675334 | -- |
| *Grayia ornata* | AF158434 | AF158503 | FJ434002 | AF544684 | -- | -- | AF544663 | FJ434103 | -- | EF144086 | EF144091 | EF144113 |
| *Grayia smithii* | -- | -- | -- | DQ112080 | DQ112077 | -- | -- | -- | -- | -- | -- | -- |
| *Grayia tholloni* | -- | -- | -- | DQ486175 | DQ486351 | -- | DQ486326 | -- | -- | -- | -- | -- |
| *Gyalopion canum* | -- | -- | -- | GQ927315 | -- | -- | -- | -- | -- | -- | -- | -- |
| *Gymnophthalmus cryptus* | AF101362 | AF101362 | -- | -- | -- | -- | -- | -- | -- | -- | -- | -- |
| *Gymnophthalmus leucomystax* | AF101363 | AF420715 | -- | AF420824 | -- | -- | AF420906 | -- | -- | -- | -- | -- |
| *Gymnophthalmus pleei* | AF101364 | AF101364 | -- | -- | -- | -- | -- | -- | -- | -- | -- | -- |
| *Gymnophthalmus speciosus* | AF101368 | AF101368 | -- | -- | -- | -- | -- | -- | -- | -- | -- | -- |
| *Gymnophthalmus underwoodi* | AF101369 | AF101369 | -- | -- | -- | -- | -- | -- | -- | -- | -- | -- |
| *Gymnophthalmus vanzoi* | AF420687 | AF420743 | -- | AF420827 | -- | -- | AF420867 | -- | -- | -- | -- | -- |
| *Haemodracon riebeckii* | -- | -- | -- | EU293672 | -- | JX041367 | -- | -- | EU293695 | -- | EU293627 | EU293717 |
| *Hakaria simonyi* | AY649122 | AY308291 | -- | AY818807 | FJ972232 | -- | -- | -- | -- | -- | -- | -- |
| *Haplocercus ceylonensis* | KC347324 | KC347361 | -- | KC347401 | KC347478 | -- | KC347514 | -- | -- | -- | KC347438 | -- |
| *Hapsidophrys lineatus* | -- | AY611873 | -- | -- | -- | -- | -- | -- | -- | -- | -- | -- |
| *Hapsidophrys principis* | -- | FJ913482 | -- | -- | -- | -- | -- | -- | -- | -- | -- | -- |
| *Hapsidophrys smaragdina* | -- | AY611875 | FJ434003 | DQ112078 | DQ112075 | -- | -- | FJ434104 | -- | EF144087 | AY487381 | EF144114 |
| *Helicops angulatus* | GQ457797 | GQ457738 | JQ599027 | AF471160 | AF471037 | FJ416751 | -- | -- | -- | -- | -- | FJ416824 |
| *Helicops carinicaudus* | JQ598815 | -- | -- | JQ598984 | -- | -- | -- | -- | -- | -- | -- | -- |
| *Helicops gomesi* | GQ457798 | GQ457739 | -- | GQ457858 | -- | -- | -- | -- | -- | -- | -- | -- |
| *Helicops hagmanni* | JQ598816 | JQ598878 | -- | JQ598985 | -- | -- | -- | -- | -- | -- | -- | -- |
| *Helicops infrataeniatus* | GQ457799 | GQ457740 | -- | GQ457859 | JQ598933 | -- | U49310 | -- | -- | -- | -- | -- |
| *Heliobolus lugubris* | -- | DQ871142 | -- | EF632261 | -- | -- | -- | -- | -- | -- | EF632216 | -- |
| *Heliobolus spekii* | AF206608 | AF206608 | -- | EF632262 | AF206544 | -- | -- | -- | -- | -- | EF632217 | -- |
| *Hellenolacerta graeca* | AF440602 | GQ142100 | -- | EF632269 | GQ142128 | -- | -- | -- | -- | -- | EF632225 | -- |
| *Heloderma horridum* | AJ563605 | -- | GU457855 | JN090140 | -- | AF407539 | -- | GU456011 | -- | JN703066 | GU457978 | -- |
| *Heloderma suspectum* | NC_008776 | NC_008776 | FJ433955 | AY662566 | NC_008776 | AF085603 | NC_008776 | GU456012 | HQ426254 | DQ119617 | AY662606 | DQ119635 |
| *Hemachatus haemachatus* | U96797 | -- | -- | -- | AF217821 | -- | -- | -- | -- | -- | -- | -- |
| *Hemerophis socotrae* | AY039132 | AY188083 | -- | AY188003 | AY188042 | AY487016 | AY487055 | -- | -- | -- | -- | -- |
| *Hemiaspis damelii* | EU547122 | EU547171 | -- | -- | DQ233952 | -- | EU547025 | -- | -- | -- | EU546896 | -- |
| *Hemiaspis signata* | EU547123 | EU547172 | -- | -- | DQ233953 | -- | EU547026 | -- | -- | -- | EU546897 | -- |
| *Hemibungarus calligaster* | -- | -- | -- | EF137419 | EF137411 | -- | EF137403 | -- | -- | -- | -- | -- |
| *Hemidactylus aaronbaueri* | HM595676 | -- | -- | -- | HM595641 | -- | -- | -- | HM622367 | -- | -- | -- |
| *Hemidactylus agrius* | DQ120429 | -- | -- | -- | DQ120261 | -- | -- | -- | -- | -- | -- | -- |
| *Hemidactylus albofasciatus* | HM595678 | -- | -- | -- | HM595643 | -- | -- | -- | -- | -- | -- | -- |
| *Hemidactylus angulatus* | EF202130 | -- | -- | HQ426540 | DQ120237 | EU268367 | EU268430 | -- | EU268336 | -- | HM559686 | -- |
| *Hemidactylus bouvieri* | DQ120424 | -- | -- | AY863042 | DQ120249 | -- | -- | -- | -- | -- | -- | EF540746 |
| *Hemidactylus bowringii* | AF323515 | -- | -- | -- | EU268405 | EU268374 | EU268437 | -- | EU268343 | -- | -- | -- |
| *Hemidactylus brasilianus* | -- | -- | -- | HQ426523 | DQ120257 | EU268351 | EU268414 | -- | EU268320 | -- | -- | HQ426439 |
| *Hemidactylus brookii* | HM595685 | HM040938 | HM180358 | AY863049 | GQ375294 | EU268375 | EU268438 | -- | EU268344 | -- | GQ375314 | -- |
| *Hemidactylus citernii* | DQ120383 | -- | -- | -- | DQ120213 | -- | -- | -- | -- | -- | -- | -- |
| *Hemidactylus depressus* | -- | -- | -- | -- | HM559594 | HM559626 | -- | -- | HM559659 | -- | HM559692 | -- |
| *Hemidactylus dracaenacolus* | DQ120380 | -- | -- | -- | DQ120209 | -- | -- | -- | -- | -- | -- | -- |
| *Hemidactylus fasciatus* | DQ852724 | -- | HM180357 | JQ945577 | EU268403 | EU268371 | EU268434 | -- | EU268340 | -- | JQ945311 | JQ945470 |
| *Hemidactylus flaviviridis* | DQ120454 | HM040937 | -- | HQ426541 | EU268388 | EU268356 | EU268419 | -- | EU268325 | -- | HM559694 | HQ426458 |
| *Hemidactylus forbesii* | DQ120339 | -- | -- | -- | DQ120168 | -- | -- | -- | -- | -- | -- | -- |
| *Hemidactylus foudaii* | DQ120385 | -- | -- | -- | DQ120214 | -- | -- | -- | -- | -- | -- | -- |
| *Hemidactylus frenatus* | NC_012902 | HM012691 | EU108360 | EF534940 | EU116513 | EU268359 | NC_012902 | EU108026 | EU268328 | -- | EF534814 | EF534982 |
| *Hemidactylus garnotii* | DQ120459 | -- | -- | -- | EU268395 | EU268364 | EU268427 | -- | EU268333 | -- | HM559697 | -- |
| *Hemidactylus giganteus* | HM595692 | -- | -- | -- | HM595658 | -- | -- | -- | HM559665 | -- | -- | -- |
| *Hemidactylus gracilis* | HM595696 | -- | -- | -- | HM595660 | EU268379 | -- | -- | HM622374 | -- | -- | -- |
| *Hemidactylus granti* | DQ120381 | -- | -- | -- | DQ120210 | -- | -- | -- | -- | -- | -- | -- |
| *Hemidactylus greefii* | DQ120413 | -- | -- | AY863044 | EU268401 | EU268369 | EU268432 | -- | EU268338 | -- | -- | HQ426459 |
| *Hemidactylus haitianus* | DQ120387 | -- | -- | HQ426543 | EU730676 | EU268372 | EU268435 | -- | EU268341 | -- | HM559700 | -- |
| *Hemidactylus homoeolepis* | DQ120340 | -- | -- | -- | DQ120171 | -- | -- | -- | -- | -- | -- | -- |
| *Hemidactylus karenorum* | DQ120464 | -- | -- | -- | EU268394 | EU268362 | EU268425 | -- | EU268331 | -- | -- | -- |
| *Hemidactylus lemurinus* | HQ675860 | -- | -- | -- | HQ675988 | -- | -- | -- | -- | -- | -- | -- |
| *Hemidactylus leschenaultii* | HM595697 | -- | -- | -- | HM595662 | -- | -- | -- | HM559669 | -- | -- | -- |
| *Hemidactylus longicephalus* | DQ120416 | -- | -- | HQ426544 | DQ120246 | HM559637 | -- | -- | HM559670 | -- | HM559703 | HQ426460 |
| *Hemidactylus mabouia* | HM180323 | JF329706 | HM180375 | HQ426545 | EU268393 | EU268361 | EU268424 | -- | EU268330 | -- | HM559704 | HQ426462 |
| *Hemidactylus macropholis* | DQ120343 | -- | -- | HQ426547 | DQ120208 | JX041369 | -- | -- | HQ426203 | -- | HQ426292 | HQ426463 |
| *Hemidactylus maculatus* | HM595699 | -- | -- | -- | HM595664 | -- | -- | -- | HM559674 | -- | -- | -- |
| *Hemidactylus mercatorius* | -- | JN172104 | JQ073123 | AY863046 | -- | -- | -- | -- | -- | -- | JQ073244 | -- |
| *Hemidactylus mindiae* | -- | -- | -- | -- | HQ833748 | -- | -- | -- | -- | -- | -- | -- |
| *Hemidactylus modestus* | DQ120386 | -- | -- | -- | DQ120215 | -- | -- | -- | -- | -- | -- | -- |
| *Hemidactylus oxyrhinus* | DQ120344 | -- | -- | -- | DQ120173 | -- | -- | -- | -- | -- | -- | -- |
| *Hemidactylus palaichthus* | DQ120434 | -- | -- | HQ426548 | EU268400 | EU268368 | EU268431 | -- | EU268337 | -- | -- | HQ426464 |
| *Hemidactylus persicus* | HM595701 | -- | -- | -- | EU268409 | EU268377 | EU268440 | -- | EU268346 | -- | -- | -- |
| *Hemidactylus platycephalus* | DQ120437 | HM192641 | JQ073125 | AY863045 | DQ120270 | -- | -- | -- | -- | -- | JQ073246 | -- |
| *Hemidactylus platyurus* | -- | -- | -- | HQ426530 | EU268384 | EU268352 | EU268415 | -- | EU268321 | -- | HM559685 | HQ426446 |
| *Hemidactylus prashadi* | HM595702 | -- | -- | -- | HM595668 | -- | -- | -- | HM559676 | -- | -- | -- |
| *Hemidactylus pumilio* | DQ120382 | -- | -- | -- | DQ120211 | -- | -- | -- | -- | -- | -- | -- |
| *Hemidactylus reticulatus* | HM595705 | -- | -- | -- | EU268410 | -- | -- | -- | EU268347 | -- | -- | -- |
| *Hemidactylus robustus* | DQ120345 | -- | -- | HQ426549 | EU268408 | EU268376 | EU268439 | -- | EU268345 | -- | EU054271 | HQ426465 |
| *Hemidactylus sataraensis* | HM595708 | -- | -- | -- | HM595672 | -- | -- | -- | -- | -- | -- | -- |
| *Hemidactylus triedrus* | HM595709 | -- | -- | HQ426550 | HM595675 | -- | -- | -- | HM559683 | -- | -- | HQ426466 |
| *Hemidactylus turcicus* | HQ675926 | -- | -- | AF363540 | AF364319 | EU268360 | EU268423 | -- | EU268329 | -- | HQ426293 | HQ676115 |
| *Hemidactylus yerburyi* | -- | -- | -- | -- | DQ120207 | -- | -- | -- | -- | -- | -- | -- |
| *Hemiergis decresiensis* | HM852459 | HM852488 | -- | -- | -- | -- | -- | -- | -- | -- | -- | -- |
| *Hemiergis initialis* | HM852469 | HM852500 | -- | -- | -- | -- | DQ915338 | -- | -- | -- | -- | -- |
| *Hemiergis millewae* | HM852462 | HM852489 | -- | -- | -- | -- | -- | -- | -- | -- | -- | -- |
| *Hemiergis peronii* | HM852467 | HM852495 | HQ655171 | HQ655206 | -- | -- | AY169665 | -- | -- | -- | -- | -- |
| *Hemiergis quadrilineatum* | HM852463 | HM852490 | -- | -- | -- | -- | -- | -- | -- | -- | -- | -- |
| *Hemiphyllodactylus aurantiacus* | -- | -- | -- | -- | FJ971012 | JN393933 | -- | -- | JN394011 | -- | JN393977 | -- |
| *Hemiphyllodactylus typus* | -- | -- | -- | -- | FJ971011 | GQ257744 | -- | -- | -- | -- | -- | -- |
| *Hemiphyllodactylus yunnanensis* | -- | -- | -- | JQ945579 | FJ971005 | JN393949 | -- | -- | JN394013 | -- | JN393979 | JQ945472 |
| *Hemirhagerrhis hildebrandtii* | FJ404134 | FJ404214 | -- | FJ387214 | FJ404311 | -- | FJ404337 | -- | -- | -- | -- | FJ404409 |
| *Hemirhagerrhis kelleri* | -- | -- | -- | DQ486159 | DQ486335 | -- | DQ486311 | -- | -- | -- | -- | -- |
| *Hemirhagerrhis viperina* | -- | -- | -- | -- | DQ486453 | -- | DQ486289 | -- | -- | -- | -- | -- |
| *Hemitheconyx caudicinctus* | AB028755 | AY140998 | -- | HQ426552 | AY141007 | AB308472 | -- | -- | HQ426205 | -- | HQ426294 | HQ426468 |
| *Hemitheconyx taylori* | AB308456 | AB308457 | -- | HQ426553 | -- | AB308473 | -- | -- | HQ426206 | -- | HQ426295 | HQ426469 |
| *Hemorrhois algirus* | AY039149 | -- | -- | AY486935 | AY486911 | AY486998 | AY487037 | -- | -- | -- | -- | -- |
| *Hemorrhois hippocrepis* | DQ451992 | -- | -- | AY486940 | DQ451987 | AY487006 | AY487045 | -- | -- | -- | -- | -- |
| *Hemorrhois nummifer* | AY039163 | AY376771 | -- | AY376800 | AY039201 | AY487010 | AY487049 | -- | -- | -- | -- | -- |
| *Hemorrhois ravergieri* | AY039131 | -- | -- | AY486944 | AY486920 | AY487011 | AY487050 | -- | -- | -- | -- | -- |
| *Heterodactylus imbricatus* | AF420661 | AF420725 | -- | AF420835 | -- | -- | AF420885 | -- | -- | -- | -- | -- |
| *Heterodon nasicus* | AY577018 | AY577027 | -- | GQ457861 | -- | -- | -- | -- | -- | -- | -- | -- |
| *Heterodon platirhinos* | AY577019 | AY577028 | EU402641 | JQ598986 | GU112412 | FJ416750 | AF402659 | GU353271 | -- | JN703080 | -- | FJ416823 |
| *Heterodon simus* | AY577020 | AY577029 | -- | AF471142 | AF217840 | DQ902242 | DQ902310 | -- | -- | -- | -- | -- |
| *Heteroliodon occipitalis* | FJ404158 | FJ404211 | -- | FJ387211 | FJ404308 | -- | -- | -- | -- | -- | -- | FJ404432 |
| *Heteronotia binoei* | NC_010292 | NC_010292 | -- | JQ945580 | NC_010292 | DQ287674 | NC_010292 | -- | EU054270 | -- | EU054285 | JQ945473 |
| *Heteronotia planiceps* | -- | -- | -- | JQ945581 | -- | EU054300 | -- | -- | EU054268 | -- | EU054284 | JQ945474 |
| *Heteronotia spelea* | -- | -- | -- | -- | -- | HQ840102 | -- | -- | -- | -- | -- | -- |
| *Hierophis gemonensis* | AY039145 | AY376770 | -- | AY376799 | AY039183 | AY487005 | AY487044 | -- | -- | -- | -- | -- |
| *Hierophis spinalis* | AY541508 | AY376773 | -- | AY486948 | AY486924 | AY487017 | AY487056 | -- | -- | -- | -- | -- |
| *Hierophis viridiflavus* | AY541505 | AY376774 | -- | AY486949 | AY486925 | AY487018 | AY487057 | -- | -- | -- | -- | -- |
| *Himalayophis tibetanus* | AY352776 | AY352715 | -- | -- | AY352749 | -- | AY352810 | -- | -- | -- | -- | -- |
| *Holaspis guentheri* | -- | HQ605784 | -- | HQ605880 | HQ605838 | -- | -- | -- | -- | -- | HQ616546 | -- |
| *Holaspis laevis* | -- | HQ605782 | -- | EF632263 | HQ605836 | -- | -- | -- | -- | -- | EF632218 | -- |
| *Holbrookia lacerata* | AF194267 | -- | -- | -- | AF194237 | EU543795 | -- | -- | -- | -- | -- | -- |
| *Holbrookia maculata* | U39560 | L41445 | DQ385341 | -- | AY141100 | AY328439 | DQ114053 | -- | -- | -- | DQ385416 | -- |
| *Holbrookia propinqua* | AF194276 | -- | -- | -- | EU543768 | AY297491 | -- | -- | -- | -- | -- | -- |
| *Holodactylus africanus* | AB028754 | AB028768 | -- | AY172933 | -- | AB308474 | -- | -- | HQ426207 | -- | HQ426296 | HQ426470 |
| *Homalopsis buccata* | EF395892 | EF395868 | EU402642 | EF395940 | EF395917 | -- | -- | EU390922 | -- | EF144070 | -- | EF144097 |
| *Homonota andicola* | FJ985061 | -- | -- | -- | FJ985041 | -- | -- | -- | -- | -- | -- | -- |
| *Homonota borellii* | FJ985062 | -- | -- | -- | FJ985042 | -- | -- | -- | -- | -- | -- | -- |
| *Homonota darwinii* | FJ985053 | -- | -- | EU293673 | FJ985038 | JX041373 | -- | -- | EU293696 | -- | EU293628 | EU293718 |
| *Homonota fasciata* | DQ852725 | -- | -- | EU293674 | FJ985032 | JX041374 | -- | -- | EU293697 | -- | EU293629 | EU293719 |
| *Homonota gaudichaudii* | FJ985065 | -- | -- | HQ426536 | FJ985045 | JX041351 | -- | -- | HQ426192 | -- | HQ426281 | HQ426454 |
| *Homonota underwoodi* | FJ985063 | -- | -- | -- | FJ985043 | -- | -- | -- | -- | -- | -- | -- |
| *Homopholis fasciata* | DQ852723 | -- | -- | DQ852735 | -- | EU054250 | EU054178 | -- | EU054202 | -- | EU054226 | JQ945475 |
| *Homopholis mulleri* | -- | -- | -- | -- | -- | EU054241 | EU054169 | -- | EU054193 | -- | EU054217 | -- |
| *Homopholis walbergii* | -- | -- | JQ073116 | -- | -- | EU054248 | EU054175 | -- | EU054200 | -- | EU054224 | -- |
| *Homoroselaps lacteus* | FJ404135 | AY611843 | JQ599029 | AY058931 | AF217833 | -- | FJ404339 | -- | -- | -- | -- | FJ404411 |
| *Hoplocephalus bitorquatus* | EU547128 | EU547177 | -- | EU546941 | -- | -- | EU547031 | -- | -- | -- | EU546902 | -- |
| *Hoplocercus spinosus* | -- | -- | EU586769 | -- | -- | U82683 | -- | -- | -- | -- | AY662592 | -- |
| *Hoplodactylus chrysosireticus* | -- | GU460040 | -- | -- | -- | GU459842 | -- | -- | GU459641 | -- | GU459438 | -- |
| *Hoplodactylus cryptozoicus* | -- | GU460007 | -- | -- | -- | GU459809 | -- | -- | GU459608 | -- | GU459406 | -- |
| *Hoplodactylus duvaucelii* | -- | GU460043 | -- | -- | -- | GU459845 | -- | -- | GU459644 | -- | GU459442 | -- |
| *Hoplodactylus granulatus* | -- | GU460016 | -- | -- | -- | GU459818 | -- | -- | GU459617 | -- | GU459415 | -- |
| *Hoplodactylus kahutarae* | -- | GU460018 | -- | -- | -- | GU459820 | -- | -- | GU459618 | -- | GU459417 | -- |
| *Hoplodactylus maculatus* | -- | HM542437 | -- | HQ343308 | -- | GU459861 | -- | -- | GU459660 | -- | GU459458 | HM542428 |
| *Hoplodactylus nebulosus* | -- | GU460019 | -- | -- | -- | GU459821 | -- | -- | GU459620 | -- | GU459418 | -- |
| *Hoplodactylus pacificus* | -- | GU459993 | -- | -- | -- | GU459795 | -- | -- | GU459594 | -- | GU459392 | -- |
| *Hoplodactylus rakiurae* | -- | GU459984 | -- | -- | -- | GU459786 | -- | -- | GU459585 | -- | GU459383 | -- |
| *Hoplodactylus stephensi* | -- | GU459982 | -- | -- | -- | -- | -- | -- | GU459583 | -- | GU459381 | -- |
| *Hormonotus modestus* | FJ404159 | FJ404195 | -- | FJ404261 | FJ404296 | -- | FJ404360 | -- | -- | -- | -- | FJ404433 |
| *Hydrelaps darwiniensis* | EU547133 | EU547182 | -- | -- | DQ233948 | -- | EU547035 | -- | -- | -- | EU546907 | -- |
| *Hydrodynastes bicinctus* | GQ457802 | GQ457742 | JQ599030 | GQ457862 | JQ598935 | -- | -- | -- | -- | -- | -- | -- |
| *Hydrodynastes gigas* | GQ457803 | GQ457743 | -- | GQ895816 | GQ895873 | -- | -- | -- | -- | -- | -- | -- |
| *Hydromorphus concolor* | -- | -- | -- | GQ895817 | GQ895874 | -- | -- | -- | -- | -- | -- | -- |
| *Hydrophis atriceps* | -- | JQ217152 | -- | -- | JQ217206 | -- | JQ217216 | -- | -- | -- | -- | -- |
| *Hydrophis brooki* | -- | DQ234028 | -- | -- | DQ233943 | -- | -- | -- | -- | -- | -- | -- |
| *Hydrophis cyanocinctus* | -- | DQ234032 | -- | FJ587189 | DQ233946 | -- | FJ593215 | -- | -- | -- | FJ587112 | -- |
| *Hydrophis czeblukovi* | -- | DQ234019 | -- | -- | DQ233944 | -- | -- | -- | -- | -- | -- | -- |
| *Hydrophis elegans* | -- | DQ234022 | -- | FJ587191 | DQ233951 | -- | FJ593217 | -- | -- | -- | FJ587114 | -- |
| *Hydrophis lapemoides* | -- | DQ234034 | -- | -- | DQ233955 | -- | FJ593219 | -- | -- | -- | -- | -- |
| *Hydrophis macdowelli* | -- | -- | -- | FJ587193 | DQ233957 | -- | FJ593221 | -- | -- | -- | FJ587116 | -- |
| *Hydrophis melanocephalus* | D31609 | -- | -- | -- | D31620 | -- | -- | -- | -- | -- | -- | -- |
| *Hydrophis ornatus* | -- | DQ234027 | -- | FJ587195 | DQ233962 | -- | FJ593223 | -- | -- | -- | FJ587118 | -- |
| *Hydrophis pacificus* | -- | DQ234037 | -- | FJ587198 | DQ233965 | -- | FJ593226 | -- | -- | -- | FJ587121 | -- |
| *Hydrophis parviceps* | -- | -- | -- | -- | JQ920439 | -- | -- | -- | -- | -- | -- | -- |
| *Hydrophis semperi* | U96798 | -- | -- | -- | AF217822 | -- | -- | -- | -- | -- | -- | -- |
| *Hydrophis spiralis* | -- | DQ234038 | -- | -- | DQ233966 | -- | -- | -- | -- | -- | -- | -- |
| *Hydrops triangularis* | GQ457804 | GQ457744 | JQ599032 | AF471158 | AF471039 | -- | -- | -- | -- | -- | -- | -- |
| *Hydrosaurus amboinensis* | NC_014178 | NC_014178 | -- | -- | NC_014178 | NC_014178 | NC_014178 | -- | -- | -- | -- | -- |
| *Hypnale hypnale* | AF057189 | AY352717 | -- | -- | AY352750 | -- | AY352812 | -- | -- | -- | -- | -- |
| *Hypnale nepa* | KC347325 | KC347362 | -- | KC347402 | KC347479 | -- | KC347515 | -- | -- | -- | KC347439 | -- |
| *Hypnale zara* | KC347326 | KC347363 | -- | KC347403 | KC347480 | -- | KC347516 | -- | -- | -- | KC347440 | -- |
| *Hypsiglena affinis* | -- | -- | -- | -- | GU353241 | -- | EU363055 | GU353278 | -- | -- | -- | -- |
| *Hypsiglena chlorophaea* | NC_013977 | EU728593 | -- | -- | EU728593 | EU728593 | EU728593 | FJ455197 | -- | -- | -- | -- |
| *Hypsiglena jani* | EU728592 | EU728592 | -- | -- | EU728592 | EU728592 | EU728592 | FJ455193 | -- | -- | -- | -- |
| *Hypsiglena ochrorhyncha* | EU728589 | EU728589 | -- | -- | EU728589 | EU728589 | EU728589 | FJ455201 | -- | -- | -- | -- |
| *Hypsiglena slevini* | EU728584 | EU728584 | -- | -- | EF078497 | EU728584 | EU728584 | FJ455191 | -- | -- | -- | -- |
| *Hypsiglena torquata* | NC_013992 | EU728591 | -- | AF471159 | AF471038 | EU728591 | U49309 | FJ455192 | -- | -- | -- | -- |
| *Hypsilurus boydii* | -- | -- | DQ340727 | DQ340682 | -- | AY133013 | -- | JF804550 | -- | JF804592 | JF806196 | -- |
| *Hypsilurus bruijnii* | -- | -- | DQ340728 | AF137522 | -- | AY133014 | -- | -- | -- | -- | -- | -- |
| *Hypsilurus dilophus* | -- | -- | DQ340729 | DQ340683 | -- | AF128466 | -- | -- | -- | -- | -- | -- |
| *Hypsilurus modestus* | -- | -- | DQ340730 | DQ340684 | -- | AF128464 | -- | -- | -- | -- | -- | -- |
| *Hypsilurus nigrigularis* | -- | -- | -- | -- | -- | AY133016 | -- | -- | -- | -- | HQ662413 | -- |
| *Hypsilurus papuensis* | -- | -- | -- | -- | -- | AY133017 | -- | -- | -- | -- | -- | -- |
| *Hypsilurus spinipes* | -- | -- | DQ340731 | DQ340685 | -- | AY133018 | -- | -- | -- | -- | -- | -- |
| *Hypsirhynchus ferox* | AF158447 | AF158515 | -- | GQ895818 | GQ895875 | FJ416780 | FJ416816 | -- | -- | -- | -- | FJ416853 |
| *Ialtris dorsalis* | AF158456 | AF158525 | -- | -- | FJ416735 | FJ416773 | FJ416809 | -- | -- | -- | -- | FJ416846 |
| *Iberolacerta aranica* | AY151955 | AF440612 | -- | AY152029 | AY267239 | -- | -- | -- | -- | -- | -- | -- |
| *Iberolacerta aurelioi* | -- | AF440610 | -- | AY152025 | AY267238 | -- | -- | -- | -- | -- | -- | -- |
| *Iberolacerta bonnali* | AY151970 | AF080292 | -- | AY152035 | AY267240 | -- | -- | -- | -- | -- | -- | -- |
| *Iberolacerta cyreni* | AY151928 | AF378940 | -- | AY152009 | AY267232 | -- | -- | -- | -- | -- | HQ616539 | -- |
| *Iberolacerta galani* | DQ497135 | -- | -- | DQ497120 | DQ497074 | -- | -- | -- | -- | -- | -- | -- |
| *Iberolacerta horvathi* | AJ238186 | AF080289 | -- | AY256658 | GQ142125 | -- | -- | -- | -- | -- | EF632219 | -- |
| *Iberolacerta monticola* | AY151940 | AF440604 | -- | DQ097146 | AF206530 | -- | -- | -- | -- | -- | EF632220 | -- |
| *Ichnotropis capensis* | -- | DQ871149 | -- | -- | -- | DQ871091 | -- | -- | -- | -- | DQ871207 | -- |
| *Ichnotropis squamulosa* | AF080365 | AF080367 | -- | EF632266 | AF080366 | -- | -- | -- | -- | -- | EF632221 | -- |
| *Iguana delicatissima* | -- | -- | -- | HM352538 | -- | -- | U66231 | HM352528 | -- | -- | -- | -- |
| *Iguana iguana* | AJ278511 | NC_002793 | -- | AF148708 | AF020251 | NC_002793 | U66230 | HM352529 | -- | -- | -- | -- |
| *Imantodes cenchoa* | EU728586 | EU728586 | EU402643 | GQ457865 | EF078505 | EU728586 | EU728586 | GQ334668 | -- | JN703067 | EU402847 | -- |
| *Imantodes gemmistratus* | -- | -- | -- | -- | EF078510 | -- | GQ334588 | -- | -- | -- | -- | -- |
| *Imantodes inornatus* | -- | -- | -- | -- | EF078511 | -- | GQ334590 | GU353279 | -- | -- | -- | -- |
| *Imantodes lentiferus* | AF158463 | AF158532 | -- | -- | EF078514 | -- | EF078562 | -- | -- | -- | -- | -- |
| *Iphisa elegans* | AF101370 | AF420714 | -- | AF420843 | -- | -- | AF420889 | -- | -- | -- | -- | -- |
| *Iranolacerta brandtii* | AF080319 | GQ142111 | -- | GQ142152 | GQ142140 | -- | -- | -- | -- | -- | GQ142162 | -- |
| *Iranolacerta zagrosica* | -- | -- | -- | -- | EF422421 | -- | -- | -- | -- | -- | -- | -- |
| *Isopachys anguinoides* | AB028803 | AB028815 | -- | -- | -- | -- | -- | -- | -- | -- | -- | -- |
| *Ithycyphus miniatus* | -- | AY188058 | -- | AY187980 | AY188019 | -- | -- | -- | -- | -- | -- | -- |
| *Ithycyphus oursi* | FJ404160 | FJ404212 | -- | FJ387212 | FJ404309 | -- | FJ404361 | -- | -- | -- | -- | FJ404434 |
| *Janetaescincus braueri* | AY818766 | AY649164 | HM160589 | AY818802 | FJ972229 | -- | -- | -- | -- | HM161063 | HM161158 | -- |
| *Janetaescincus veseyfitzgeraldi* | AY818767 | -- | -- | AY818803 | FJ972230 | -- | -- | -- | -- | -- | -- | -- |
| *Japalura flaviceps* | -- | -- | -- | -- | -- | AF128500 | -- | -- | -- | -- | -- | -- |
| *Japalura polygonata* | AB031968 | AB031985 | -- | -- | -- | -- | -- | -- | -- | -- | -- | -- |
| *Japalura splendida* | -- | -- | AF497713 | -- | -- | AF288230 | -- | -- | -- | -- | -- | -- |
| *Japalura tricarinata* | -- | -- | -- | -- | -- | AF128478 | -- | -- | -- | -- | AY662585 | -- |
| *Japalura variegata* | -- | -- | -- | -- | -- | AF128479 | -- | -- | -- | -- | -- | -- |
| *Kanakysaurus viviparus* | -- | -- | -- | DQ675413 | -- | DQ675209 | -- | -- | -- | -- | DQ675289 | -- |
| *Kentropyx altamazonica* | AY046456 | AY046498 | -- | -- | -- | -- | -- | -- | -- | -- | -- | -- |
| *Kentropyx calcarata* | AY046458 | AF420760 | -- | AF420864 | -- | -- | AF420913 | -- | -- | -- | -- | -- |
| *Kentropyx paulensis* | EU345187 | EU345181 | -- | -- | -- | -- | -- | -- | -- | -- | -- | -- |
| *Kentropyx pelviceps* | AY046459 | AY046501 | -- | -- | -- | -- | -- | -- | -- | -- | -- | -- |
| *Kentropyx striata* | AY046460 | AY046502 | -- | -- | -- | -- | -- | -- | -- | -- | -- | -- |
| *Kentropyx vanzoi* | EU345188 | EU345178 | -- | -- | -- | -- | -- | -- | -- | -- | -- | -- |
| *Kentropyx viridistriga* | EU345186 | EU345183 | -- | -- | AF151193 | -- | AF151207 | -- | -- | -- | -- | -- |
| *Kinyongia adolfifriderici* | -- | DQ923820 | -- | FJ984257 | -- | AF448727 | FJ981755 | -- | -- | -- | DQ996659 | -- |
| *Kinyongia boehmei* | -- | GQ221948 | -- | -- | -- | GQ221969 | -- | -- | HE602016 | -- | GQ221953 | -- |
| *Kinyongia carpenteri* | -- | DQ923822 | -- | -- | -- | EF014305 | -- | -- | -- | -- | DQ996660 | -- |
| *Kinyongia excubitor* | -- | DQ923823 | -- | -- | -- | EF014307 | -- | -- | -- | -- | DQ996661 | -- |
| *Kinyongia fischeri* | NC_012465 | DQ923828 | -- | -- | U69759 | EF014312 | EF222188 | -- | -- | -- | DQ996665 | -- |
| *Kinyongia matschiei* | -- | GQ221949 | -- | -- | -- | GQ221967 | -- | -- | -- | -- | GQ221956 | -- |
| *Kinyongia multituberculata* | -- | GQ221947 | -- | -- | -- | GQ221968 | -- | -- | -- | -- | GQ221957 | -- |
| *Kinyongia oxyrhina* | AM422437 | DQ923832 | -- | -- | -- | EF014316 | -- | -- | -- | -- | DQ996670 | -- |
| *Kinyongia tavetana* | AM422431 | DQ923833 | -- | -- | -- | AF448730 | -- | -- | -- | -- | DQ996672 | -- |
| *Kinyongia tenue* | -- | -- | -- | -- | -- | EF014319 | -- | -- | -- | -- | DQ996673 | -- |
| *Kinyongia uthmoelleri* | DQ397246 | DQ923837 | -- | -- | -- | EF014321 | -- | -- | -- | -- | DQ996675 | -- |
| *Kinyongia vosseleri* | -- | GQ221945 | -- | -- | -- | GQ221966 | -- | -- | -- | -- | GQ221955 | -- |
| *Kinyongia xenorhina* | DQ397250 | DQ923840 | -- | -- | -- | EF014324 | -- | -- | -- | -- | DQ996678 | -- |
| *Lacerta agilis* | DQ097096 | DQ658846 | -- | EU365405 | GQ142118 | -- | -- | -- | -- | -- | EF632222 | -- |
| *Lacerta bilineata* | EF422437 | AY714979 | -- | AF211204 | AY714981 | -- | -- | -- | -- | -- | -- | -- |
| *Lacerta media* | AF206590 | AF206590 | -- | DQ097144 | U88603 | -- | -- | -- | -- | -- | -- | -- |
| *Lacerta pamphylica* | AF149954 | AF149970 | -- | DQ097143 | DQ097089 | -- | -- | -- | -- | -- | -- | -- |
| *Lacerta schreiberi* | AF206591 | DQ097097 | -- | EU365406 | AF372119 | -- | -- | -- | -- | -- | -- | -- |
| *Lacerta strigata* | DQ097094 | DQ097099 | -- | DQ097137 | DQ097091 | -- | -- | -- | -- | -- | -- | -- |
| *Lacerta trilineata* | AF149950 | AF149935 | -- | DQ097140 | AF233427 | -- | -- | -- | -- | -- | -- | -- |
| *Lacerta viridis* | NC_008328 | AF086952 | EU108361 | DQ097131 | EU116514 | NC_008328 | NC_008328 | GU456031 | -- | JF804593 | EU108535 | -- |
| *Lacertaspis chriswildi* | AY308375 | AY308226 | -- | -- | -- | -- | -- | -- | -- | -- | -- | -- |
| *Lacertaspis gemmiventris* | AY308380 | AY308233 | -- | -- | -- | -- | -- | -- | -- | -- | -- | -- |
| *Lacertaspis lepesmei* | AY308384 | AY308234 | -- | -- | -- | -- | -- | -- | -- | -- | -- | -- |
| *Lacertaspis reichenowi* | AY308385 | AY308235 | -- | -- | -- | -- | -- | -- | -- | -- | -- | -- |
| *Lacertaspis rohdei* | AY308386 | AY308238 | -- | -- | -- | -- | -- | -- | -- | -- | -- | -- |
| *Lacertoides pardalis* | -- | -- | -- | DQ675351 | -- | DQ675211 | -- | -- | -- | -- | DQ675291 | -- |
| *Lachesis muta* | AF057221 | AF057268 | -- | -- | U96021 | -- | U41885 | EU390924 | -- | JN703071 | -- | -- |
| *Lachesis stenophrys* | AF057220 | AF057267 | EU402644 | -- | U96016 | -- | U96026 | -- | -- | -- | -- | -- |
| *Laemanctus longipes* | -- | -- | -- | -- | -- | AF528716 | -- | -- | -- | -- | -- | -- |
| *Lamprolepis smaragdina* | AB028774 | AY169605 | HQ655172 | AY217854 | AY217803 | -- | AY169643 | -- | -- | HQ907626 | -- | -- |
| *Lampropeltis alterna* | -- | -- | -- | FJ627799 | AF337130 | FJ627846 | AY497307 | -- | -- | -- | -- | -- |
| *Lampropeltis californiae* | -- | -- | -- | -- | FJ997662 | -- | -- | -- | -- | -- | EU402848 | -- |
| *Lampropeltis calligaster* | -- | -- | -- | DQ902091 | DQ902129 | DQ902243 | AY739644 | -- | -- | -- | -- | -- |
| *Lampropeltis elapsoides* | -- | -- | -- | FJ627795 | AF337095 | -- | -- | -- | -- | -- | -- | -- |
| *Lampropeltis extenuata* | -- | -- | -- | DQ902093 | DQ902131 | DQ902245 | -- | -- | -- | -- | -- | -- |
| *Lampropeltis getula* | AY122822 | -- | EU402645 | -- | DQ360325 | -- | DQ360458 | EU390925 | -- | HQ876362 | EU402848 | -- |
| *Lampropeltis holbrooki* | -- | -- | -- | -- | FJ997653 | -- | -- | -- | -- | -- | -- | -- |
| *Lampropeltis mexicana* | FJ623962 | -- | -- | FJ627800 | AF337146 | FJ627836 | AY497310 | -- | -- | -- | -- | -- |
| *Lampropeltis nigra* | -- | -- | -- | FJ627796 | DQ360350 | -- | DQ360483 | -- | -- | -- | -- | -- |
| *Lampropeltis pyromelana* | FJ623961 | -- | -- | FJ627794 | AF337151 | FJ627845 | -- | -- | -- | -- | -- | -- |
| *Lampropeltis ruthveni* | AY122820 | -- | -- | FJ627803 | AY122736 | FJ627847 | AY497311 | -- | -- | -- | -- | -- |
| *Lampropeltis splendida* | -- | -- | -- | -- | FJ997776 | -- | -- | -- | -- | -- | -- | -- |
| *Lampropeltis triangulum* | FJ623963 | -- | -- | FJ627798 | AF337161 | FJ627837 | AY497312 | -- | -- | -- | -- | -- |
| *Lampropeltis webbi* | -- | -- | -- | -- | -- | -- | AY497308 | -- | -- | -- | -- | -- |
| *Lampropeltis zonata* | -- | -- | -- | FJ627802 | AF337150 | -- | -- | -- | -- | -- | -- | -- |
| *Lamprophis aurora* | -- | -- | -- | HQ207101 | HQ207143 | -- | HQ207185 | -- | -- | -- | -- | -- |
| *Lamprophis fiskii* | FJ404163 | FJ404202 | -- | DQ486178 | DQ486354 | -- | FJ404363 | -- | -- | -- | -- | FJ404437 |
| *Lamprophis fuliginosus* | AY122681 | JF357950 | FJ433993 | DQ486163 | AF471060 | -- | AF544664 | EU390926 | -- | EF144077 | AY487378 | FJ404438 |
| *Lamprophis fuscus* | -- | -- | -- | -- | HQ207127 | -- | HQ207169 | -- | -- | -- | -- | -- |
| *Lamprophis guttatus* | FJ404166 | AY611890 | -- | DQ486179 | DQ486355 | -- | FJ404366 | -- | -- | -- | -- | FJ404439 |
| *Lamprophis inornatus* | FJ404167 | AY611891 | -- | AY611982 | AY235727 | -- | FJ404367 | -- | -- | -- | -- | FJ404440 |
| *Lamprophis lineatus* | FJ404168 | FJ404205 | -- | FJ387205 | FJ404303 | -- | -- | -- | -- | -- | -- | FJ404441 |
| *Lamprophis olivaceus* | FJ404170 | AY611862 | -- | AY611953 | AY612044 | -- | -- | -- | -- | -- | -- | FJ404443 |
| *Lamprophis swazicus* | -- | -- | -- | DQ486180 | DQ486356 | -- | DQ486331 | -- | -- | -- | -- | -- |
| *Lamprophis virgatus* | FJ404171 | AY611825 | -- | AY611917 | AY612008 | -- | FJ404369 | -- | -- | -- | -- | FJ404444 |
| *Lampropholis coggeri* | AY818754 | FJ379527 | -- | AY818786 | -- | -- | FJ379463 | -- | -- | HM030089 | -- | -- |
| *Lampropholis delicata* | AY818753 | -- | -- | AY818785 | AY818823 | -- | -- | -- | -- | -- | -- | -- |
| *Lampropholis guichenoti* | EU568020 | AY626712 | -- | DQ675352 | EU567836 | EF567304 | GQ403645 | -- | -- | -- | EU568111 | -- |
| *Lampropholis robertsi* | -- | -- | -- | HM030191 | -- | -- | HM030318 | -- | -- | HM030393 | -- | -- |
| *Langaha madagascariensis* | FJ404172 | AY188059 | -- | AY187981 | AY188020 | -- | FJ404370 | -- | -- | -- | -- | FJ404445 |
| *Lankascincus fallax* | AY308390 | AY308240 | -- | -- | -- | -- | -- | -- | -- | -- | -- | -- |
| *Lanthanotus borneensis* | AF004475 | -- | GU457859 | AY662564 | -- | AY662537 | -- | GU456015 | -- | -- | AY662609 | -- |
| *Lapemis curtus* | EU547134 | EU547183 | -- | -- | DQ233973 | -- | EU547036 | -- | -- | -- | EU366437 | -- |
| *Larutia seribuatensis* | -- | -- | HQ907229 | -- | -- | HQ907429 | -- | -- | -- | HQ907636 | -- | -- |
| *Latastia longicaudata* | AF206609 | AF080358 | -- | EF632272 | AF206545 | -- | -- | -- | -- | -- | EF632229 | -- |
| *Laticauda colubrina* | EU547089 | EU547138 | EU402647 | AY058932 | AF217834 | -- | EU546998 | EU390927 | -- | EF144074 | EU366433 | EF144101 |
| *Laticauda guineai* | -- | -- | -- | -- | -- | -- | FJ606516 | -- | -- | -- | -- | -- |
| *Laticauda laticaudata* | D31608 | FJ587204 | -- | -- | -- | -- | FJ606526 | -- | -- | -- | -- | -- |
| *Laticauda saintgironsi* | -- | -- | -- | -- | -- | -- | FJ606507 | -- | -- | -- | -- | -- |
| *Laudakia caucasia* | AY053643 | AY053765 | DQ340732 | DQ340686 | -- | AF172705 | AY053998 | -- | -- | -- | -- | -- |
| *Laudakia erythrogastra* | -- | -- | -- | -- | -- | AF028680 | -- | -- | -- | -- | -- | -- |
| *Laudakia himalayana* | -- | -- | -- | -- | -- | AF028676 | -- | -- | -- | -- | -- | -- |
| *Laudakia lehmanni* | -- | -- | -- | -- | -- | GQ242233 | -- | -- | -- | -- | GQ242235 | -- |
| *Laudakia microlepis* | -- | -- | -- | -- | -- | AF028678 | -- | -- | -- | -- | -- | -- |
| *Laudakia nupta* | -- | HQ901101 | -- | -- | -- | AF128513 | -- | -- | -- | -- | -- | -- |
| *Laudakia sacra* | -- | -- | -- | -- | -- | AF128515 | -- | -- | -- | -- | -- | -- |
| *Laudakia stellio* | GU952115 | GU128464 | -- | -- | AB266886 | AF128516 | -- | -- | -- | -- | -- | -- |
| *Laudakia stoliczkana* | AF236821 | -- | -- | -- | -- | AF128519 | -- | -- | -- | -- | -- | -- |
| *Laudakia tuberculata* | -- | -- | -- | -- | -- | AF128514 | -- | -- | -- | -- | -- | -- |
| *Leiocephalus barahonensis* | U39564 | -- | HQ876223 | DQ119594 | -- | EF591774 | -- | JF804551 | -- | DQ119622 | DQ119624 | DQ119640 |
| *Leiocephalus carinatus* | -- | -- | AY987970 | -- | -- | AF049864 | -- | -- | -- | -- | AY662598 | -- |
| *Leiocephalus personatus* | AB266739 | NC_012834 | -- | -- | NC_012834 | NC_012834 | NC_012834 | -- | -- | -- | -- | -- |
| *Leiocephalus psammodromus* | -- | -- | -- | -- | -- | JF812253 | -- | -- | -- | -- | -- | -- |
| *Leiocephalus raviceps* | -- | -- | -- | -- | -- | GQ502767 | -- | -- | -- | -- | FJ356744 | -- |
| *Leiocephalus schreibersii* | -- | Z48656 | -- | -- | -- | AF528753 | -- | -- | -- | -- | -- | -- |
| *Leioheterodon geayi* | -- | AY188060 | -- | AY187982 | AY188021 | -- | -- | -- | -- | -- | -- | -- |
| *Leioheterodon madagascariensis* | AF544768 | AY188061 | FJ433992 | AY187983 | AY188022 | -- | U49318 | FJ434093 | -- | EF144076 | AY487377 | EF144103 |
| *Leioheterodon modestus* | -- | JF357951 | -- | AY058933 | AY058967 | -- | AY058978 | -- | -- | -- | -- | -- |
| *Leiolepis belliana* | AB031969 | AB031986 | AY987965 | FJ984253 | -- | U82689 | -- | JF804552 | -- | HQ876324 | AY662587 | -- |
| *Leiolepis guentherpetersi* | -- | -- | DQ340733 | AF137529 | -- | AF128461 | -- | -- | -- | -- | -- | -- |
| *Leiolepis guttata* | NC_014179 | NC_014179 | -- | -- | NC_014179 | NC_014179 | NC_014179 | -- | -- | -- | -- | -- |
| *Leiolepis reevesii* | -- | EU428189 | -- | -- | -- | -- | -- | -- | -- | -- | -- | -- |
| *Leiolopisma mauritiana* | -- | -- | -- | -- | AY818817 | -- | -- | -- | -- | -- | -- | -- |
| *Leiolopisma telfairii* | AY308391 | AY308241 | -- | AY818780 | AY818815 | DQ675259 | -- | -- | -- | -- | -- | -- |
| *Leiopython albertisii* | EF545026 | EF545053 | -- | -- | U69835 | -- | -- | -- | -- | -- | -- | -- |
| *Leiosaurus bellii* | -- | -- | -- | -- | -- | AF528730 | -- | -- | -- | -- | -- | -- |
| *Leiosaurus catamarcensis* | AF338341 | -- | JF806022 | -- | -- | AF528731 | -- | JF804553 | -- | JF804594 | JF806207 | -- |
| *Leiosaurus paronae* | AF338328 | -- | -- | -- | -- | -- | -- | -- | -- | -- | -- | -- |
| *Lepidoblepharis festae* | -- | EF564007 | -- | EF564068 | -- | -- | -- | -- | HQ426208 | -- | HQ426297 | EF564094 |
| *Lepidoblepharis xanthostigma* | GU139948 | EF564010 | -- | EU435262 | -- | JX041376 | -- | GU139895 | EF534831 | -- | EU435217 | EF564095 |
| *Lepidodactylus lugubris* | DQ852704 | -- | -- | EF534938 | -- | JN393944 | -- | -- | EF534853 | -- | EF534812 | EF534980 |
| *Lepidodactylus moestus* | -- | -- | -- | -- | -- | JN019079 | -- | -- | JN019111 | -- | JN019143 | -- |
| *Lepidodactylus novaeguineae* | -- | -- | -- | JQ945583 | -- | JX041378 | -- | -- | JQ945380 | -- | JQ945312 | JQ945476 |
| *Lepidodactylus orientalis* | -- | -- | -- | -- | -- | JN019080 | -- | -- | JN019112 | -- | JN019144 | -- |
| *Lepidophyma cuicateca* | -- | -- | EU108370 | EU116691 | EU116522 | -- | -- | EU108036 | -- | -- | EU108543 | -- |
| *Lepidophyma dontomasi* | GQ850626 | -- | EU108371 | EU116694 | EU116525 | -- | AY584467 | EU108038 | -- | -- | EU108546 | -- |
| *Lepidophyma flavimaculatum* | NC_008775 | DQ249036 | EU108374 | EU116715 | EU116546 | NC_008775 | AB162908 | EU108060 | -- | HQ876350 | EU108567 | -- |
| *Lepidophyma gaigeae* | GQ850643 | -- | EU108395 | EU116722 | EU116553 | -- | -- | EU108066 | -- | -- | EU108573 | -- |
| *Lepidophyma lineri* | -- | -- | EU108402 | EU116723 | EU116554 | -- | -- | EU108068 | -- | -- | EU108575 | -- |
| *Lepidophyma lipetzi* | GQ850644 | -- | EU108403 | EU116724 | EU116555 | -- | -- | EU108069 | -- | -- | EU108576 | -- |
| *Lepidophyma lowei* | GQ850645 | -- | EU108404 | EU116726 | EU116557 | -- | -- | EU108070 | -- | -- | EU108578 | -- |
| *Lepidophyma mayae* | GQ850648 | -- | EU108406 | EU116730 | EU116561 | -- | AY584468 | EU108074 | -- | -- | EU108581 | -- |
| *Lepidophyma micropholis* | GQ850649 | -- | EU108410 | EU116735 | EU116567 | -- | -- | EU108077 | -- | -- | EU108587 | -- |
| *Lepidophyma occulor* | GQ850652 | -- | EU108416 | EU116737 | EU116568 | -- | -- | EU108082 | -- | -- | EU108589 | -- |
| *Lepidophyma pajapanensis* | GQ850653 | -- | EU108417 | EU116738 | EU116569 | -- | -- | EU108083 | -- | -- | EU108590 | -- |
| *Lepidophyma radula* | GQ850654 | -- | EU108418 | EU116739 | EU116570 | -- | -- | EU108084 | -- | -- | EU108591 | -- |
| *Lepidophyma reticulatum* | GQ850656 | -- | EU108419 | EU116742 | EU116574 | -- | -- | EU108088 | -- | -- | EU108595 | -- |
| *Lepidophyma smithii* | GQ850659 | -- | EU108423 | EU116754 | EU116585 | -- | AY584469 | EU108089 | -- | -- | EU108604 | -- |
| *Lepidophyma sylvaticum* | GQ850660 | AY217994 | EU108434 | EU116768 | EU116595 | -- | AY584470 | EU108112 | -- | -- | EU108616 | -- |
| *Lepidophyma tuxtlae* | GQ850665 | -- | EU108448 | EU116774 | EU116604 | -- | AY584471 | EU108115 | -- | -- | EU108624 | -- |
| *Lepidothyris fernandi* | AY649124 | AY169606 | -- | -- | -- | -- | AY169644 | -- | -- | -- | -- | -- |
| *Leposoma annectans* | JN588621 | -- | -- | JN588701 | JN588675 | -- | JN588735 | -- | -- | -- | -- | -- |
| *Leposoma baturitensis* | JN588650 | -- | -- | JN588714 | JN588688 | -- | JN588744 | -- | -- | -- | -- | -- |
| *Leposoma guianense* | JN588625 | -- | -- | JN588711 | JN588683 | -- | JN588741 | -- | -- | -- | -- | -- |
| *Leposoma nanodactylus* | JN588653 | -- | -- | JN588717 | JN588691 | -- | JN588747 | -- | -- | -- | -- | -- |
| *Leposoma osvaldoi* | JN588629 | -- | -- | AF420854 | JN588687 | -- | AF420897 | -- | -- | -- | -- | -- |
| *Leposoma parietale* | JN588654 | -- | -- | JN588716 | JN588690 | AY662543 | JN588746 | -- | -- | -- | AY662621 | -- |
| *Leposoma percarinatum* | AF420700 | AF420735 | -- | -- | JN588673 | -- | AF420898 | -- | -- | -- | -- | -- |
| *Leposoma puk* | JN588651 | -- | -- | JN588718 | JN588692 | -- | JN588748 | -- | -- | -- | -- | -- |
| *Leposoma scincoides* | AY218004 | AY217954 | -- | AY217851 | AY217800 | -- | JN588727 | -- | -- | -- | -- | -- |
| *Leposoma southi* | JN588652 | -- | -- | JN588715 | JN588689 | -- | JN588745 | -- | -- | -- | -- | -- |
| *Leposternon infraorbitale* | -- | FJ441723 | FJ441906 | FJ441783 | -- | FJ441966 | -- | -- | -- | -- | FJ441843 | -- |
| *Leposternon microcephalum* | -- | FJ441718 | FJ441897 | FJ441778 | -- | FJ441961 | -- | -- | -- | -- | FJ441838 | -- |
| *Leposternon polystegum* | -- | FJ441721 | FJ441903 | FJ441781 | -- | FJ441964 | -- | -- | -- | -- | FJ441841 | -- |
| *Leptodeira annulata* | GQ457806 | GQ457746 | FJ433998 | AF544690 | EF078515 | FJ416749 | FJ416787 | FJ434099 | -- | EF144081 | AY487375 | EF144108 |
| *Leptodeira bakeri* | -- | -- | -- | -- | GQ334517 | -- | GQ334620 | GQ334673 | -- | -- | -- | -- |
| *Leptodeira frenata* | -- | -- | -- | -- | EF078531 | -- | FJ810244 | FJ810242 | -- | -- | -- | -- |
| *Leptodeira maculata* | -- | -- | -- | -- | GQ334524 | -- | GQ334627 | GQ334674 | -- | -- | -- | -- |
| *Leptodeira nigrofasciata* | -- | -- | -- | -- | GQ334526 | -- | GQ334630 | FJ810241 | -- | -- | -- | -- |
| *Leptodeira punctata* | -- | -- | -- | -- | EF078529 | -- | EF078578 | GQ334682 | -- | -- | -- | -- |
| *Leptodeira rubricata* | -- | -- | -- | -- | GQ334527 | -- | GQ334631 | -- | -- | -- | -- | -- |
| *Leptodeira septentrionalis* | GU018148 | EU728590 | -- | -- | EF078522 | EU728590 | EU728590 | FJ455188 | -- | -- | -- | -- |
| *Leptodeira splendida* | -- | -- | -- | -- | EF078521 | -- | GQ334657 | GQ334680 | -- | -- | -- | -- |
| *Leptophis ahaetulla* | HM565762 | -- | -- | GQ927316 | GQ927321 | -- | -- | -- | -- | -- | -- | -- |
| *Leptosiaphos amieti* | AY308392 | AY308242 | -- | -- | -- | -- | -- | -- | -- | -- | -- | -- |
| *Leptosiaphos graueri* | AY308394 | AY308246 | -- | -- | -- | -- | -- | -- | -- | -- | -- | -- |
| *Leptosiaphos hackarsi* | AY308397 | AY308247 | -- | -- | -- | -- | -- | -- | -- | -- | -- | -- |
| *Leptosiaphos kilimensis* | AY308398 | AY308249 | -- | -- | -- | -- | -- | -- | -- | -- | -- | -- |
| *Leptosiaphos vigintiserierum* | AY308409 | AY308259 | -- | EU164504 | -- | -- | -- | -- | -- | -- | -- | -- |
| *Leptotyphlops adleri* | GQ469246 | GQ469246 | GQ469172 | GQ469058 | GQ469155 | -- | -- | GQ469013 | -- | -- | GQ469035 | -- |
| *Leptotyphlops albifrons* | GQ469224 | GQ469224 | GQ469180 | GQ469065 | GQ469096 | -- | -- | GQ469020 | -- | -- | GQ469043 | -- |
| *Leptotyphlops algeriensis* | GQ469243 | GQ469243 | GQ469173 | -- | GQ469151 | -- | -- | GQ469014 | -- | -- | GQ469036 | -- |
| *Leptotyphlops asbolepis* | GQ469210 | GQ469210 | GQ469174 | GQ469059 | GQ469088 | -- | -- | GQ469015 | -- | -- | GQ469037 | -- |
| *Leptotyphlops bicolor* | GQ469234 | GQ469234 | GQ469175 | GQ469060 | GQ469153 | -- | -- | GQ469016 | -- | -- | GQ469038 | -- |
| *Leptotyphlops blanfordi* | GQ469241 | GQ469241 | -- | GQ469061 | GQ469104 | -- | -- | -- | -- | -- | GQ469039 | -- |
| *Leptotyphlops boueti* | GQ469248 | GQ469248 | GQ469177 | GQ469062 | GQ469157 | -- | -- | GQ469017 | -- | -- | GQ469040 | -- |
| *Leptotyphlops breuili* | GQ469207 | GQ469207 | -- | -- | GQ469111 | -- | -- | -- | -- | -- | -- | -- |
| *Leptotyphlops carlae* | GQ469204 | GQ469204 | -- | -- | GQ469107 | -- | -- | -- | -- | -- | -- | -- |
| *Leptotyphlops columbi* | GQ469215 | GQ469215 | FJ433957 | AF544718 | AF544671 | -- | -- | FJ434063 | -- | FJ433911 | AY487383 | -- |
| *Leptotyphlops conjunctus* | GQ469261 | GQ469281 | GQ469179 | GQ469069 | GQ469167 | -- | -- | GQ469023 | -- | -- | GQ469042 | -- |
| *Leptotyphlops distanti* | GQ469271 | GQ469271 | GQ469181 | GQ469066 | GQ469162 | -- | -- | GQ469021 | -- | -- | GQ469044 | -- |
| *Leptotyphlops dulcis* | GQ469229 | GQ469229 | GQ469182 | GQ469067 | GQ469105 | -- | -- | GQ469022 | -- | -- | GQ469045 | -- |
| *Leptotyphlops goudotii* | GQ469222 | GQ469222 | -- | -- | GQ469117 | -- | -- | -- | -- | -- | -- | -- |
| *Leptotyphlops humilis* | GQ469228 | GQ469228 | EU402648 | AY099979 | AY099991 | AB079597 | NC_005961 | EU390928 | -- | HQ876364 | EU402851 | -- |
| *Leptotyphlops leptipilepta* | GQ469201 | GQ469201 | GQ469185 | GQ469070 | GQ469085 | -- | -- | GQ469024 | -- | -- | GQ469047 | -- |
| *Leptotyphlops longicaudus* | GQ469244 | GQ469244 | GQ469186 | GQ469071 | GQ469131 | -- | -- | GQ469025 | -- | -- | GQ469048 | -- |
| *Leptotyphlops macrolepis* | GQ469227 | GQ469227 | -- | -- | GQ469114 | -- | -- | -- | -- | -- | -- | -- |
| *Leptotyphlops macrorhynchus* | GQ469245 | GQ469245 | GQ469187 | GQ469072 | GQ469115 | -- | -- | GQ469026 | -- | -- | GQ469049 | -- |
| *Leptotyphlops nigricans* | GQ469239 | GQ469239 | -- | -- | GQ469134 | -- | -- | -- | -- | -- | -- | -- |
| *Leptotyphlops nigroterminus* | GQ469259 | GQ469259 | GQ469188 | GQ469073 | GQ469142 | -- | -- | GQ469027 | -- | -- | GQ469050 | -- |
| *Leptotyphlops occidentalis* | GQ469252 | GQ469252 | GQ469189 | GQ469074 | GQ469133 | -- | -- | GQ469028 | -- | -- | GQ469051 | -- |
| *Leptotyphlops pyrites* | GQ469194 | GQ469194 | GQ469170 | GQ469056 | GQ469079 | -- | -- | GQ469011 | -- | -- | GQ469033 | -- |
| *Leptotyphlops rouxestevae* | GQ469247 | GQ469247 | GQ469190 | GQ469075 | GQ469156 | -- | -- | GQ469029 | -- | -- | GQ469052 | -- |
| *Leptotyphlops scutifrons* | GQ469268 | GQ469270 | -- | -- | GQ469169 | -- | -- | -- | -- | -- | -- | -- |
| *Leptotyphlops septemstriatus* | GQ469232 | GQ469232 | GQ469191 | GQ469076 | GQ469116 | -- | -- | GQ469030 | -- | -- | GQ469053 | -- |
| *Leptotyphlops sylvicolus* | GQ469282 | GQ469284 | GQ469192 | GQ469077 | GQ469168 | -- | -- | GQ469031 | -- | -- | GQ469054 | -- |
| *Lerista aericeps* | EF672754 | EF672825 | -- | -- | -- | -- | EF672966 | -- | -- | -- | -- | -- |
| *Lerista allochira* | EF672755 | EF672826 | -- | -- | -- | -- | EF672967 | -- | -- | -- | -- | -- |
| *Lerista ameles* | EF672756 | EF672827 | -- | -- | -- | -- | EF672968 | -- | -- | -- | -- | -- |
| *Lerista apoda* | EF672757 | EF672828 | -- | -- | -- | -- | EF672969 | -- | -- | -- | -- | -- |
| *Lerista arenicola* | EF672758 | EF672829 | -- | -- | -- | -- | EF672970 | -- | -- | -- | -- | -- |
| *Lerista axillaris* | EF672759 | EF672830 | -- | -- | -- | -- | EF672971 | -- | -- | -- | -- | -- |
| *Lerista baynesi* | EF672760 | EF672831 | -- | -- | -- | -- | EF672972 | -- | -- | -- | -- | -- |
| *Lerista bipes* | EF672761 | AY169628 | -- | -- | -- | -- | AY169666 | -- | -- | -- | -- | -- |
| *Lerista borealis* | EF672762 | EF672833 | -- | -- | -- | -- | EF672974 | -- | -- | -- | -- | -- |
| *Lerista bougainvillii* | AY308411 | AY169629 | -- | -- | AF020033 | -- | AY169667 | -- | -- | -- | -- | -- |
| *Lerista carpentariae* | EF672763 | EF672834 | -- | -- | -- | -- | EF672975 | -- | -- | -- | -- | -- |
| *Lerista chordae* | EF672809 | EF672880 | -- | -- | -- | -- | EF673021 | -- | -- | -- | -- | -- |
| *Lerista christinae* | EF672764 | EF672835 | -- | -- | -- | -- | EF672976 | -- | -- | -- | -- | -- |
| *Lerista cinerea* | EF672765 | EF672836 | -- | -- | -- | -- | EF672977 | -- | -- | -- | -- | -- |
| *Lerista connivens* | EF672766 | EF672837 | -- | -- | -- | -- | EF672978 | -- | -- | -- | -- | -- |
| *Lerista desertorum* | EF672767 | EF672838 | -- | -- | -- | -- | EF672979 | -- | -- | -- | -- | -- |
| *Lerista distinguenda* | EF672768 | EF672839 | -- | -- | -- | -- | EF672980 | -- | -- | -- | -- | -- |
| *Lerista dorsalis* | EF672769 | EF672840 | -- | -- | AF020035 | -- | EF672981 | -- | -- | -- | -- | -- |
| *Lerista edwardsae* | EF672770 | EF672841 | -- | -- | -- | -- | EF672982 | -- | -- | -- | -- | -- |
| *Lerista elegans* | EF672771 | EF672842 | -- | -- | -- | -- | EF672983 | -- | -- | -- | -- | -- |
| *Lerista elongata* | EF672772 | EF672843 | -- | -- | -- | -- | EF672984 | -- | -- | -- | -- | -- |
| *Lerista emmotti* | EF672773 | EF672844 | -- | -- | -- | -- | EF672985 | -- | -- | -- | -- | -- |
| *Lerista eupoda* | EF672774 | EF672845 | -- | -- | -- | -- | EF672986 | -- | -- | -- | -- | -- |
| *Lerista flammicauda* | EF672775 | EF672846 | -- | -- | -- | -- | EF672987 | -- | -- | -- | -- | -- |
| *Lerista fragilis* | EF672776 | EF672847 | -- | -- | -- | -- | EF672988 | -- | -- | -- | -- | -- |
| *Lerista frosti* | EF672777 | EF672848 | -- | -- | -- | -- | EF672989 | -- | -- | -- | -- | -- |
| *Lerista gascoynensis* | EF672778 | EF672849 | -- | -- | -- | -- | EF672990 | -- | -- | -- | -- | -- |
| *Lerista gerrardii* | EF672779 | EF672850 | -- | -- | -- | -- | EF672991 | -- | -- | -- | -- | -- |
| *Lerista greeri* | EF672780 | EF672851 | -- | -- | -- | -- | EF672992 | -- | -- | -- | -- | -- |
| *Lerista griffini* | EF672781 | EF672852 | -- | -- | -- | -- | EF672993 | -- | -- | -- | -- | -- |
| *Lerista haroldi* | EF672782 | EF672853 | -- | -- | -- | -- | EF672994 | -- | -- | -- | -- | -- |
| *Lerista humphriesi* | EF672783 | EF672854 | -- | -- | -- | -- | EF672995 | -- | -- | -- | -- | -- |
| *Lerista ingrami* | EF672784 | EF672855 | -- | -- | -- | -- | EF672996 | -- | -- | -- | -- | -- |
| *Lerista ips* | EF672785 | EF672856 | -- | -- | -- | -- | EF672997 | -- | -- | -- | -- | -- |
| *Lerista kalumburu* | EF672786 | EF672857 | -- | -- | -- | -- | EF672998 | -- | -- | -- | -- | -- |
| *Lerista karlschmidti* | EF672787 | EF672858 | -- | -- | -- | -- | EF672999 | -- | -- | -- | -- | -- |
| *Lerista kendricki* | EF672788 | EF672859 | -- | -- | -- | -- | EF673000 | -- | -- | -- | -- | -- |
| *Lerista kennedyensis* | EF672789 | EF672860 | -- | -- | -- | -- | EF673001 | -- | -- | -- | -- | -- |
| *Lerista labialis* | EF672790 | EF672861 | -- | -- | -- | -- | EF673002 | -- | -- | -- | -- | -- |
| *Lerista lineata* | EF672791 | EF672862 | -- | -- | -- | -- | EF673003 | -- | -- | -- | -- | -- |
| *Lerista lineopunctulata* | EF672792 | EF672863 | -- | -- | -- | -- | EF673004 | -- | -- | -- | -- | -- |
| *Lerista macropisthopus* | EF672793 | EF672864 | -- | -- | -- | -- | EF673005 | -- | -- | -- | -- | -- |
| *Lerista microtis* | EF672794 | EF672865 | -- | -- | AF020036 | -- | EF673006 | -- | -- | -- | -- | -- |
| *Lerista muelleri* | EF672795 | EF672866 | -- | -- | -- | -- | EF673007 | -- | -- | -- | -- | -- |
| *Lerista neander* | EF672796 | EF672867 | HQ655173 | HQ655208 | -- | -- | EF673008 | -- | -- | -- | -- | -- |
| *Lerista nichollsi* | EF672797 | EF672868 | -- | -- | -- | -- | EF673009 | -- | -- | -- | -- | -- |
| *Lerista onsloviana* | EF672798 | EF672869 | -- | -- | -- | -- | EF673010 | -- | -- | -- | -- | -- |
| *Lerista orientalis* | EF672799 | EF672870 | -- | -- | -- | -- | EF673011 | -- | -- | -- | -- | -- |
| *Lerista petersoni* | EF672800 | EF672871 | -- | -- | -- | -- | EF673012 | -- | -- | -- | -- | -- |
| *Lerista picturata* | EF672801 | EF672872 | -- | -- | -- | -- | EF673013 | -- | -- | -- | -- | -- |
| *Lerista planiventralis* | EF672802 | EF672873 | -- | -- | -- | -- | EF673014 | -- | -- | -- | -- | -- |
| *Lerista praepedita* | EF672803 | EF672874 | -- | -- | -- | -- | EF673015 | -- | -- | -- | -- | -- |
| *Lerista punctatovittata* | EF672804 | EF672875 | -- | -- | -- | -- | EF673016 | -- | -- | -- | -- | -- |
| *Lerista puncticauda* | EF672805 | EF672876 | -- | -- | -- | -- | EF673017 | -- | -- | -- | -- | -- |
| *Lerista robusta* | EF672806 | EF672877 | -- | -- | -- | -- | EF673018 | -- | -- | -- | -- | -- |
| *Lerista simillima* | EF672807 | EF672878 | -- | -- | -- | -- | EF673019 | -- | -- | -- | -- | -- |
| *Lerista speciosa* | EF672808 | EF672879 | -- | -- | -- | -- | EF673020 | -- | -- | -- | -- | -- |
| *Lerista stictopleura* | EF672810 | EF672881 | -- | -- | -- | -- | EF673022 | -- | -- | -- | -- | -- |
| *Lerista stylis* | EF672811 | EF672882 | -- | -- | -- | -- | EF673023 | -- | -- | -- | -- | -- |
| *Lerista taeniata* | EF672812 | EF672883 | -- | -- | -- | -- | EF673024 | -- | -- | -- | -- | -- |
| *Lerista terdigitata* | EF672813 | EF672884 | -- | -- | -- | -- | EF673025 | -- | -- | -- | -- | -- |
| *Lerista tridactyla* | EF672814 | EF672885 | -- | -- | -- | -- | EF673026 | -- | -- | -- | -- | -- |
| *Lerista uniduo* | EF672815 | EF672886 | -- | -- | -- | -- | EF673027 | -- | -- | -- | -- | -- |
| *Lerista varia* | EF672816 | EF672887 | -- | -- | -- | -- | EF673028 | -- | -- | -- | -- | -- |
| *Lerista vermicularis* | EF672817 | EF672888 | -- | -- | -- | -- | EF673029 | -- | -- | -- | -- | -- |
| *Lerista viduata* | EF672818 | EF672889 | -- | -- | -- | -- | EF673030 | -- | -- | -- | -- | -- |
| *Lerista walkeri* | EF672819 | EF672890 | -- | -- | -- | -- | EF673031 | -- | -- | -- | -- | -- |
| *Lerista wilkinsi* | EF672820 | EF672891 | -- | -- | -- | -- | EF673032 | -- | -- | -- | -- | -- |
| *Lerista xanthura* | EF672821 | EF672892 | -- | -- | -- | -- | EF673033 | -- | -- | -- | -- | -- |
| *Lerista yuna* | EF672822 | EF672893 | -- | -- | -- | -- | EF673034 | -- | -- | -- | -- | -- |
| *Lerista zietzi* | EF672823 | EF672894 | -- | -- | -- | -- | EF673035 | -- | -- | -- | -- | -- |
| *Lerista zonulata* | EF672824 | EF672895 | -- | -- | -- | -- | EF673036 | -- | -- | -- | -- | -- |
| *Letheobia obtusa* | -- | -- | -- | -- | -- | -- | -- | GU902625 | -- | -- | GU902700 | -- |
| *Lialis burtonis* | AF090183 | GU460140 | GU457868 | EF534906 | -- | AY134599 | -- | GU456024 | GU459742 | HQ876382 | EF534782 | EF534948 |
| *Lialis jicari* | -- | AY134528 | -- | AY134564 | -- | AY369025 | -- | -- | -- | -- | AY662628 | -- |
| *Liasis fuscus* | EF545025 | EF545052 | -- | -- | EF545100 | -- | -- | -- | -- | -- | -- | -- |
| *Liasis mackloti* | EF545024 | EF545051 | FJ433970 | AF544726 | U69840 | -- | -- | FJ434075 | -- | -- | AY487397 | -- |
| *Liasis olivaceus* | EF545023 | EF545050 | -- | -- | U69842 | -- | -- | -- | -- | -- | -- | -- |
| *Lichanura trivirgata* | GQ200595 | GQ200595 | FJ433979 | AY099974 | AY099988 | -- | -- | FJ434080 | -- | HQ876361 | EU402852 | -- |
| *Liolaemus abaucan* | DQ237526 | -- | -- | JN683080 | DQ237647 | AF099263 | DQ237764 | -- | -- | -- | -- | -- |
| *Liolaemus albiceps* | DQ237557 | -- | -- | JN683084 | DQ237677 | AF099267 | DQ237795 | -- | -- | -- | -- | -- |
| *Liolaemus andinus* | -- | -- | -- | -- | -- | AF305789 | -- | -- | -- | -- | -- | -- |
| *Liolaemus archeforus* | JF272798 | -- | -- | -- | JF272765 | AY661901 | -- | -- | -- | -- | -- | -- |
| *Liolaemus atacamensis* | -- | -- | -- | -- | -- | AF305787 | -- | -- | -- | -- | -- | -- |
| *Liolaemus audituvelatus* | -- | -- | -- | -- | -- | AF305792 | -- | -- | -- | -- | -- | -- |
| *Liolaemus austromendocinus* | AY367843 | -- | -- | AY367899 | AY173839 | AF099239 | AY367872 | -- | -- | -- | -- | -- |
| *Liolaemus azarai* | JN614950 | -- | -- | -- | JN614931 | -- | -- | -- | -- | -- | -- | -- |
| *Liolaemus baguali* | JF272799 | -- | -- | -- | JF272766 | AY661905 | -- | -- | -- | -- | -- | -- |
| *Liolaemus bellii* | AY662069 | -- | HQ876220 | -- | AY173828 | AF099223 | AY173757 | JF804554 | -- | HQ876331 | HQ876436 | -- |
| *Liolaemus bibronii* | DQ237494 | -- | -- | -- | DQ989788 | AF099221 | AY173721 | -- | -- | -- | -- | -- |
| *Liolaemus bitaeniatus* | AY662062 | -- | -- | -- | -- | AF099219 | -- | -- | -- | -- | -- | -- |
| *Liolaemus boulengeri* | JF272801 | -- | -- | -- | AY389294 | -- | -- | -- | -- | -- | -- | -- |
| *Liolaemus buergeri* | AY173912 | -- | -- | AY367896 | AY850636 | AF099236 | AY367868 | -- | -- | -- | -- | -- |
| *Liolaemus calchaqui* | -- | -- | -- | JN683086 | JN683134 | -- | -- | -- | -- | -- | -- | -- |
| *Liolaemus canqueli* | DQ237524 | -- | -- | -- | DQ237638 | AY297536 | DQ237777 | -- | -- | -- | -- | -- |
| *Liolaemus capillitas* | AY173913 | -- | -- | AY367897 | AY367811 | AF099234 | AY367869 | -- | -- | -- | -- | -- |
| *Liolaemus ceii* | AY662073 | -- | -- | -- | -- | AF099237 | -- | -- | -- | -- | -- | -- |
| *Liolaemus chacoensis* | DQ237590 | -- | -- | JN683090 | AY389289 | AF099270 | DQ237828 | -- | -- | -- | -- | -- |
| *Liolaemus chaltin* | AY662061 | -- | -- | -- | -- | AF099218 | -- | -- | -- | -- | -- | -- |
| *Liolaemus chehuachekenk* | JN614944 | -- | -- | -- | JN614925 | -- | -- | -- | -- | -- | -- | -- |
| *Liolaemus chiliensis* | -- | -- | -- | -- | EU649245 | AF099224 | -- | -- | -- | -- | -- | -- |
| *Liolaemus coeruleus* | -- | -- | -- | -- | -- | AF099217 | -- | -- | -- | -- | -- | -- |
| *Liolaemus crepuscularis* | -- | -- | -- | JN683092 | JN683140 | -- | -- | -- | -- | -- | -- | -- |
| *Liolaemus cuyanus* | DQ237592 | -- | -- | DQ237482 | DQ237710 | DQ002486 | DQ237466 | -- | -- | -- | -- | -- |
| *Liolaemus cyanogaster* | EU649176 | -- | -- | -- | EU649247 | -- | -- | -- | -- | -- | -- | -- |
| *Liolaemus darwinii* | DQ237530 | -- | -- | AY367883 | DQ237650 | DQ002490 | AY367855 | -- | -- | -- | -- | -- |
| *Liolaemus dicktracy* | AY367844 | -- | -- | AY367902 | AY367816 | -- | AY367873 | -- | -- | -- | -- | -- |
| *Liolaemus donosobarrosi* | DQ237550 | -- | -- | -- | DQ237324 | AY661899 | DQ237788 | -- | -- | -- | -- | -- |
| *Liolaemus dorbignyi* | -- | -- | -- | -- | -- | AF099248 | -- | -- | -- | -- | -- | -- |
| *Liolaemus elongatus* | AY662079 | -- | -- | AY367881 | AY173855 | AY661898 | AY367853 | JN703030 | -- | JN703074 | -- | -- |
| *Liolaemus escarchadosi* | JF272805 | -- | -- | -- | JF272772 | -- | -- | -- | -- | -- | -- | -- |
| *Liolaemus espinozai* | EU795765 | -- | -- | JN683098 | JN683146 | -- | -- | -- | -- | -- | -- | -- |
| *Liolaemus fabiani* | -- | -- | -- | -- | -- | AF305793 | -- | -- | -- | -- | -- | -- |
| *Liolaemus famatinae* | DQ237469 | -- | -- | DQ237477 | DQ237485 | AF099246 | DQ237461 | -- | -- | -- | -- | -- |
| *Liolaemus fitzingerii* | DQ237572 | -- | -- | DQ237480 | DQ237691 | AF099253 | DQ237810 | -- | -- | -- | -- | -- |
| *Liolaemus fuscus* | -- | -- | -- | -- | -- | AF099232 | -- | -- | -- | -- | -- | -- |
| *Liolaemus gallardoi* | JF272806 | -- | -- | -- | JF272773 | AY661902 | -- | -- | -- | -- | -- | -- |
| *Liolaemus gracilis* | AY367833 | -- | -- | AY367890 | DQ989774 | AF099222 | AY367862 | -- | -- | -- | -- | -- |
| *Liolaemus gravenhorstii* | -- | -- | -- | -- | -- | AY297527 | -- | -- | -- | -- | -- | -- |
| *Liolaemus grosseorum* | -- | -- | -- | JN683102 | AY389284 | AF099272 | -- | -- | -- | -- | -- | -- |
| *Liolaemus hatcheri* | JF272808 | -- | -- | -- | JF272775 | AY661904 | -- | -- | -- | -- | -- | -- |
| *Liolaemus heliodermis* | AY662064 | -- | -- | -- | -- | -- | -- | -- | -- | -- | -- | -- |
| *Liolaemus hermannunezi* | -- | -- | -- | -- | -- | DQ385512 | -- | -- | -- | -- | -- | -- |
| *Liolaemus hernani* | -- | -- | -- | -- | -- | AY297529 | -- | -- | -- | -- | -- | -- |
| *Liolaemus huacahuasicus* | -- | -- | -- | -- | -- | AY297533 | -- | -- | -- | -- | -- | -- |
| *Liolaemus inacayali* | DQ237502 | -- | -- | DQ237478 | DQ237486 | -- | DQ237729 | -- | -- | -- | -- | -- |
| *Liolaemus irregularis* | DQ237556 | -- | -- | JN683104 | DQ237676 | AF099268 | DQ237794 | -- | -- | -- | -- | -- |
| *Liolaemus kingii* | DQ237473 | -- | -- | DQ237481 | DQ989771 | -- | DQ237465 | -- | -- | -- | -- | -- |
| *Liolaemus kolengh* | JF272810 | -- | -- | -- | JF272777 | -- | -- | -- | -- | -- | -- | -- |
| *Liolaemus koslowskyi* | EU795753 | -- | -- | JN683108 | EU822969 | AF099264 | DQ237760 | -- | -- | -- | -- | -- |
| *Liolaemus kriegi* | AY173883 | -- | -- | -- | AY173814 | AY297530 | AY173785 | -- | -- | -- | -- | -- |
| *Liolaemus laurenti* | EU795759 | -- | -- | JN683110 | AY389256 | AF099273 | DQ237823 | -- | -- | -- | -- | -- |
| *Liolaemus lavillai* | EU795762 | -- | -- | JN683114 | JN683158 | -- | -- | -- | -- | -- | -- | -- |
| *Liolaemus lemniscatus* | AY662054 | -- | -- | -- | EU649127 | AF099229 | -- | -- | -- | -- | -- | -- |
| *Liolaemus leopardinus* | -- | -- | -- | -- | -- | AF099235 | -- | -- | -- | -- | -- | -- |
| *Liolaemus lineomaculatus* | AY173929 | -- | -- | AY367903 | DQ237727 | AF099241 | AY367875 | -- | -- | -- | FJ356740 | -- |
| *Liolaemus lutzae* | -- | -- | -- | -- | -- | AF099255 | -- | -- | -- | -- | -- | -- |
| *Liolaemus magellanicus* | JF272812 | -- | -- | -- | JF272779 | AF099243 | -- | -- | -- | -- | -- | -- |
| *Liolaemus melanops* | DQ237555 | -- | -- | DQ237476 | DQ237673 | DQ002488 | DQ237731 | -- | -- | -- | -- | -- |
| *Liolaemus molinai* | -- | -- | -- | -- | -- | AF305795 | -- | -- | -- | -- | -- | -- |
| *Liolaemus monticola* | EU649303 | -- | -- | -- | AY851728 | AF099230 | -- | -- | -- | -- | -- | -- |
| *Liolaemus morenoi* | DQ237608 | -- | -- | -- | DQ237726 | -- | DQ237846 | -- | -- | -- | -- | -- |
| *Liolaemus multicolor* | -- | -- | -- | -- | -- | AF099250 | -- | -- | -- | -- | -- | -- |
| *Liolaemus multimaculatus* | DQ237602 | -- | -- | -- | DQ237720 | AF099257 | DQ237840 | -- | -- | -- | -- | -- |
| *Liolaemus nigromaculatus* | -- | -- | -- | -- | EU220834 | -- | -- | -- | -- | -- | -- | -- |
| *Liolaemus nigroviridis* | -- | -- | -- | -- | AY850633 | AF099233 | -- | -- | -- | -- | -- | -- |
| *Liolaemus nitidus* | -- | -- | -- | -- | EU220835 | AF099231 | -- | -- | -- | -- | -- | -- |
| *Liolaemus occipitalis* | -- | -- | -- | -- | -- | AF099256 | -- | -- | -- | -- | -- | -- |
| *Liolaemus olongasta* | DQ237528 | -- | -- | JN683116 | AY389288 | AF099271 | DQ237766 | -- | -- | -- | -- | -- |
| *Liolaemus orientalis* | -- | -- | -- | -- | -- | AF099247 | -- | -- | -- | -- | -- | -- |
| *Liolaemus ornatus* | -- | -- | -- | JN683118 | JN683168 | AF099266 | -- | -- | -- | -- | -- | -- |
| *Liolaemus pagaburoi* | AY662058 | -- | -- | -- | -- | -- | -- | -- | -- | -- | -- | -- |
| *Liolaemus paulinae* | -- | -- | -- | -- | -- | AY297531 | -- | -- | -- | -- | -- | -- |
| *Liolaemus petrophilus* | JF272813 | -- | -- | AY367898 | AY173849 | AF099238 | AY367849 | -- | -- | -- | -- | -- |
| *Liolaemus pictus* | EU649355 | -- | AY987974 | AY367877 | EU649409 | U82684 | AY367848 | -- | -- | -- | AY662595 | -- |
| *Liolaemus platei* | -- | -- | -- | -- | AY850635 | AY297528 | -- | -- | -- | -- | -- | -- |
| *Liolaemus pseudoanomalus* | AY173881 | -- | -- | AY367882 | AY173812 | AF099254 | AY367854 | -- | -- | -- | -- | -- |
| *Liolaemus pseudolemniscatus* | -- | -- | -- | -- | EU220833 | -- | -- | -- | -- | -- | -- | -- |
| *Liolaemus puna* | AY662059 | -- | -- | -- | -- | AF305790 | -- | -- | -- | -- | -- | -- |
| *Liolaemus quilmes* | DQ237596 | -- | -- | JN683122 | DQ237714 | AF099265 | DQ237834 | -- | -- | -- | -- | -- |
| *Liolaemus ramirezae* | DQ237597 | -- | -- | -- | DQ989772 | -- | DQ237835 | -- | -- | -- | -- | -- |
| *Liolaemus reichei* | -- | -- | -- | -- | -- | AF305794 | -- | -- | -- | -- | -- | -- |
| *Liolaemus riojanus* | -- | -- | -- | -- | -- | AY297534 | -- | -- | -- | -- | -- | -- |
| *Liolaemus robertmertensi* | DQ237499 | -- | -- | -- | DQ989769 | AF099220 | DQ237736 | -- | -- | -- | -- | -- |
| *Liolaemus rothi* | DQ237513 | -- | -- | -- | DQ237718 | AF099262 | DQ237838 | -- | -- | -- | -- | -- |
| *Liolaemus ruibali* | DQ237525 | -- | -- | -- | DQ237646 | AF099244 | DQ237763 | -- | -- | -- | -- | -- |
| *Liolaemus salinicola* | DQ237527 | -- | -- | -- | DQ237648 | AF099259 | DQ237765 | -- | -- | -- | -- | -- |
| *Liolaemus sarmientoi* | JF272815 | -- | -- | -- | JF272782 | AY661907 | -- | -- | -- | -- | -- | -- |
| *Liolaemus saxatilis* | -- | -- | -- | -- | DQ989773 | -- | -- | -- | -- | -- | -- | -- |
| *Liolaemus scapularis* | DQ237595 | L41447 | -- | -- | DQ237713 | AF099258 | DQ237833 | -- | -- | -- | -- | -- |
| *Liolaemus schroederi* | -- | -- | -- | -- | -- | AF305791 | -- | -- | -- | -- | -- | -- |
| *Liolaemus scolaroi* | JF272816 | -- | -- | -- | JF272783 | -- | -- | -- | -- | -- | -- | -- |
| *Liolaemus silvanae* | JF272817 | -- | -- | -- | JF272784 | -- | -- | -- | -- | -- | -- | -- |
| *Liolaemus somuncurae* | JF272818 | -- | -- | -- | JF272785 | AF099242 | -- | -- | -- | -- | -- | -- |
| *Liolaemus tari* | JF272826 | -- | -- | -- | JF272793 | AY661906 | -- | -- | -- | -- | -- | -- |
| *Liolaemus telsen* | JN614935 | -- | -- | -- | JN614916 | -- | -- | -- | -- | -- | -- | -- |
| *Liolaemus tenuis* | EU649302 | -- | -- | -- | EU649243 | AF099228 | -- | -- | -- | -- | -- | -- |
| *Liolaemus thermarum* | AY662076 | -- | -- | -- | -- | -- | -- | -- | -- | -- | -- | -- |
| *Liolaemus tristis* | JF272827 | -- | -- | -- | JF272794 | -- | -- | -- | -- | -- | -- | -- |
| *Liolaemus umbrifer* | -- | -- | -- | AY367901 | AY367814 | -- | AY367871 | -- | -- | -- | -- | -- |
| *Liolaemus uptoni* | JF272828 | -- | -- | -- | JF272795 | -- | -- | -- | -- | -- | -- | -- |
| *Liolaemus uspallatensis* | DQ237601 | -- | -- | JN683124 | DQ237719 | AF099269 | DQ237839 | -- | -- | -- | -- | -- |
| *Liolaemus vallecurensis* | AY367838 | -- | -- | AY367894 | AY173546 | -- | AY367866 | -- | -- | -- | -- | -- |
| *Liolaemus walkeri* | AY662057 | -- | -- | -- | -- | -- | -- | -- | -- | -- | -- | -- |
| *Liolaemus wiegmannii* | DQ237471 | -- | -- | DQ237479 | DQ237709 | AF099260 | DQ237463 | -- | -- | -- | -- | -- |
| *Liolaemus xanthoviridis* | -- | -- | -- | -- | -- | AY297535 | -- | -- | -- | -- | -- | -- |
| *Liolaemus yanalcu* | AY662056 | -- | -- | -- | -- | -- | -- | -- | -- | -- | -- | -- |
| *Liolaemus zapallarensis* | -- | L41448 | -- | -- | -- | AF099227 | -- | -- | -- | -- | -- | -- |
| *Liolaemus zullyi* | -- | -- | -- | -- | -- | AY661903 | -- | -- | -- | -- | -- | -- |
| *Liophidium chabaudi* | FJ404173 | FJ404210 | -- | FJ387210 | EU394721 | -- | FJ404372 | -- | -- | -- | -- | FJ404446 |
| *Liophidium mayottensis* | -- | GQ913670 | -- | -- | -- | -- | -- | -- | -- | -- | -- | -- |
| *Liophidium rhodogaster* | -- | GQ913672 | -- | DQ979971 | DQ979992 | -- | -- | -- | -- | -- | -- | -- |
| *Liophidium therezieni* | -- | GQ913673 | -- | -- | -- | -- | -- | -- | -- | -- | -- | -- |
| *Liophidium torquatum* | -- | AY188062 | -- | AY187984 | AY188023 | -- | -- | -- | -- | -- | -- | -- |
| *Liophidium vaillanti* | -- | GQ913674 | -- | GQ913668 | DQ979995 | -- | -- | -- | -- | -- | -- | -- |
| *Liophis almadensis* | JQ598808 | JQ598871 | -- | JQ598979 | -- | -- | -- | -- | -- | -- | -- | -- |
| *Liophis amarali* | GQ457807 | GQ457747 | -- | GQ457867 | JQ598921 | -- | -- | -- | -- | -- | -- | -- |
| *Liophis anomalus* | JQ598817 | JQ598879 | -- | -- | -- | -- | -- | -- | -- | -- | -- | -- |
| *Liophis atraventer* | JQ598809 | JQ598872 | -- | JQ598980 | -- | -- | -- | -- | -- | -- | -- | -- |
| *Liophis breviceps* | AF158464 | AF158533 | -- | -- | -- | -- | -- | -- | -- | -- | -- | -- |
| *Liophis ceii* | JQ598810 | JQ598873 | -- | JQ598981 | -- | -- | -- | -- | -- | -- | -- | -- |
| *Liophis elegantissimus* | GQ457808 | GQ457748 | -- | GQ457868 | -- | -- | -- | -- | -- | -- | -- | -- |
| *Liophis epinephelus* | GU018158 | GU018176 | -- | -- | -- | -- | -- | -- | -- | -- | -- | -- |
| *Liophis flavifrenatus* | JQ598818 | JQ598880 | -- | -- | -- | -- | -- | -- | -- | -- | -- | -- |
| *Liophis jaegeri* | GQ457809 | GQ457749 | -- | GQ457869 | -- | -- | -- | -- | -- | -- | -- | -- |
| *Liophis juliae* | AF158445 | AF158514 | -- | -- | -- | -- | -- | -- | -- | -- | -- | -- |
| *Liophis lineatus* | -- | -- | DQ469795 | DQ469789 | -- | -- | -- | DQ469793 | -- | -- | DQ469791 | -- |
| *Liophis meridionalis* | GQ457810 | GQ457750 | -- | GQ457870 | -- | -- | -- | -- | -- | -- | -- | -- |
| *Liophis miliaris* | JQ598811 | JQ598874 | JQ599025 | JQ598982 | JQ598931 | -- | -- | -- | -- | -- | -- | -- |
| *Liophis paucidens* | JQ598819 | -- | -- | JQ598987 | -- | -- | -- | -- | -- | -- | -- | -- |
| *Liophis poecilogyrus* | JQ598812 | JQ598875 | -- | -- | -- | -- | -- | -- | -- | -- | -- | -- |
| *Liophis reginae* | JQ598813 | JQ598876 | -- | JQ598983 | -- | -- | -- | -- | -- | -- | -- | -- |
| *Liophis typhlus* | GQ457811 | GQ457751 | -- | GQ457871 | -- | -- | -- | -- | -- | -- | -- | -- |
| *Liopholidophis dimorphus* | -- | DQ979966 | -- | DQ979973 | DQ979991 | -- | -- | -- | -- | -- | -- | -- |
| *Liopholidophis dolicocercus* | -- | DQ979968 | -- | DQ979975 | DQ979990 | -- | -- | -- | -- | -- | -- | -- |
| *Liopholidophis sexlineatus* | FJ404174 | AY188063 | -- | AY187985 | AY188024 | -- | FJ404373 | -- | -- | -- | -- | FJ404447 |
| *Lioscincus maruia* | -- | -- | -- | DQ675354 | -- | DQ675214 | -- | -- | -- | -- | DQ675294 | -- |
| *Lioscincus nigrofasciolatum* | EU837125 | EU837121 | -- | DQ675356 | -- | DQ675215 | -- | -- | -- | -- | EU837128 | -- |
| *Lioscincus novaecaledoniae* | -- | -- | -- | -- | -- | DQ675252 | -- | -- | -- | -- | DQ675332 | -- |
| *Lioscincus steindachneri* | EU837124 | EU837120 | -- | DQ675358 | EU837117 | DQ675218 | -- | -- | -- | -- | EU837129 | -- |
| *Lioscincus tillieri* | EU567929 | EU567923 | -- | DQ675360 | EU567833 | DQ675220 | EU567729 | -- | -- | -- | EU568022 | -- |
| *Lioscincus vivae* | -- | -- | -- | DQ675361 | -- | DQ675221 | -- | -- | -- | -- | DQ675301 | -- |
| *Liotyphlops albirostris* | Z46461 | AF366762 | FJ433960 | AF544727 | AF544672 | -- | -- | -- | -- | HQ876356 | EU402853 | FJ433890 |
| *Lipinia noctua* | JF497868 | JF497992 | HQ655175 | AF039465 | -- | JF498120 | JF498473 | -- | -- | JF498348 | -- | -- |
| *Lipinia pulchella* | JF497869 | JF497994 | HQ907220 | -- | -- | JF498121 | JF498475 | -- | -- | HQ907625 | -- | -- |
| *Lipinia vittigera* | AB028804 | AB028816 | -- | -- | -- | -- | -- | -- | -- | -- | -- | -- |
| *Lophognathus gilberti* | -- | -- | DQ340699 | DQ340659 | -- | AY133019 | -- | -- | -- | -- | HQ662518 | -- |
| *Lophognathus longirostris* | -- | -- | DQ340700 | DQ340660 | -- | AF128462 | -- | -- | -- | -- | HQ662516 | -- |
| *Lophognathus temporalis* | AB031970 | AB031987 | DQ340703 | DQ340662 | -- | AY133002 | -- | -- | -- | -- | HQ662492 | -- |
| *Loxocemus bicolor* | AF512737 | AF512737 | FJ433967 | AY099969 | AY099993 | -- | -- | FJ434072 | -- | FJ433921 | AY444061 | FJ433897 |
| *Lucasium alboguttatum* | -- | -- | -- | -- | -- | EF681774 | -- | -- | -- | -- | -- | -- |
| *Lucasium byrnei* | -- | -- | -- | FJ855474 | -- | EF681802 | -- | -- | -- | -- | FJ855453 | -- |
| *Lucasium damaeum* | -- | GU460152 | -- | AY134570 | -- | EF681776 | JQ398448 | -- | GU459754 | -- | HQ426279 | HQ426452 |
| *Lucasium immaculatum* | -- | -- | -- | -- | -- | EF681800 | -- | -- | -- | -- | -- | -- |
| *Lucasium maini* | -- | -- | -- | -- | -- | EF681778 | JQ398451 | -- | -- | -- | -- | -- |
| *Lucasium squarrosum* | -- | -- | -- | -- | -- | EF681780 | -- | -- | -- | -- | -- | -- |
| *Lucasium steindachneri* | -- | -- | -- | -- | -- | EF681799 | -- | -- | -- | -- | -- | -- |
| *Lucasium stenodactylum* | -- | -- | -- | FJ855475 | -- | EF681781 | -- | -- | JQ173676 | -- | FJ855454 | JQ945477 |
| *Lucasium wombeyi* | -- | -- | -- | -- | -- | EF681783 | -- | -- | -- | -- | -- | -- |
| *Luperosaurus cumingii* | -- | -- | -- | JQ945585 | -- | JQ437902 | -- | -- | JQ945381 | -- | JQ945313 | JQ945478 |
| *Luperosaurus iskandari* | -- | -- | -- | -- | -- | JQ437906 | -- | -- | JQ437948 | -- | -- | -- |
| *Luperosaurus joloensis* | -- | -- | -- | -- | -- | JQ437900 | -- | -- | -- | -- | -- | -- |
| *Luperosaurus macgregori* | -- | -- | -- | -- | -- | JQ437905 | -- | -- | JQ437946 | -- | -- | -- |
| *Lycodon aulicus* | -- | -- | -- | HQ735418 | HQ735416 | -- | -- | -- | -- | -- | -- | -- |
| *Lycodon capucinus* | -- | -- | -- | -- | -- | -- | U49317 | -- | -- | -- | -- | -- |
| *Lycodon fasciatus* | -- | EU999215 | -- | -- | -- | -- | -- | -- | -- | -- | -- | -- |
| *Lycodon laoensis* | Z46455 | Z46485 | -- | -- | -- | -- | -- | -- | -- | -- | -- | -- |
| *Lycodon osmanhilli* | -- | KC347364 | -- | KC347404 | -- | -- | KC347517 | -- | -- | -- | KC347441 | -- |
| *Lycodon paucifasciatus* | -- | EU999213 | -- | -- | -- | -- | -- | -- | -- | -- | -- | -- |
| *Lycodon ruhstrati* | -- | EU999209 | -- | -- | -- | -- | -- | -- | -- | -- | -- | -- |
| *Lycodon zawi* | -- | -- | -- | AF471111 | AF471040 | -- | -- | -- | -- | -- | -- | -- |
| *Lycodonomorphus laevissimus* | -- | -- | -- | DQ486162 | DQ486338 | -- | -- | -- | -- | -- | -- | -- |
| *Lycodonomorphus rufulus* | FJ404175 | FJ404199 | -- | FJ387200 | FJ404299 | -- | FJ404374 | -- | -- | -- | -- | FJ404448 |
| *Lycodonomorphus whytii* | FJ404176 | FJ404200 | -- | FJ387201 | FJ404300 | -- | FJ404375 | -- | -- | -- | -- | FJ404449 |
| *Lycodryas sanctijohannis* | FJ404177 | GU994857 | -- | AY611919 | AY612010 | -- | -- | -- | -- | -- | -- | -- |
| *Lycognathophis seychellensis* | -- | -- | -- | FJ387220 | -- | -- | -- | -- | -- | -- | -- | FJ404465 |
| *Lycophidion capense* | FJ404178 | AY611893 | EU402652 | DQ486168 | DQ486344 | -- | FJ404376 | JN703031 | -- | JN703075 | EU402855 | FJ404450 |
| *Lycophidion laterale* | FJ404179 | FJ404197 | -- | FJ387198 | FJ404297 | -- | FJ404377 | -- | -- | -- | -- | FJ404451 |
| *Lycophidion nigromaculatum* | FJ404180 | -- | -- | FJ404281 | FJ404298 | -- | -- | -- | -- | -- | -- | -- |
| *Lycophidion ornatum* | -- | -- | -- | AF471144 | AF471086 | -- | -- | -- | -- | -- | -- | -- |
| *Lygisaurus abscondita* | -- | -- | -- | -- | -- | -- | AY533656 | -- | -- | -- | -- | -- |
| *Lygisaurus aeratus* | -- | FJ379515 | -- | -- | -- | -- | AY533660 | -- | -- | -- | -- | -- |
| *Lygisaurus foliorum* | -- | FJ379517 | -- | DQ675362 | -- | DQ675222 | AJ290548 | -- | -- | -- | DQ675302 | -- |
| *Lygisaurus laevis* | -- | FJ379518 | -- | -- | -- | -- | FJ379461 | -- | -- | -- | -- | -- |
| *Lygisaurus macfarlani* | -- | -- | -- | -- | -- | -- | AJ290549 | -- | -- | -- | -- | -- |
| *Lygisaurus malleolus* | -- | FJ379520 | -- | -- | -- | -- | AY533657 | -- | -- | -- | -- | -- |
| *Lygisaurus novaeguineae* | AY218014 | AY217964 | -- | AY217861 | AY217810 | -- | AJ290523 | -- | -- | -- | -- | -- |
| *Lygisaurus parrhasius* | -- | -- | -- | -- | -- | -- | AJ290525 | -- | -- | -- | -- | -- |
| *Lygisaurus sesbrauna* | -- | -- | -- | -- | -- | -- | AJ290550 | -- | -- | -- | -- | -- |
| *Lygisaurus tanneri* | -- | FJ379523 | -- | -- | -- | -- | AJ290551 | -- | -- | -- | -- | -- |
| *Lygodactylus angularis* | -- | HQ872460 | -- | -- | -- | -- | -- | -- | -- | -- | -- | -- |
| *Lygodactylus arnoulti* | -- | GU593462 | -- | -- | GU593523 | -- | -- | -- | -- | -- | GU593585 | GU593395 |
| *Lygodactylus blancae* | -- | JF934723 | -- | -- | -- | -- | -- | -- | -- | -- | -- | -- |
| *Lygodactylus bradfieldi* | DQ852705 | GU593426 | -- | AY172935 | -- | EU423279 | -- | -- | HQ426212 | -- | HQ426301 | GU593360 |
| *Lygodactylus capensis* | -- | HQ872461 | -- | -- | GU593510 | -- | -- | -- | -- | -- | -- | GU593375 |
| *Lygodactylus chobiensis* | -- | GU593457 | -- | -- | GU593520 | -- | -- | -- | -- | -- | -- | GU593390 |
| *Lygodactylus conraui* | -- | GU593434 | -- | -- | GU593502 | -- | -- | -- | -- | -- | -- | GU593368 |
| *Lygodactylus expectatus* | -- | -- | JQ073112 | -- | -- | -- | -- | -- | -- | -- | JQ073233 | -- |
| *Lygodactylus gravis* | -- | GU593432 | -- | -- | GU593500 | -- | -- | -- | -- | -- | GU593556 | GU593366 |
| *Lygodactylus guibei* | -- | GU593463 | -- | -- | -- | -- | -- | -- | -- | -- | GU593587 | GU593397 |
| *Lygodactylus gutturalis* | -- | GU593460 | -- | -- | GU593522 | -- | -- | -- | -- | -- | GU593583 | GU593393 |
| *Lygodactylus heterurus* | -- | GU593491 | -- | -- | GU593546 | -- | -- | -- | -- | -- | GU593611 | GU593421 |
| *Lygodactylus keniensis* | -- | GU593476 | -- | -- | GU593534 | -- | -- | -- | -- | -- | -- | GU593408 |
| *Lygodactylus kimhowelli* | -- | GU593479 | -- | -- | GU593535 | -- | -- | -- | -- | -- | -- | GU593409 |
| *Lygodactylus klugei* | -- | -- | -- | HQ426555 | -- | -- | -- | -- | HQ426209 | -- | HQ426298 | HQ426471 |
| *Lygodactylus lawrencei* | -- | GU593428 | -- | -- | GU593496 | -- | -- | -- | -- | -- | GU593552 | GU593362 |
| *Lygodactylus luteopicturatus* | -- | -- | -- | FJ830142 | FJ830051 | -- | -- | -- | -- | -- | FJ830234 | -- |
| *Lygodactylus madagascariensis* | EU596612 | AY653258 | EU596846 | -- | EU596695 | -- | -- | -- | -- | -- | -- | -- |
| *Lygodactylus miops* | -- | AY653263 | -- | HQ426556 | -- | -- | -- | -- | HQ426210 | -- | HQ426299 | HQ426472 |
| *Lygodactylus mirabilis* | -- | GU593483 | -- | HQ426557 | GQ910848 | JX041382 | -- | -- | HQ426211 | -- | HQ426300 | GU593413 |
| *Lygodactylus montanus* | -- | GU593481 | -- | -- | GU593536 | -- | -- | -- | -- | -- | GU593601 | GU593411 |
| *Lygodactylus pauliani* | -- | AY653264 | -- | -- | -- | -- | -- | -- | -- | -- | -- | -- |
| *Lygodactylus picturatus* | -- | FJ829971 | JQ073114 | -- | FJ830051 | -- | -- | -- | -- | -- | JQ073235 | FJ830325 |
| *Lygodactylus pictus* | -- | GU593455 | -- | -- | GU593518 | -- | -- | -- | -- | -- | -- | GU593388 |
| *Lygodactylus rarus* | -- | AY653274 | -- | -- | -- | -- | -- | -- | -- | -- | -- | -- |
| *Lygodactylus stevensoni* | -- | GU593453 | -- | -- | GU593516 | -- | -- | -- | -- | -- | -- | GU593386 |
| *Lygodactylus thomensis* | -- | GU593485 | -- | -- | GU593541 | -- | -- | -- | -- | -- | -- | GU593415 |
| *Lygodactylus tolampyae* | -- | GU593466 | JQ073113 | HQ426559 | GU593526 | JX041383 | -- | -- | HQ426213 | -- | HQ426302 | GU593399 |
| *Lygodactylus tuberosus* | -- | GU593469 | -- | -- | -- | -- | -- | -- | -- | -- | -- | GU593402 |
| *Lygodactylus verticillatus* | -- | GU593471 | -- | -- | GU593529 | -- | -- | -- | -- | -- | GU593595 | GU593404 |
| *Lygodactylus williamsi* | -- | GU593423 | -- | -- | GU593492 | -- | -- | -- | -- | -- | -- | GU593357 |
| *Lygosoma afrum* | AY308413 | AY308261 | -- | -- | -- | -- | -- | -- | -- | -- | -- | -- |
| *Lygosoma albopunctata* | AY308414 | AY308262 | -- | -- | -- | -- | -- | -- | -- | -- | -- | -- |
| *Lygosoma bowringii* | AB028775 | AY308263 | HQ655177 | HQ655212 | -- | HQ907430 | -- | -- | -- | HQ907637 | -- | -- |
| *Lygosoma koratense* | AB028805 | AY308269 | -- | -- | -- | -- | -- | -- | -- | -- | -- | -- |
| *Lygosoma lineolatum* | AY308422 | AY308271 | -- | -- | -- | -- | -- | -- | -- | -- | -- | -- |
| *Lygosoma punctata* | -- | -- | -- | -- | -- | DQ675265 | -- | -- | -- | -- | DQ675345 | -- |
| *Lygosoma quadrupes* | AB028806 | AB028818 | HQ907232 | -- | -- | HQ907432 | -- | -- | -- | HQ907639 | -- | -- |
| *Lyriocephalus scutatus* | -- | -- | -- | -- | -- | AF364052 | -- | -- | -- | -- | -- | -- |
| *Lystrophis dorbignyi* | GQ457812 | GQ457752 | -- | -- | -- | -- | -- | -- | -- | -- | -- | -- |
| *Lystrophis histricus* | GQ457813 | GQ457753 | JQ599061 | -- | JQ598962 | -- | -- | -- | -- | -- | -- | -- |
| *Lystrophis matogrossensis* | JQ598850 | JQ598910 | -- | -- | -- | -- | -- | -- | -- | -- | -- | -- |
| *Lystrophis nattereri* | JQ598851 | JQ598912 | -- | -- | -- | -- | -- | -- | -- | -- | -- | -- |
| *Lystrophis pulcher* | JQ598852 | JQ598913 | -- | -- | -- | -- | -- | -- | -- | -- | -- | -- |
| *Lystrophis semicinctus* | GU018156 | GU018173 | -- | -- | GQ895877 | -- | -- | -- | -- | -- | -- | -- |
| *Lytorhynchus diadema* | AY647229 | AY188064 | -- | AY187986 | AY188025 | -- | -- | -- | -- | -- | -- | -- |
| *Mabuya agilis* | DQ239207 | AY151468 | -- | DQ239014 | EU443102 | -- | -- | -- | -- | -- | -- | -- |
| *Mabuya agmosticha* | DQ239215 | DQ238893 | -- | DQ238974 | DQ239134 | -- | -- | -- | -- | -- | -- | -- |
| *Mabuya altamazonica* | GQ982525 | -- | -- | -- | EU443103 | -- | -- | -- | -- | -- | -- | -- |
| *Mabuya bistriata* | DQ239240 | DQ238936 | -- | DQ239026 | EU443105 | -- | -- | -- | -- | -- | -- | -- |
| *Mabuya caissara* | AF548787 | AF549176 | -- | -- | -- | -- | -- | -- | -- | -- | -- | -- |
| *Mabuya carvalhoi* | DQ239267 | DQ238945 | -- | DQ239025 | EU443106 | -- | -- | -- | -- | -- | -- | -- |
| *Mabuya cochabambae* | AF202625 | AF202630 | -- | -- | -- | -- | -- | -- | -- | -- | -- | -- |
| *Mabuya croizati* | EU477260 | -- | -- | -- | EU443107 | -- | -- | -- | -- | -- | -- | -- |
| *Mabuya dorsivittata* | AY070346 | AY151460 | -- | DQ239015 | DQ239176 | -- | -- | -- | -- | -- | -- | -- |
| *Mabuya falconensis* | EU477261 | -- | -- | -- | EU443110 | -- | -- | -- | -- | -- | -- | -- |
| *Mabuya frenata* | DQ239209 | DQ238932 | -- | DQ239012 | EU443111 | -- | -- | -- | -- | -- | -- | -- |
| *Mabuya guaporicola* | DQ239222 | DQ238928 | -- | DQ239008 | EU443113 | -- | -- | -- | -- | -- | -- | -- |
| *Mabuya heathi* | DQ239232 | AY151462 | -- | DQ239009 | DQ239170 | -- | -- | -- | -- | -- | -- | -- |
| *Mabuya mabouya* | EU477265 | AY070357 | -- | -- | EU443115 | JF498123 | -- | -- | -- | JF498350 | -- | -- |
| *Mabuya macrophthalma* | AY159048 | AY159077 | -- | -- | -- | -- | -- | -- | -- | -- | -- | -- |
| *Mabuya macrorhyncha* | DQ239213 | DQ238921 | -- | DQ239001 | DQ239162 | -- | -- | -- | -- | -- | -- | -- |
| *Mabuya meridensis* | EU477267 | -- | -- | -- | EU443117 | -- | -- | -- | -- | -- | -- | -- |
| *Mabuya nigropalmata* | EU515211 | -- | -- | -- | EU515213 | -- | -- | -- | -- | -- | -- | -- |
| *Mabuya nigropunctata* | EU477268 | AY308280 | -- | -- | EU443121 | -- | -- | -- | -- | -- | -- | -- |
| *Mabuya sloanii* | EU477270 | -- | -- | -- | EU443123 | -- | -- | -- | -- | -- | -- | -- |
| *Mabuya unimarginata* | DQ368665 | AB057393 | -- | -- | EU443129 | JF498124 | JF498476 | -- | -- | JF498351 | -- | -- |
| *Macrelaps microlepidotus* | FJ404137 | AY611810 | -- | AY611902 | AY611993 | -- | FJ404340 | -- | -- | -- | -- | FJ404412 |
| *Macropisthodon rudis* | -- | -- | -- | JQ687442 | GQ281780 | JQ687458 | JQ687417 | -- | -- | -- | -- | -- |
| *Macroprotodon abubakeri* | AY643297 | AY643338 | -- | -- | AY643383 | -- | -- | -- | -- | -- | -- | -- |
| *Macroprotodon cucullatus* | AY643290 | AY188065 | -- | AY187987 | AY188026 | AY487025 | AY487064 | -- | -- | -- | -- | -- |
| *Macroscincus coctei* | AF280181 | -- | -- | -- | AF280314 | -- | -- | -- | -- | -- | -- | -- |
| *Macrovipera deserti* | -- | AJ275765 | -- | -- | AJ275712 | -- | -- | -- | -- | -- | -- | -- |
| *Macrovipera lebetina* | EU624260 | EU624294 | -- | -- | AJ275713 | -- | DQ897729 | -- | -- | -- | -- | -- |
| *Macrovipera mauritanica* | EU624261 | EU624295 | -- | -- | AJ275714 | -- | EU624229 | -- | -- | -- | -- | -- |
| *Macrovipera schweizeri* | EU624262 | AJ275768 | -- | -- | AJ275715 | -- | -- | -- | -- | -- | -- | -- |
| *Maculophis bella* | -- | -- | -- | DQ902097 | DQ902134 | DQ902248 | DQ902316 | -- | -- | -- | -- | -- |
| *Madagascarophis colubrinus* | -- | AY586216 | -- | AY187989 | AY188028 | -- | U49313 | -- | -- | -- | -- | -- |
| *Madagascarophis meridionalis* | FJ404181 | AY586213 | JQ073077 | AY187988 | AY188027 | -- | FJ404378 | -- | -- | -- | JQ073198 | FJ404452 |
| *Madascincus igneocaudatus* | -- | FJ667629 | FJ667648 | AY818795 | AF280125 | -- | -- | -- | FJ667706 | -- | -- | FJ667735 |
| *Madascincus intermedius* | -- | -- | FJ667649 | AY391181 | AY391163 | -- | -- | -- | FJ667707 | -- | -- | FJ667736 |
| *Madascincus melanopleura* | AY315481 | AY802764 | HM160578 | AY802768 | AY802770 | -- | -- | -- | -- | HM161052 | HM161147 | HQ913901 |
| *Madascincus mouroundavae* | -- | -- | FJ667650 | AY391185 | AY391167 | -- | -- | -- | FJ667708 | -- | -- | FJ667737 |
| *Madascincus stumpffi* | AY315497 | AY391152 | -- | AY391188 | AY391170 | -- | -- | -- | -- | -- | -- | HQ913911 |
| *Malpolon moilensis* | AY643313 | AY643355 | -- | DQ486157 | DQ486333 | -- | DQ486309 | -- | -- | -- | -- | -- |
| *Malpolon monspessulanus* | FJ404116 | AY643354 | -- | AY187990 | AY188029 | -- | FJ404320 | -- | -- | -- | -- | FJ404390 |
| *Manolepis putnami* | JQ598820 | JQ598881 | JQ599035 | JQ598988 | JQ598936 | -- | -- | -- | -- | -- | -- | -- |
| *Mantheyus phuwuanensis* | AB023750 | AB023772 | -- | -- | -- | AY555836 | -- | -- | -- | -- | FJ356735 | -- |
| *Marmorosphax montana* | -- | -- | -- | DQ675392 | -- | DQ675255 | -- | -- | -- | -- | DQ675335 | -- |
| *Marmorosphax tricolor* | EU567932 | EU567925 | -- | DQ675367 | EU567831 | EU423133 | EU423131 | -- | -- | -- | EU568023 | -- |
| *Masticophis flagellum* | AY122823 | -- | -- | AY234228 | AY486928 | AY487021 | AY487060 | -- | -- | -- | -- | -- |
| *Masticophis taeniatus* | AY122669 | -- | -- | -- | -- | -- | -- | -- | -- | -- | -- | -- |
| *Mastigodryas bifossatus* | HM565763 | -- | -- | -- | -- | -- | -- | -- | -- | -- | -- | -- |
| *Mastigodryas boddaerti* | AF158431 | AF158496 | -- | GQ895811 | GQ895867 | -- | -- | -- | -- | -- | -- | -- |
| *Mastigodryas melanolomus* | -- | -- | -- | -- | GQ895868 | -- | -- | -- | -- | -- | -- | -- |
| *Matoatoa brevipes* | EU596614 | -- | EU596848 | JQ945587 | EF490751 | EF490777 | -- | -- | EF490698 | -- | EF490724 | JQ945480 |
| *Mehelya capensis* | AF544769 | -- | FJ433995 | AF544703 | HQ207116 | -- | AF544665 | FJ434096 | -- | EF144079 | AY487379 | EF144106 |
| *Mehelya nyassae* | FJ404182 | AY611896 | -- | -- | AY235726 | -- | FJ404379 | -- | -- | -- | -- | FJ404453 |
| *Mehelya poensis* | FJ404183 | AY611863 | -- | AY611954 | AY612045 | -- | FJ404380 | -- | -- | -- | -- | FJ404454 |
| *Mehelya stenophthalmus* | FJ404185 | AY611835 | -- | AY611927 | AY612018 | -- | FJ404382 | -- | -- | -- | -- | FJ404456 |
| *Melanophidium punctatum* | AY700993 | AY701024 | -- | -- | -- | -- | -- | -- | -- | -- | -- | -- |
| *Melanoseps ater* | DQ316885 | DQ316893 | -- | -- | DQ316877 | -- | -- | -- | -- | -- | -- | -- |
| *Melanoseps loveridgei* | DQ316886 | DQ316894 | -- | -- | DQ316878 | -- | -- | -- | -- | -- | -- | -- |
| *Melanoseps occidentalis* | AY391123 | AY649166 | HM160592 | AY217870 | AY217819 | -- | -- | -- | -- | HM161066 | HM161161 | -- |
| *Menetia alanae* | -- | -- | -- | -- | -- | -- | AY364910 | -- | -- | -- | -- | -- |
| *Menetia greyii* | -- | -- | -- | -- | -- | DQ675266 | AY364930 | -- | -- | -- | DQ675346 | -- |
| *Menetia timlowi* | -- | FJ379526 | -- | -- | -- | -- | AJ290552 | -- | -- | -- | -- | -- |
| *Meroles anchietae* | AY192426 | AY192417 | -- | -- | AY184401 | AY184391 | -- | -- | -- | -- | -- | -- |
| *Meroles ctenodactylus* | AF206610 | AY192418 | -- | -- | AY184402 | AY184392 | -- | -- | -- | -- | -- | -- |
| *Meroles cuneirostris* | AF042545 | AY192419 | -- | -- | AY184403 | AY184393 | -- | -- | -- | -- | -- | -- |
| *Meroles knoxii* | AY192433 | DQ871147 | -- | -- | AY184407 | AY184397 | -- | -- | -- | -- | DQ871205 | -- |
| *Meroles micropholidotus* | AY192430 | AY192420 | -- | -- | AY184404 | AY184394 | -- | -- | -- | -- | -- | -- |
| *Meroles reticulatus* | AF042543 | DQ871144 | -- | -- | AY184405 | AY184395 | -- | -- | -- | -- | DQ871202 | -- |
| *Meroles suborbitalis* | AF042542 | DQ871145 | -- | EF632273 | AF206540 | AY184396 | -- | -- | -- | -- | EF632230 | -- |
| *Mesalina adramitana* | AF080359 | AY035843 | -- | -- | -- | -- | -- | -- | -- | -- | -- | -- |
| *Mesalina bahaeldini* | -- | EF555287 | -- | -- | EF555246 | -- | -- | -- | -- | -- | -- | -- |
| *Mesalina balfouri* | AY035825 | AY035835 | -- | -- | -- | -- | -- | -- | -- | -- | -- | -- |
| *Mesalina brevirostris* | AF206606 | EF555309 | -- | -- | FJ416173 | -- | -- | -- | -- | -- | -- | -- |
| *Mesalina guttulata* | AY218019 | AY217969 | -- | EF632274 | AY217815 | -- | -- | -- | -- | -- | EF632231 | -- |
| *Mesalina olivieri* | AY035829 | EF555323 | -- | -- | EF555281 | -- | -- | -- | -- | -- | -- | -- |
| *Mesalina rubropunctata* | AY035830 | EF555316 | -- | EF632275 | EF555274 | -- | -- | -- | -- | -- | EF632232 | -- |
| *Mesalina simoni* | -- | EF555301 | -- | -- | EF555259 | -- | -- | -- | -- | -- | -- | -- |
| *Mesaspis gadovii* | AF056615 | -- | -- | -- | AF056600 | -- | AY605104 | -- | -- | -- | -- | -- |
| *Mesaspis moreletii* | -- | -- | -- | JN090139 | -- | AF085616 | -- | -- | -- | -- | -- | -- |
| *Mesoscincus managuae* | AY308433 | AY649156 | -- | -- | -- | -- | -- | -- | -- | -- | -- | -- |
| *Mesoscincus schwartzei* | AY649117 | AY649158 | HM160593 | -- | -- | -- | -- | -- | -- | HM161067 | HM161162 | -- |
| *Micrablepharus atticolus* | AF420664 | AF420718 | -- | AF420826 | -- | -- | AF420904 | -- | -- | -- | -- | -- |
| *Micrablepharus maximiliani* | AF420657 | AF420730 | -- | AF420850 | -- | -- | AF420875 | -- | -- | -- | -- | -- |
| *Micrelaps bicoloratus* | -- | -- | -- | DQ486173 | DQ486349 | -- | -- | -- | -- | -- | -- | -- |
| *Microacontias lineatus* | DQ249008 | HQ180032 | -- | DQ249042 | DQ249087 | -- | -- | -- | -- | -- | DQ249107 | -- |
| *Microacontias litoralis* | AY217996 | HQ180030 | -- | AY217843 | AY217791 | -- | -- | -- | -- | -- | -- | -- |
| *Microlophus albemarlensis* | FJ470007 | EF615698 | -- | EF615771 | EF616062 | AY625189 | FJ459313 | -- | -- | -- | EF616423 | -- |
| *Microlophus atacamensis* | EF615600 | EF615676 | -- | EF615749 | EF616037 | AF528752 | EF616332 | -- | -- | -- | EF616401 | -- |
| *Microlophus bivittatus* | EF615612 | EF615681 | -- | EF615754 | EF616045 | AY625164 | EF616334 | -- | -- | -- | EF616406 | -- |
| *Microlophus delanonis* | EF615613 | EF615684 | -- | EF615757 | EF616048 | AY625169 | EF616337 | -- | -- | -- | EF616409 | -- |
| *Microlophus duncanensis* | FJ458481 | EF615685 | -- | EF615758 | EF616049 | AY625184 | EF616338 | -- | -- | -- | EF616410 | -- |
| *Microlophus grayii* | EF615618 | EF615687 | -- | EF615760 | EF616051 | AY625170 | EF616340 | -- | -- | -- | EF616411 | -- |
| *Microlophus habelii* | EF615619 | EF615689 | -- | EF615762 | EF616053 | AY625166 | EF616342 | -- | -- | -- | EF616414 | -- |
| *Microlophus heterolepis* | EF615638 | EF615710 | -- | EF615783 | EF616074 | -- | EF616362 | -- | -- | -- | EF616435 | -- |
| *Microlophus koepckeorum* | EF615630 | EF615701 | -- | EF615774 | EF616065 | AY625159 | EF616353 | -- | -- | -- | EF616426 | -- |
| *Microlophus occipitalis* | EF615634 | EF615707 | -- | EF615780 | EF616071 | AY625162 | EF616359 | -- | -- | -- | EF616432 | -- |
| *Microlophus pacificus* | FJ458491 | EF615700 | -- | EF615773 | EF616064 | AY625172 | EF616352 | -- | -- | -- | EF616425 | -- |
| *Microlophus peruvianus* | EF615639 | EF615718 | -- | EF615791 | EF616081 | AY625158 | EF616370 | -- | -- | -- | EF616443 | -- |
| *Microlophus quadrivittatus* | EF615601 | EF615716 | -- | EF615789 | EF616080 | -- | EF616368 | -- | -- | -- | EF616441 | -- |
| *Microlophus stolzmanni* | AF362518 | EF615724 | -- | EF615797 | EF616088 | AY625161 | EF616376 | -- | -- | -- | EF616449 | -- |
| *Microlophus theresiae* | EF615652 | EF615727 | -- | EF615799 | EF616091 | AY625155 | EF616379 | -- | -- | -- | EF616452 | -- |
| *Microlophus theresioides* | EF615605 | EF615675 | -- | EF615748 | EF616039 | -- | EF616331 | -- | -- | -- | EF616400 | -- |
| *Microlophus thoracicus* | EF615660 | EF615736 | -- | EF615808 | EF616098 | -- | EF616388 | -- | -- | -- | EF616461 | -- |
| *Microlophus tigris* | EF615648 | EF615723 | -- | EF615796 | EF616087 | AY625156 | EF616375 | -- | -- | -- | EF616448 | -- |
| *Microlophus yanezi* | EF615608 | EF615677 | -- | EF615750 | EF616041 | -- | EF616333 | -- | -- | -- | EF616402 | -- |
| *Micropechis ikaheka* | EU547091 | EU547140 | -- | -- | AF217826 | -- | EU547000 | -- | -- | -- | EU366435 | -- |
| *Micropisthodon ochraceus* | -- | AF215271 | -- | AY187991 | AY188030 | -- | -- | -- | -- | -- | -- | -- |
| *Micruroides euryxanthus* | Z46433 | Z46483 | -- | EF137423 | AF217823 | -- | EF137408 | -- | -- | -- | -- | -- |
| *Micrurus albicinctus* | -- | -- | -- | -- | -- | -- | JF308714 | -- | -- | -- | -- | -- |
| *Micrurus altirostris* | -- | JQ627286 | -- | -- | -- | -- | AF228432 | -- | -- | -- | -- | -- |
| *Micrurus baliocoryphus* | -- | -- | -- | -- | -- | -- | AF228433 | -- | -- | -- | -- | -- |
| *Micrurus brasiliensis* | -- | -- | -- | -- | -- | -- | AF228428 | -- | -- | -- | -- | -- |
| *Micrurus corallinus* | -- | -- | -- | -- | -- | -- | AF228424 | -- | -- | -- | -- | -- |
| *Micrurus decoratus* | -- | -- | -- | -- | -- | -- | AF228441 | -- | -- | -- | -- | -- |
| *Micrurus diastema* | Z46454 | Z46484 | -- | -- | -- | -- | JF308710 | -- | -- | -- | -- | -- |
| *Micrurus dissoleucus* | -- | -- | -- | -- | -- | -- | JF308717 | -- | -- | -- | -- | -- |
| *Micrurus frontalis* | -- | -- | -- | -- | -- | -- | AF228426 | -- | -- | -- | -- | -- |
| *Micrurus fulvius* | NC_013481 | NC_13481 | EU402653 | AY058935 | AF217839 | -- | U49298 | EU390929 | -- | HQ876363 | EU402856 | -- |
| *Micrurus hemprichii* | -- | -- | -- | -- | -- | -- | AF228442 | -- | -- | -- | -- | -- |
| *Micrurus ibiboboca* | -- | -- | -- | -- | -- | -- | AF228440 | -- | -- | -- | -- | -- |
| *Micrurus lemniscatus* | -- | -- | -- | -- | -- | -- | AF228439 | -- | -- | -- | -- | -- |
| *Micrurus mipartitus* | -- | -- | -- | -- | EF137414 | -- | EF137406 | -- | -- | -- | -- | -- |
| *Micrurus narduccii* | -- | -- | -- | EF137420 | EF137412 | -- | EF137404 | -- | -- | -- | -- | -- |
| *Micrurus psyches* | -- | -- | -- | -- | -- | -- | JF308713 | -- | -- | -- | -- | -- |
| *Micrurus pyrrhocryptus* | -- | -- | -- | -- | -- | -- | AF228434 | -- | -- | -- | -- | -- |
| *Micrurus spixii* | -- | -- | -- | -- | -- | -- | AF228443 | -- | -- | -- | -- | -- |
| *Micrurus surinamensis* | AF544770 | AF544799 | FJ433991 | AF544708 | EF137415 | -- | EF137407 | FJ434092 | -- | EF144075 | AY487411 | EF144102 |
| *Mimophis mahfalensis* | AF544771 | AF215266 | JQ073081 | AY187993 | AY188032 | -- | DQ486276 | -- | -- | -- | JQ073202 | FJ404391 |
| *Mochlus brevicaudis* | -- | -- | HM160590 | -- | -- | -- | -- | -- | -- | HM161064 | HM161159 | -- |
| *Mochlus sundevalli* | AY308427 | AY308279 | -- | -- | -- | -- | -- | -- | -- | -- | -- | -- |
| *Moloch horridus* | -- | -- | DQ340746 | DQ340697 | -- | AF128467 | -- | JF804555 | -- | JF804595 | JF806197 | -- |
| *Monopeltis capensis* | -- | -- | -- | AY444009 | -- | -- | -- | -- | -- | -- | AY444036 | -- |
| *Morelia amethistina* | EF545018 | EF545045 | -- | -- | AF241387 | -- | -- | -- | -- | -- | -- | -- |
| *Morelia boeleni* | AF544750 | EF545047 | -- | AF544723 | EF545097 | -- | -- | -- | -- | -- | -- | -- |
| *Morelia bredli* | EF545016 | EF545043 | -- | -- | EF545094 | -- | -- | -- | -- | -- | -- | -- |
| *Morelia carinata* | EF545017 | EF545044 | -- | -- | EF545095 | -- | -- | -- | -- | -- | -- | -- |
| *Morelia oenpelliensis* | EF545019 | EF545046 | -- | -- | EF545096 | -- | -- | -- | -- | -- | -- | -- |
| *Morelia spilota* | EF545015 | EF545042 | -- | -- | U69851 | -- | -- | -- | -- | -- | -- | -- |
| *Morelia viridis* | EF545022 | EF545049 | -- | -- | EF545098 | -- | -- | -- | -- | -- | -- | -- |
| *Morethia adelaidensis* | EU567930 | EU567926 | -- | DQ675368 | EU567830 | EU567728 | EU567767 | -- | -- | -- | EU568109 | -- |
| *Morethia butleri* | AY169572 | AY169609 | -- | -- | -- | -- | AY169647 | -- | -- | -- | -- | -- |
| *Morethia ruficauda* | -- | -- | HQ655178 | HQ655213 | -- | -- | -- | -- | -- | -- | -- | -- |
| *Morunasaurus annularis* | -- | -- | EU586768 | -- | -- | EU586758 | -- | JF804556 | -- | HQ876328 | FJ356741 | -- |
| *Myron richardsonii* | AF499291 | EF395869 | -- | EF395941 | EF395918 | -- | -- | -- | -- | -- | -- | -- |
| *Nactus acutus* | -- | -- | -- | -- | -- | EU054289 | -- | -- | EU054257 | -- | EU054273 | -- |
| *Nactus cheverti* | -- | -- | -- | -- | -- | HM997154 | -- | -- | HM997178 | -- | HM997166 | -- |
| *Nactus eboracensis* | -- | -- | -- | -- | -- | HM997156 | -- | -- | HM997180 | -- | HM997168 | -- |
| *Nactus galgajuga* | -- | -- | -- | -- | -- | HM997158 | -- | -- | HM997182 | -- | HM997170 | -- |
| *Nactus multicarinatus* | -- | -- | -- | -- | -- | HM997160 | -- | -- | HM997184 | -- | HM997172 | -- |
| *Nactus pelagicus* | DQ852706 | -- | -- | AY172936 | -- | HM997163 | -- | -- | HM997187 | -- | EU054275 | JQ945484 |
| *Nactus vankampeni* | -- | -- | -- | JQ945591 | -- | EU054296 | -- | -- | EU054264 | -- | EU054280 | JQ945485 |
| *Nadzikambia mlanjense* | -- | -- | -- | -- | -- | AY289918 | -- | -- | -- | -- | DQ996681 | -- |
| *Naja annulata* | -- | AY188049 | -- | AY187971 | AY188010 | AY058998 | AY058970 | -- | -- | -- | -- | -- |
| *Naja annulifera* | GQ359667 | GQ359753 | -- | -- | AF155216 | -- | GQ359586 | -- | -- | -- | -- | -- |
| *Naja ashei* | GQ359656 | GQ359742 | -- | -- | -- | -- | -- | -- | -- | -- | -- | -- |
| *Naja atra* | EU913475 | HM439991 | -- | -- | EF206656 | NC_011389 | NC 011389 | -- | -- | -- | -- | -- |
| *Naja haje* | GQ359663 | GQ359752 | -- | -- | -- | -- | -- | -- | -- | -- | -- | -- |
| *Naja kaouthia* | EU624235 | JF357948 | EU402654 | AY058938 | AF217835 | -- | EU624209 | EU390930 | -- | JN703083 | EU402857 | -- |
| *Naja katiensis* | GQ359657 | GQ359743 | -- | -- | -- | -- | -- | -- | -- | -- | -- | -- |
| *Naja mandalayensis* | -- | -- | -- | -- | AF155211 | -- | -- | -- | -- | -- | -- | -- |
| *Naja melanoleuca* | U96801 | JF357949 | -- | -- | -- | -- | JF357931 | -- | -- | -- | -- | -- |
| *Naja mossambica* | GQ359658 | -- | -- | -- | AF399747 | -- | DQ897725 | -- | -- | -- | -- | -- |
| *Naja multifasciata* | -- | -- | -- | AY058941 | AF217837 | -- | -- | -- | -- | -- | -- | -- |
| *Naja naja* | NC_010225 | GQ359756 | -- | AF435020 | DQ272477 | DQ343648 | DQ897690 | -- | -- | -- | EU366432 | -- |
| *Naja nigricollis* | EU624237 | GQ359754 | -- | -- | AF399746 | -- | DQ897697 | -- | -- | -- | -- | -- |
| *Naja nivea* | EU624238 | GQ359755 | -- | AY058939 | AF217827 | -- | -- | -- | -- | -- | -- | -- |
| *Naja nubiae* | GQ359660 | GQ359746 | -- | -- | AF399752 | -- | DQ897718 | -- | -- | -- | -- | -- |
| *Naja pallida* | GQ359659 | GQ359745 | -- | -- | AF399750 | -- | DQ897714 | -- | -- | -- | -- | -- |
| *Naja siamensis* | JN687926 | JN687927 | -- | -- | AF096295 | -- | -- | -- | -- | -- | -- | -- |
| *Naja sumatrana* | JN687928 | JN687929 | -- | -- | -- | -- | -- | -- | -- | -- | -- | -- |
| *Nangura spinosa* | -- | AY169630 | HQ655179 | HQ655214 | -- | -- | AY169668 | -- | -- | -- | -- | -- |
| *Nannoscincus garrulus* | -- | -- | -- | -- | -- | DQ675262 | -- | -- | -- | -- | DQ675342 | -- |
| *Nannoscincus gracilis* | -- | -- | -- | DQ675373 | -- | DQ675233 | -- | -- | -- | -- | DQ675313 | -- |
| *Nannoscincus greeri* | -- | -- | -- | DQ675370 | -- | DQ675230 | -- | -- | -- | -- | DQ675310 | -- |
| *Nannoscincus hanchisteus* | -- | -- | -- | -- | -- | DQ675270 | -- | -- | -- | -- | -- | -- |
| *Nannoscincus humectus* | -- | -- | -- | -- | -- | DQ675269 | -- | -- | -- | -- | -- | -- |
| *Nannoscincus mariei* | -- | EU567924 | -- | DQ675372 | EU567832 | EU423132 | EU423130 | -- | -- | -- | EU568021 | -- |
| *Nannoscincus slevini* | -- | -- | -- | -- | -- | DQ675256 | -- | -- | -- | -- | DQ675336 | -- |
| *Narudasia festiva* | DQ852707 | -- | -- | EF534934 | -- | JX041387 | -- | -- | EF534850 | -- | EF534808 | EF534976 |
| *Natriciteres olivacea* | AF544772 | AF544801 | -- | AF471146 | AF471058 | -- | -- | -- | -- | -- | -- | -- |
| *Natrix maura* | AF402623 | -- | -- | -- | AY866530 | AY487770 | AY487785 | -- | -- | JF946392 | -- | -- |
| *Natrix natrix* | AF158461 | AF158530 | EU402655 | AF471121 | AY487756 | AY870625 | AY487792 | EU390931 | -- | JF946390 | EU402858 | -- |
| *Natrix tessellata* | -- | -- | -- | -- | AY866534 | AY870642 | AY873734 | -- | -- | JF946388 | -- | -- |
| *Naultinus elegans* | -- | GU459955 | -- | -- | -- | GU459757 | -- | -- | GU459556 | -- | GU459354 | -- |
| *Naultinus gemmeus* | -- | GU459962 | -- | JQ945592 | -- | GU459764 | -- | -- | GU459563 | -- | GU459361 | JQ945486 |
| *Naultinus grayii* | -- | GU459965 | -- | -- | -- | GU459767 | -- | -- | GU459566 | -- | GU459364 | -- |
| *Naultinus manukanus* | -- | GU459968 | -- | -- | -- | GU459770 | -- | -- | GU459569 | -- | GU459367 | -- |
| *Naultinus poecilochlorus* | -- | GU459969 | -- | -- | -- | GU459771 | -- | -- | GU459570 | -- | GU459368 | -- |
| *Naultinus rudis* | -- | GU459970 | -- | -- | -- | GU459772 | -- | -- | GU459571 | -- | GU459369 | -- |
| *Naultinus stellatus* | -- | GU459974 | -- | -- | -- | GU459776 | -- | -- | GU459575 | -- | GU459373 | -- |
| *Naultinus tuberculatus* | -- | GU459977 | -- | -- | -- | GU459779 | -- | -- | GU459578 | -- | GU459376 | -- |
| *Nephrurus amyae* | -- | -- | -- | JF807319 | -- | JF807368 | -- | -- | -- | -- | JF807385 | -- |
| *Nephrurus asper* | -- | -- | -- | -- | -- | JF807363 | -- | -- | -- | -- | -- | -- |
| *Nephrurus deleani* | -- | -- | -- | JF807322 | -- | JF807345 | -- | -- | -- | -- | JF807388 | -- |
| *Nephrurus laevissimus* | -- | -- | -- | JF807323 | -- | AY369020 | -- | -- | -- | -- | JF807389 | -- |
| *Nephrurus levis* | -- | GU460144 | -- | JF807326 | -- | AY369018 | -- | -- | GU459746 | -- | GU459544 | JQ945487 |
| *Nephrurus sheai* | -- | -- | -- | JF807320 | -- | JF807364 | -- | -- | -- | -- | JF807386 | -- |
| *Nephrurus stellatus* | -- | -- | -- | -- | -- | JF807340 | -- | -- | -- | -- | -- | -- |
| *Nephrurus vertebralis* | -- | -- | -- | JF807321 | -- | AY369019 | -- | -- | -- | -- | JF807387 | -- |
| *Nephrurus wheeleri* | -- | -- | -- | JF807317 | -- | AY369021 | -- | -- | -- | -- | JF807384 | -- |
| *Nerodia cyclopion* | AF402626 | -- | -- | -- | AF402909 | AF384828 | -- | -- | -- | -- | -- | -- |
| *Nerodia erythrogaster* | AF402629 | -- | -- | JN090137 | L33336 | AF384831 | -- | -- | -- | -- | -- | -- |
| *Nerodia fasciata* | AF402627 | -- | -- | -- | AY866529 | AY870612 | AY873705 | -- | -- | JF946384 | -- | -- |
| *Nerodia floridana* | AF402628 | -- | -- | -- | AF402911 | AF384830 | -- | -- | -- | -- | -- | -- |
| *Nerodia harteri* | AF402652 | -- | -- | -- | AF402935 | AF384854 | -- | -- | -- | -- | -- | -- |
| *Nerodia rhombifer* | Z46452 | Z46481 | -- | -- | AF402915 | AF384834 | -- | -- | -- | -- | -- | -- |
| *Nerodia sipedon* | AF402630 | -- | -- | -- | AF402913 | DQ915161 | NC_015793 | -- | -- | -- | -- | -- |
| *Nerodia taxispilota* | AF402631 | -- | -- | -- | AF402914 | AF384833 | U49322 | -- | -- | -- | -- | -- |
| *Neusticurus bicarinatus* | -- | AF420708 | -- | -- | -- | -- | -- | -- | -- | -- | -- | -- |
| *Neusticurus rudis* | -- | AF420709 | -- | -- | -- | -- | AF420905 | -- | -- | -- | -- | -- |
| *Ninia atrata* | GQ457814 | JQ598882 | JQ599037 | GQ457874 | GQ334553 | -- | GQ334659 | GQ334683 | -- | -- | -- | -- |
| *Niveoscincus greeni* | -- | -- | HQ655180 | HQ655215 | -- | -- | -- | -- | -- | -- | -- | -- |
| *Niveoscincus metallicus* | AY308434 | AY308282 | -- | -- | -- | -- | -- | -- | -- | -- | -- | -- |
| *Niveoscincus ocellatus* | AY818751 | -- | -- | AY818783 | AY818821 | -- | -- | -- | -- | -- | -- | -- |
| *Niveoscincus pretiosus* | EU568019 | EU567927 | -- | AY818782 | EU567834 | EU567726 | EU567768 | -- | -- | -- | EU568110 | -- |
| *Notechis scutatus* | U96802 | -- | EU402656 | EU546944 | AF217836 | -- | EU547034 | EU390932 | -- | JN703081 | EU402859 | -- |
| *Nothobachia ablephara* | -- | AF420740 | -- | AF420851 | -- | -- | AF420900 | -- | -- | -- | -- | -- |
| *Nothopsis rugosus* | GU018159 | GU018177 | -- | -- | -- | -- | -- | -- | -- | -- | -- | -- |
| *Notoscincus ornatus* | AY169594 | AY169631 | HQ655181 | HQ655216 | -- | -- | AY169669 | -- | -- | -- | -- | -- |
| *Nucras lalandii* | -- | -- | -- | EF632276 | -- | -- | -- | -- | -- | -- | EF632233 | -- |
| *Nucras tessellata* | -- | DQ871143 | -- | -- | AF206550 | DQ871085 | -- | -- | -- | -- | DQ871201 | -- |
| *Oedodera marmorata* | -- | GU460146 | -- | JQ945594 | -- | GU459947 | JQ398455 | -- | GU459748 | -- | GU459546 | JQ945488 |
| *Oedura castelnaui* | -- | -- | -- | -- | -- | JQ173634 | JQ398457 | -- | JQ173680 | -- | JQ173728 | -- |
| *Oedura coggeri* | -- | -- | -- | -- | -- | JQ173635 | JQ398458 | -- | JQ173681 | -- | JQ173729 | -- |
| *Oedura filicipoda* | -- | -- | -- | -- | -- | JQ173636 | JQ398459 | -- | JQ173682 | -- | JQ173730 | -- |
| *Oedura gemmata* | -- | -- | -- | -- | -- | JQ173637 | JQ398460 | -- | JQ173683 | -- | JQ173731 | -- |
| *Oedura gracilis* | -- | -- | -- | -- | -- | JQ173638 | JQ398461 | -- | JQ173684 | -- | JQ173732 | -- |
| *Oedura lesueurii* | -- | -- | -- | -- | -- | JQ173642 | JQ398463 | -- | JQ173688 | -- | JQ173736 | -- |
| *Oedura marmorata* | -- | GU460150 | -- | EF534903 | -- | AY369015 | JQ398471 | -- | GU459752 | -- | FJ571623 | EF534945 |
| *Oedura monilis* | -- | -- | -- | -- | -- | JQ173652 | JQ398473 | -- | JQ173698 | -- | JQ173746 | -- |
| *Oedura obscura* | -- | -- | -- | -- | -- | JQ173656 | JQ398477 | -- | JQ173702 | -- | JQ173748 | -- |
| *Oedura reticulata* | -- | -- | -- | FJ855471 | -- | EF681803 | JQ398478 | -- | JQ173703 | -- | FJ855450 | -- |
| *Oedura rhombifer* | -- | -- | -- | JQ945595 | -- | JQ173660 | JQ398485 | -- | JQ173708 | -- | JQ173754 | JQ945489 |
| *Oedura robusta* | -- | -- | -- | -- | -- | JQ173662 | JQ398486 | -- | JQ173710 | -- | JQ173756 | -- |
| *Oedura tryoni* | AF090179 | AY583929 | -- | AF090848 | AY583956 | EF532898 | JQ398487 | -- | JQ173712 | -- | JQ173758 | -- |
| *Oligodon arnensis* | KC347327 | KC347365 | -- | KC347405 | KC347481 | -- | KC347518 | -- | -- | -- | KC347442 | -- |
| *Oligodon barroni* | HM591523 | HM591523 | -- | -- | -- | -- | -- | -- | -- | -- | -- | -- |
| *Oligodon chinensis* | HM591524 | HM591527 | -- | -- | -- | -- | -- | -- | -- | -- | -- | -- |
| *Oligodon cinereus* | HM591503 | HM591508 | -- | AF471101 | AF471033 | -- | -- | -- | -- | -- | -- | -- |
| *Oligodon cruentatus* | HM591517 | HM591517 | -- | -- | -- | -- | -- | -- | -- | -- | -- | -- |
| *Oligodon cyclurus* | HM591535 | HM591536 | -- | -- | -- | -- | -- | -- | -- | -- | -- | -- |
| *Oligodon formosanus* | HM591532 | HM591533 | -- | -- | -- | -- | -- | -- | -- | -- | -- | -- |
| *Oligodon maculatus* | HM591511 | HM591511 | -- | -- | -- | -- | -- | -- | -- | -- | -- | -- |
| *Oligodon ocellatus* | HM591534 | HM591534 | -- | -- | -- | -- | -- | -- | -- | -- | -- | -- |
| *Oligodon octolineatus* | HM591519 | HM591519 | -- | -- | -- | -- | -- | -- | -- | -- | -- | -- |
| *Oligodon planiceps* | HM591514 | HM591514 | -- | -- | -- | -- | -- | -- | -- | -- | -- | -- |
| *Oligodon splendidus* | HM591510 | HM591510 | -- | -- | -- | -- | -- | -- | -- | -- | -- | -- |
| *Oligodon sublineatus* | KC347329 | KC347367 | -- | KC347407 | KC347483 | -- | KC347520 | -- | -- | -- | KC347444 | -- |
| *Oligodon taeniatus* | HM591520 | HM591522 | -- | -- | -- | -- | -- | -- | -- | -- | -- | -- |
| *Oligodon taeniolatus* | KC347330 | KC347368 | -- | KC347408 | KC347484 | -- | KC347521 | -- | -- | -- | KC347445 | -- |
| *Oligodon theobaldi* | HM591516 | HM591516 | -- | -- | -- | -- | -- | -- | -- | -- | -- | -- |
| *Oligodon torquatus* | HM591513 | HM591513 | -- | -- | -- | -- | -- | -- | -- | -- | -- | -- |
| *Oligosoma acrinasum* | EU567994 | EU567905 | -- | -- | EU567811 | EF033047 | EF033061 | -- | -- | -- | EU568099 | -- |
| *Oligosoma chloronoton* | EU567964 | EU567901 | -- | -- | EU837108 | EF103969 | EF104010 | -- | -- | -- | EU568053 | -- |
| *Oligosoma fallai* | EU567988 | EU567848 | -- | -- | EU567782 | EU567723 | EU567733 | -- | -- | -- | EU568062 | -- |
| *Oligosoma grande* | EU567935 | EU567881 | -- | -- | EU567808 | EU567721 | EU567757 | -- | -- | -- | EU568031 | -- |
| *Oligosoma homalonotum* | EU568010 | EU567840 | -- | -- | EU567819 | EU567724 | EU567748 | -- | -- | -- | EU568086 | -- |
| *Oligosoma inconspicuum* | EU567940 | EU567879 | -- | -- | -- | EU567709 | EU567738 | -- | -- | -- | -- | -- |
| *Oligosoma infrapunctatum* | EU568006 | EU567917 | -- | -- | EF175864 | EF033051 | EF033059 | -- | -- | -- | EU568104 | -- |
| *Oligosoma lineoocellatum* | EU567958 | EU567903 | -- | -- | EU837105 | EF103994 | EF447112 | -- | -- | -- | EU568054 | -- |
| *Oligosoma longipes* | EU567962 | EU567899 | -- | -- | EU567805 | EU567717 | EU567755 | -- | -- | -- | EU568043 | -- |
| *Oligosoma maccanni* | EU567947 | AY308284 | -- | -- | EU567794 | EF447145 | EF447178 | -- | -- | -- | EU568035 | -- |
| *Oligosoma microlepis* | EU568015 | EU567850 | -- | DQ675375 | EU567825 | EF043107 | EU567760 | -- | -- | -- | EU568088 | -- |
| *Oligosoma moco* | EU567990 | EU567921 | -- | -- | EU567784 | EF567303 | EU567765 | -- | -- | -- | EU568072 | -- |
| *Oligosoma nigriplantare* | EU567990 | EU567891 | -- | -- | EU567802 | EF043155 | EF043224 | -- | -- | -- | EU568030 | -- |
| *Oligosoma notosaurus* | EU567944 | EU567877 | -- | -- | EU567791 | EU567711 | EU567741 | -- | -- | -- | EU568050 | -- |
| *Oligosoma otagense* | EU568000 | EU567909 | -- | -- | EU567814 | EF033054 | EF033065 | -- | -- | -- | EU568096 | -- |
| *Oligosoma pikitanga* | -- | -- | -- | -- | -- | EU885755 | EU885758 | -- | -- | -- | -- | -- |
| *Oligosoma smithi* | EU568018 | EU567852 | -- | DQ675386 | EU567827 | EF033055 | EU567761 | -- | -- | -- | EU568090 | -- |
| *Oligosoma stenotis* | EU567936 | EU567887 | -- | -- | EU567810 | EU567719 | EU567759 | -- | -- | -- | EU568047 | -- |
| *Oligosoma striatum* | EU568011 | EU567868 | -- | -- | EU567821 | EU567725 | EU567749 | -- | -- | -- | EU568056 | -- |
| *Oligosoma suteri* | EU567968 | EU567838 | -- | DQ675387 | EU567773 | EF567282 | EU567751 | -- | -- | -- | EU568106 | -- |
| *Oligosoma taumakae* | EU567996 | EU567907 | -- | -- | EU567813 | EF033049 | EF033063 | -- | -- | -- | EU568092 | -- |
| *Oligosoma waimatense* | EU567999 | EU567918 | -- | -- | EU567816 | EU567712 | EU567742 | -- | -- | -- | EU568095 | -- |
| *Oligosoma zelandicum* | EU568014 | EU567842 | -- | AY818781 | EU567823 | EF033057 | EF447216 | -- | -- | -- | EU568082 | -- |
| *Omanosaura cyanura* | -- | AF080349 | -- | -- | AF080348 | -- | -- | -- | -- | -- | -- | -- |
| *Omanosaura jayakari* | -- | AF080352 | -- | EF632277 | AF080351 | -- | -- | -- | -- | -- | EF632234 | -- |
| *Opheodrys aestivus* | -- | -- | -- | AF471147 | AF471057 | -- | -- | -- | -- | -- | -- | -- |
| *Opheodrys vernalis* | -- | -- | -- | GQ927317 | GQ927322 | -- | -- | -- | -- | -- | -- | -- |
| *Ophidiocephalus taeniatus* | -- | AY134529 | -- | FJ571645 | -- | AY134601 | -- | -- | HQ426214 | -- | FJ571630 | HQ426476 |
| *Ophiodes striatus* | -- | -- | -- | -- | -- | AF085610 | -- | -- | -- | -- | -- | -- |
| *Ophiomorus latastii* | EF581272 | -- | -- | -- | EF581259 | -- | -- | -- | -- | -- | -- | -- |
| *Ophiomorus punctatissimus* | AY649127 | AY649168 | HM160594 | -- | EF581258 | AY607279 | -- | -- | -- | HM161068 | HM161163 | -- |
| *Ophiophagus hannah* | EU921899 | NC_11394 | -- | AY058940 | EF694840 | NC_011394 | NC 011394 | -- | -- | -- | -- | -- |
| *Ophioscincus ophioscincus* | AY169595 | AY169632 | -- | -- | -- | -- | AY169670 | -- | -- | -- | -- | -- |
| *Ophioscincus truncatus* | DQ915306 | DQ915330 | -- | -- | -- | -- | DQ915354 | -- | -- | -- | -- | -- |
| *Ophisaurus attenuatus* | EU747729 | EU747729 | -- | -- | EU747729 | EU747729 | EU747729 | -- | -- | -- | AY662602 | -- |
| *Ophisaurus gracilis* | AF380953 | -- | -- | AY444030 | AF380964 | -- | -- | -- | -- | -- | AY444056 | -- |
| *Ophisaurus harti* | AF526565 | -- | -- | -- | AF380962 | AF085624 | -- | -- | -- | -- | -- | -- |
| *Ophisaurus koellikeri* | -- | -- | -- | -- | -- | AF085621 | -- | -- | -- | -- | -- | -- |
| *Ophisaurus ventralis* | JN393208 | -- | -- | -- | -- | AF085626 | -- | -- | -- | -- | -- | -- |
| *Ophisops elegans* | -- | GQ142092 | -- | EF632278 | GQ142116 | -- | -- | -- | -- | -- | EF632235 | -- |
| *Ophisops occidentalis* | -- | EU081755 | -- | -- | EU081680 | -- | -- | -- | -- | -- | -- | -- |
| *Ophryacus melanurus* | AF057210 | AF057257 | -- | -- | AY223587 | -- | AY223634 | -- | -- | -- | -- | -- |
| *Ophryacus undulatus* | AF057209 | AF057256 | -- | -- | AY223586 | -- | AY223633 | -- | -- | -- | -- | -- |
| *Opisthotropis cheni* | -- | -- | -- | JQ687441 | GQ281779 | JQ687457 | JQ687416 | -- | -- | -- | -- | -- |
| *Opisthotropis guangxiensis* | -- | -- | -- | JQ687447 | GQ281776 | JQ687462 | JQ687422 | -- | -- | -- | -- | -- |
| *Opisthotropis lateralis* | -- | -- | -- | JQ687445 | GQ281782 | JQ687461 | JQ687420 | -- | -- | -- | -- | -- |
| *Opisthotropis latouchii* | -- | -- | -- | JQ687446 | GQ281783 | -- | JQ687421 | -- | -- | -- | -- | -- |
| *Oplurus cuvieri* | U39587 | AF215260 | AY987971 | EU099677 | -- | U82685 | U66225 | -- | -- | -- | AY662601 | -- |
| *Oplurus cyclurus* | U39585 | EU099750 | GU457850 | EU099680 | -- | -- | -- | GU456006 | -- | HQ876332 | GU457973 | -- |
| *Oplurus fierinensis* | -- | EU099754 | -- | EU099702 | -- | -- | -- | -- | -- | -- | -- | -- |
| *Oplurus grandidieri* | AB218720 | EU099755 | -- | EU099703 | NC_012827 | AB218720 | AB218720 | -- | -- | -- | -- | -- |
| *Oplurus quadrimaculatus* | -- | EU099752 | -- | EU099686 | AF020259 | -- | -- | -- | -- | -- | -- | -- |
| *Oplurus saxicola* | U39566 | EU099746 | -- | EU099693 | -- | -- | -- | -- | -- | -- | -- | -- |
| *Oreocryptophis porphyracea* | -- | -- | -- | DQ902076 | DQ902118 | NC_012770 | DQ902298 | -- | -- | -- | -- | -- |
| *Orraya occultus* | -- | -- | -- | -- | -- | JX041389 | -- | -- | JQ945388 | -- | JQ945320 | -- |
| *Orthriophis cantoris* | AY122769 | -- | -- | DQ902095 | DQ902135 | DQ902246 | DQ902315 | -- | -- | -- | -- | -- |
| *Orthriophis hodgsoni* | -- | -- | -- | DQ902096 | DQ902136 | DQ902247 | DQ902318 | -- | -- | -- | -- | -- |
| *Orthriophis moellendorffi* | AY122786 | -- | -- | DQ902074 | DQ902116 | DQ902223 | DQ902295 | -- | -- | -- | -- | -- |
| *Orthriophis taeniurus* | AY122807 | HM439981 | -- | EF076705 | EF076709 | EF076707 | EF076708 | -- | -- | -- | -- | -- |
| *Otocryptis wiegmanni* | -- | -- | -- | -- | -- | AF128480 | -- | -- | -- | -- | -- | -- |
| *Ovophis monticola* | HQ325275 | HQ325078 | -- | -- | AY763230 | -- | AY352809 | -- | -- | -- | -- | -- |
| *Ovophis okinavensis* | AB175670 | AB175670 | -- | -- | DQ305464 | AB175670 | AY352824 | -- | -- | -- | -- | -- |
| *Ovophis tonkinensis* | HQ325282 | HQ325081 | -- | -- | HQ325140 | -- | HQ325243 | -- | -- | -- | -- | -- |
| *Ovophis zayuensis* | HQ325304 | HQ325118 | -- | -- | HQ325150 | -- | HQ325239 | -- | -- | -- | -- | -- |
| *Oxybelis aeneus* | AF158416 | AF158498 | -- | AF471148 | AF471056 | -- | -- | -- | -- | -- | -- | -- |
| *Oxybelis fulgidus* | AF158432 | AF158497 | -- | -- | -- | -- | -- | -- | -- | -- | -- | -- |
| *Oxyrhabdium leporinum* | -- | -- | -- | DQ112081 | AF471029 | -- | -- | -- | -- | -- | -- | FJ404466 |
| *Oxyrhopus clathratus* | GQ457815 | GQ457754 | -- | GQ457875 | -- | -- | -- | -- | -- | -- | -- | -- |
| *Oxyrhopus formosus* | JQ598821 | AF158482 | -- | -- | -- | -- | -- | -- | -- | -- | -- | -- |
| *Oxyrhopus guibei* | JQ598822 | JQ598883 | JQ599038 | JQ598989 | AF236813 | -- | -- | -- | -- | -- | -- | -- |
| *Oxyrhopus melanogenys* | JQ598823 | AF158489 | -- | JQ598990 | -- | -- | -- | -- | -- | -- | -- | -- |
| *Oxyrhopus petola* | GU018144 | GU018170 | -- | -- | GQ334554 | -- | GQ334660 | GQ334684 | -- | -- | -- | -- |
| *Oxyrhopus rhombifer* | GU018146 | GU018165 | -- | -- | -- | -- | -- | -- | -- | -- | -- | -- |
| *Oxyrhopus trigeminus* | JQ598824 | JQ598884 | -- | -- | JQ598939 | -- | -- | -- | -- | -- | -- | -- |
| *Oxyuranus microlepidotus* | EU547099 | EU547148 | -- | -- | -- | -- | EF210823 | -- | -- | -- | EU366439 | -- |
| *Oxyuranus scutellatus* | EU547100 | EU547149 | -- | -- | DQ897772 | -- | AY340788 | -- | -- | -- | EU546877 | -- |
| *Pachydactylus affinis* | DQ275389 | AY123392 | -- | -- | AY123414 | -- | -- | -- | -- | -- | DQ275433 | -- |
| *Pachydactylus austeni* | DQ275371 | AF449110 | -- | JQ945596 | AF449126 | JX041390 | -- | -- | JQ945389 | -- | DQ275415 | -- |
| *Pachydactylus barnardi* | DQ275378 | AY123388 | -- | -- | AY123410 | -- | -- | -- | -- | -- | DQ275422 | -- |
| *Pachydactylus bicolor* | DQ275394 | AY123378 | -- | -- | AY123400 | -- | -- | -- | -- | -- | DQ275438 | -- |
| *Pachydactylus capensis* | DQ275387 | AF449117 | -- | -- | AF449133 | HQ165962 | -- | -- | HQ165977 | -- | DQ275431 | -- |
| *Pachydactylus caraculicus* | DQ275393 | AY123377 | -- | -- | AY123399 | -- | -- | -- | -- | -- | DQ275437 | -- |
| *Pachydactylus carinatus* | -- | -- | -- | -- | DQ349165 | -- | -- | -- | -- | -- | -- | -- |
| *Pachydactylus fasciatus* | DQ275384 | AF449112 | -- | -- | AF449128 | HQ165949 | -- | -- | HQ165964 | -- | DQ275428 | -- |
| *Pachydactylus formosus* | DQ275377 | AY123389 | -- | -- | AY123411 | -- | -- | -- | -- | -- | DQ275421 | -- |
| *Pachydactylus gaiasensis* | DQ275399 | AY123385 | -- | JQ945597 | AY123407 | JX041391 | -- | -- | JQ945390 | -- | DQ275443 | JQ945490 |
| *Pachydactylus geitje* | DQ275380 | AF449116 | -- | -- | AF449132 | -- | -- | -- | -- | -- | DQ275424 | -- |
| *Pachydactylus griffini* | -- | -- | -- | -- | DQ349166 | -- | -- | -- | -- | -- | -- | -- |
| *Pachydactylus haackei* | DQ275402 | AF449105 | -- | -- | AF449121 | -- | -- | -- | -- | -- | DQ275446 | -- |
| *Pachydactylus kladaroderma* | DQ275401 | AF449106 | -- | JQ945598 | AF449122 | JX041392 | -- | -- | JQ945391 | -- | DQ275445 | JQ945491 |
| *Pachydactylus labialis* | DQ275379 | AF449114 | -- | -- | AF449130 | -- | -- | -- | -- | -- | DQ275423 | -- |
| *Pachydactylus laevigatus* | AF186118 | -- | -- | -- | -- | -- | -- | -- | -- | -- | -- | -- |
| *Pachydactylus maculatus* | DQ275382 | AF449111 | -- | -- | AF449127 | -- | -- | -- | -- | -- | DQ275426 | -- |
| *Pachydactylus mariquensis* | DQ275370 | DQ278599 | -- | -- | DQ278601 | -- | -- | -- | -- | -- | DQ275414 | -- |
| *Pachydactylus mclachlani* | -- | -- | -- | -- | -- | HQ165951 | -- | -- | HQ165966 | -- | HQ165981 | -- |
| *Pachydactylus monicae* | -- | -- | -- | -- | DQ349180 | HQ165953 | -- | -- | HQ165968 | -- | HQ165983 | -- |
| *Pachydactylus montanus* | -- | -- | -- | -- | DQ349157 | -- | -- | -- | -- | -- | -- | -- |
| *Pachydactylus namaquensis* | DQ275400 | AF449107 | -- | -- | AF449123 | -- | -- | -- | -- | -- | DQ275444 | -- |
| *Pachydactylus oculatus* | DQ275381 | AY123380 | -- | -- | AY123402 | -- | -- | -- | -- | -- | DQ275425 | -- |
| *Pachydactylus oreophilus* | DQ275398 | AY123384 | -- | -- | AY123406 | -- | -- | -- | -- | -- | DQ275442 | -- |
| *Pachydactylus oshaughnessyi* | DQ275391 | AY123391 | -- | -- | AY123413 | -- | -- | -- | -- | -- | DQ275435 | -- |
| *Pachydactylus parascutatus* | DQ275397 | AY123383 | -- | -- | AY123405 | -- | -- | -- | -- | -- | DQ275441 | -- |
| *Pachydactylus punctatus* | DQ275411 | AF449120 | -- | EU293691 | AF449135 | JX041393 | -- | -- | EU293713 | -- | EU293646 | EU293736 |
| *Pachydactylus purcelli* | -- | -- | -- | -- | DQ349161 | HQ165955 | -- | -- | HQ165970 | -- | HQ165985 | -- |
| *Pachydactylus rangei* | -- | -- | -- | JQ945599 | AY123397 | JX041394 | -- | -- | JQ945392 | -- | JQ945324 | JQ945492 |
| *Pachydactylus reconditus* | -- | -- | -- | -- | DQ349182 | -- | -- | -- | -- | -- | -- | -- |
| *Pachydactylus robertsi* | -- | -- | -- | -- | DQ349185 | -- | -- | -- | -- | -- | -- | -- |
| *Pachydactylus rugosus* | DQ275376 | AF449113 | -- | JQ945600 | AF449129 | JX041395 | -- | -- | JQ945393 | -- | DQ275420 | JQ945493 |
| *Pachydactylus sansteynae* | -- | -- | -- | -- | AY123404 | -- | -- | -- | -- | -- | -- | -- |
| *Pachydactylus scherzi* | DQ275392 | AY123379 | -- | -- | AY123401 | -- | -- | -- | -- | -- | DQ275436 | -- |
| *Pachydactylus scutatus* | DQ275395 | AY123381 | -- | -- | AY123403 | -- | -- | -- | -- | -- | DQ275439 | -- |
| *Pachydactylus serval* | DQ275385 | AY123386 | -- | -- | DQ349168 | HQ165957 | -- | -- | HQ165972 | -- | DQ275429 | -- |
| *Pachydactylus tigrinus* | DQ275390 | AY123390 | -- | -- | AY123412 | -- | -- | -- | -- | -- | DQ275434 | -- |
| *Pachydactylus tsodiloensis* | DQ275383 | AY123387 | -- | -- | AY123408 | -- | -- | -- | -- | -- | DQ275427 | -- |
| *Pachydactylus vansoni* | DQ275388 | AY123393 | -- | -- | AY123415 | -- | -- | -- | -- | -- | DQ275432 | -- |
| *Pachydactylus vanzyli* | -- | -- | -- | JQ945601 | AY123396 | JX041396 | -- | -- | JQ945394 | -- | JQ945326 | JQ945494 |
| *Pachydactylus waterbergensis* | -- | -- | -- | -- | DQ349176 | -- | -- | -- | -- | -- | -- | -- |
| *Pachydactylus weberi* | DQ275386 | AF449115 | -- | JQ945602 | AF449131 | HQ165961 | -- | -- | HQ165976 | -- | DQ275430 | JQ945495 |
| *Pamelaescincus gardineri* | AY649128 | AY151446 | -- | AY818804 | AF280128 | -- | -- | -- | -- | -- | -- | -- |
| *Panaspis breviceps* | AY308439 | AY308288 | -- | -- | -- | -- | -- | -- | -- | -- | -- | -- |
| *Panaspis togoensis* | AY308441 | AY308290 | -- | -- | -- | JF498127 | -- | -- | -- | JF498354 | -- | -- |
| *Pantherophis alleghaniensis* | -- | -- | -- | FJ627793 | AF283644 | -- | -- | -- | -- | -- | -- | -- |
| *Pantherophis bairdi* | AY122812 | -- | -- | DQ902061 | AY122729 | DQ902209 | -- | -- | -- | -- | -- | -- |
| *Pantherophis emoryi* | -- | -- | -- | FJ627791 | AF337173 | FJ627839 | -- | -- | -- | -- | -- | -- |
| *Pantherophis guttatus* | AY122842 | AM236349 | -- | DQ902070 | DQ902111 | DQ902218 | DQ902291 | -- | -- | AJ293982 | -- | -- |
| *Pantherophis obsoletus* | AY122843 | Z46493 | -- | FJ627805 | AF283632 | FJ627844 | -- | -- | -- | -- | -- | -- |
| *Pantherophis slowinskii* | NC_009769 | DQ523162 | -- | FJ627792 | AY582866 | DQ523162 | -- | -- | -- | -- | -- | -- |
| *Pantherophis spiloides* | -- | -- | -- | AF471140 | AF283643 | FJ627835 | -- | -- | -- | -- | -- | -- |
| *Pantherophis vulpinus* | AY122818 | -- | -- | DQ902089 | AY122734 | DQ902238 | DQ902306 | -- | -- | -- | -- | -- |
| *Papuascincus stanleyanus* | JF497877 | JF498000 | -- | -- | -- | JF498128 | JF498479 | -- | -- | JF498355 | -- | -- |
| *Paracontias brocchii* | AY391137 | AY391155 | FJ667655 | FJ667684 | AY391173 | -- | -- | -- | FJ667713 | -- | -- | FJ667742 |
| *Paracontias hildebrandti* | AY391138 | FJ667633 | FJ667662 | FJ667691 | AY391174 | -- | -- | -- | FJ667720 | HM161069 | HM161164 | FJ667749 |
| *Paracontias holomelas* | AY391139 | AY391157 | -- | AY391193 | AY391175 | -- | -- | -- | -- | -- | -- | -- |
| *Paracontias manify* | -- | -- | FJ667656 | FJ667685 | -- | -- | -- | -- | FJ667714 | -- | -- | FJ667743 |
| *Paracontias rothschildi* | FJ667618 | GU048759 | FJ667653 | FJ667682 | -- | -- | -- | -- | FJ667711 | -- | -- | FJ667740 |
| *Paradelma orientalis* | -- | -- | -- | FJ571642 | -- | AY134605 | -- | -- | HQ426215 | -- | FJ571626 | HQ426477 |
| *Paragehyra gabriellae* | -- | -- | -- | JQ945603 | -- | JX041399 | -- | -- | JQ945396 | -- | JQ945328 | JQ945496 |
| *Parahydrophis mertoni* | -- | DQ234048 | -- | FJ587177 | DQ233974 | -- | FJ593201 | -- | -- | -- | FJ587099 | -- |
| *Pareas boulengeri* | -- | -- | -- | JF827710 | JF827683 | -- | JF827661 | -- | -- | -- | -- | -- |
| *Pareas carinatus* | AF544773 | AF544802 | FJ433985 | AF544692 | JQ598940 | -- | GEA | FJ434086 | -- | EF144069 | -- | EF144096 |
| *Pareas formosensis* | -- | -- | -- | JF827720 | HQ528535 | -- | HQ528434 | -- | -- | -- | -- | -- |
| *Pareas hamptoni* | -- | -- | EU402657 | JF827703 | AY425809 | -- | JF827654 | EU390933 | -- | JN703093 | EU402860 | -- |
| *Pareas macularius* | -- | -- | -- | AF471150 | AF471082 | -- | -- | -- | -- | -- | -- | -- |
| *Pareas margaritophorus* | -- | -- | -- | JF827700 | AY425805 | -- | -- | -- | -- | -- | -- | -- |
| *Pareas monticola* | -- | -- | -- | JF827715 | JF827689 | -- | JF827666 | -- | -- | -- | -- | -- |
| *Pareas nuchalis* | -- | -- | -- | -- | -- | -- | U49311 | -- | -- | -- | -- | -- |
| *Parias flavomaculatus* | AY059535 | AY059551 | -- | -- | AY371831 | -- | AY059584 | -- | -- | -- | -- | -- |
| *Parias hageni* | AY371761 | AY059552 | -- | -- | AY059567 | -- | AY059585 | -- | -- | -- | -- | -- |
| *Parias malcolmi* | AY371758 | AY371793 | -- | -- | AY371832 | -- | AY371861 | -- | -- | -- | -- | -- |
| *Parias schultzei* | AY352785 | AY352725 | -- | -- | AY352756 | -- | AY352819 | -- | -- | -- | -- | -- |
| *Parias sumatranus* | AY371762 | AY371792 | -- | -- | AY371830 | -- | AY371866 | -- | -- | -- | -- | -- |
| *Paroedura androyensis* | GU128974 | GU129000 | -- | HQ256721 | EF490748 | EF490774 | EF536218 | -- | EF490695 | -- | EF490721 | -- |
| *Paroedura bastardi* | GU128980 | GU129005 | -- | HQ256725 | -- | EF536211 | EF536237 | -- | EF536187 | -- | EF536163 | -- |
| *Paroedura gracilis* | GU128969 | GU129012 | -- | HQ256726 | -- | EF536209 | EF536235 | -- | EF536185 | -- | EF536161 | -- |
| *Paroedura homalorhina* | -- | -- | -- | -- | -- | EF536214 | EF536240 | -- | EF536190 | -- | EF536166 | -- |
| *Paroedura karstophila* | -- | -- | -- | -- | EF490749 | EF490775 | EF536229 | -- | EF490696 | -- | EF490722 | -- |
| *Paroedura lohatsara* | GU128971 | GU128997 | -- | HQ256731 | -- | EF536203 | EF536228 | -- | EF536179 | -- | EF536155 | -- |
| *Paroedura masobe* | GU128977 | GU129003 | -- | HQ426560 | -- | EF536193 | EF536217 | -- | EF536169 | -- | EF536145 | HQ426478 |
| *Paroedura oviceps* | EU596615 | GU129009 | EU596850 | HQ256733 | -- | EF536208 | EF536234 | -- | EF536184 | -- | EF536160 | -- |
| *Paroedura picta* | GU128965 | GU128991 | -- | EU293692 | -- | EF536198 | EF536223 | -- | EF536174 | -- | EF536150 | EU293737 |
| *Paroedura sanctijohannis* | -- | -- | JQ073122 | -- | -- | EF536205 | EF536231 | -- | EF536181 | -- | EF536157 | -- |
| *Paroedura stumpffi* | GU128964 | GU129004 | JQ073118 | HQ256740 | -- | EF536202 | EF536227 | -- | EF536178 | -- | EF536154 | -- |
| *Paroedura tanjaka* | -- | -- | -- | -- | -- | EF536201 | EF536226 | -- | EF536177 | -- | EF536153 | -- |
| *Paroedura vazimba* | GU128981 | GU129008 | -- | HQ256742 | -- | EF536195 | EF536220 | -- | EF536171 | -- | EF536147 | -- |
| *Parvilacerta fraasii* | -- | GQ142106 | -- | GQ142148 | GQ142136 | -- | -- | -- | -- | -- | GQ142158 | -- |
| *Parvilacerta parva* | -- | GQ142105 | -- | EF632279 | GQ142135 | -- | -- | -- | -- | -- | EF632236 | -- |
| *Parvoscincus sisoni* | JF497902 | JF498030 | -- | -- | -- | JF498158 | JF498509 | -- | -- | JF498386 | -- | -- |
| *Pedioplanis breviceps* | -- | DQ871119 | -- | -- | -- | DQ871061 | -- | -- | -- | -- | DQ871177 | -- |
| *Pedioplanis burchelli* | AF042548 | DQ871123 | -- | -- | AF080339 | EU723566 | -- | -- | -- | -- | DQ871180 | -- |
| *Pedioplanis gaerdesi* | AY192434 | DQ871135 | -- | -- | AY184408 | AY184398 | -- | -- | -- | -- | DQ871192 | -- |
| *Pedioplanis husabensis* | -- | DQ871139 | -- | -- | -- | DQ871081 | -- | -- | -- | -- | DQ871196 | -- |
| *Pedioplanis inornata* | AY192435 | DQ871140 | -- | -- | AY184409 | AY184399 | -- | -- | -- | -- | DQ871194 | -- |
| *Pedioplanis laticeps* | -- | DQ871127 | -- | -- | -- | DQ871069 | -- | -- | -- | -- | DQ871184 | -- |
| *Pedioplanis lineoocellata* | -- | DQ871103 | -- | -- | -- | DQ871047 | -- | -- | -- | -- | DQ871163 | -- |
| *Pedioplanis namaquensis* | AF206613 | DQ871102 | -- | -- | AF206546 | -- | -- | -- | -- | -- | DQ871159 | -- |
| *Pedioplanis rubens* | -- | DQ871110 | -- | -- | -- | DQ871052 | -- | -- | -- | -- | DQ871168 | -- |
| *Pedioplanis undata* | AF042549 | DQ871115 | -- | EF632280 | -- | -- | -- | -- | -- | -- | EF632237 | -- |
| *Pelamis platura* | -- | DQ234052 | -- | -- | DQ233978 | -- | U49299 | -- | -- | -- | -- | -- |
| *Perochirus ateles* | -- | -- | -- | JQ945604 | -- | JN393946 | -- | -- | JN394015 | -- | JN393984 | JQ945497 |
| *Petracola ventrimaculatus* | -- | -- | -- | AY507910 | -- | -- | AY507894 | -- | -- | -- | -- | -- |
| *Petrosaurus mearnsi* | -- | L41450 | HQ876221 | -- | EF653316 | JN648436 | AF210354 | JF804557 | -- | HQ876333 | GQ896005 | -- |
| *Petrosaurus repens* | -- | -- | -- | -- | EF653318 | JN648438 | JN648458 | -- | -- | -- | -- | -- |
| *Petrosaurus thalassinus* | -- | L41451 | -- | -- | EF653317 | AF049858 | -- | -- | -- | -- | GQ896006 | -- |
| *Phalotris lativittatus* | JQ598825 | JQ598885 | -- | JQ598991 | -- | -- | -- | -- | -- | -- | -- | -- |
| *Phalotris lemniscatus* | GQ457817 | GQ457756 | JQ599039 | GQ457877 | JQ598941 | -- | -- | -- | -- | -- | -- | -- |
| *Phalotris mertensi* | JQ598826 | JQ598886 | -- | -- | -- | -- | -- | -- | -- | -- | -- | -- |
| *Phalotris nasutus* | GQ457818 | GQ457757 | -- | GQ895822 | GQ895880 | -- | -- | -- | -- | -- | -- | -- |
| *Phelsuma abbotti* | EU596618 | DQ270565 | EU596851 | AY221346 | FJ829977 | EU423290 | -- | -- | -- | -- | FJ830148 | FJ830239 |
| *Phelsuma andamanense* | AY221277 | FJ829894 | -- | FJ830063 | FJ829982 | -- | -- | -- | -- | -- | FJ830155 | FJ830246 |
| *Phelsuma antanosy* | -- | FJ829895 | -- | FJ830064 | FJ829983 | -- | -- | -- | -- | -- | FJ830156 | FJ830247 |
| *Phelsuma astriata* | AY221273 | FJ829897 | JQ073106 | AY221337 | FJ829985 | EU423286 | -- | -- | -- | -- | FJ830158 | FJ830249 |
| *Phelsuma barbouri* | -- | FJ829900 | -- | FJ830069 | FJ829988 | EU423289 | -- | -- | -- | -- | FJ830161 | FJ830252 |
| *Phelsuma berghofi* | -- | FJ829901 | -- | FJ830070 | FJ829989 | -- | -- | -- | -- | -- | FJ830162 | FJ830253 |
| *Phelsuma borbonica* | AY221286 | DQ270574 | -- | AY221350 | FJ829990 | JX041400 | -- | -- | HQ426216 | -- | FJ830163 | HQ426479 |
| *Phelsuma breviceps* | -- | FJ829902 | -- | FJ830072 | FJ829991 | -- | -- | -- | -- | -- | FJ830164 | FJ830255 |
| *Phelsuma cepediana* | AY221294 | FJ829903 | -- | FJ830073 | FJ829992 | EU423294 | -- | -- | -- | -- | FJ830165 | FJ830256 |
| *Phelsuma comorensis* | DQ911636 | FJ829904 | JQ073108 | -- | -- | -- | -- | -- | -- | -- | JQ073229 | FJ830257 |
| *Phelsuma dubia* | EF424452 | FJ829910 | -- | FJ830080 | FJ829996 | EU423285 | -- | -- | -- | -- | FJ830172 | FJ830263 |
| *Phelsuma edwardnewtoni* | AY221292 | -- | -- | AY221353 | AY221398 | -- | -- | -- | -- | -- | -- | -- |
| *Phelsuma flavigularis* | EF424466 | FJ829912 | -- | FJ830082 | FJ829997 | -- | -- | -- | -- | -- | FJ830174 | FJ830265 |
| *Phelsuma gigas* | AY221293 | -- | -- | AY221354 | AY221399 | -- | -- | -- | -- | -- | -- | -- |
| *Phelsuma guentheri* | AY221310 | FJ829914 | -- | FJ830084 | FJ830000 | -- | -- | -- | -- | -- | FJ830176 | FJ830267 |
| *Phelsuma guimbeaui* | -- | DQ270557 | -- | AY221372 | FJ830041 | EU423291 | -- | -- | HQ426217 | -- | FJ830221 | HQ426480 |
| *Phelsuma guttata* | -- | FJ829915 | -- | FJ830085 | FJ830001 | EU423284 | -- | -- | -- | -- | FJ830177 | FJ830268 |
| *Phelsuma hielscheri* | -- | FJ829916 | -- | FJ830086 | FJ830002 | -- | -- | -- | -- | -- | FJ830178 | FJ830269 |
| *Phelsuma inexpectata* | -- | FJ829917 | JQ073109 | FJ830087 | FJ830003 | JN393939 | -- | -- | JN394016 | -- | FJ830179 | FJ830270 |
| *Phelsuma kely* | -- | FJ829918 | -- | FJ830088 | FJ830004 | -- | -- | -- | -- | -- | FJ830180 | FJ830271 |
| *Phelsuma klemmeri* | -- | FJ829920 | -- | FJ830090 | FJ830006 | -- | -- | -- | -- | -- | FJ830182 | FJ830273 |
| *Phelsuma laticauda* | DQ911673 | AF215244 | -- | AY221343 | FJ830012 | EU423281 | -- | -- | JQ945398 | -- | FJ830189 | FJ830280 |
| *Phelsuma lineata* | AY221279 | GU734090 | HQ876236 | AY221342 | FJ830021 | EU423283 | -- | JN568338 | -- | HQ876379 | JN654861 | FJ830285 |
| *Phelsuma madagascariensis* | DQ852709 | FJ829937 | JQ073104 | EF534937 | EF424450 | EU423288 | -- | -- | AB081507 | -- | EF534811 | EF534979 |
| *Phelsuma malamakibo* | -- | FJ829938 | -- | FJ830108 | FJ830023 | -- | -- | -- | -- | -- | FJ830200 | FJ830291 |
| *Phelsuma modesta* | EF424465 | DQ270572 | -- | FJ830110 | EF424451 | EU423287 | -- | -- | HQ426218 | -- | FJ830202 | HQ426481 |
| *Phelsuma mutabilis* | AY221272 | FJ829944 | -- | FJ830114 | FJ830029 | -- | -- | -- | -- | -- | FJ830206 | FJ830297 |
| *Phelsuma nigristriata* | -- | FJ829947 | JQ073107 | FJ830117 | FJ830032 | -- | -- | -- | -- | -- | FJ830209 | FJ830300 |
| *Phelsuma ocellata* | -- | -- | EU596852 | FJ830141 | FJ830050 | EU423280 | -- | -- | HQ426219 | -- | FJ830233 | -- |
| *Phelsuma ornata* | AY221323 | FJ829948 | JQ073110 | FJ830118 | FJ830033 | EU423282 | -- | -- | -- | -- | FJ830210 | FJ830301 |
| *Phelsuma parkeri* | DQ911693 | FJ829949 | -- | FJ830119 | DQ911751 | -- | -- | -- | -- | -- | FJ830211 | FJ830302 |
| *Phelsuma pronki* | -- | FJ829951 | -- | FJ830121 | FJ830034 | -- | -- | -- | -- | -- | FJ830213 | FJ830304 |
| *Phelsuma pusilla* | -- | FJ829952 | -- | FJ830122 | FJ830035 | -- | -- | -- | -- | -- | FJ830214 | FJ830305 |
| *Phelsuma quadriocellata* | AY221282 | FJ829953 | -- | FJ830123 | FJ830036 | -- | -- | -- | -- | -- | FJ830215 | FJ830306 |
| *Phelsuma ravenala* | -- | FJ829908 | -- | FJ830078 | FJ829994 | -- | -- | -- | -- | -- | FJ830170 | FJ830261 |
| *Phelsuma robertmertensi* | DQ911643 | FJ829958 | -- | FJ830128 | DQ911701 | -- | -- | -- | -- | -- | FJ830220 | FJ830311 |
| *Phelsuma seippi* | -- | FJ829959 | -- | FJ830130 | FJ830042 | -- | -- | -- | -- | -- | FJ830222 | FJ830313 |
| *Phelsuma serraticauda* | AY221278 | HM030812 | -- | FJ830131 | FJ830043 | EU423296 | -- | -- | -- | -- | FJ830223 | FJ830314 |
| *Phelsuma standingi* | AY221281 | AF215243 | -- | FJ830133 | FJ830045 | -- | -- | -- | -- | -- | FJ830225 | FJ830316 |
| *Phelsuma sundbergi* | -- | -- | -- | -- | FJ830048 | EU423295 | -- | -- | -- | -- | FJ830228 | FJ830319 |
| *Phelsuma vanheygeni* | -- | FJ829966 | -- | FJ830137 | FJ830049 | -- | -- | -- | -- | -- | FJ830229 | FJ830320 |
| *Philochortus spinalis* | -- | -- | -- | EF632281 | -- | -- | -- | -- | -- | -- | EF632238 | -- |
| *Philodryas aestivus* | GQ457819 | GQ457758 | -- | GQ457879 | -- | -- | -- | -- | -- | -- | -- | -- |
| *Philodryas baroni* | JQ598828 | JQ598888 | -- | -- | AF236812 | -- | -- | -- | -- | -- | -- | -- |
| *Philodryas mattogrossensis* | GQ457820 | GQ457759 | -- | GQ457880 | -- | -- | -- | -- | -- | -- | -- | -- |
| *Philodryas nattereri* | JQ598829 | JQ598889 | -- | JQ598992 | AF236806 | -- | -- | -- | -- | -- | -- | -- |
| *Philodryas olfersii* | JQ598830 | JQ598890 | JQ599041 | JQ598993 | JQ598945 | -- | -- | -- | -- | -- | -- | -- |
| *Philodryas patagoniensis* | GQ457821 | GQ457760 | -- | GQ457881 | AF236808 | -- | -- | -- | -- | -- | -- | -- |
| *Philodryas psammophidea* | GU018149 | GU018168 | -- | -- | -- | -- | -- | -- | -- | -- | -- | -- |
| *Philodryas viridissima* | AF158419 | AF158474 | -- | -- | AF236807 | -- | -- | -- | -- | -- | -- | -- |
| *Philothamnus angolensis* | -- | AY611886 | -- | -- | -- | -- | -- | -- | -- | -- | -- | -- |
| *Philothamnus carinatus* | -- | AY611870 | -- | -- | -- | -- | -- | -- | -- | -- | -- | -- |
| *Philothamnus girardi* | -- | FJ913475 | -- | -- | -- | -- | -- | -- | -- | -- | -- | -- |
| *Philothamnus heterodermus* | -- | AY611856 | -- | AF471149 | AF471055 | -- | -- | -- | -- | -- | -- | -- |
| *Philothamnus hoplogaster* | -- | FJ913484 | -- | -- | -- | -- | -- | -- | -- | -- | -- | -- |
| *Philothamnus natalensis* | -- | AY611887 | -- | -- | -- | -- | -- | -- | -- | -- | -- | -- |
| *Philothamnus nitidus* | -- | AY611871 | -- | -- | -- | -- | -- | -- | -- | -- | -- | -- |
| *Philothamnus semivariegatus* | -- | AY611889 | -- | -- | -- | -- | -- | -- | -- | -- | -- | -- |
| *Philothamnus thomensis* | -- | FJ913480 | -- | -- | -- | -- | -- | -- | -- | -- | -- | -- |
| *Phimophis guerini* | GQ457822 | GQ457761 | -- | GQ457882 | -- | -- | -- | -- | -- | -- | -- | -- |
| *Phimophis iglesiasi* | JQ598831 | JQ598891 | -- | GQ895823 | GQ895881 | -- | -- | -- | -- | -- | -- | -- |
| *Phoboscincus garnieri* | -- | -- | -- | DQ675377 | -- | -- | -- | -- | -- | -- | DQ675317 | -- |
| *Phoenicolacerta cyanisparsa* | -- | -- | -- | DQ461721 | DQ461750 | -- | -- | -- | -- | -- | -- | -- |
| *Phoenicolacerta kulzeri* | FJ460596 | GQ142110 | -- | GQ142151 | DQ461764 | NC_011606 | NC_011606 | -- | -- | -- | GQ142161 | -- |
| *Phoenicolacerta laevis* | AJ238182 | AF149941 | -- | DQ461737 | DQ461752 | -- | -- | -- | -- | -- | EF632226 | -- |
| *Pholidobolus macbrydei* | AY507848 | AY507867 | GU457874 | AY507896 | -- | -- | AY507886 | GU456030 | -- | -- | GU457996 | -- |
| *Pholidobolus montium* | -- | AY046463 | -- | AF420820 | -- | -- | AF420884 | -- | -- | -- | -- | -- |
| *Phoxophrys nigrilabris* | AB031971 | AB031988 | -- | -- | -- | -- | -- | -- | -- | -- | -- | -- |
| *Phrynocephalus albolineatus* | AY053646 | AY053769 | -- | -- | -- | -- | -- | -- | -- | -- | -- | -- |
| *Phrynocephalus axillaris* | AY053656 | AY053779 | -- | -- | -- | AY396570 | -- | -- | -- | -- | -- | -- |
| *Phrynocephalus forsythii* | AY053659 | AY053784 | -- | -- | -- | EF375684 | -- | -- | -- | -- | -- | -- |
| *Phrynocephalus guttatus* | AY053678 | AY053800 | -- | -- | -- | GQ242221 | -- | -- | -- | -- | GQ242266 | -- |
| *Phrynocephalus helioscopus* | AY053682 | AY053817 | -- | -- | -- | GQ242182 | -- | -- | -- | -- | -- | -- |
| *Phrynocephalus interscapularis* | -- | -- | -- | -- | -- | AF128517 | -- | -- | -- | -- | -- | -- |
| *Phrynocephalus lidskii* | -- | -- | -- | -- | -- | EU275221 | -- | -- | -- | -- | -- | -- |
| *Phrynocephalus melanurus* | -- | -- | -- | -- | -- | GQ242198 | -- | -- | -- | -- | GQ242252 | -- |
| *Phrynocephalus mystaceus* | AY053700 | AY053822 | DQ340735 | AF137527 | -- | AF128518 | -- | JF804558 | -- | JF804596 | GQ242268 | -- |
| *Phrynocephalus przewalskii* | AY053702 | AY053828 | -- | -- | -- | GQ303142 | -- | -- | -- | -- | -- | -- |
| *Phrynocephalus putjatai* | -- | -- | -- | -- | -- | EF375636 | -- | -- | -- | -- | -- | -- |
| *Phrynocephalus raddei* | -- | -- | -- | -- | -- | U82691 | -- | -- | -- | -- | AY662586 | -- |
| *Phrynocephalus scutellatus* | -- | HQ901103 | -- | -- | -- | -- | -- | -- | -- | -- | -- | -- |
| *Phrynocephalus theobaldi* | AY053730 | AY053856 | -- | -- | -- | EF688071 | -- | -- | -- | -- | -- | -- |
| *Phrynocephalus versicolor* | AY053735 | AY053865 | -- | -- | -- | AY396579 | -- | -- | -- | -- | -- | -- |
| *Phrynocephalus vlangalii* | AY053749 | AY053880 | AF497714 | -- | -- | EF375649 | EU294095 | -- | -- | -- | -- | -- |
| *Phrynosoma asio* | -- | L41452 | DQ385321 | -- | AY141086 | DQ385351 | AY141048 | -- | -- | -- | DQ385409 | -- |
| *Phrynosoma blainvillii* | -- | -- | GQ279723 | -- | -- | GQ279451 | -- | -- | -- | -- | GQ279664 | -- |
| *Phrynosoma braconnieri* | -- | -- | -- | -- | AY141096 | -- | AY141060 | -- | -- | -- | -- | -- |
| *Phrynosoma cerroense* | -- | -- | GQ279677 | -- | -- | DQ385347 | -- | -- | -- | -- | GQ279623 | -- |
| *Phrynosoma cornutum* | DQ385390 | L41453 | AY987975 | AY987989 | AY141087 | DQ385344 | AY141049 | -- | -- | -- | FJ356738 | -- |
| *Phrynosoma coronatum* | GQ279528 | -- | DQ385335 | -- | AY141097 | DQ385349 | AY141050 | -- | -- | GQ464691 | GQ279625 | -- |
| *Phrynosoma ditmarsi* | DQ385400 | -- | DQ385333 | -- | AY141088 | DQ385353 | AY141051 | -- | -- | -- | DQ385421 | -- |
| *Phrynosoma douglassii* | -- | L41454 | -- | -- | AY141089 | U82686 | U71597 | -- | -- | -- | -- | -- |
| *Phrynosoma hernandesi* | DQ385389 | -- | DQ385324 | -- | AY141090 | DQ385343 | AY141053 | -- | -- | -- | DQ385412 | -- |
| *Phrynosoma mcallii* | DQ385402 | -- | DQ385328 | -- | AY141098 | DQ385355 | AY141054 | -- | -- | -- | DQ385417 | -- |
| *Phrynosoma modestum* | DQ385397 | L41455 | DQ385325 | -- | AY141091 | DQ385350 | AY141055 | -- | -- | -- | DQ385413 | -- |
| *Phrynosoma orbiculare* | DQ385399 | -- | DQ385326 | -- | AY141092 | DQ385352 | AY141056 | -- | -- | -- | DQ385414 | -- |
| *Phrynosoma platyrhinos* | DQ385401 | -- | DQ385330 | -- | AY141093 | DQ385354 | AY141057 | JF804559 | -- | JF804597 | DQ385419 | -- |
| *Phrynosoma solare* | DQ385388 | -- | DQ385327 | -- | AY141094 | DQ385342 | AY141058 | -- | -- | -- | DQ385415 | -- |
| *Phrynosoma taurus* | DQ385403 | -- | DQ385329 | -- | -- | DQ385356 | AY141059 | -- | -- | -- | -- | -- |
| *Phrynosoma wigginsi* | DQ385395 | -- | DQ385336 | -- | -- | DQ385348 | -- | -- | -- | -- | DQ385424 | -- |
| *Phyllodactylus bordai* | -- | -- | -- | HQ426565 | -- | JX041405 | -- | -- | HQ426220 | -- | HQ426309 | HQ426483 |
| *Phyllodactylus bugastrolepis* | -- | -- | -- | EU293676 | -- | JX041406 | -- | -- | EU293699 | -- | EU293631 | EU293721 |
| *Phyllodactylus davisi* | FJ662563 | FJ662563 | FJ662478 | FJ662499 | -- | -- | FJ662542 | -- | -- | -- | -- | -- |
| *Phyllodactylus delcampoi* | -- | -- | -- | HQ426566 | -- | JX041407 | -- | -- | HQ426221 | -- | HQ426310 | HQ426484 |
| *Phyllodactylus duellmani* | FJ662562 | FJ662562 | FJ662477 | FJ662498 | -- | -- | FJ662541 | -- | -- | -- | -- | -- |
| *Phyllodactylus homolepidurus* | -- | -- | -- | JQ945606 | -- | JX041408 | -- | -- | JQ945399 | -- | JQ945330 | JQ945499 |
| *Phyllodactylus lanei* | FJ662565 | HM012690 | FJ662480 | FJ662501 | -- | -- | FJ662544 | -- | -- | -- | -- | -- |
| *Phyllodactylus nocticolus* | FJ662583 | FJ662583 | FJ662489 | FJ662518 | -- | JX041409 | FJ662561 | -- | HQ426222 | -- | HQ426311 | HQ426485 |
| *Phyllodactylus paucituberculatus* | FJ662564 | FJ662564 | FJ662479 | FJ662500 | -- | -- | FJ662543 | -- | -- | -- | -- | -- |
| *Phyllodactylus reissii* | -- | -- | -- | EU293677 | -- | JX041410 | -- | -- | EU293700 | -- | EU293632 | EU293722 |
| *Phyllodactylus tuberculosus* | -- | -- | -- | EU293675 | -- | JX041411 | -- | -- | EU293698 | -- | EU293630 | EU293720 |
| *Phyllodactylus unctus* | FJ662568 | FJ662567 | FJ662482 | FJ662503 | -- | JX041412 | FJ662546 | -- | HQ426223 | -- | HQ426312 | HQ426486 |
| *Phyllodactylus wirshingi* | AY763262 | AY763286 | -- | JQ945607 | -- | JX041413 | -- | -- | JQ945400 | -- | JQ945331 | JQ945500 |
| *Phyllodactylus xanti* | FJ662569 | FJ662571 | FJ662483 | FJ662506 | -- | JN393940 | FJ662549 | -- | EF534849 | -- | EF534807 | EF534975 |
| *Phyllopezus maranjonensis* | -- | JN935557 | -- | EU293678 | -- | JX041416 | -- | -- | EU293701 | -- | EU293633 | EU293723 |
| *Phyllopezus periosus* | -- | JN935552 | -- | JN935475 | -- | -- | -- | -- | -- | -- | JN935434 | -- |
| *Phyllopezus pollicaris* | DQ852721 | JN935589 | -- | EU293680 | AY630401 | JX041417 | -- | -- | HQ426225 | -- | EU293635 | EU293725 |
| *Phyllorhynchus decurtatus* | -- | -- | -- | AF471098 | AF471083 | -- | -- | FJ434105 | -- | -- | -- | -- |
| *Phyllurus amnicola* | -- | -- | -- | -- | -- | JF807330 | -- | -- | -- | -- | -- | -- |
| *Phyllurus kabikabi* | -- | -- | -- | -- | -- | JF807331 | -- | -- | -- | -- | JF807379 | -- |
| *Phyllurus platurus* | -- | -- | -- | AY172941 | -- | JF807329 | -- | -- | HQ426226 | -- | FJ855443 | HQ426488 |
| *Phymaturus antofagastensis* | -- | -- | -- | -- | -- | AY661892 | -- | -- | -- | -- | -- | -- |
| *Phymaturus dorsimaculatus* | JF272814 | -- | -- | -- | JF272781 | -- | -- | -- | -- | -- | -- | -- |
| *Phymaturus indistinctus* | AY173870 | -- | -- | AY367880 | DQ237649 | AY661893 | AY367851 | -- | -- | -- | -- | -- |
| *Phymaturus mallimaccii* | AY662052 | -- | -- | -- | -- | AF305785 | -- | -- | -- | -- | -- | -- |
| *Phymaturus palluma* | AY662050 | -- | JF806024 | -- | -- | AF099216 | -- | JF804560 | -- | JF804598 | JF806209 | -- |
| *Phymaturus patagonicus* | -- | -- | -- | -- | -- | AY661894 | -- | -- | -- | -- | -- | -- |
| *Phymaturus punae* | AY662053 | -- | -- | -- | -- | AF305786 | -- | -- | -- | -- | -- | -- |
| *Phymaturus somuncurensis* | AY662051 | AF215261 | -- | -- | -- | AF049865 | -- | -- | -- | -- | AY662594 | -- |
| *Physignathus cocincinus* | AF236822 | AB031990 | AY987978 | AY987991 | AB263945 | U82690 | -- | JF804561 | -- | HQ876322 | AY662582 | -- |
| *Physignathus lesueurii* | AB031974 | AB031991 | DQ340737 | DQ340689 | -- | AF128463 | -- | JF804562 | -- | JF804599 | AY662581 | -- |
| *Pituophis catenifer* | FJ623965 | -- | -- | FJ627790 | AF337112 | FJ627842 | AF138764 | -- | -- | -- | -- | -- |
| *Pituophis deppei* | AY122827 | -- | -- | FJ627801 | FJ627818 | FJ627848 | AF141096 | -- | -- | -- | -- | -- |
| *Pituophis lineaticollis* | AF512746 | AF512746 | -- | FJ627804 | -- | FJ627841 | AY138767 | -- | -- | -- | -- | -- |
| *Pituophis melanoleucus* | AY122824 | -- | -- | FJ627797 | AF337110 | DQ902244 | AF141116 | -- | -- | -- | -- | -- |
| *Pituophis ruthveni* | FJ623966 | -- | -- | DQ902092 | AF337111 | -- | AF138772 | -- | -- | -- | -- | -- |
| *Pituophis vertebralis* | -- | -- | -- | FJ627789 | FJ627819 | FJ627840 | AF141128 | -- | -- | -- | -- | -- |
| *Placosoma cordylinum* | -- | AF420734 | -- | AF420823 | -- | -- | AF420879 | -- | -- | -- | -- | -- |
| *Placosoma glabellum* | -- | AF420742 | -- | AF420833 | -- | -- | AF420907 | -- | -- | -- | -- | -- |
| *Plagiopholis styani* | -- | -- | -- | EU496916 | EU496918 | -- | -- | -- | -- | -- | -- | -- |
| *Platyceps collaris* | AY039157 | -- | -- | AY486946 | AY486922 | -- | AY487053 | -- | -- | -- | -- | -- |
| *Platyceps florulentus* | AY039130 | -- | -- | AY486939 | AY486915 | AY487004 | AY487043 | -- | -- | -- | -- | -- |
| *Platyceps karelini* | AY647232 | -- | -- | AY486942 | AY486918 | AY487008 | AY487047 | -- | -- | -- | -- | -- |
| *Platyceps najadum* | AY039128 | -- | -- | AY486936 | AY486912 | AY487009 | AY487038 | -- | -- | -- | -- | -- |
| *Platyceps rhodorachis* | AY039154 | -- | -- | AY486945 | AY486921 | AY487012 | AY487051 | -- | -- | -- | -- | -- |
| *Platyceps rogersi* | AY039127 | AY188082 | -- | AY188002 | AY188041 | AY487013 | AY487052 | -- | -- | -- | -- | -- |
| *Platyceps ventromaculatus* | AY039136 | -- | -- | -- | -- | -- | -- | -- | -- | -- | -- | -- |
| *Platysaurus broadleyi* | HQ167136 | HQ167247 | -- | -- | -- | HQ167025 | -- | -- | -- | -- | -- | -- |
| *Platysaurus capensis* | HQ167137 | HQ167248 | -- | -- | AY167392 | U71329 | -- | -- | -- | -- | -- | -- |
| *Platysaurus intermedius* | HQ167141 | AF236037 | -- | -- | -- | HQ167030 | -- | -- | -- | -- | -- | -- |
| *Platysaurus minor* | HQ167139 | HQ167250 | -- | -- | -- | HQ167028 | -- | -- | -- | -- | -- | -- |
| *Platysaurus mitchelli* | HQ167140 | HQ167251 | -- | -- | -- | HQ167029 | -- | -- | -- | -- | -- | -- |
| *Platysaurus monotropis* | AF236038 | AF236038 | -- | -- | -- | -- | -- | -- | -- | -- | -- | -- |
| *Platysaurus pungweensis* | -- | -- | EU108363 | EU116686 | EU116516 | -- | -- | EU108029 | -- | JN568488 | JN654862 | -- |
| *Plestiodon anthracinus* | AY308346 | AY308197 | HQ907225 | -- | -- | AY662552 | JF498545 | -- | -- | HM161070 | AY662634 | -- |
| *Plestiodon barbouri* | EU202957 | DQ173534 | HM160605 | -- | EU203041 | -- | -- | -- | -- | HM161079 | HM161174 | -- |
| *Plestiodon brevirostris* | AY308347 | AY308198 | HM160598 | -- | -- | -- | -- | -- | -- | HM161072 | HM161167 | -- |
| *Plestiodon callicephalus* | -- | -- | HM160606 | -- | -- | -- | -- | -- | -- | HM161080 | HM161175 | -- |
| *Plestiodon capito* | -- | -- | HM160608 | -- | -- | -- | -- | -- | -- | HM161082 | HM161177 | -- |
| *Plestiodon chinensis* | JN117724 | -- | HM160610 | -- | -- | -- | -- | -- | -- | HM161084 | HM161179 | -- |
| *Plestiodon copei* | -- | -- | HM160612 | -- | -- | -- | -- | -- | -- | HM161086 | HM161181 | -- |
| *Plestiodon dugesii* | -- | -- | HM160616 | -- | -- | -- | -- | -- | -- | HM161090 | HM161185 | -- |
| *Plestiodon egregius* | AB016606 | AB016606 | HM160618 | -- | AB016606 | NC_000888 | NC_000888 | -- | -- | HM161092 | HM161187 | -- |
| *Plestiodon elegans* | JN117723 | AY649153 | HM160621 | -- | EU203036 | -- | -- | -- | -- | HM161095 | HM161190 | -- |
| *Plestiodon fasciatus* | AY308349 | AY308200 | HQ876228 | AY217869 | DQ241639 | -- | JF498546 | JF804547 | -- | HM161096 | HQ876444 | -- |
| *Plestiodon gilberti* | AY308350 | AY308201 | HM160624 | -- | -- | -- | -- | -- | -- | HM161098 | HM161193 | -- |
| *Plestiodon inexpectatus* | AY308353 | AY308204 | HM160626 | AY217888 | AY217837 | AY662550 | -- | -- | HQ426253 | HM161100 | AY662632 | DQ119628 |
| *Plestiodon japonicus* | EU202993 | EU203034 | HM160629 | -- | EU203143 | -- | -- | -- | -- | HM161103 | HM161198 | -- |
| *Plestiodon kishinouyei* | -- | -- | HM160631 | -- | -- | -- | -- | -- | -- | HM161105 | HM161200 | -- |
| *Plestiodon lagunensis* | -- | -- | HM160632 | -- | -- | -- | -- | -- | -- | HM161106 | HM161201 | -- |
| *Plestiodon laticeps* | AY218039 | AY308205 | EU108357 | EU116681 | EU116510 | -- | -- | EU108023 | -- | HM161107 | EU108531 | -- |
| *Plestiodon latiscutatus* | AY308355 | AY308206 | HM160635 | -- | EU203035 | -- | -- | -- | -- | HM161109 | HM161204 | -- |
| *Plestiodon longirostris* | AY649113 | AY649154 | HM160637 | -- | -- | -- | -- | -- | -- | HM161111 | HM161206 | -- |
| *Plestiodon lynxe* | AY649114 | AY308209 | HM160639 | -- | -- | -- | -- | -- | -- | HM161113 | HM161208 | -- |
| *Plestiodon marginatus* | EU202955 | EU202996 | HM160640 | -- | EU203038 | -- | -- | -- | -- | HM161114 | HM161209 | -- |
| *Plestiodon multivirgatus* | -- | -- | HM160645 | -- | -- | -- | -- | -- | -- | HM161119 | HM161214 | -- |
| *Plestiodon obsoletus* | AY649116 | AY649157 | HM160646 | -- | -- | -- | AY550688 | -- | -- | HM161120 | HM161215 | -- |
| *Plestiodon ochoterenae* | -- | -- | HM160648 | -- | -- | -- | -- | -- | -- | HM161122 | HM161217 | -- |
| *Plestiodon parviauriculatus* | -- | -- | HM160649 | -- | -- | -- | -- | -- | -- | HM161123 | HM161218 | -- |
| *Plestiodon parvulus* | -- | HM012697 | HM160651 | -- | -- | -- | -- | -- | -- | HM161124 | HM161220 | -- |
| *Plestiodon quadrilineatus* | JF497945 | JF498073 | HQ907223 | -- | -- | -- | JF498547 | -- | -- | HM161125 | HM161221 | -- |
| *Plestiodon reynoldsi* | AY649126 | AY308211 | HM160654 | -- | AF470654 | -- | -- | -- | -- | HM161127 | HM161223 | -- |
| *Plestiodon septentrionalis* | AY308363 | AY308214 | HM160656 | -- | -- | -- | AY169638 | -- | -- | HM161129 | HM161225 | -- |
| *Plestiodon skiltonianus* | AY308364 | AY308215 | HM160658 | AF315396 | -- | AY662551 | -- | -- | -- | HM161131 | AY662633 | -- |
| *Plestiodon stimpsonii* | AB543279 | EU202997 | HM160660 | -- | EU203039 | -- | -- | -- | -- | HM161133 | HM161229 | -- |
| *Plestiodon sumichrasti* | -- | -- | HM160661 | -- | -- | -- | -- | -- | -- | HM161134 | HM161230 | -- |
| *Plestiodon tamdaoensis* | -- | -- | HM160663 | -- | -- | -- | -- | -- | -- | HM161136 | HM161232 | -- |
| *Plestiodon tetragrammus* | -- | -- | HM160664 | -- | -- | -- | -- | -- | -- | HM161137 | HM161233 | -- |
| *Plestiodon tunganus* | -- | -- | HM160666 | -- | -- | -- | -- | -- | -- | HM161139 | HM161235 | -- |
| *Pletholax gracilis* | -- | AY134530 | -- | AY134566 | -- | AY134602 | -- | -- | HQ426227 | -- | FJ571631 | HQ426489 |
| *Plica lumaria* | AF362519 | -- | -- | -- | -- | -- | -- | -- | -- | -- | -- | -- |
| *Plica plica* | AB218961 | AB218961 | JF806028 | EF615737 | AB218961 | AF528748 | AB218961 | JF804573 | -- | JF804607 | FJ356742 | -- |
| *Plica umbra* | AF362521 | -- | -- | -- | -- | -- | -- | -- | -- | -- | -- | -- |
| *Podarcis atrata* | AJ250169 | -- | -- | -- | AY234160 | -- | AY234171 | -- | -- | -- | -- | -- |
| *Podarcis bocagei* | AF469425 | DQ081077 | -- | AF315399 | AF469426 | -- | EF081129 | -- | -- | -- | -- | -- |
| *Podarcis carbonelli* | AY214449 | DQ081080 | -- | -- | DQ081140 | -- | EF081155 | -- | -- | -- | -- | -- |
| *Podarcis erhardii* | AJ250160 | AY896242 | -- | -- | FJ895786 | -- | -- | -- | -- | -- | -- | -- |
| *Podarcis filfolensis* | DQ017660 | -- | -- | -- | AF133443 | -- | -- | -- | -- | -- | -- | -- |
| *Podarcis gaigeae* | AF133444 | AY768739 | -- | -- | AF133445 | -- | -- | -- | -- | -- | -- | -- |
| *Podarcis hispanicus* | -- | DQ081104 | -- | AY151997 | AY151907 | AY234144 | EU269587 | -- | -- | -- | -- | -- |
| *Podarcis lilfordi* | AJ250159 | -- | -- | EF679323 | EF990548 | EU006763 | -- | -- | -- | -- | -- | -- |
| *Podarcis melisellensis* | AY184998 | EU362088 | -- | -- | AY185097 | -- | -- | -- | -- | -- | -- | -- |
| *Podarcis milensis* | AJ250162 | AY768741 | -- | -- | AF133450 | -- | -- | -- | -- | -- | -- | -- |
| *Podarcis muralis* | AJ001470 | AY896190 | -- | EF632282 | DQ646343 | NC_011607 | EU269594 | -- | -- | -- | EF632239 | -- |
| *Podarcis peloponnesiacus* | -- | AY896177 | -- | -- | AY896124 | -- | -- | -- | -- | -- | -- | -- |
| *Podarcis pityusensis* | EF694768 | -- | -- | EF679328 | EF694828 | EU006764 | -- | -- | -- | -- | -- | -- |
| *Podarcis raffonei* | AJ250157 | -- | -- | -- | -- | -- | -- | -- | -- | -- | -- | -- |
| *Podarcis siculus* | AJ001476 | HM746964 | -- | EF679329 | EU916823 | EU006727 | NC_011609 | -- | HQ426255 | -- | -- | -- |
| *Podarcis tauricus* | -- | AY768727 | -- | -- | AY768763 | -- | -- | -- | -- | -- | -- | -- |
| *Podarcis tiliguerta* | DQ017657 | -- | -- | -- | AF133457 | -- | -- | -- | -- | -- | -- | -- |
| *Podarcis vaucheri* | AJ250167 | HQ898059 | -- | -- | AY234163 | AY234153 | EF081115 | -- | -- | -- | AY487363 | -- |
| *Pogona barbata* | -- | -- | DQ340738 | DQ340690 | -- | AF128474 | -- | -- | -- | -- | HQ662419 | -- |
| *Pogona henrylawsoni* | -- | -- | -- | -- | -- | AY133021 | -- | -- | -- | -- | HQ662416 | -- |
| *Pogona minima* | -- | -- | -- | -- | -- | AY133022 | -- | -- | -- | -- | -- | -- |
| *Pogona minor* | -- | -- | -- | -- | -- | AY133023 | -- | -- | -- | -- | HQ662418 | -- |
| *Pogona nullarbor* | -- | -- | -- | -- | -- | AY133025 | -- | -- | -- | -- | HQ662415 | -- |
| *Pogona vitticeps* | AB166795 | AB031992 | DQ340739 | DQ340691 | NC_006922 | AY133026 | NC_006922 | JF804563 | -- | JF804600 | JF806200 | -- |
| *Polemon acanthias* | FJ404138 | AY611848 | -- | AY611940 | AY612031 | -- | FJ404341 | -- | -- | -- | -- | FJ404413 |
| *Polemon collaris* | FJ404139 | AY611846 | -- | AY611938 | AY612029 | -- | FJ404342 | -- | -- | -- | -- | FJ404414 |
| *Polemon notatus* | -- | -- | -- | AY611939 | AY612030 | -- | FJ404343 | -- | -- | -- | -- | -- |
| *Polychrus acutirostris* | AF338331 | -- | -- | -- | -- | AF055925 | -- | -- | -- | -- | -- | -- |
| *Polychrus femoralis* | AF338335 | -- | -- | -- | -- | -- | -- | -- | -- | -- | -- | -- |
| *Polychrus gutturosus* | AF338338 | -- | -- | -- | -- | -- | -- | -- | -- | -- | -- | -- |
| *Polychrus marmoratus* | NC_012839 | NC_012839 | AY987966 | AY987983 | NC_012839 | AF528738 | NC_012839 | JF804564 | -- | HQ876335 | FJ356748 | -- |
| *Polyodontognathus caerulescens* | -- | JQ217154 | -- | -- | JQ217208 | -- | JQ217218 | -- | -- | -- | -- | -- |
| *Popeia popeiorum* | AY371754 | AY059558 | -- | -- | AY371821 | -- | AY059588 | -- | -- | -- | -- | -- |
| *Poromera fordii* | AF080368 | AF080370 | -- | EF632283 | AF080369 | -- | -- | -- | -- | -- | EF632240 | -- |
| *Porthidium dunni* | AY223654 | AY223667 | -- | -- | DQ061218 | -- | DQ061242 | -- | -- | -- | -- | -- |
| *Porthidium lansbergii* | AY223655 | AY223668 | -- | -- | DQ061206 | -- | DQ061231 | -- | -- | -- | -- | -- |
| *Porthidium nasutum* | AF057204 | EU624277 | -- | -- | DQ061210 | -- | U41887 | -- | -- | -- | -- | -- |
| *Porthidium ophryomegas* | AF057205 | AF057252 | -- | -- | DQ061216 | -- | U41888 | -- | -- | -- | -- | -- |
| *Porthidium porrasi* | DQ305421 | DQ305444 | -- | -- | DQ061214 | -- | DQ061239 | -- | -- | -- | -- | -- |
| *Porthidium yucatanicum* | JN870189 | JN870198 | -- | -- | DQ061215 | -- | DQ061244 | -- | -- | -- | -- | -- |
| *Potamites ecpleopus* | -- | -- | -- | AF420829 | -- | -- | AF420890 | -- | -- | -- | -- | -- |
| *Potamites juruazensis* | -- | -- | -- | AF420857 | -- | -- | AF420878 | -- | -- | -- | -- | -- |
| *Prasinohaema virens* | AY169596 | AY169633 | HQ655184 | HQ655219 | -- | -- | AY169671 | -- | -- | -- | -- | -- |
| *Pristidactylus scapulatus* | AF338333 | -- | -- | -- | -- | AF528732 | -- | -- | -- | -- | FJ356746 | -- |
| *Pristidactylus torquatus* | -- | L41456 | JF806025 | -- | -- | -- | -- | JF804565 | -- | JF804601 | JF806210 | -- |
| *Pristurus abdelkuri* | -- | -- | -- | -- | -- | GU271137 | -- | -- | -- | -- | GU256883 | -- |
| *Pristurus carteri* | DQ852720 | -- | -- | EF534929 | -- | GU271139 | -- | -- | EF534845 | -- | EF534803 | EF534971 |
| *Pristurus celerrimus* | -- | -- | -- | -- | -- | GU271140 | -- | -- | -- | -- | GU256886 | -- |
| *Pristurus crucifer* | -- | -- | -- | -- | -- | GU271141 | -- | -- | -- | -- | GU256887 | -- |
| *Pristurus flavipunctatus* | -- | -- | -- | -- | -- | GU271143 | -- | -- | -- | -- | GU256889 | -- |
| *Pristurus guichardi* | -- | -- | -- | -- | -- | GU271144 | -- | -- | -- | -- | GU256890 | -- |
| *Pristurus insignis* | -- | -- | -- | -- | -- | GU271145 | -- | -- | -- | -- | GU256891 | -- |
| *Pristurus minimus* | -- | -- | -- | -- | -- | -- | -- | -- | -- | -- | GU256892 | -- |
| *Pristurus rupestris* | -- | -- | -- | -- | -- | GU271142 | -- | -- | -- | -- | GU256888 | -- |
| *Pristurus sokotranus* | -- | -- | -- | -- | -- | GU271149 | -- | -- | -- | -- | GU256895 | -- |
| *Pristurus somalicus* | -- | -- | -- | -- | -- | GU271150 | -- | -- | -- | -- | GU256897 | -- |
| *Proablepharus reginae* | -- | -- | HQ655185 | HQ655220 | -- | -- | -- | -- | -- | -- | -- | -- |
| *Proatheris superciliaris* | EU624263 | EU624296 | -- | -- | AJ275685 | -- | EU624230 | -- | -- | -- | -- | -- |
| *Procellosaurinus erythrocercus* | AF420679 | AF420711 | -- | AF420836 | -- | -- | AF420870 | -- | -- | -- | -- | -- |
| *Procellosaurinus tetradactylus* | AF420703 | AF420713 | -- | AF420818 | -- | -- | AF420871 | -- | -- | -- | -- | -- |
| *Proctoporus bolivianus* | AY507850 | AY968828 | -- | AY234225 | -- | -- | AY968812 | -- | -- | -- | -- | -- |
| *Proctoporus guentheri* | AY507849 | AY507872 | -- | AY507901 | -- | -- | AY225185 | -- | -- | -- | -- | -- |
| *Proctoporus pachyurus* | AY968824 | AY968834 | -- | -- | -- | -- | AY968816 | -- | -- | -- | -- | -- |
| *Proctoporus subsolanus* | AY968822 | AY968833 | -- | -- | -- | -- | AY968814 | -- | -- | -- | -- | -- |
| *Proctoporus sucullucu* | AY507858 | AY507879 | -- | AY507906 | -- | -- | AY968817 | -- | -- | -- | -- | -- |
| *Proctoporus unsaacae* | AY507859 | AY507882 | -- | AY507909 | -- | -- | AY225186 | -- | -- | -- | -- | -- |
| *Proscelotes eggeli* | AY218032 | AY217982 | -- | AY662573 | AY217829 | AY662558 | -- | -- | -- | -- | AY662636 | -- |
| *Prosymna greigerti* | -- | JF340125 | -- | -- | -- | -- | -- | -- | -- | -- | -- | -- |
| *Prosymna janii* | FJ404193 | FJ404222 | -- | FJ387219 | FJ404319 | -- | FJ404389 | -- | -- | -- | -- | FJ404464 |
| *Prosymna meleagris* | -- | JF340123 | -- | -- | -- | -- | -- | -- | -- | -- | -- | -- |
| *Prosymna ruspolii* | -- | -- | -- | DQ486171 | DQ486347 | -- | DQ486323 | -- | -- | -- | -- | -- |
| *Prosymna visseri* | FJ404192 | AY188072 | -- | AY187994 | AY188033 | -- | -- | -- | -- | -- | -- | FJ404463 |
| *Protobothrops cornutus* | AY294276 | AY294267 | -- | -- | AY294272 | -- | AY294262 | -- | -- | -- | -- | -- |
| *Protobothrops elegans* | D31612 | AF057248 | -- | -- | AY223575 | -- | U41893 | -- | -- | -- | -- | -- |
| *Protobothrops flavoviridis* | -- | -- | -- | -- | AY223574 | -- | AY352826 | -- | -- | -- | -- | -- |
| *Protobothrops jerdonii* | AY294277 | EU809981 | -- | -- | AY763218 | -- | AY294263 | -- | -- | -- | -- | -- |
| *Protobothrops kaulbacki* | DQ666056 | DQ666055 | -- | -- | -- | -- | -- | -- | -- | -- | -- | -- |
| *Protobothrops mucrosquamatus* | AY763184 | AY763206 | -- | -- | AY763225 | -- | AY294265 | -- | -- | -- | -- | -- |
| *Protobothrops tokarensis* | -- | -- | -- | -- | AF038884 | -- | AY223628 | -- | -- | -- | -- | -- |
| *Protobothrops xiangchengensis* | AY763188 | AY763208 | -- | -- | HM567539 | -- | HM567470 | -- | -- | -- | -- | -- |
| *Psammodromus algirus* | DQ298635 | AY217970 | EU108364 | AY151998 | EU116517 | DQ150391 | DQ150380 | EU108030 | -- | -- | EF632241 | -- |
| *Psammodromus blanci* | DQ298608 | DQ298678 | -- | -- | DQ298563 | -- | -- | -- | -- | -- | -- | -- |
| *Psammodromus hispanicus* | DQ298607 | DQ298677 | -- | EF632285 | AF080378 | -- | FJ587826 | -- | -- | -- | EF632242 | -- |
| *Psammodynastes pictus* | FJ404186 | FJ404221 | -- | FJ387218 | FJ404318 | -- | FJ404383 | -- | -- | -- | -- | FJ404457 |
| *Psammodynastes pulverulentus* | AF544784 | AF544813 | -- | AF471157 | AF471031 | -- | -- | -- | -- | -- | -- | -- |
| *Psammophis angolensis* | -- | -- | -- | DQ486189 | DQ486439 | -- | DQ486275 | -- | -- | -- | -- | -- |
| *Psammophis biseriatus* | -- | -- | -- | -- | DQ486448 | -- | DQ486284 | -- | -- | -- | -- | -- |
| *Psammophis condanarus* | Z46450 | Z46479 | -- | AF471104 | DQ486466 | -- | AY058987 | -- | -- | -- | -- | -- |
| *Psammophis crucifer* | -- | -- | -- | DQ486188 | DQ486334 | -- | DQ486310 | -- | -- | -- | -- | -- |
| *Psammophis jallae* | -- | -- | -- | -- | DQ486409 | -- | DQ486247 | -- | -- | -- | -- | -- |
| *Psammophis leightoni* | -- | -- | -- | DQ486197 | DQ486467 | -- | DQ486303 | -- | -- | -- | -- | -- |
| *Psammophis leopardinus* | -- | -- | -- | -- | DQ486462 | -- | DQ486298 | -- | -- | -- | -- | -- |
| *Psammophis lineatus* | FJ404152 | FJ404216 | -- | FJ404256 | DQ486428 | -- | EU526861 | -- | -- | -- | -- | FJ404426 |
| *Psammophis lineolatus* | FJ404117 | AY188073 | -- | AY187995 | AY188034 | -- | DQ486286 | -- | -- | -- | -- | FJ404392 |
| *Psammophis mossambicus* | FJ404118 | FJ404217 | -- | DQ486185 | DQ486468 | -- | FJ404322 | -- | -- | -- | -- | FJ404393 |
| *Psammophis notostictus* | -- | -- | -- | DQ486182 | DQ486463 | -- | DQ486299 | -- | -- | -- | -- | -- |
| *Psammophis orientalis* | FJ404119 | FJ404218 | -- | -- | DQ486459 | -- | DQ486295 | -- | -- | -- | -- | FJ404394 |
| *Psammophis phillipsi* | FJ404120 | AY611879 | -- | -- | DQ486454 | -- | FJ404323 | -- | -- | -- | -- | FJ404395 |
| *Psammophis praeornatus* | FJ404153 | -- | -- | FJ387216 | -- | -- | FJ404355 | -- | -- | -- | -- | FJ404427 |
| *Psammophis punctulatus* | -- | -- | -- | DQ486186 | DQ486445 | -- | DQ486281 | -- | -- | -- | -- | -- |
| *Psammophis rukwae* | -- | -- | -- | -- | DQ486452 | -- | DQ486288 | -- | -- | -- | -- | -- |
| *Psammophis schokari* | FJ404121 | AY643356 | -- | DQ486194 | DQ486441 | -- | FJ404324 | -- | -- | -- | -- | FJ404396 |
| *Psammophis sibilans* | FJ404122 | FJ404219 | -- | FJ404228 | DQ486470 | -- | FJ404325 | -- | -- | -- | -- | FJ404397 |
| *Psammophis subtaeniatus* | -- | -- | -- | -- | DQ486415 | -- | DQ486253 | -- | -- | -- | -- | -- |
| *Psammophis sudanensis* | -- | -- | -- | DQ486184 | DQ486431 | -- | DQ486307 | -- | -- | -- | -- | -- |
| *Psammophis tanganicus* | -- | -- | -- | DQ486183 | DQ486381 | -- | DQ486220 | -- | -- | -- | -- | -- |
| *Psammophis trigrammus* | -- | -- | -- | DQ486196 | DQ486469 | -- | DQ486305 | -- | -- | -- | -- | -- |
| *Psammophylax acutus* | -- | -- | -- | DQ486192 | DQ486464 | -- | DQ486300 | -- | -- | -- | -- | -- |
| *Psammophylax rhombeatus* | FJ404124 | FJ404215 | -- | DQ486166 | DQ486342 | -- | FJ404327 | -- | -- | -- | -- | FJ404399 |
| *Psammophylax tritaeniatus* | -- | -- | -- | DQ486190 | DQ486451 | -- | DQ486287 | -- | -- | -- | -- | -- |
| *Psammophylax variabilis* | AF544774 | AY611864 | FJ433996 | DQ486193 | AY235724 | -- | AF544666 | FJ434097 | -- | EF144080 | AY487380 | EF144107 |
| *Pseudablabes agassizii* | GQ457823 | GQ457762 | -- | GQ457883 | GQ895883 | -- | -- | -- | -- | -- | -- | -- |
| *Pseudaspis cana* | FJ404187 | AY611898 | -- | DQ486167 | DQ486343 | -- | FJ404384 | -- | -- | -- | -- | FJ404458 |
| *Pseudechis australis* | AJ749363 | EU547144 | -- | -- | AY343091 | -- | AY340177 | -- | -- | -- | EU546873 | -- |
| *Pseudechis butleri* | AJ749360 | AJ749372 | -- | -- | AY340151 | -- | AY340180 | -- | -- | -- | -- | -- |
| *Pseudechis colletti* | AJ749353 | AJ749378 | -- | -- | AY340142 | -- | AY340171 | -- | -- | -- | -- | -- |
| *Pseudechis guttatus* | AJ749352 | AJ749371 | -- | -- | AY340143 | -- | AY340172 | -- | -- | -- | -- | -- |
| *Pseudechis papuanus* | AJ749354 | AJ749374 | -- | -- | AY340144 | -- | AY340173 | -- | -- | -- | -- | -- |
| *Pseudechis porphyriacus* | EU547096 | EU547145 | -- | -- | -- | -- | AY340170 | -- | -- | -- | EU546874 | -- |
| *Pseudelaphe flavirufa* | AY122840 | -- | -- | DQ902068 | DQ902109 | DQ902216 | DQ902289 | -- | -- | -- | -- | -- |
| *Pseudemoia entrecasteauxii* | EU837126 | EU837122 | -- | -- | EU837119 | -- | -- | -- | -- | -- | EU837131 | -- |
| *Pseudemoia pagenstecheri* | -- | -- | -- | -- | -- | DQ675267 | -- | -- | -- | -- | DQ675347 | -- |
| *Pseuderemias smithii* | -- | -- | -- | EF632286 | -- | -- | -- | -- | -- | -- | EF632243 | -- |
| *Pseudoacontias menamainty* | AY315511 | AY315560 | -- | -- | -- | -- | -- | -- | -- | -- | -- | -- |
| *Pseudoboa coronata* | GQ457824 | GQ457763 | -- | GQ457884 | -- | -- | -- | -- | -- | -- | -- | -- |
| *Pseudoboa neuwiedii* | AF158423 | AF158490 | -- | GQ895825 | GQ895884 | -- | -- | -- | -- | -- | -- | -- |
| *Pseudoboa nigra* | AF544775 | GQ457764 | JQ599043 | GQ457885 | JQ598948 | -- | -- | -- | -- | -- | -- | -- |
| *Pseudoboodon lemniscatus* | -- | -- | -- | DQ486174 | DQ486350 | -- | DQ486325 | -- | -- | -- | -- | -- |
| *Pseudocalotes brevipes* | -- | -- | -- | -- | AY572869 | AF128502 | -- | -- | -- | -- | -- | -- |
| *Pseudocalotes flavigula* | -- | -- | -- | -- | -- | AF128503 | -- | -- | -- | -- | -- | -- |
| *Pseudocerastes fieldi* | -- | -- | -- | -- | AJ275716 | -- | -- | -- | -- | -- | -- | -- |
| *Pseudocerastes persicus* | EU624264 | AJ275770 | -- | -- | AJ275717 | -- | -- | -- | -- | -- | -- | -- |
| *Pseudocyclophis persicus* | -- | -- | -- | AY376815 | AY376757 | -- | -- | -- | -- | -- | -- | -- |
| *Pseudoeryx plicatilis* | GQ457826 | GQ457765 | -- | GQ895826 | GQ895885 | -- | -- | -- | -- | -- | -- | -- |
| *Pseudoficimia frontalis* | -- | -- | -- | GQ895827 | GQ895886 | -- | -- | -- | -- | -- | -- | -- |
| *Pseudogekko compressicorpus* | -- | -- | -- | -- | -- | JQ437898 | -- | -- | JQ437941 | -- | -- | -- |
| *Pseudogekko smaragdinus* | -- | -- | -- | JQ945608 | -- | JQ437897 | -- | -- | JQ437940 | -- | JQ945332 | JQ945501 |
| *Pseudogonatodes guianensis* | -- | EU435276 | -- | EU435261 | -- | JX041421 | -- | -- | HQ426229 | -- | EF534784 | EF534950 |
| *Pseudogonatodes lunulatus* | GU139951 | -- | -- | GU139844 | -- | -- | -- | GU139897 | -- | -- | -- | -- |
| *Pseudogonatodes manessi* | GU139952 | -- | -- | GU139845 | -- | -- | -- | GU139898 | -- | -- | -- | -- |
| *Pseudoleptodeira latifasciata* | NC_013981 | EU728579 | -- | -- | EF078534 | EU728579 | EU728579 | FJ455190 | -- | -- | -- | -- |
| *Pseudoleptodeira uribei* | -- | -- | -- | -- | FJ810236 | -- | FJ810247 | FJ810243 | -- | -- | -- | -- |
| *Pseudonaja modesta* | EU547098 | EU547147 | -- | EU546915 | -- | -- | DQ098503 | -- | -- | -- | EU546876 | -- |
| *Pseudonaja textilis* | EU547097 | EU547146 | -- | EU546914 | -- | -- | DQ098645 | -- | -- | -- | EU546875 | -- |
| *Pseudopus apodus* | AF380955 | -- | GU457851 | -- | AF380965 | AF085623 | -- | GU456007 | -- | JN703073 | GU457974 | -- |
| *Pseudorabdion oxycephalum* | -- | -- | -- | DQ112083 | AF471073 | -- | -- | -- | -- | -- | -- | -- |
| *Pseudothecadactylus lindneri* | AF090177 | GU460145 | -- | AF090846 | -- | AY369024 | -- | -- | GU459747 | -- | AY662626 | HQ426492 |
| *Pseudotomodon trigonatus* | GQ457827 | GQ457766 | -- | GQ457887 | -- | -- | -- | -- | -- | -- | -- | -- |
| *Pseudotrapelus sinaitus* | NC_013603 | NC_013603 | -- | -- | AB116956 | AF128507 | NC_013603 | -- | -- | -- | -- | -- |
| *Pseudotyphlops philippinus* | KC347331 | KC347369 | -- | KC347409 | -- | -- | KC347522 | -- | -- | -- | KC347446 | -- |
| *Pseudoxenodon bambusicola* | JQ598833 | JQ598893 | JQ599044 | JQ598996 | -- | -- | -- | FJ434101 | -- | EF144084 | EF144090 | EF144111 |
| *Pseudoxenodon karlschmidti* | JQ598834 | JQ598894 | JQ599045 | AF471102 | AF471080 | -- | -- | -- | -- | -- | -- | -- |
| *Pseudoxenodon macrops* | JQ598835 | JQ598895 | -- | -- | JQ598949 | -- | -- | -- | -- | -- | -- | -- |
| *Pseudoxyrhopus ambreensis* | FJ404188 | AY188074 | -- | AY187996 | AY188035 | -- | FJ404385 | -- | -- | -- | -- | FJ404459 |
| *Pseustes sulphureus* | HM565766 | -- | -- | -- | -- | -- | -- | -- | -- | -- | -- | -- |
| *Psilophthalmus paeminosus* | AF420702 | AF420710 | -- | AF420825 | -- | -- | AF420872 | -- | -- | -- | -- | -- |
| *Psomophis genimaculatus* | GQ457828 | GQ457767 | -- | GQ457888 | -- | -- | -- | -- | -- | -- | -- | -- |
| *Psomophis joberti* | GQ457829 | GQ457768 | JQ599046 | GQ895828 | GQ895887 | -- | -- | -- | -- | -- | -- | -- |
| *Psomophis obtusus* | JQ598836 | JQ598896 | -- | -- | -- | -- | -- | -- | -- | -- | -- | -- |
| *Ptenopus carpi* | DQ852711 | -- | -- | AY172942 | -- | JX041422 | -- | -- | JQ945402 | -- | JQ945333 | JQ945502 |
| *Ptyas korros* | AF236680 | -- | -- | AY486953 | AY122652 | AY487023 | AY487062 | -- | -- | -- | -- | -- |
| *Ptyas mucosa* | AY122828 | FJ907950 | -- | AF471151 | AF471054 | AY487024 | AY487063 | -- | -- | -- | -- | -- |
| *Ptychoglossus brevifrontalis* | AF420697 | AF420757 | -- | AY507911 | -- | -- | AY507895 | -- | -- | -- | -- | -- |
| *Ptychophis flavovirgatus* | GQ457830 | GQ457769 | -- | GQ457890 | -- | -- | -- | -- | -- | -- | -- | -- |
| *Ptychozoon kuhli* | -- | -- | -- | JQ945610 | -- | JQ437918 | -- | -- | JQ437960 | -- | JQ945334 | JQ945503 |
| *Ptychozoon lionotum* | -- | -- | -- | JQ945611 | -- | JQ437914 | -- | -- | JQ437956 | -- | JQ945335 | JQ945504 |
| *Ptychozoon rhacophorus* | -- | -- | -- | -- | -- | JQ437913 | -- | -- | JQ437955 | -- | -- | -- |
| *Ptyctolaemus collicristatus* | -- | -- | -- | -- | -- | AY555837 | -- | -- | -- | -- | -- | -- |
| *Ptyctolaemus gularis* | -- | -- | -- | -- | -- | AY555838 | -- | -- | -- | -- | -- | -- |
| *Ptyodactylus guttatus* | -- | -- | -- | EU293681 | -- | JX041426 | -- | -- | EU293703 | -- | EU293636 | EU293726 |
| *Ptyodactylus hasselquistii* | GU195735 | GU195770 | -- | EU293682 | -- | -- | -- | -- | EU293704 | -- | EU293637 | EU293727 |
| *Ptyodactylus oudrii* | GU195746 | GU195780 | -- | -- | -- | -- | -- | -- | -- | -- | -- | -- |
| *Ptyodactylus ragazzii* | GU195733 | GU195781 | -- | -- | -- | -- | -- | -- | -- | -- | -- | -- |
| *Pygomeles braconnieri* | AY315514 | AY391159 | FJ667657 | FJ667686 | AY391177 | -- | -- | -- | FJ667715 | -- | -- | FJ667744 |
| *Pygopus lepidopodus* | -- | AY134531 | -- | FJ571643 | -- | AY134603 | -- | -- | HQ426231 | -- | FJ571627 | HQ426493 |
| *Pygopus nigriceps* | DQ852693 | AY134532 | -- | EF534907 | -- | AY134604 | -- | -- | EF534823 | -- | FJ571628 | EF534949 |
| *Python brongersmai* | EF545039 | EF545066 | -- | -- | EF545107 | -- | -- | -- | -- | -- | -- | -- |
| *Python curtus* | AF368060 | AF215277 | -- | -- | -- | -- | -- | -- | -- | -- | -- | -- |
| *Python molurus* | AF236682 | NC_15812 | EU402658 | AY099968 | AY099983 | NC_015812 | NC_015812 | -- | -- | JN703057 | -- | -- |
| *Python regius* | AB177878 | AB177878 | -- | -- | AF337116 | AB177878 | NC_007399 | -- | -- | -- | -- | -- |
| *Python sebae* | EF545037 | EF545064 | -- | -- | U69863 | -- | -- | -- | -- | -- | -- | -- |
| *Pythonodipsas carinata* | FJ404189 | AY188075 | -- | AY187997 | AY188036 | -- | FJ404386 | -- | -- | -- | -- | FJ404460 |
| *Quedenfeldtia moerens* | -- | -- | -- | HQ426574 | -- | JX041427 | -- | -- | HQ426232 | -- | HQ426320 | HQ426494 |
| *Quedenfeldtia trachyblepharus* | -- | -- | -- | EF534930 | -- | JX041428 | -- | -- | EF534846 | -- | EF534804 | EF534972 |
| *Rachidelus brazili* | JQ598837 | JQ598897 | JQ599048 | -- | JQ598952 | -- | -- | -- | -- | -- | -- | -- |
| *Ramphotyphlops acuticauda* | -- | -- | GU902381 | -- | -- | -- | -- | GU902554 | -- | -- | GU902631 | -- |
| *Ramphotyphlops albiceps* | -- | -- | GU902382 | -- | -- | -- | -- | GU902555 | -- | -- | GU902632 | -- |
| *Ramphotyphlops bicolor* | AY442900 | AY442836 | GU902410 | -- | -- | -- | -- | GU902581 | -- | -- | -- | -- |
| *Ramphotyphlops braminus* | DQ343649 | NC_10196 | FJ433959 | AY099980 | DQ343649 | AY662539 | NC_010196 | FJ434065 | HQ426256 | FJ433913 | AY662612 | FJ433889 |
| *Ramphotyphlops lineatus* | -- | -- | GU902384 | -- | -- | -- | -- | GU902557 | -- | -- | GU902634 | -- |
| *Ramphotyphlops polygrammicus* | -- | -- | GU902421 | -- | -- | -- | -- | GU902591 | -- | -- | GU902671 | -- |
| *Rankinia diemensis* | -- | -- | DQ340741 | DQ340693 | -- | AF375619 | -- | -- | -- | -- | HQ662421 | -- |
| *Regina alleni* | AF402633 | -- | -- | -- | AF402916 | AF384835 | -- | -- | -- | -- | -- | -- |
| *Regina grahami* | AF402635 | -- | -- | -- | AF402918 | AF384837 | -- | -- | -- | -- | -- | -- |
| *Regina rigida* | AF402636 | -- | -- | AF471120 | AF471052 | AF384838 | -- | -- | -- | -- | -- | -- |
| *Regina septemvittata* | AF402634 | -- | -- | -- | AF402917 | AF384836 | -- | -- | -- | -- | -- | -- |
| *Rhabdophis nuchalis* | AF236678 | -- | -- | JQ687438 | GQ281786 | JQ687454 | JQ687413 | -- | -- | -- | -- | -- |
| *Rhabdophis subminiatus* | AF544776 | AF544805 | JQ599047 | JQ687436 | JQ598951 | -- | JQ687411 | -- | -- | -- | -- | -- |
| *Rhabdophis tigrinus* | AF236679 | -- | -- | JQ687444 | GQ281785 | JQ687460 | JQ687419 | -- | -- | -- | -- | -- |
| *Rhachisaurus brachylepis* | -- | -- | -- | AF420853 | -- | -- | AF420877 | -- | -- | -- | -- | -- |
| *Rhacodactylus auriculatus* | -- | AF215248 | HQ876235 | AY172944 | -- | JF972429 | -- | JN568328 | -- | HQ876377 | JN654863 | -- |
| *Rhacodactylus chahoua* | -- | AF215249 | -- | -- | -- | DQ533741 | JQ398489 | -- | JQ173713 | -- | JQ173759 | -- |
| *Rhacodactylus ciliatus* | -- | AF215250 | -- | EF534902 | -- | -- | -- | -- | EF534818 | -- | EF534778 | EF534944 |
| *Rhacodactylus leachianus* | AF090176 | GU460148 | -- | AF090845 | -- | -- | -- | -- | GU459750 | -- | FJ855447 | JQ945505 |
| *Rhacodactylus sarasinorum* | -- | AF215257 | -- | -- | -- | -- | -- | -- | -- | -- | -- | -- |
| *Rhacodactylus trachyrhynchus* | AB028745 | AB028759 | -- | -- | -- | -- | -- | -- | -- | -- | -- | -- |
| *Rhadinaea flavilata* | -- | -- | -- | AF471152 | AF471078 | -- | -- | -- | -- | -- | -- | -- |
| *Rhadinaea fulvivittis* | -- | -- | -- | -- | EF078539 | -- | EF078587 | GU353275 | -- | -- | -- | -- |
| *Rhadinophis frenatum* | -- | -- | -- | DQ902069 | DQ902110 | DQ902217 | DQ902290 | -- | -- | -- | -- | -- |
| *Rhadinophis prasina* | -- | -- | -- | DQ902077 | DQ902119 | DQ902227 | DQ902299 | -- | -- | -- | -- | -- |
| *Rhamphiophis oxyrhynchus* | Z46443 | FJ404213 | -- | AF544710 | -- | -- | -- | -- | -- | -- | -- | FJ404400 |
| *Rhamphiophis rubropunctatus* | FJ404127 | -- | -- | FJ404232 | DQ486417 | -- | FJ404330 | -- | -- | -- | -- | FJ404402 |
| *Rhampholeon acuminatus* | AM055672 | AM055673 | -- | -- | -- | -- | -- | -- | -- | -- | -- | -- |
| *Rhampholeon beraduccii* | AM055666 | AM055667 | -- | -- | -- | -- | -- | -- | -- | -- | -- | -- |
| *Rhampholeon boulengeri* | DQ397241 | AY524878 | -- | -- | -- | AY524916 | -- | -- | -- | -- | AY524953 | -- |
| *Rhampholeon chapmanorum* | -- | AY524881 | -- | -- | -- | AY524919 | -- | -- | -- | -- | AY524956 | -- |
| *Rhampholeon marshalli* | -- | AY524872 | -- | -- | -- | AY524910 | -- | -- | -- | -- | AY524948 | -- |
| *Rhampholeon moyeri* | AM055668 | AY524876 | -- | -- | -- | AY524914 | -- | -- | -- | -- | AY524952 | -- |
| *Rhampholeon nchisiensis* | AM055676 | AY524886 | -- | -- | -- | AY524924 | -- | -- | -- | -- | AY524961 | -- |
| *Rhampholeon platyceps* | AM055680 | AY524880 | -- | -- | -- | AY524918 | -- | -- | -- | -- | EF114341 | -- |
| *Rhampholeon spectrum* | AM055682 | AY524865 | JQ073091 | FJ984254 | -- | AY524903 | AF443236 | -- | -- | -- | AY524940 | -- |
| *Rhampholeon spinosus* | AM055684 | DQ397233 | -- | -- | -- | -- | -- | -- | -- | -- | -- | -- |
| *Rhampholeon temporalis* | AM055687 | AY524867 | -- | -- | -- | AY524905 | -- | -- | -- | -- | AY524943 | -- |
| *Rhampholeon uluguruensis* | AM055689 | AY524897 | -- | -- | -- | AY524935 | -- | -- | -- | -- | -- | -- |
| *Rhampholeon viridis* | AM055697 | AM055698 | -- | -- | -- | -- | -- | -- | -- | -- | -- | -- |
| *Rhinechis scalaris* | AY122802 | -- | -- | AY486956 | AY122718 | AY487029 | AY487068 | -- | -- | -- | -- | -- |
| *Rhineura floridana* | EU203657 | EU203657 | FJ441848 | AY444022 | NC_006282 | NC_006282 | AY881080 | GU456034 | EU293714 | DQ119613 | AY662618 | DQ119631 |
| *Rhinobothryum lentiginosum* | AF158535 | AF158535 | -- | AF544693 | -- | -- | -- | -- | -- | -- | -- | -- |
| *Rhinocheilus lecontei* | AY122850 | -- | -- | FJ627788 | AY122766 | FJ627838 | AF138774 | -- | -- | -- | -- | -- |
| *Rhinophis blythii* | KC347332 | KC347370 | -- | KC347410 | -- | -- | KC347523 | -- | -- | -- | KC347447 | -- |
| *Rhinophis dorsimaculatus* | AY701009 | AY701040 | -- | -- | -- | -- | -- | -- | -- | -- | -- | -- |
| *Rhinophis drummondhayi* | AY700994 | AY701029 | FJ433966 | AF544719 | AF544673 | -- | -- | FJ434071 | -- | FJ433920 | AY487386 | FJ433896 |
| *Rhinophis homolepis* | KC347334 | KC347372 | -- | -- | KC347486 | -- | KC347525 | -- | -- | -- | -- | -- |
| *Rhinophis oxyrhynchus* | AY701014 | AY701045 | -- | -- | -- | -- | -- | -- | -- | -- | -- | -- |
| *Rhinophis philippinus* | GQ200594 | AY701048 | -- | -- | -- | -- | -- | -- | -- | -- | -- | -- |
| *Rhinophis travancoricus* | AY701010 | AY701041 | -- | -- | -- | -- | -- | -- | -- | -- | -- | -- |
| *Rhinoplocephalus bicolor* | EU547117 | EU547166 | -- | EU546930 | -- | -- | EU547020 | -- | -- | -- | EU546891 | -- |
| *Rhinoplocephalus nigrescens* | EU547119 | EU547168 | -- | -- | -- | -- | EU547022 | -- | -- | -- | EU546893 | -- |
| *Rhinotyphlops feae* | -- | -- | GU902385 | -- | -- | -- | -- | GU902558 | -- | -- | GU902635 | -- |
| *Rhinotyphlops lalandei* | -- | -- | GU902386 | -- | -- | -- | -- | GU902559 | -- | -- | GU902636 | -- |
| *Rhinotyphlops newtoni* | -- | -- | GU902388 | -- | -- | -- | -- | GU902561 | -- | -- | GU902638 | -- |
| *Rhinotyphlops schlegelii* | -- | -- | GU902449 | -- | -- | -- | -- | GU902619 | -- | -- | GU902695 | -- |
| *Rhoptropus afer* | DQ275409 | AY026931 | -- | JQ945613 | AY026923 | JX041430 | -- | -- | JQ945405 | -- | DQ275453 | JQ945506 |
| *Rhoptropus barnardi* | -- | AY026929 | -- | -- | AY026921 | -- | -- | -- | -- | -- | -- | -- |
| *Rhoptropus biporosus* | -- | AY026928 | -- | -- | AY026920 | -- | -- | -- | -- | -- | -- | -- |
| *Rhoptropus boultoni* | DQ852712 | AY026930 | -- | EF534936 | AY026922 | JX041431 | -- | -- | EF534852 | -- | EF534810 | EF534978 |
| *Rhoptropus bradfieldi* | EU596606 | AY026932 | EU596840 | JQ945614 | AY026924 | JX041432 | -- | -- | JQ945406 | -- | JQ945337 | JQ945507 |
| *Rhynchoedura ornata* | AF090181 | GU460153 | -- | AF090849 | -- | AY369014 | -- | -- | GU459755 | -- | GU459553 | JQ945508 |
| *Rhynchophis boulengeri* | -- | -- | -- | AF471153 | AF471053 | -- | -- | -- | -- | -- | -- | -- |
| *Riama colomaromani* | -- | -- | -- | AY507899 | -- | -- | AY507888 | -- | -- | -- | -- | -- |
| *Riama simoterus* | -- | -- | -- | AY507902 | -- | -- | AY507890 | -- | -- | -- | -- | -- |
| *Riama unicolor* | -- | -- | -- | AY507907 | -- | -- | AY507893 | -- | -- | -- | -- | -- |
| *Rieppeleon brachyurus* | AM055648 | AY524899 | -- | -- | -- | AY524937 | -- | -- | -- | -- | AY524969 | -- |
| *Rieppeleon brevicaudatus* | AF215134 | AY524889 | -- | FJ984255 | -- | AY524927 | AF443235 | -- | -- | -- | AY524964 | -- |
| *Rieppeleon kerstenii* | AB474918 | AY524894 | -- | -- | -- | AY524932 | -- | -- | -- | -- | AY524967 | -- |
| *Ristella rurkii* | AY308443 | AY308292 | -- | -- | -- | -- | -- | -- | -- | -- | -- | -- |
| *Saiphos equalis* | AY169597 | AY169634 | -- | -- | AF373260 | AF373279 | AY169672 | -- | -- | -- | -- | -- |
| *Salea horsfieldii* | -- | -- | -- | -- | -- | AF128490 | -- | -- | -- | -- | -- | -- |
| *Salea kakhienensis* | -- | -- | -- | -- | -- | GQ502784 | -- | -- | -- | -- | -- | -- |
| *Saltuarius cornutus* | -- | -- | HQ876234 | -- | AF109521 | AY369023 | -- | JF804567 | -- | HQ876376 | HQ876451 | -- |
| *Saltuarius kateae* | -- | -- | -- | -- | EU625330 | -- | -- | -- | -- | -- | -- | -- |
| *Saltuarius moritzi* | -- | -- | -- | -- | EU625317 | -- | -- | -- | -- | -- | -- | -- |
| *Saltuarius salebrosus* | -- | -- | -- | -- | EU625342 | -- | -- | -- | -- | -- | -- | -- |
| *Saltuarius swaini* | -- | GU460141 | -- | FJ855464 | EU625326 | -- | -- | -- | GU459743 | -- | FJ855444 | JQ945509 |
| *Saltuarius wyberba* | -- | -- | -- | -- | EU625339 | JF807327 | -- | -- | -- | -- | -- | -- |
| *Salvadora mexicana* | -- | -- | -- | AY486958 | AY486934 | AY487036 | AY487075 | -- | -- | -- | -- | -- |
| *Sanzinia madagascariensis* | AF368055 | AF215272 | AY988033 | AY099982 | U69866 | -- | -- | -- | -- | -- | AY988067 | -- |
| *Saproscincus basiliscus* | -- | AY626728 | HQ655187 | EF450998 | -- | -- | FJ195252 | -- | -- | -- | -- | -- |
| *Saproscincus challengeri* | -- | AY626750 | -- | -- | -- | -- | AY626703 | -- | -- | -- | -- | -- |
| *Saproscincus czechurai* | -- | AY626733 | -- | -- | -- | -- | FJ195291 | -- | -- | -- | -- | -- |
| *Saproscincus hannahae* | -- | AY626737 | -- | -- | -- | -- | AY626690 | -- | -- | -- | -- | -- |
| *Saproscincus lewisi* | -- | AY626716 | -- | -- | -- | -- | FJ195205 | -- | -- | -- | -- | -- |
| *Saproscincus mustelinus* | EU837127 | EU837123 | -- | -- | EU837118 | EF567305 | AY626710 | -- | -- | -- | EU837130 | -- |
| *Saproscincus oriarius* | -- | AY626752 | -- | -- | -- | -- | AY626705 | -- | -- | -- | -- | -- |
| *Saproscincus rosei* | -- | AY626745 | -- | -- | -- | DQ675248 | AY626698 | -- | -- | -- | DQ675328 | -- |
| *Saproscincus spectabilis* | -- | AY626748 | -- | -- | -- | -- | AY626701 | -- | -- | -- | -- | -- |
| *Saproscincus tetradactylus* | -- | AY626730 | -- | -- | -- | -- | FJ195325 | -- | -- | -- | -- | -- |
| *Saurodactylus fasciatus* | EU014299 | -- | -- | EU014323 | -- | JX041434 | EU014343 | -- | HQ426234 | -- | EU014354 | HQ426495 |
| *Saurodactylus mauritanicus* | EU014315 | -- | -- | EU014324 | -- | JX041435 | EU014349 | GU139899 | HQ426235 | -- | EU014356 | HQ426496 |
| *Sauromalus ater* | -- | -- | JF806026 | AF315400 | AF020247 | U82687 | U66232 | JF804568 | -- | JF804603 | AY662591 | -- |
| *Sauromalus hispidus* | -- | -- | -- | -- | AF020240 | -- | -- | -- | -- | -- | -- | -- |
| *Sauromalus klauberi* | -- | -- | -- | HM352536 | -- | -- | HM352519 | HM352527 | -- | -- | -- | -- |
| *Sauromalus varius* | -- | -- | -- | -- | AF020245 | -- | U66233 | -- | -- | -- | -- | -- |
| *Scaphiodontophis annulatus* | -- | -- | -- | GQ927318 | GQ927323 | -- | -- | -- | -- | -- | GQ927325 | -- |
| *Scaphiophis albopunctatus* | -- | -- | -- | DQ486169 | DQ486345 | -- | -- | -- | -- | -- | -- | -- |
| *Scelarcis perspicillata* | AF206602 | GQ142097 | -- | GQ142145 | GQ142122 | -- | -- | -- | -- | -- | GQ142155 | -- |
| *Sceloporus adleri* | AF000799 | -- | -- | JN090145 | -- | AY297519 | GQ895850 | -- | -- | -- | -- | -- |
| *Sceloporus aeneus* | -- | -- | -- | -- | -- | -- | U88280 | -- | -- | -- | -- | -- |
| *Sceloporus angustus* | -- | -- | -- | -- | -- | AF049859 | AF210360 | -- | -- | -- | -- | -- |
| *Sceloporus arenicolus* | GQ464524 | AF000863 | GQ464421 | -- | EF558634 | -- | GQ895848 | -- | -- | GQ464645 | GQ464701 | -- |
| *Sceloporus bicanthalis* | EU086047 | AF000840 | GQ464435 | -- | -- | -- | U88269 | -- | -- | GQ464659 | EU085725 | -- |
| *Sceloporus bulleri* | DQ525887 | EF608028 | -- | -- | -- | -- | DQ525865 | -- | -- | -- | -- | -- |
| *Sceloporus carinatus* | -- | -- | -- | -- | -- | AY297496 | AF210348 | -- | -- | -- | -- | -- |
| *Sceloporus cautus* | AF440019 | -- | GQ464447 | -- | -- | AY297522 | GQ895840 | -- | -- | GQ464671 | GQ464727 | -- |
| *Sceloporus chaneyi* | -- | -- | -- | -- | -- | -- | U88291 | -- | -- | -- | -- | -- |
| *Sceloporus chrysostictus* | -- | -- | -- | -- | -- | -- | AF210367 | -- | -- | -- | -- | -- |
| *Sceloporus clarkii* | EU086041 | -- | GQ464412 | -- | -- | AY297511 | EU085836 | -- | -- | GQ464636 | EU085719 | -- |
| *Sceloporus consobrinus* | -- | -- | GQ494864 | -- | -- | -- | -- | -- | -- | GQ494834 | GQ494819 | -- |
| *Sceloporus couchii* | GQ464528 | AF000829 | GQ464461 | -- | -- | -- | AF210364 | -- | -- | GQ464685 | GQ896008 | -- |
| *Sceloporus cozumelae* | AF000790 | AF000830 | -- | -- | -- | -- | -- | -- | -- | -- | -- | -- |
| *Sceloporus cryptus* | GQ464529 | AF000842 | GQ464415 | -- | -- | -- | GQ464753 | -- | -- | GQ464639 | GQ464695 | -- |
| *Sceloporus cyanogenys* | AF154144 | DQ525910 | -- | -- | -- | AY297524 | DQ525868 | -- | -- | -- | -- | -- |
| *Sceloporus dugesii* | AF154170 | L41461 | GQ464439 | -- | -- | -- | DQ525878 | -- | -- | GQ464663 | GQ464719 | -- |
| *Sceloporus edwardtaylori* | GQ464531 | -- | EU085926 | -- | -- | -- | EU085837 | -- | -- | GQ464638 | EU085720 | -- |
| *Sceloporus formosus* | GQ464532 | L41462 | GQ464416 | -- | -- | AY297498 | AF210346 | -- | -- | GQ464640 | GQ464696 | -- |
| *Sceloporus gadoviae* | GQ464533 | AF000836 | GQ464453 | -- | -- | -- | AF210336 | -- | -- | GQ464677 | GQ896010 | -- |
| *Sceloporus goldmani* | -- | -- | -- | -- | -- | -- | U88290 | -- | -- | -- | -- | -- |
| *Sceloporus graciosus* | AF440090 | L41463 | EU085928 | -- | EF558641 | AF049860 | EU085839 | -- | -- | GQ464646 | EU085722 | -- |
| *Sceloporus grammicus* | GQ464535 | -- | EU085929 | AF039478 | -- | AY297509 | EU085840 | -- | -- | GQ464648 | EU085723 | -- |
| *Sceloporus grandaevus* | GQ464536 | -- | GQ464452 | -- | -- | -- | AF210359 | -- | -- | GQ464676 | GQ464732 | -- |
| *Sceloporus heterolepis* | GQ464537 | DQ525907 | GQ464425 | -- | -- | -- | DQ525869 | -- | -- | GQ464649 | GQ464705 | -- |
| *Sceloporus horridus* | AF440091 | AF000844 | AY987976 | -- | -- | -- | EF025744 | -- | -- | GQ464661 | GQ464717 | -- |
| *Sceloporus hunsakeri* | GQ464539 | AF000845 | EU085846 | -- | -- | AY297506 | EU085818 | -- | -- | GQ464651 | EU085642 | -- |
| *Sceloporus insignis* | AF154133 | AF000846 | -- | -- | -- | -- | -- | -- | -- | -- | -- | -- |
| *Sceloporus jalapae* | -- | AF000837 | GQ464456 | -- | -- | AY297504 | AF210333 | -- | -- | GQ464680 | GQ464736 | -- |
| *Sceloporus jarrovii* | GQ464541 | L41465 | GQ464440 | -- | EU543743 | EU543774 | AF210343 | -- | -- | GQ464664 | GQ464720 | -- |
| *Sceloporus licki* | GQ464542 | -- | EU085847 | -- | -- | -- | EU085819 | -- | -- | GQ464652 | EU085643 | -- |
| *Sceloporus lineatulus* | AF000810 | AF000850 | -- | -- | -- | -- | -- | -- | -- | -- | -- | -- |
| *Sceloporus lundelli* | -- | -- | -- | -- | -- | AY297499 | -- | -- | -- | -- | -- | -- |
| *Sceloporus macdougalli* | GQ464544 | AF000849 | GQ464442 | -- | -- | -- | GQ464768 | -- | -- | GQ464666 | GQ464722 | -- |
| *Sceloporus maculosus* | GQ464545 | AF000838 | GQ464458 | -- | -- | AY297501 | EU085841 | -- | -- | GQ464682 | EU085724 | -- |
| *Sceloporus magister* | AF440092 | AF440092 | EU085854 | -- | -- | AF528741 | EU085815 | -- | -- | GQ464653 | EU085706 | -- |
| *Sceloporus malachiticus* | GQ464547 | L41467 | GQ464417 | -- | -- | AY297518 | GQ895852 | -- | -- | GQ464641 | GQ464697 | -- |
| *Sceloporus megalepidurus* | AF440093 | AF000862 | GQ464432 | -- | -- | AY297500 | AF210358 | -- | -- | GQ464656 | GQ464712 | -- |
| *Sceloporus melanorhinus* | GQ464549 | HM012693 | GQ464413 | -- | -- | -- | GQ464773 | -- | -- | GQ464637 | GQ464693 | -- |
| *Sceloporus merriami* | GQ464550 | -- | GQ464459 | -- | AY141103 | AY297520 | AY141064 | -- | -- | GQ464683 | GQ464739 | -- |
| *Sceloporus minor* | DQ525891 | DQ525908 | -- | -- | -- | -- | DQ525872 | -- | -- | -- | -- | -- |
| *Sceloporus mucronatus* | AF440094 | L41469 | GQ464443 | -- | JF317190 | -- | GQ464775 | -- | -- | GQ464667 | GQ464723 | -- |
| *Sceloporus nelsoni* | -- | -- | -- | -- | -- | -- | AF210351 | -- | -- | -- | -- | -- |
| *Sceloporus occidentalis* | AF440023 | AB079242 | GQ464448 | -- | NC_005960 | NC_005960 | NC_005960 | -- | -- | GQ464672 | GQ464728 | -- |
| *Sceloporus ochoterenae* | GQ464553 | AF000853 | GQ464457 | -- | -- | AF528743 | AF210332 | -- | -- | GQ464681 | GQ464737 | -- |
| *Sceloporus olivaceus* | AF440095 | AF440095 | GQ464434 | -- | -- | AY297521 | AF210361 | -- | -- | GQ464658 | GQ464714 | -- |
| *Sceloporus orcutti* | GQ464555 | L41472 | GQ464430 | -- | -- | AY297508 | EU085823 | -- | -- | GQ464654 | EU085710 | -- |
| *Sceloporus ornatus* | AF154137 | DQ525896 | GQ464444 | -- | -- | AY297523 | DQ525862 | -- | -- | GQ464668 | GQ464724 | -- |
| *Sceloporus palaciosi* | GQ464557 | -- | GQ464426 | -- | -- | -- | GQ464781 | -- | -- | GQ464650 | GQ464706 | -- |
| *Sceloporus parvus* | GQ464558 | AF000832 | GQ464462 | -- | -- | -- | AF210366 | -- | -- | GQ464686 | GQ896019 | -- |
| *Sceloporus poinsettii* | AF154176 | L41473 | GQ464445 | -- | -- | DQ357990 | DQ358011 | -- | -- | GQ464669 | GQ464725 | -- |
| *Sceloporus pyrocephalus* | GQ464561 | AF000833 | GQ464454 | -- | -- | AY297502 | AF210353 | -- | -- | GQ464678 | GQ464734 | -- |
| *Sceloporus samcolemani* | -- | -- | -- | -- | -- | -- | JN985719 | -- | -- | -- | -- | -- |
| *Sceloporus scalaris* | GQ464562 | L41474 | GQ464436 | -- | -- | AF528742 | AF210368 | -- | -- | GQ464660 | GQ464716 | -- |
| *Sceloporus serrifer* | DQ525882 | DQ525900 | -- | -- | JF317189 | -- | DQ525874 | -- | -- | -- | -- | -- |
| *Sceloporus siniferus* | GQ464563 | AF000834 | GQ464460 | -- | -- | AY297494 | AF210350 | -- | -- | GQ464684 | GQ464740 | -- |
| *Sceloporus slevini* | -- | -- | -- | -- | -- | -- | JN985737 | -- | -- | -- | -- | -- |
| *Sceloporus smaragdinus* | EU086043 | AF000855 | EU085927 | -- | -- | AY297517 | EU085838 | -- | -- | -- | EU085721 | -- |
| *Sceloporus smithi* | GQ464564 | AF000868 | GQ464463 | -- | -- | -- | GQ464788 | -- | -- | GQ464687 | GQ464743 | -- |
| *Sceloporus spinosus* | GQ464565 | L41475 | GQ464438 | -- | -- | AY297525 | AF210342 | -- | -- | GQ464662 | GQ464718 | -- |
| *Sceloporus squamosus* | -- | -- | -- | -- | -- | AY297495 | AF210347 | -- | -- | -- | -- | -- |
| *Sceloporus stejnegeri* | GQ464566 | AF000856 | GQ464418 | -- | -- | -- | GQ464790 | -- | -- | GQ464642 | GQ464698 | -- |
| *Sceloporus subpictus* | GQ464567 | AF000857 | GQ464419 | -- | -- | -- | AF210355 | -- | -- | GQ464643 | GQ464699 | -- |
| *Sceloporus taeniocnemis* | GQ464568 | L41476 | GQ464420 | -- | -- | -- | GQ464792 | -- | -- | GQ464644 | GQ464700 | -- |
| *Sceloporus teapensis* | -- | -- | -- | -- | -- | AY297505 | -- | -- | -- | -- | -- | -- |
| *Sceloporus torquatus* | EU086048 | L41477 | EU085932 | -- | JF317191 | -- | EU085843 | -- | -- | GQ464670 | EU085726 | -- |
| *Sceloporus undulatus* | L28075 | L41478 | GQ494865 | -- | -- | DQ357989 | EU045304 | -- | -- | GQ494837 | GQ464729 | -- |
| *Sceloporus utiformis* | GQ464571 | HM012692 | GQ464455 | -- | -- | AF528740 | AF210340 | -- | -- | GQ464679 | GQ464735 | -- |
| *Sceloporus vandenburgianus* | GQ464572 | AF000861 | GQ464423 | -- | -- | -- | GQ464796 | -- | -- | GQ464647 | GQ464703 | -- |
| *Sceloporus variabilis* | GQ464573 | L41479 | GQ464464 | -- | -- | AY297507 | EU085844 | JF804569 | -- | JF804604 | EU085727 | -- |
| *Sceloporus virgatus* | AF440085 | L41480 | GQ464450 | -- | -- | DQ357988 | DQ358009 | -- | -- | -- | GQ464730 | -- |
| *Sceloporus woodi* | AF440088 | AF000858 | GQ464451 | -- | AF144635 | AY297513 | GQ464799 | -- | -- | GQ464675 | GQ464731 | -- |
| *Sceloporus zosteromus* | EU086036 | AF000851 | EU085922 | -- | -- | AY297503 | EU085835 | -- | -- | GQ464655 | EU085715 | -- |
| *Scelotes anguineus* | -- | -- | -- | AY217878 | AY217827 | AY662559 | -- | -- | -- | -- | AY662635 | -- |
| *Scelotes arenicolus* | -- | -- | -- | AY217886 | AY217835 | -- | -- | -- | -- | -- | -- | -- |
| *Scelotes bipes* | AY218028 | AY028892 | -- | AY217876 | AY217825 | -- | -- | -- | -- | -- | -- | -- |
| *Scelotes caffer* | AY218035 | AY217985 | -- | AY217883 | AY217832 | -- | -- | -- | -- | -- | -- | -- |
| *Scelotes gronovii* | AY218036 | AY217986 | -- | AY217884 | AY217833 | -- | -- | -- | -- | -- | -- | -- |
| *Scelotes kasneri* | AY218037 | AY217987 | -- | AY217885 | AY217834 | -- | -- | -- | -- | -- | -- | -- |
| *Scelotes mirus* | AY649130 | AY028893 | JQ073075 | AY217879 | AY217828 | -- | AF228558 | -- | -- | -- | JQ073196 | -- |
| *Scelotes montispectus* | AY218027 | AY217978 | -- | AY217875 | AY217824 | -- | -- | -- | -- | -- | -- | -- |
| *Scelotes sexlineatus* | AY218034 | AY217984 | -- | AY217882 | AY217831 | -- | -- | -- | -- | -- | -- | -- |
| *Scincella gemmingeri* | AY308445 | AY308294 | -- | -- | -- | -- | -- | -- | -- | -- | -- | -- |
| *Scincella lateralis* | AY169598 | HM852503 | HQ655188 | AY217857 | AY217806 | JF498187 | AY169673 | -- | -- | HM161140 | HM161236 | -- |
| *Scincella reevesii* | JF497949 | AY308299 | HQ655189 | HQ655222 | -- | -- | JF498551 | -- | -- | HQ907634 | -- | -- |
| *Scincopus fasciatus* | AY649132 | AY308302 | HM160668 | -- | -- | -- | -- | -- | -- | HM161141 | HM161237 | -- |
| *Scincus mitranus* | AY649133 | AY308303 | -- | AY818806 | AF280131 | -- | -- | -- | -- | -- | -- | -- |
| *Scincus scincus* | AY218025 | AY308304 | HM160669 | AY217873 | AY217822 | -- | -- | GU456028 | -- | HM161142 | HM161238 | -- |
| *Seminatrix pygaea* | -- | -- | -- | -- | AF402920 | AF384839 | -- | -- | -- | -- | -- | -- |
| *Senticolis triaspis* | AY122816 | -- | -- | DQ902086 | AY122732 | DQ902237 | AF138775 | -- | -- | -- | -- | -- |
| *Sepsina angolensis* | AY218024 | AY217975 | -- | AY217872 | AY217821 | -- | -- | -- | -- | -- | -- | -- |
| *Shinisaurus crocodilurus* | NC_005959 | AB080274 | GU457857 | AY099976 | NC_005959 | NC_005959 | NC_005959 | GU456013 | -- | HQ876341 | AY662610 | -- |
| *Sibon nebulatus* | NC_013985 | EU728583 | -- | AF544736 | EU728583 | EU728583 | EU728583 | GQ334685 | -- | -- | -- | -- |
| *Sibynomorphus mikanii* | GQ457832 | GQ457771 | JQ599050 | GQ457892 | JQ598954 | -- | -- | -- | -- | -- | -- | -- |
| *Sibynomorphus neuwiedi* | JQ598838 | JQ598898 | -- | -- | -- | -- | -- | -- | -- | -- | -- | -- |
| *Sibynomorphus turgidus* | JQ598839 | JQ598899 | -- | -- | -- | -- | -- | -- | -- | -- | -- | -- |
| *Sibynomorphus ventrimaculatus* | JQ598840 | JQ598900 | -- | JQ598997 | -- | -- | -- | -- | -- | -- | -- | -- |
| *Sibynophis bistrigatus* | -- | -- | -- | KC000112 | KC000127 | KC000130 | -- | -- | -- | -- | KC000105 | -- |
| *Sibynophis chinensis* | -- | -- | -- | KC000113 | KC000124 | KC000131 | -- | -- | -- | -- | KC000103 | -- |
| *Sibynophis collaris* | NC_016424 | NC_016424 | -- | KC000118 | KC000121 | KC000135 | NC_016424 | -- | -- | -- | KC000107 | -- |
| *Sibynophis subpunctatus* | KC347335 | KC347373 | -- | KC347412 | KC347487 | -- | KC347526 | -- | -- | -- | KC347449 | -- |
| *Sibynophis triangularis* | -- | -- | -- | KC000116 | KC000123 | KC000132 | -- | -- | -- | -- | KC000106 | -- |
| *Sigaloseps deplanchei* | -- | -- | -- | DQ675378 | -- | DQ675238 | -- | -- | -- | -- | DQ675318 | -- |
| *Sigaloseps ruficauda* | -- | -- | -- | DQ675379 | -- | DQ675239 | -- | -- | -- | -- | DQ675319 | -- |
| *Simiscincus aurantiacus* | -- | -- | -- | DQ675389 | -- | DQ675250 | -- | -- | -- | -- | DQ675330 | -- |
| *Simoselaps anomalus* | EU547110 | EU547159 | -- | EU546924 | EU547061 | -- | EU547014 | -- | -- | -- | EU546885 | -- |
| *Simoselaps bertholdi* | EU547111 | EU547160 | -- | EU546925 | EU547062 | -- | EU547015 | -- | -- | -- | EU546886 | -- |
| *Simoselaps calonotus* | -- | -- | -- | EU546923 | -- | -- | EF210841 | -- | -- | -- | EU546884 | -- |
| *Simoselaps semifasciatus* | -- | -- | -- | EU546922 | EU547058 | -- | EU547012 | -- | -- | -- | -- | -- |
| *Sinomicrurus japonicus* | D31615 | -- | -- | AY058926 | AF217831 | -- | -- | -- | -- | -- | -- | -- |
| *Sinomicrurus kelloggi* | -- | -- | -- | EF137424 | AF220408 | -- | EF137409 | -- | -- | -- | -- | -- |
| *Sinomicrurus macclellandi* | D31616 | -- | -- | EF137425 | AF220409 | -- | EF137410 | -- | -- | -- | -- | -- |
| *Sinonatrix aequifasciata* | -- | -- | -- | JQ687440 | JQ687430 | JQ687456 | JQ687415 | -- | -- | -- | -- | -- |
| *Sinonatrix annularis* | AF544778 | HM439988 | -- | JQ687449 | JQ687431 | -- | JQ687424 | -- | -- | -- | -- | -- |
| *Sinonatrix percarinata* | -- | -- | -- | JQ687439 | GQ281784 | JQ687455 | JQ687414 | -- | -- | -- | -- | -- |
| *Siphlophis cervinus* | JQ598841 | JQ598901 | -- | JQ598998 | GQ895888 | -- | -- | -- | -- | -- | -- | -- |
| *Siphlophis compressus* | GQ457833 | GQ457772 | -- | GQ457893 | -- | -- | -- | -- | -- | -- | -- | -- |
| *Siphlophis longicaudatus* | JQ598842 | JQ598902 | -- | JQ598999 | -- | -- | -- | -- | -- | -- | -- | -- |
| *Siphlophis pulcher* | GQ457834 | GQ457773 | JQ599051 | GQ457894 | JQ598955 | -- | -- | -- | -- | -- | -- | -- |
| *Sistrurus catenatus* | HQ257513 | AF259119 | -- | JN090134 | AY223610 | -- | AF156575 | GQ334686 | -- | -- | -- | -- |
| *Sistrurus miliarius* | HQ257514 | AF259120 | -- | -- | AY223611 | -- | U41889 | -- | -- | -- | -- | -- |
| *Sitana ponticeriana* | -- | -- | -- | -- | -- | AF128481 | -- | -- | -- | -- | -- | -- |
| *Sonora semiannulata* | -- | -- | EU402659 | AF471164 | AF471048 | -- | -- | EU390934 | -- | JN703058 | EU402861 | -- |
| *Sordellina punctata* | JQ598843 | JQ598903 | JQ599052 | JQ599000 | JQ598956 | -- | -- | -- | -- | -- | -- | -- |
| *Spalerosophis diadema* | AY039144 | HQ658450 | -- | AF471155 | AF471049 | AY487020 | AY487059 | -- | -- | -- | -- | -- |
| *Spalerosophis microlepis* | AY647230 | -- | -- | -- | -- | -- | -- | -- | -- | -- | -- | -- |
| *Sphaerodactylus altavelensis* | -- | X86037 | -- | -- | -- | -- | -- | -- | -- | -- | -- | -- |
| *Sphaerodactylus argus* | -- | -- | -- | HQ426578 | -- | JX041436 | -- | -- | HQ426236 | -- | HQ426324 | HQ426497 |
| *Sphaerodactylus armstrongi* | -- | X86038 | -- | -- | -- | -- | -- | -- | -- | -- | -- | -- |
| *Sphaerodactylus cinereus* | -- | X86049 | -- | -- | -- | -- | -- | -- | -- | -- | -- | -- |
| *Sphaerodactylus copei* | -- | X86062 | -- | -- | -- | -- | -- | -- | -- | -- | -- | -- |
| *Sphaerodactylus cricoderus* | -- | X86036 | -- | -- | -- | -- | -- | -- | -- | -- | -- | -- |
| *Sphaerodactylus cryphius* | -- | X86039 | -- | -- | -- | -- | -- | -- | -- | -- | -- | -- |
| *Sphaerodactylus darlingtoni* | -- | X86040 | -- | -- | -- | -- | -- | -- | -- | -- | -- | -- |
| *Sphaerodactylus elegans* | -- | X86048 | -- | EF534912 | -- | JN393942 | -- | -- | EF534828 | -- | EF534787 | EF534954 |
| *Sphaerodactylus elegantulus* | -- | X86057 | -- | -- | AF525911 | -- | -- | -- | -- | -- | -- | -- |
| *Sphaerodactylus fantasticus* | EU191670 | X86058 | -- | -- | FJ404596 | -- | -- | -- | -- | -- | -- | -- |
| *Sphaerodactylus gaigeae* | -- | X86042 | -- | -- | -- | -- | -- | -- | -- | -- | -- | -- |
| *Sphaerodactylus glaucus* | -- | -- | -- | HQ426579 | -- | JX041437 | -- | -- | HQ426237 | -- | HQ426325 | HQ426498 |
| *Sphaerodactylus goniorhynchus* | -- | X86041 | -- | -- | -- | -- | -- | -- | -- | -- | -- | -- |
| *Sphaerodactylus intermedius* | -- | X86050 | -- | -- | -- | -- | -- | -- | -- | -- | -- | -- |
| *Sphaerodactylus kirbyi* | -- | -- | -- | -- | AF525910 | -- | -- | -- | -- | -- | -- | -- |
| *Sphaerodactylus klauberi* | -- | X86046 | -- | HQ426581 | -- | -- | -- | -- | HQ426239 | -- | HQ426327 | HQ426500 |
| *Sphaerodactylus leucaster* | -- | X86056 | -- | -- | -- | -- | -- | -- | -- | -- | -- | -- |
| *Sphaerodactylus macrolepis* | -- | X86047 | -- | HQ426580 | -- | -- | -- | -- | HQ426238 | -- | HQ426326 | HQ426499 |
| *Sphaerodactylus microlepis* | -- | -- | -- | -- | FJ404597 | -- | -- | -- | -- | -- | -- | -- |
| *Sphaerodactylus molei* | GU139949 | -- | -- | GU139843 | -- | -- | -- | GU139896 | -- | -- | -- | -- |
| *Sphaerodactylus nicholsi* | -- | X86043 | -- | HQ426582 | -- | JX041438 | -- | -- | HQ426240 | -- | HQ426328 | HQ426501 |
| *Sphaerodactylus nigropunctatus* | -- | X86051 | -- | EF534911 | -- | JX041439 | -- | -- | EF534827 | -- | HQ426329 | EF534953 |
| *Sphaerodactylus notatus* | -- | X86061 | -- | EF564067 | -- | -- | -- | -- | HQ426241 | -- | HQ426330 | EF564093 |
| *Sphaerodactylus ocoae* | -- | -- | -- | EF534910 | -- | -- | -- | -- | EF534826 | -- | EF534786 | EF534952 |
| *Sphaerodactylus oliveri* | -- | X86053 | -- | -- | -- | -- | -- | -- | -- | -- | -- | -- |
| *Sphaerodactylus parvus* | -- | -- | -- | -- | AF525916 | -- | -- | -- | -- | -- | -- | -- |
| *Sphaerodactylus ramsdeni* | -- | X86034 | -- | -- | -- | -- | -- | -- | -- | -- | -- | -- |
| *Sphaerodactylus richardi* | -- | X86054 | -- | -- | -- | -- | -- | -- | -- | -- | -- | -- |
| *Sphaerodactylus roosevelti* | DQ852713 | -- | -- | EF534909 | -- | JN393943 | -- | -- | EF534825 | -- | EF534785 | EF534951 |
| *Sphaerodactylus sabanus* | EU191623 | -- | -- | -- | EU191671 | -- | -- | -- | -- | -- | -- | -- |
| *Sphaerodactylus schwartzi* | -- | X86035 | -- | -- | -- | -- | -- | -- | -- | -- | -- | -- |
| *Sphaerodactylus semasiops* | -- | X86045 | -- | -- | -- | -- | -- | -- | -- | -- | -- | -- |
| *Sphaerodactylus shrevei* | -- | -- | -- | AY662570 | -- | AY662547 | -- | -- | -- | -- | AY662623 | -- |
| *Sphaerodactylus sputator* | EU191624 | X86059 | -- | -- | EU191672 | -- | -- | -- | -- | -- | -- | -- |
| *Sphaerodactylus thompsoni* | -- | X86063 | -- | -- | -- | -- | -- | -- | -- | -- | -- | -- |
| *Sphaerodactylus torrei* | -- | X86052 | -- | EF534913 | -- | JX041440 | -- | -- | EF534829 | -- | EF534788 | EF534955 |
| *Sphaerodactylus townsendi* | -- | -- | -- | HQ426583 | -- | -- | -- | -- | HQ426242 | -- | HQ426331 | HQ426502 |
| *Sphaerodactylus vincenti* | -- | -- | -- | -- | FJ404649 | -- | -- | -- | -- | -- | -- | -- |
| *Sphenodon punctatus* | L28076 | L28076 | GU457846 | AF039483 | NC_004815 | NC_004815 | NC_004815 | GU456002 | HQ426257 | HQ876320 | AY662576 | HQ426516 |
| *Sphenomorphus abdictus* | JF497920 | JF498048 | -- | -- | -- | GU573562 | JF498522 | -- | -- | HQ907632 | -- | -- |
| *Sphenomorphus acutus* | JF497950 | JF498079 | -- | -- | -- | JF498188 | JF498552 | -- | -- | JF498431 | -- | -- |
| *Sphenomorphus aesculeticola* | JF497970 | JF498100 | -- | -- | -- | JF498208 | JF498570 | -- | -- | JF498452 | -- | -- |
| *Sphenomorphus arborens* | JF497863 | JF497986 | -- | -- | -- | JF498113 | JF498467 | -- | -- | JF498341 | -- | -- |
| *Sphenomorphus assatus* | JF497946 | JF498074 | -- | -- | -- | JF498186 | JF498548 | -- | -- | JF498428 | -- | -- |
| *Sphenomorphus atrigularis* | JF497972 | JF498102 | -- | -- | -- | JF498210 | JF498572 | -- | -- | JF498454 | -- | -- |
| *Sphenomorphus beyeri* | JF497878 | JF498002 | -- | -- | -- | JF498130 | JF498481 | -- | -- | JF498357 | -- | -- |
| *Sphenomorphus buenloicus* | -- | HM773218 | -- | -- | -- | -- | -- | -- | -- | -- | -- | -- |
| *Sphenomorphus cherriei* | AB057377 | AB057392 | -- | -- | GQ450315 | -- | JF498550 | -- | -- | JF498429 | -- | -- |
| *Sphenomorphus concinnatus* | JF497951 | JF498080 | -- | -- | -- | JF498190 | JF498554 | -- | -- | JF498432 | -- | -- |
| *Sphenomorphus coxi* | JF497928 | JF498056 | -- | -- | -- | -- | JF498530 | -- | -- | JF498412 | -- | -- |
| *Sphenomorphus cranei* | JF497952 | JF498082 | -- | -- | -- | JF498192 | JF498556 | -- | -- | JF498434 | -- | -- |
| *Sphenomorphus cumingi* | JF497873 | JF497998 | -- | -- | -- | JF498126 | JF498477 | -- | -- | JF498353 | -- | -- |
| *Sphenomorphus cyanolaemus* | JF497954 | JF498084 | -- | -- | -- | JF498193 | JF498557 | -- | -- | JF498436 | -- | -- |
| *Sphenomorphus decipiens* | JF497890 | JF498014 | -- | -- | -- | JF498144 | JF498496 | -- | -- | JF498371 | -- | -- |
| *Sphenomorphus diwata* | JF497956 | JF498086 | -- | -- | -- | JF498194 | JF498558 | -- | -- | JF498438 | -- | -- |
| *Sphenomorphus fasciatus* | JF497958 | JF498088 | -- | DQ675380 | AF373234 | DQ675240 | JF498560 | -- | -- | JF498440 | DQ675320 | -- |
| *Sphenomorphus hallieri* | JF497973 | JF498104 | -- | -- | -- | JF498212 | JF498574 | -- | -- | JF498455 | -- | -- |
| *Sphenomorphus indicus* | AB028808 | AB028820 | -- | -- | -- | JF498198 | JF498562 | -- | -- | JF498441 | -- | -- |
| *Sphenomorphus jagori* | JF497932 | JF498060 | -- | -- | -- | JF498180 | JF498540 | -- | -- | JF498416 | -- | -- |
| *Sphenomorphus jobiensis* | DQ915291 | AY308306 | HQ655190 | DQ675395 | AF151648 | DQ675258 | DQ915339 | -- | -- | -- | -- | -- |
| *Sphenomorphus kitangladensis* | JF497894 | JF498022 | -- | -- | -- | JF498150 | JF498500 | -- | -- | JF498377 | -- | -- |
| *Sphenomorphus laterimaculatus* | JF497896 | JF498024 | -- | -- | -- | JF498152 | JF498502 | -- | -- | JF498379 | -- | -- |
| *Sphenomorphus lawtoni* | JF497898 | JF498025 | -- | -- | -- | JF498153 | JF498504 | -- | -- | JF498381 | -- | -- |
| *Sphenomorphus leptofasciatus* | -- | -- | -- | AF039464 | AF373233 | AF373263 | -- | -- | -- | -- | -- | -- |
| *Sphenomorphus leucospilos* | JF497900 | JF498026 | -- | -- | -- | JF498154 | JF498506 | -- | -- | JF498383 | -- | -- |
| *Sphenomorphus llanosi* | JF497940 | JF498068 | -- | -- | -- | -- | JF498542 | -- | -- | JF498424 | -- | -- |
| *Sphenomorphus luzonense* | JF497901 | JF498028 | -- | -- | -- | JF498156 | JF498508 | -- | -- | JF498384 | -- | -- |
| *Sphenomorphus maculatus* | AY308461 | AY308310 | -- | -- | -- | JF498199 | JF498563 | -- | -- | JF498442 | -- | -- |
| *Sphenomorphus maindroni* | AY308462 | AY308311 | -- | -- | -- | -- | -- | -- | -- | -- | -- | -- |
| *Sphenomorphus melanopogon* | AY308463 | AY308312 | -- | -- | -- | -- | -- | -- | -- | -- | -- | -- |
| *Sphenomorphus mindanensis* | JF497942 | JF498070 | -- | -- | -- | JF498184 | JF498544 | -- | -- | JF498426 | -- | -- |
| *Sphenomorphus muelleri* | AY169599 | AY169636 | -- | -- | -- | -- | AY169674 | -- | -- | -- | -- | -- |
| *Sphenomorphus multisquamatus* | JF497961 | JF498091 | -- | -- | -- | JF498200 | JF498564 | -- | -- | JF498443 | -- | -- |
| *Sphenomorphus parvus* | JF497974 | JF498106 | -- | -- | -- | JF498214 | JF498576 | -- | -- | JF498456 | -- | -- |
| *Sphenomorphus praesignis* | AB028810 | AB028822 | -- | -- | -- | -- | -- | -- | -- | -- | -- | -- |
| *Sphenomorphus sabanus* | AY308465 | AY308314 | -- | -- | -- | JF498201 | JF498565 | -- | -- | JF498444 | -- | -- |
| *Sphenomorphus scutatus* | JF497963 | JF498093 | -- | -- | -- | JF498202 | JF498566 | -- | -- | JF498445 | -- | -- |
| *Sphenomorphus simus* | AY218017 | AY217967 | EU108365 | EU116688 | EU116518 | -- | -- | EU108031 | -- | -- | EU108539 | -- |
| *Sphenomorphus solomonis* | DQ915304 | AY308316 | HQ655191 | HQ655224 | -- | JF498204 | DQ915352 | JN568332 | -- | JF498446 | JN654864 | -- |
| *Sphenomorphus steerei* | JF497904 | JF498032 | -- | -- | -- | JF498160 | JF498510 | -- | -- | JF498400 | -- | -- |
| *Sphenomorphus stellatus* | -- | HM773221 | -- | -- | -- | -- | -- | -- | -- | -- | -- | -- |
| *Sphenomorphus tagapayo* | JF497918 | JF498046 | -- | -- | -- | JF498174 | JF498520 | -- | -- | JF498402 | -- | -- |
| *Sphenomorphus variegatus* | JF497966 | JF498096 | -- | -- | -- | JF498206 | JF498569 | -- | -- | JF498448 | -- | -- |
| *Sphenomorphus victoria* | -- | JF497989 | -- | -- | -- | JF498117 | -- | -- | -- | JF498345 | -- | -- |
| *Sphenomorphus wrighti* | JF497867 | JF497990 | -- | -- | -- | JF498119 | JF498471 | -- | -- | JF498347 | -- | -- |
| *Spilotes pullatus* | HM565768 | -- | -- | AF471110 | AF471041 | -- | -- | -- | -- | -- | -- | -- |
| *Stenocercus angel* | -- | -- | -- | -- | -- | DQ080212 | -- | -- | -- | -- | -- | -- |
| *Stenocercus angulifer* | -- | -- | -- | -- | -- | EF565142 | -- | -- | -- | -- | -- | -- |
| *Stenocercus apurimacus* | -- | -- | -- | -- | -- | DQ080213 | -- | -- | -- | -- | -- | -- |
| *Stenocercus azureus* | -- | -- | -- | -- | -- | DQ080214 | -- | -- | -- | -- | -- | -- |
| *Stenocercus boettgeri* | -- | -- | -- | -- | -- | DQ080215 | -- | -- | -- | -- | -- | -- |
| *Stenocercus caducus* | -- | -- | -- | -- | -- | DQ080216 | -- | -- | -- | -- | -- | -- |
| *Stenocercus chota* | -- | -- | -- | -- | -- | DQ080217 | -- | -- | -- | -- | -- | -- |
| *Stenocercus chrysopygus* | -- | -- | -- | -- | -- | EF565141 | -- | -- | -- | -- | -- | -- |
| *Stenocercus crassicaudatus* | -- | -- | -- | -- | -- | AF049866 | -- | -- | -- | -- | AY662597 | -- |
| *Stenocercus cupreus* | -- | -- | -- | -- | -- | DQ080218 | -- | -- | -- | -- | -- | -- |
| *Stenocercus doellojuradoi* | -- | -- | -- | -- | -- | AF528744 | -- | -- | -- | -- | -- | -- |
| *Stenocercus empetrus* | -- | -- | -- | -- | -- | DQ080219 | -- | -- | -- | -- | -- | -- |
| *Stenocercus eunetopsis* | -- | -- | -- | -- | -- | DQ080220 | -- | -- | -- | -- | -- | -- |
| *Stenocercus festae* | -- | -- | -- | -- | -- | DQ080221 | -- | -- | -- | -- | -- | -- |
| *Stenocercus formosus* | -- | -- | -- | -- | -- | DQ080238 | -- | -- | -- | -- | -- | -- |
| *Stenocercus guentheri* | -- | L41481 | HQ876224 | -- | -- | DQ080223 | -- | JF804570 | -- | HQ876337 | HQ876440 | -- |
| *Stenocercus humeralis* | -- | -- | -- | -- | -- | DQ080224 | -- | -- | -- | -- | -- | -- |
| *Stenocercus imitator* | -- | -- | -- | -- | -- | DQ080225 | -- | -- | -- | -- | -- | -- |
| *Stenocercus iridescens* | -- | -- | -- | -- | -- | DQ080226 | -- | -- | -- | -- | -- | -- |
| *Stenocercus latebrosus* | -- | -- | -- | -- | -- | DQ080227 | -- | -- | -- | -- | -- | -- |
| *Stenocercus limitaris* | -- | -- | -- | -- | -- | DQ080228 | -- | -- | -- | -- | -- | -- |
| *Stenocercus marmoratus* | -- | -- | -- | -- | -- | EF565143 | -- | -- | -- | -- | -- | -- |
| *Stenocercus melanopygus* | -- | -- | -- | -- | -- | DQ080229 | -- | -- | -- | -- | -- | -- |
| *Stenocercus ochoai* | -- | -- | -- | -- | -- | AF528746 | -- | -- | -- | -- | -- | -- |
| *Stenocercus orientalis* | -- | -- | -- | -- | -- | DQ080230 | -- | -- | -- | -- | -- | -- |
| *Stenocercus ornatissimus* | -- | -- | -- | -- | -- | DQ080240 | -- | -- | -- | -- | -- | -- |
| *Stenocercus ornatus* | -- | -- | -- | -- | -- | DQ080231 | -- | -- | -- | -- | -- | -- |
| *Stenocercus percultus* | -- | -- | -- | -- | -- | DQ080232 | -- | -- | -- | -- | -- | -- |
| *Stenocercus puyango* | -- | -- | -- | -- | -- | DQ080233 | -- | -- | -- | -- | -- | -- |
| *Stenocercus rhodomelas* | -- | -- | -- | -- | -- | DQ080234 | -- | -- | -- | -- | -- | -- |
| *Stenocercus roseiventris* | AF362522 | -- | -- | -- | -- | DQ080235 | -- | -- | -- | -- | -- | -- |
| *Stenocercus scapularis* | -- | -- | -- | -- | -- | DQ080222 | -- | -- | -- | -- | -- | -- |
| *Stenocercus stigmosus* | -- | -- | -- | -- | -- | DQ080236 | -- | -- | -- | -- | -- | -- |
| *Stenocercus torquatus* | -- | -- | -- | -- | -- | DQ080239 | -- | -- | -- | -- | -- | -- |
| *Stenocercus varius* | -- | -- | -- | -- | -- | DQ080237 | -- | -- | -- | -- | -- | -- |
| *Stenodactylus arabicus* | -- | -- | HQ443599 | -- | -- | HQ443552 | -- | -- | -- | -- | -- | -- |
| *Stenodactylus doriae* | -- | -- | HQ443640 | AF148707 | -- | HQ443527 | -- | -- | -- | -- | -- | -- |
| *Stenodactylus khobarensis* | -- | -- | HQ443614 | -- | -- | HQ443551 | -- | -- | -- | -- | -- | -- |
| *Stenodactylus leptocosymbotus* | -- | -- | HQ443631 | -- | -- | HQ443536 | -- | -- | -- | -- | -- | -- |
| *Stenodactylus petrii* | DQ852722 | -- | HQ443622 | DQ852738 | -- | HQ443558 | -- | -- | -- | -- | -- | -- |
| *Stenodactylus sthenodactylus* | -- | -- | HQ443604 | JQ945617 | -- | HQ443546 | -- | -- | JQ945408 | -- | JQ945339 | JQ945510 |
| *Stenodactylus yemenensis* | -- | -- | HQ443609 | -- | -- | HQ443550 | -- | -- | -- | -- | -- | -- |
| *Stenolepis ridleyi* | EF405618 | EF405619 | -- | EF405622 | -- | -- | EF405620 | -- | -- | -- | -- | -- |
| *Stenophis betsileanus* | FJ404190 | -- | -- | AY187998 | AY188037 | -- | FJ404387 | -- | -- | -- | -- | -- |
| *Stenophis citrinus* | FJ404191 | AY611865 | -- | AY611956 | AY612047 | -- | FJ404388 | -- | -- | -- | -- | -- |
| *Stenophis granuliceps* | -- | GU994849 | -- | GU994824 | GU994799 | -- | -- | -- | -- | -- | -- | -- |
| *Stenophis inopinae* | -- | GU994850 | -- | GU994825 | GU994800 | -- | -- | -- | -- | -- | -- | -- |
| *Stenophis inornatus* | -- | GU994859 | -- | GU994834 | GU994809 | -- | -- | -- | -- | -- | -- | -- |
| *Stenophis pseudogranuliceps* | -- | GU994858 | -- | AY187999 | AY188038 | -- | -- | -- | -- | -- | -- | -- |
| *Stenorrhina freminvillei* | HM565769 | -- | -- | GQ895830 | GQ895889 | -- | -- | -- | -- | -- | -- | -- |
| *Stoliczkaia borneensis* | AF544779 | AF544808 | FJ433982 | AF544721 | -- | -- | -- | FJ434083 | -- | EF144066 | AY487398 | EF144094 |
| *Storeria dekayi* | AF402639 | JQ598904 | JQ599053 | AF471154 | AF471050 | AF384841 | EF417365 | -- | -- | JF946386 | -- | -- |
| *Storeria occipitomaculata* | AF402638 | -- | -- | -- | AF402921 | AF384840 | U49323 | -- | -- | -- | -- | -- |
| *Strobilurus torquatus* | AF362523 | -- | -- | -- | -- | -- | -- | -- | -- | -- | -- | -- |
| *Strophurus assimilis* | -- | -- | -- | -- | -- | AY368999 | -- | -- | -- | -- | -- | -- |
| *Strophurus ciliaris* | AY583856 | AY583928 | GU457865 | -- | AY583945 | AY368996 | JQ398491 | GU456021 | JQ173716 | HQ876375 | JQ173762 | JQ945511 |
| *Strophurus elderi* | -- | -- | -- | JQ945618 | -- | AY369000 | -- | -- | JQ173717 | -- | JQ173763 | JQ945512 |
| *Strophurus intermedius* | AF090180 | GU460151 | -- | AF039469 | -- | AY369001 | -- | -- | -- | -- | FJ571625 | -- |
| *Strophurus jeanae* | -- | -- | -- | FJ855477 | -- | -- | -- | -- | -- | -- | FJ855456 | -- |
| *Strophurus krisalys* | AY583883 | AY583926 | -- | -- | AY583957 | -- | -- | -- | -- | -- | -- | -- |
[truncated: 115,823 more chars]
